# 1,4,6,10-Tetraazaadamantanes (TAADs) with *N*-amino groups: synthesis and formation of boron chelates and host–guest complexes

**DOI:** 10.3762/bjoc.18.148

**Published:** 2022-10-11

**Authors:** Artem N Semakin, Ivan S Golovanov, Yulia V Nelyubina, Alexey Yu Sukhorukov

**Affiliations:** 1 Laboratory of organic and metal-organic nitrogen-oxygen systems, N. D. Zelinsky Institute of Organic Chemistry, Russian Academy of Sciences, Leninsky Prospect, 47, Moscow, 119991, Russian Federation; 2 Center for molecular composition studies, A. N. Nesmeyanov Institute of Organoelement Compounds, Russian Academy of Sciences, 119991, Vavilova str. 28, Moscow, Russian Federation

**Keywords:** azaadamantanes, cyclotrimerization, hydrazones, inclusion complexes, molecular recognition

## Abstract

A synthetic route to 1,4,6,10-tetraazaadamantanes (TAADs) bearing free and protected amino groups at the bridge *N*-atoms has been developed via intramolecular cyclotrimerization of C=N units in the corresponding tris(hydrazonoalkyl)amines. In a similar fashion, unsymmetrically substituted TAADs having both amino and hydroxy groups at the bridge *N*-atoms were prepared via a hitherto unknown co-trimerization of oxime and hydrazone groups. The use of *N*-TAAD derivatives as potential ligands and receptors was showcased through forming boron chelates and host–guest complexes with water and simple alcohols.

## Introduction

**Figure 1:**
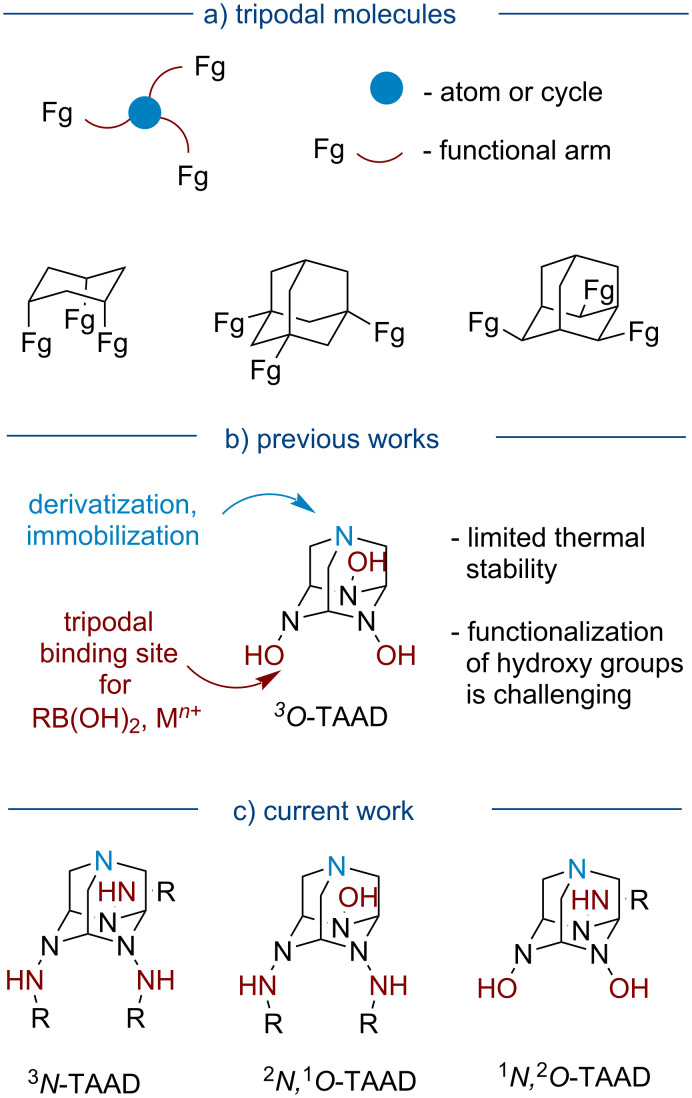
Adamantane-based tripodal scaffolds and current work.

Tripodal molecules are widely used as chelating ligands for transition metal catalysis [1–3], sensors for ions and small molecules [4–5], reagents for surface grafting [6], building blocks for the construction of supramolecular structures [7], polycyclic cage molecules [8], and porous materials [9]. In tripodal molecules, three functional arms are bound to a central trivalent scaffold, which can be a single atom (e.g., carbon, nitrogen, or phosphorus), a small ring (usually aromatic or saturated six-membered ring), or a macrocycle (Figure 1a). The functional properties of tripodal molecules are governed not only by the nature and length of the side arms but also by the size and geometry of the central scaffold [2,4]. By changing the scaffold, the volume of the cavity created by the three arms can be tuned allowing for the coordination of ions and molecules of different sizes [4]. For this reason, the central scaffold should also have a rigid geometry without much conformational flexibility to create a preorganized binding pocket. In this regard, the adamantane scaffold received some attention as it represents a conformationally strained analog of the cyclohexane ring [10–13]. However, the preparation of trisubstituted adamantanes is challenging because multiple functionalization of the parent hydrocarbon is not selective, while the synthesis from acyclic precursors requires multistep synthetic routes [10,14]. The use of azaadamantanes is more advantageous as they are prepared via self-assembly reactions from simple precursors and easily functionalized through addition reactions at heteroatoms [15–20].

Some time ago, our group introduced 1,4,6,10-tetraazaadamantane (TAAD) as a promising molecular platform for the design of functional tripodal molecules (Figure 1b) [21–23]. In particular, *N*,*N*,*N*-trihydroxy-TAAD derivatives (^3^*O*-TAADs) were shown to be chelating ligands for boron [24]. The application of TAAD-boronate complexes was demonstrated by the preparation of conjugates of boronic acids with biomolecules [25–26], COF-like materials [25], and dynamic covalent libraries [25]. TAAD can be covalently bound to a polymer matrix through the nucleophilic nitrogen N(1) that was used to prepare scavengers of boronic acids [25]. Also, TAAD was demonstrated to serve as a scorpionate-type ligand for manganese and iron leading to complexes with the metal in an atypical +4 oxidation state [27–29]. Recently, TAAD was used to improve the thermal and photochemical stability of MAPbI_3_ perovskite films (most likely through coordination of Pb(II) ions) [30]. The disadvantages of ^3^*O*-TAADs, however, are limited thermal stability (due to cage opening to corresponding linear tris-oximes) and difficulties in the functionalization of *N*-hydroxy groups [21].

Currently, our research is focusing on TAAD derivatives bearing protected and free amino groups at the bridge nitrogen atoms (*N*-TAADs, Figure 1c). Our main interest in these products was due to their potential as synthetic receptors to form host–guest complexes based on H-bond interactions with amide/carbamoyl groups [31–32]. In addition, *N*-TAADs can be viewed as analogs of hexahydrazide ligands, which were reported to stabilize high-valent metal complexes [33–34]. Since *N*-amino groups can be easily functionalized through the addition of electrophilic reagents, various functional arms can be attached. Moreover, *N*-TAAD derivatives were expected to be less prone to cage-opening reaction compared to ^3^*O*-TAADs.

Here, we wish to report the synthesis of TAADs decorated with *N*-amino(amido) groups (^3^*N*-TAADs) by cyclization of the corresponding tris-hydrazones, as well as the assembly of unsymmetrically substituted TAADs having both amino(amido) and hydroxy groups at bridge nitrogen atoms (^2^*N*,^1^*O*-TAADs and ^1^*N*,^2^*O*-TAADs) via a hitherto unknown co-trimerization of oxime and hydrazone units [35]. Also, structural studies of the obtained TAAD derivatives were performed, and the formation of boron chelates and host–guest complexes having an unusual intramolecular H-bonded network was showcased in this work.

## Results and Discussion

### Synthesis of ^3^*N*-TAADs, ^2^*N*,^1^*O*-TAADs and ^1^*N*,^2^*O*-TAADs

**Scheme 1:**
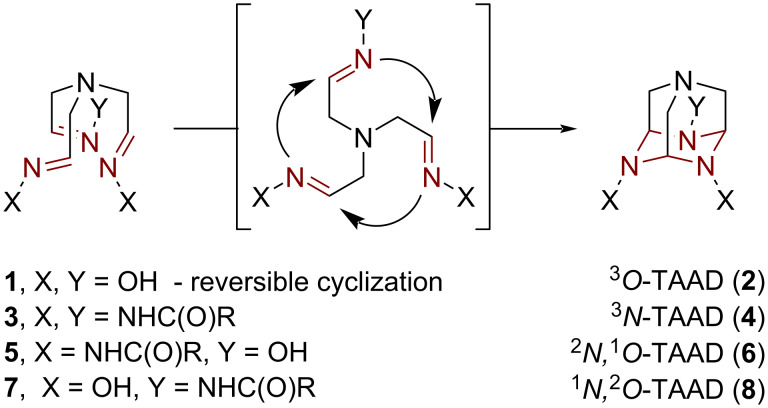
A general strategy for the assembly of TAAD derivatives.

Our strategy to construct TAAD derivatives involves the intramolecular cyclotrimerization of C=N bonds in a suitable trisimine precursor (Scheme 1). Previously, ^3^*O*-TAADs **2** were prepared by cyclization of corresponding trisoximes **1** [21]. However, this reaction is slow and reversible (upon heating adamantane structure **2** reverts to the tris-oxime form **1**). Previous experimental and computational data evidence that cyclotrimerization of hydrazone groups proceeds more readily compared to oximes [18]. Hence, hydrazones **3** were expected to cyclize to corresponding TAADs **4** with high efficiency. On the other hand, cyclization of mixed oxime-hydrazone acyclic precursors **5** and **7** to give ^2^*N,*^1^*O*-TAADs (**6**) and ^1^*N,*^2^*O*-TAADs (**8**) was ambiguous as no examples of co-trimerization of hydrazone and oxime groups (either inter- or intramolecular) have been reported to the best of our knowledge.

**Scheme 2:**
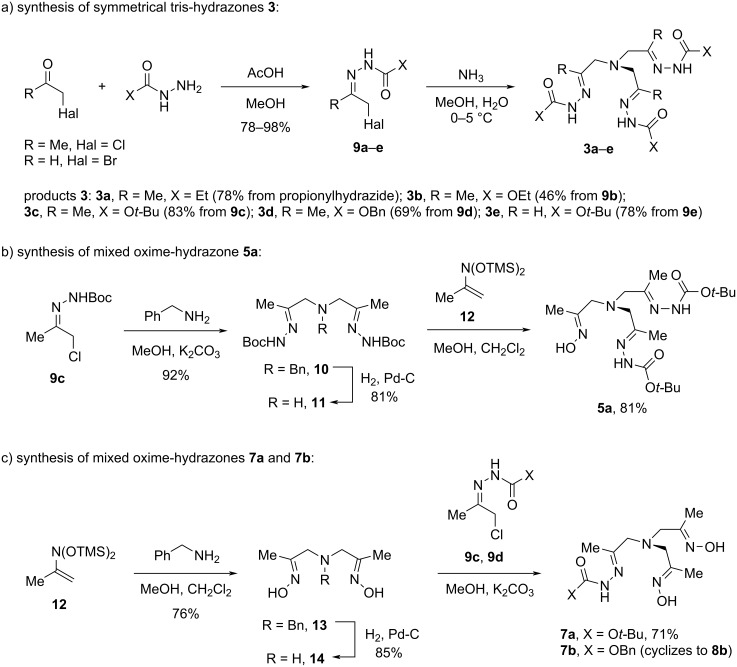
Synthesis of acyclic precursors to ^3^*N*-TAADs, ^2^*N,*^1^*O*-TAADs, and ^1^*N,*^2^*O*-TAADs.

Initially, a series of trishydrazones **3a**–**e** were synthesized by a triple alkylation of ammonia with α-halohydrazones **9a**–**e** following a protocol previously developed by us [35] (Scheme 2a). Halohydrazones **9a**–**e** were prepared by condensation of readily available chloroacetone or bromoacetaldehyde (generated from the corresponding diethyl acetal) with hydrazides or carbazates. In the synthesis of product **3a** the intermediate α-chlorohydrazone **9a** was not isolated.

The preparation of mixed oxime-hydrazones **5** and **7** was more challenging as it required a modular synthetic approach and the use of protecting groups. The developed synthetic route starting from benzylamine is shown in Scheme 2b and c. To access mixed oxime-hydrazone **5a**, benzylamine was reacted with two equivalents of α-chlorohydrazone **9c** to give bishydrazone **10**, which was debenzylated by hydrogenolysis with Pd–C catalyst. The subsequent introduction of the oximinoalkyl unit was selectively achieved by treatment of secondary amine **11** with ene-nitrosoacetal **12** [36] (Scheme 2b). The synthesis of products **7a**,**b** containing two oxime groups was accomplished via double oximinoalkylation of benzylamine to give dioxime **13** [36], cleavage of the *N*-benzyl group, and reaction of the secondary amine **14** with α-chlorohydrazones **9c**,**d** (Scheme 2c).

**Scheme 3:**
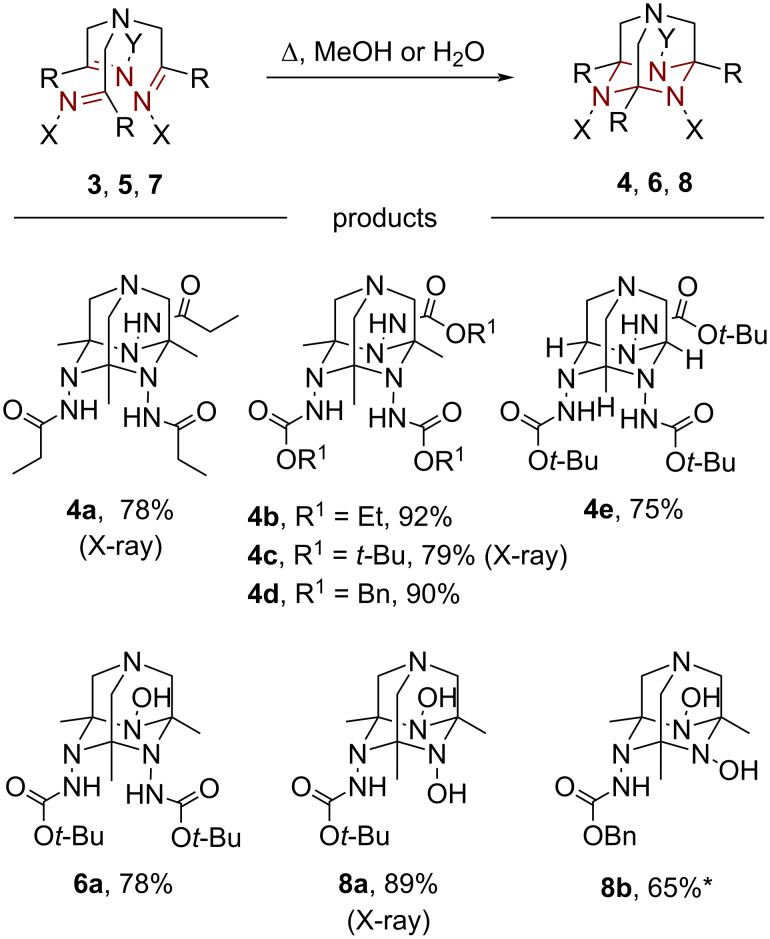
Synthesis of ^3^*N*-TAADs, ^2^*N,*^1^*O*-TAADs, and ^1^*N,*^2^*O*-TAADs. *Yield based on compound **14**.

We then studied the cyclization of the obtained trishydrazones **3** and mixed oxime-hydrazones **5** and **7** to the corresponding TAAD derivatives **4**, **6**, and **8** (Scheme 3). Upon reflux in methanol, tris-hydrazones **3a**,**c**–**e** underwent smooth intramolecular [2 + 2 + 2]-cyclization to give the corresponding TAADs **4a**,**c**–**e** in good to high isolated yields. Trishydrazone **3b** with X = NHCO_2_Et was somewhat less reactive, and its conversion to heteroadamantane **4b** required more harsh conditions (reflux in water). TAADs **4a** and **4c** were converted into their corresponding hydrochlorides, which were used for X-ray analysis (vide infra).

Similar to trishydrazones **3**, the mixed oxime-hydrazones **5** and **7** cyclized to the corresponding heteroadamantanes **6** and **8** smoothly. Moreover, compound **7b** underwent partial cyclization to heteroadamantane **8b** already upon preparation. This is surprising as the parent trisoximes **1** cyclized only in the presence of acid as a promoter [21], while with trishydrazones **3** the process takes place only at elevated temperatures. Thus, a synergistic effect of the oxime and hydrazone groups on the intramolecular cyclotrimerization reaction takes place. The structure of ^1^*N,*^2^*O*-TAAD **8a** was secured by X-ray analysis (vide infra).

Unlike ^3^*O*-TAAD derivatives **2** [21], the obtained ^3^*N*-TAADs, ^2^*N,*^1^*O*-TAADs, and ^1^*N,*^2^*O*-TAADs are thermally stable and do not suffer from retro-[2 + 2 + 2]-cyclotrimerization to the open-chain tris-imines upon heating. Thus, the presence of at least one *N*-amido group stabilizes the azaadamantane form making it more preferable under thermodynamic conditions.

### Transformations of ^3^*N*-TAADs, ^2^*N*,^1^*O*-TAADs, and ^1^*N*,^2^*O*-TAADs

**Scheme 4:**
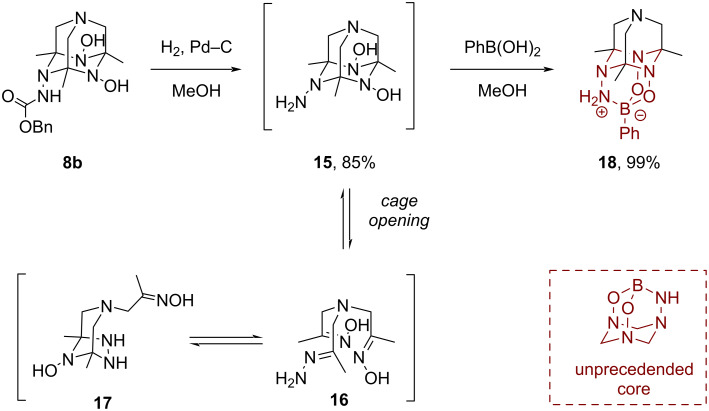
Deprotection of TAAD **8b** and subsequent complexation with phenylboronic acid.

We then focused on the preparation of TAAD derivatives with free amino groups by the removal of the protecting *N*-acyl or alkoxycarbonyl groups. However, treatment of adamantanes **4a** and **4b** with excess hydrazine (100–200 equiv) upon heating did not lead to any conversion. Also attempts to deprotect Boc-derivatives **4c**, **4e**, **6a**, and **8a** with trifluoroacetic acid or hydrochloric acid (in water or dioxane) were not successful and led to complex mixtures of products. Hydrazinium dihydrochloride was isolated in some cases (confirmed by X-ray, mp, and FT-IR data) demonstrating the degradation of the heteroadamantane cage. Deprotection of Cbz derivatives by hydrogenation over Pd–C was more productive. Thus, hydrogenolysis of product **8b** delivered the ^1^*N,*^2^*O*-TAAD derivative **15** bearing a free amino group (Scheme 4). However, this product was prone to a ring–chain isomerization forming an equilibrating mixture of azaadamantane **15**, tris-imine **16**, and bicycle **17** in solution as determined by NMR data (see Supporting Information File 1 for details). We were able to shift the equilibrium to the adamantane form by forming a stable boronate adduct **18** having a diamantane structure through the reaction with phenylboronic acid. Note that product **18** contains an unprecedented 2,4-dioxa-1,5,7,10-tetraaza-3-boraadamantane motif (Scheme 4). This result demonstrates that *N*-amino-substituted TAADs are able to serve as chelating ligands. In recent years, cyclic boron ate-complexes have received much interest as protected forms of boronic acids [37–39].

**Scheme 5:**
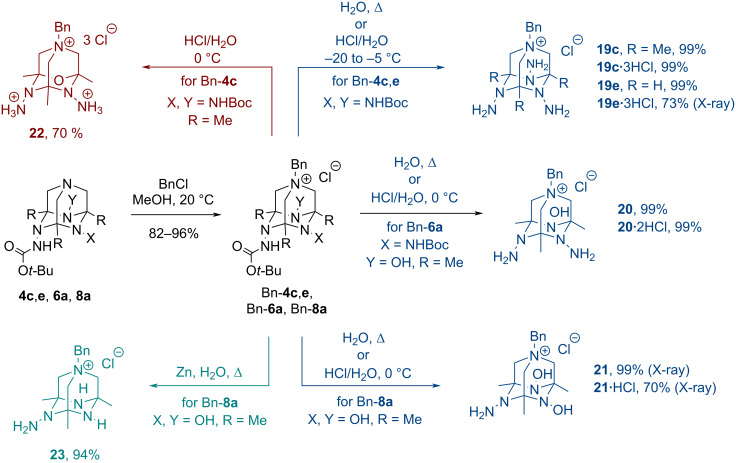
Quaternization of TAADs **4c**, **4e**, **6a**, and **8a** followed by deprotection of *N*-Boc groups.

From this experiment, it became apparent that TAAD derivatives bearing free *N*-amino groups are prone to cage opening. To stabilize the tetraazaadamantane cage, quaternization of the tertiary bridge-head nitrogen in Boc-protected TAADs **4c**, **4e**, **6a**, and **8a** was performed by benzylation [21,40] (Scheme 5). The obtained *N*-benzyl salts were quantitatively converted into corresponding deprotected TAADs **19**–**21** by reflux in water (Scheme 5, blue). Alternatively, removal of Boc groups could be performed by treatment with aqueous HCl to give the corresponding hydrochloride salts, which could be converted into free hydrazines by treatment with excess NaHCO_3_ in ethanol (as demonstrated for product **19c**). Note that TAADs **19**–**21** with free amino groups are more stable than the corresponding hydrochloride salts, which slowly decompose upon storage at rt. The structures of products **19e**·3HCl, **21** and its hydrochloride **21**·HCl were confirmed by X-ray diffraction analysis (vide infra). In hydrochloride salts, the primary amino groups were protonated.

In most cases, deprotection reactions were performed at low temperatures to prevent degradation of the aminal fragments. Indeed, treatment of Bn-**4c** with hydrochloric acid at 0 °C resulted in a substitution of one NNH_2_ unit with the oxygen atom producing product **22**, having a hitherto undescribed 4-oxa-1,6,10-triazaadamantane skeleton (Scheme 5, red). Also, hydrazinium dihydrochloride was isolated in this experiment.

Deoxygenation of N–OH units in TAAD Bn-**8a** was also performed by reflux with Zn in water (Scheme 5, green). The Boc group was removed under these conditions producing TAAD **23** in high yield.

### Structure of *N*-TAADs and the formation of host–guest complexes

**Figure 2:**
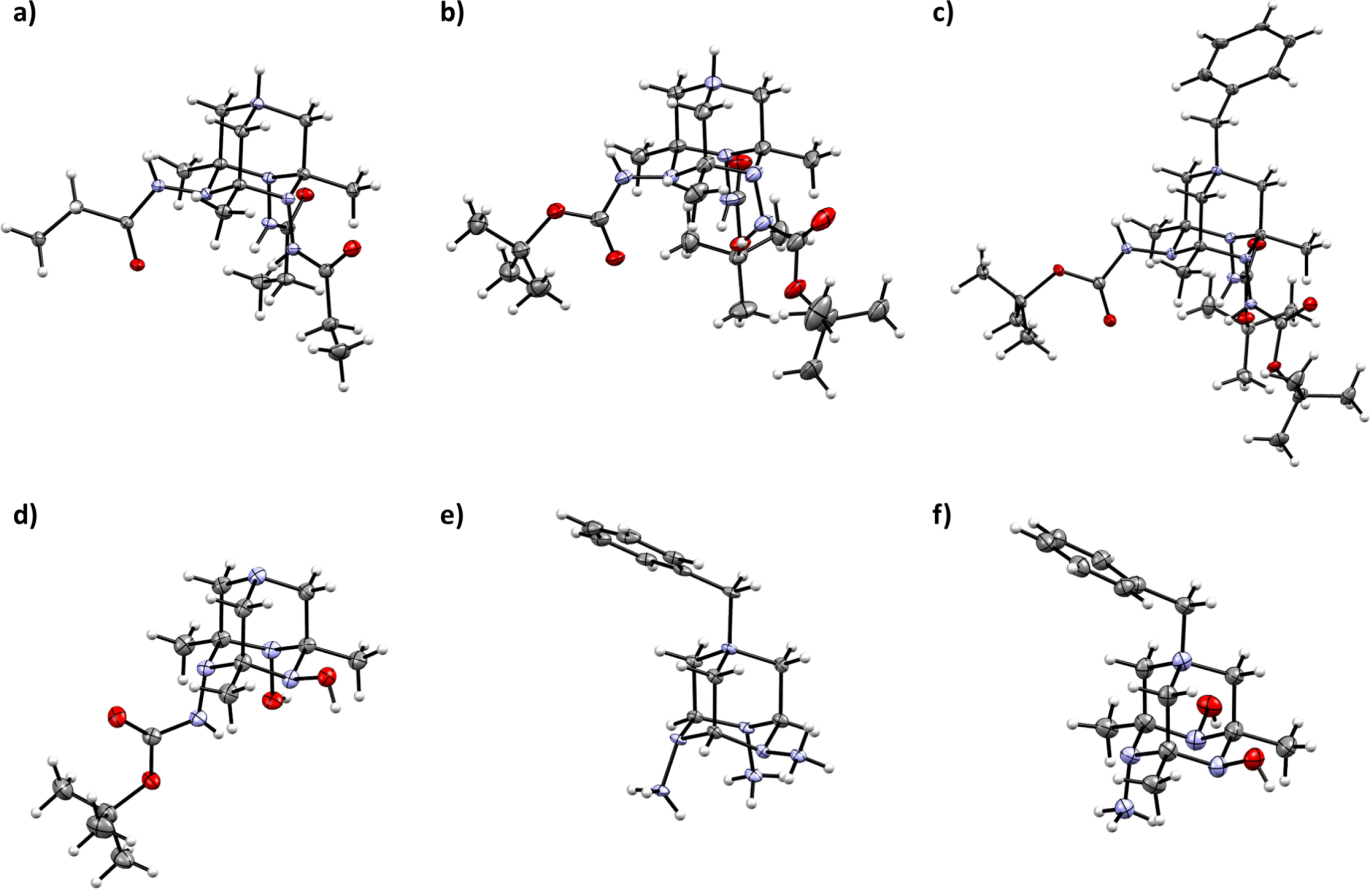
General view of the 1,4,6,10-tetraazaadamantane motif in X-ray structures of the obtained *N*-TAAD derivatives (representation of non-hydrogen atoms as thermal ellipsoids at 50% probability level). Anions and crystal-solvate molecules are omitted for clarity. (a) **4a**·HCl·H_2_O·MeOH. (b) **4c**·HCl·3.5MeOH. (c) Bn-**4c**·3CD_3_OD (bromide salt). (d) **8a**. (e) **19e**·3HCl·H_2_O. (f) **21**·HCl·2H_2_O.

Several of the obtained TAAD derivatives were characterized by X-ray analysis (namely, **4a**, **4a**·HCl, **4b** [35], **4c**·HCl, Bn-**4c**, **8a**, **19e**·3HCl, **21** and **21**·HCl, see Figure 2 and Supporting Information File 2). The geometrical parameters of the 1,4,6,10-tetraazaadamantane cage in these products are close to that observed in ^3^*O*-TAADs of type **2** [21]. Carbon–carbon bonds fall within a narrow range of 1.52–1.53 Å. Carbon–nitrogen bonds of the triazinane ring are within 1.45–1.50 Å, while the C–N distances involving bridge-head nitrogen N(1) expectedly differ in free amines (1.46–1.47 Å) and quaternary ammonium salts (1.48–1.52 Å).

Remarkably, in all ^3^*N*-TAADs (both with protected and free amino groups) two *N*-substituents are located in axial positions, while the third one is equatorial (Figure 2a–c, and e). Carbon–nitrogen bonds involving the nitrogen atom bearing an equatorial group appear to be somewhat elongated (by ca. 0.02–0.03 Å) compared to other C–N bonds of the triazinane ring. In the ^1^*N*,^2^*O-*TAADs the amino group is always axial, while OH groups occupy either *eq*,*eq* (in **21** and **21**·HCl) or *ax*,*eq* (in **8a**) positions (Figure 2d and f). A similar isomerism is observed in the previously described ^3^*O*-TAAD derivatives **2** [21]. In all TAADs, the nitrogen with an equatorial substituent is more deviated (0.55–0.61 Å) from the mean plane created by three carbon atoms of the triazinane ring than the nitrogen atom bearing and axial group (0.37–0.45 Å).

**Figure 3:**
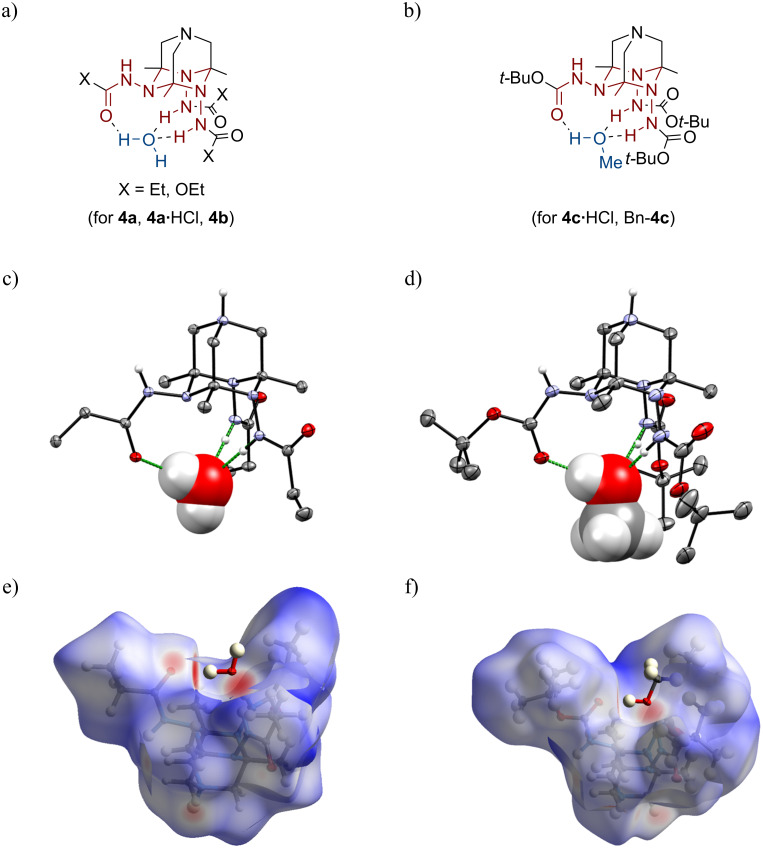
(a) General structure of host–guest complexes of ^3^*N*-TAADs with water. (b) The general structure of host–guest complexes of ^3^*N*-TAADs with methanol. (c) Host–guest motif in the X-ray structure of **4a**·HCl·H_2_O·MeOH (guest molecule is shown in “spacefill” representation). (d) Host–guest motif in the X-ray structure of **4c**·HCl·3.5MeOH (guest molecule is shown in “spacefill” representation). (e) Hirshfeld surface of the TAAD cation in **4a**·HCl·H_2_O·MeOH. (e) Hirshfeld surface of the TAAD cation in **4c**·HCl·3.5MeOH.

^3^*N*-TAADs **4** bearing amide and carbamate groups form inclusion complexes with water or methanol (Figure 3a–d). In the crystal state, a common hydrogen-bonded motif is observed, in which the guest molecule is located in a pocket created by amide/carbamate groups and the triazinane ring of the ^3^*N*-TAAD structure that is well-illustrated by Hirshfeld surfaces [41] (Figure 3e and f). The guest molecule (water or methanol) acts as an H-bond acceptor with two axial NH units (distances N···O 2.85–2.93 Å, 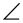 NHO 166.4–178.0°) and as an H-bond donor for the oxygen atom of the equatorial amide group (distances O···O 2.69–2.78 Å, 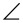 OHO 163.7–178.1°) forming two 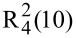-cyclic motifs.

There seems to be a preference for the coordination of certain guest molecules depending on the nature of the amide/carbamate groups. Thus, *N*-propionyl and ethyl carbamate derivatives (**4a**, **4a**·HCl, and **4b**) form the aforementioned complexes with water (Figure 3c), although lattice methanol molecules and chloride anions are present in the structure. The larger tris-Boc derivatives **4c**·HCl and Bn-**4c** form the same guest–host complexes with methanol (Figure 3d). Thus, these TAAD-based systems may exhibit molecular recognition properties.

Deprotected *N*-TAADs such as **19e**, **21**, and **21**·HCl as well as the previously described ^3^*O*-TAADs **2** do not form such host–guest complexes, although water and methanol molecules are present in their crystal lattices. Thus, the presence of amide/carbamate groups is essential for the assembly of these supramolecular structures.

**Figure 4:**
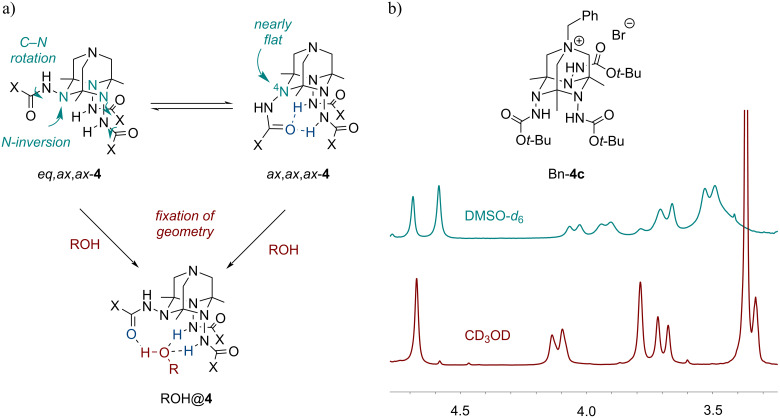
(a) Dynamic processes in TAADs **4** and complexation with ROH. (b) Fragment of ^1^H NMR spectra of Bn-**4c** in DMSO-*d*_6_ and CD_3_OD.

The formation of complexes can be evidenced in the solution state by NMR (Figure 4). Thus, ^1^H NMR spectra of protected tetraazaadamantanes **4** and Bn-**4** in DMSO-*d*_6_ are rather complicated and contain several sets of broad multiplets. This can be attributed to the presence of several invertomers (inversion of the cage nitrogen atoms) and/or rotamers (restricted rotation around amide C–N bonds), which is well-known for amides and carbamates (Figure 4a). The activation barriers for the rotation across amide C–N bonds in **4a** and **4c** estimated by DFT (18.4 and 19.4 kcal/mol, respectively, ωB97XD/Def2TZVP, gas phase) are close to those observed experimentally for amides and carbamates [42–43]. Upon changing the solvent to CD_3_OD or D_2_O the spectra become much simpler and a picture expected for a *C*_s_ symmetrical structure is observed (for example, cf. ^1^H NMR spectra of Bn-**4c** in different solvents shown in Figure 4b). Such symmetrization can be explained by a pre-organization of *N*-amido groups through the coordination of water/alcohol leading to the host–guest complex observed in the X-ray.

The formation of the inclusion complexes H_2_O@TAADs **4a** and MeOH@**4c**·H^+^ was additionally explored by DFT calculations at the ωB97XD/Def2TZVP level of theory with basis set superposition error (BSSE) correction (Table 1). Interestingly, in the most energetically favored invertomer *ax*,*ax*,*ax*-**4** (Figure 4a) the geometry around the nitrogen atom N(4) is close to planar (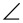 CN(4)N 121.1–121.4°, 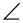 CN(4)C 112.5°). Such flattening is due to the formation of intramolecular bifurcated hydrogen-bonds between the corresponding carbonyl oxygen and NH hydrogens of two axial *N*-amido groups. The invertomer *eq*,*ax*,*ax*-**4a** (Figure 4a) lacking this H-bond is less stable by ca. 2 kcal/mol (Δ*G*°). Upon the formation of inclusion complexes with water/methanol, this H-bond is broken and two new H-bonds are formed resulting in a less strained structure with a normal pyramidal geometry of the N-4 nitrogen atom. The calculated structural metrics of complexes modeled by DFT are close to those observed in the X-ray structures.

**Table 1:** DFT calculated binding energies for the guest–host complexes ROH@**4**.

Complex	Calculated binding energy, kcal/mol^a,b^		

	Δ*E*_0_	Δ*H*°	Δ*G*°

H_2_O@**4a**	−14.1	−12.6	−2.8
H_2_O@**4c**·H^+^	−10.8	−9.1	+1.8
MeOH@**4c**·H^+^	−12.3	−10.9	−0.8
*t*-BuOH@**4c**·H^+^	−18.0	−16.4	−3.7

^a^ωB97XD/Def2TZVP with BSSE (gas phase). ^b^Relative energies were calculated as Δ*E* = Δ*E*_(ROH@_*_ax,ax,ax_*_-_**_4_**_)_ − *E*_(_*_ax,ax,ax_*_-_**_4_**_)_ − *E*_(ROH)_.

The formation of inclusion complexes is thermodynamically favored in the gas phase by 1–4 kcal/mol (Δ*G*°) showing rather weak bond character in these structures. Indeed, the guest molecules are liberated upon drying of the obtained crystal solvates in a vacuum with gentle heating.

Interestingly, a higher preference in coordination of *tert*-butanol over water and methanol was observed in competition experiments with TAAD **4c** (see Supporting Information File 1 for details). DFT calculations demonstrate that binding energy increases in the series H_2_O < MeOH < *t*-BuOH (cf. data in Table 1). This can be explained by additional stabilization of the complex through weak hydrophobic interactions of with *tert*-butyl groups of Boc. We believe that by adjustment of the substituents on the amide/carbamate groups, receptors for supramolecular sensing of alcohols [44] could be designed based on ^3^*N*-TAADs of type **4**. Further research in this direction is currently underway.

## Conclusion

In conclusion, we developed a synthetic route to 1,4,6,10-tetraazaadamantanes bearing free and protected amino groups at the bridge *N*-atoms (*N*-TAADs). It involves the assembly of a tris(iminoalkyl)amine precursor, which undergoes smooth intramolecular cyclotrimerization of the C=N bonds to afford the 1,4,6,10-tetraazaadamantane cage. This route was successfully exploited to prepare unsymmetrically substituted TAADs having both amino(amido) and hydroxy groups at bridge nitrogen atoms via a hitherto unknown co-trimerization of oxime and hydrazone groups. The *N*-TAADs with acylated amino groups are highly stable. In contrast, unprotected *N*-TAADs are prone to cage opening and hydrolysis, yet quaternization of the bridge-head nitrogen greatly stabilizes their structure. We have showcased that *N*-TAADs can serve as platforms for the design of chelating ligands and supramolecular receptors. In particular, we have shown that the ^1^*N*,^2^*O*-TAAD derivative reacts with phenylboronic acid producing a novel type of boron ate-complex with a rigid diamantane geometry. ^3^*N*-TAADs bearing *N*-acyl and alkoxycarbonyl groups form guest–host complexes with water or methanol, in which the guest molecule is fixed by a system of H-bonds in a pocket created by amide units and the triazinane ring. This creates opportunities for a rational design of supramolecular receptors for alcohols based on ^3^*N*-TAAD as a template.

## Supporting Information

File 1 Experimental procedures, NMR spectra, infrared spectra, X-ray data, and computation data.

File 2 Crystallographic information files (CIFs) for compounds **19e**·3HCl·H_2_O, Bn-**4c**(bromide)·3CD_3_OD, **21**·2D_2_O, **4a**·H_2_O·0.5MeOH, **4a**·HCl·H_2_O·MeOH, **4c**·HCl·3.5MeOH, **8a**, **21**·HCl·2H_2_O, and hydrazinium dihydrochloride. CCDC deposition numbers 2190361, 2190362, 2190363, 2182991, 2182992, 2182993, 2182994, 2182995, 2183048.

## References

[1] Liang L, Astruc D (2011). Coord Chem Rev.

[2] Hu X, Meyer K (2005). J Organomet Chem.

[3] Berg R, Straub B F (2013). Beilstein J Org Chem.

[4] Kuswandi B, Nuriman, Verboom W, Reinhoudt D N (2006). Sensors.

[5] Wadas T J, Wong E H, Weisman G R, Anderson C J (2010). Chem Rev.

[6] Valášek M, Lindner M, Mayor M (2016). Beilstein J Nanotechnol.

[7] Savyasachi A J, Kotova O, Shanmugaraju S, Bradberry S J, Ó’Máille G M, Gunnlaugsson T (2017). Chem.

[8] Zhang G, Mastalerz M (2014). Chem Soc Rev.

[9] Beuerle F, Gole B (2018). Angew Chem, Int Ed.

[10] Fleck C, Franzmann E, Claes D, Rickert A, Maison W (2013). Synthesis.

[11] Grillaud M, Bianco A (2015). J Pept Sci.

[12] Lamanna G, Grillaud M, Macri C, Chaloin O, Muller S, Bianco A (2014). Biomaterials.

[13] Fleck C, Memmel E, Fölsing M, Poll B, Hackl T, Seibel J, Maison W (2015). Eur J Org Chem.

[14] Ivleva E A, Pogulyaiko A V, Klimochkin Y N (2018). Russ J Org Chem.

[15] Balija A M, Kohman R E, Zimmerman S C (2008). Angew Chem, Int Ed.

[16] Kohman R E, Zimmerman S C (2009). Chem Commun.

[17] Hou T, Zhang J, Wang C, Luo J (2017). Org Chem Front.

[18] Semakin A N, Nelyubina Y V, Ioffe S L, Sukhorukov A Y (2020). Eur J Org Chem.

[19] Suslov E V, Ponomarev K Y, Volcho K P, Salakhutdinov N F (2021). Russ J Bioorg Chem.

[20] Dalinger A I, Medved’ko A V, Kalinin M A, Sereda V A, Churakov A V, Vatsadze S Z (2021). Russ Chem Bull.

[21] Semakin A N, Sukhorukov A Y, Lesiv A V, Ioffe S L, Lyssenko K A, Nelyubina Y V, Tartakovsky V A (2009). Org Lett.

[22] Semakin A N, Sukhorukov A Y, Nelyubina Y V, Khomutova Y A, Ioffe S L, Tartakovsky V A (2014). J Org Chem.

[23] Semakin A N, Sukhorukov A Y (2017). Targets Heterocycl Syst.

[24] Golovanov I S, Sukhorukov A Y, Nelyubina Y V, Khomutova Y A, Ioffe S L, Tartakovsky V A (2015). J Org Chem.

[25] Golovanov I S, Mazeina G S, Nelyubina Y V, Novikov R A, Mazur A S, Britvin S N, Tartakovsky V A, Ioffe S L, Sukhorukov A Y (2018). J Org Chem.

[26] de Vries R H, Viel J H, Kuipers O P, Roelfes G (2021). Angew Chem, Int Ed.

[27] Samoľová E, Premužić D, Plociennik S, Hołyńska M (2019). J Mol Struct.

[28] Premužić D, Hołyńska M, Ozarowski A, Pietzonka C, Roseborough A, Stoian S A (2020). Inorg Chem.

[29] Golovanov I S, Leonov A V, Lesnikov V K, Pospelov E V, Frolov K V, Korlyukov A A, Nelyubina Y V, Novikov V V, Sukhorukov A Y (2022). Dalton Trans.

[30] Ozerova V V, Zhidkov I S, Boldyreva A, Dremova N N, Emelianov N A, Shilov G V, Frolova L A, Kurmaev E Z, Sukhorukov A Y, Aldoshin S M (2021). Energies (Basel, Switz).

[31] Bondy C R, Loeb S J (2003). Coord Chem Rev.

[32] Martínez-Crespo L, Halgreen L, Soares M, Marques I, Félix V, Valkenier H (2021). Org Biomol Chem.

[33] Tomyn S, Shylin S I, Bykov D, Ksenofontov V, Gumienna-Kontecka E, Bon V, Fritsky I O (2017). Nat Commun.

[34] Shylin S I, Pogrebetsky J L, Husak A O, Bykov D, Mokhir A, Hampel F, Shova S, Ozarowski A, Gumienna-Kontecka E, Fritsky I O (2021). Chem Commun.

[35] Semakin A N, Kokuev A O, Nelyubina Y V, Sukhorukov A Y, Zhmurov P A, Ioffe S L, Tartakovsky V A (2016). Beilstein J Org Chem.

[36] Semakin A N, Sukhorukov A Yu, Lesiv A V, Khomutova Y A, Ioffe S L, Lyssenko K A (2007). Synthesis.

[37] Gillis E P, Burke M D (2007). J Am Chem Soc.

[38] Churches Q I, Hutton C A (2016). Introduction, Interconversion and Removal of Boron Protecting Groups. Boron Reagents in Synthesis.

[39] Golovanov I S, Sukhorukov A Y (2021). Top Curr Chem.

[40] Semakin A N, Golovanov I S, Sukhorukov A Y, Ioffe S L, Tartakovsky V A (2016). Russ Chem Bull.

[41] Spackman P R, Turner M J, McKinnon J J, Wolff S K, Grimwood D J, Jayatilaka D, Spackman M A (2021). J Appl Crystallogr.

[42] Kang Y K, Park H S (2004). J Mol Struct: THEOCHEM.

[43] Cox C, Lectka T (1998). J Org Chem.

[44] Pinalli R, Nachtigall F F, Ugozzoli F, Dalcanale E (1999). Angew Chem, Int Ed.

