# Supplementary material for: 1,4,6,10-Tetraazaadamantanes (TAADs) with N-amino groups: synthesis and formation of boron chelates and host–guest complexes
Source: Beilstein J Org Chem. 2022 Oct 11;18:1424–34. doi: 10.3762/bjoc.18.148 (PMC9577388; doi:10.3762/bjoc.18.148)
Supplement: File 1 — Experimental procedures, NMR spectra, infrared spectra, X-ray data, and computation data. [file Beilstein_J_Org_Chem-18-1424-s001.pdf]

BEILSTEIN JOURNAL OF ORGANIC CHEMISTRY

Supporting Information

for

1,4,6,10-Tetraazaadamantanes (TAADs) with *N*-amino groups: synthesis and formation of boron chelates and host–guest complexes

Artem N. Semakin, Ivan S. Golovanov, Yulia V. Nelyubina and Alexey Yu. Sukhorukov

Beilstein J. Org. Chem. **2022,** *18,* 1424–1434. doi:10.3762/bjoc.18.148

Experimental procedures, NMR spectra, infrared spectra, X-ray data, and computation data

License and Terms: This is a supporting information file under the terms of the Creative Commons Attribution License (https://creativecommons.org/licenses/by/4.0). Please note that the reuse, redistribution and reproduction in particular requires that the author(s) and source are credited and that individual graphics may be subject to special legal provisions.
The license is subject to the *Beilstein Journal of Organic Chemistry* terms and conditions: (https://www.beilstein-journals.org/bjoc/terms)

Table of contents

1. Experimental part	S1
2. Characterization of compounds, copies of NMR and FTIR spectra	S6
3. X-ray data	S87
4. DFT calculations	S106

1. Experimental part

Reactions were monitored by analytical TLC using silica gel TLC plates with QF-254 indicator. Visualization was accomplished with UV light and staining with a solution of ninhydrin in methanol. NMR spectra were acquired on Bruker AM300 spectrometers at 297 K (if not specified) with residual solvents peaks as an internal standard. Chemical shifts in ^{11}B spectra are given relative to $BF_3{\cdot}Et_2O$. Peaks in FTIR spectra data are reported in cm^{-1} with the following relative intensities: s (strong), m (medium), w (weak), br (broad). Coupling constants (*J*) are given in Hz. HRMS spectra were acquired on a Bruker MicrOTOF instrument. Elemental analyses (CHN) were performed at the Analytical center of the N.D. Zelinsky Institute of Organic Chemistry. Analyses for chlorine and bromine was performed by titration with 0.01 M $Hg(NO_3)_2$ and diphenylcarbazone visual indicator in ethanolic solution. Melting points (uncorrected) were determined on a Kofler hot-stage microscope. Commercial reagents and solvents were used without additional purification. Concentrated aqueous hydrochloric acid refers to ca. 37 wt %. solution. Compounds **3b**,**c**,[1] **9b**,**c**,[1] **10**,[1] **12**[2] and **14**[3] were prepared accordingly to a literature procedures.

Synthesis of trishydrazone 3a

A solution of propionylhydrazide (2 g, 22.7 mmol) in MeOH (20 mL) was cooled on an ice-bath and chloroacetone (2 ml, 2.3 g, 24.8 mmol) was added dropwise. The reaction mixture was kept at the same temperature for 10 min and aqueous ammonia (25–28 wt %, 20 ml) was added. A pale yellow precipitate was formed immediately after ammonia addition. The reaction mixture was diluted with cold water (50 ml) and the precipitate was filtered, washed thoroughly with water and methanol, and dried on a filter to afford trishydrazone **3a** (2.35 g, 78%) as off-white powder.

Synthesis of benzyl 2-(1-chloropropan-2-ylidene)hydrazine-1-carboxylate (9d)

To a solution of benzylcarbazate (1.66 g, 10 mmol) in MeOH (20 ml) was added acetic acid (0.86 ml, 15 mmol). The solution was cooled on an ice-bath and chloroacetone (1.2 ml, 15 mmol) was added dropwise. The reaction mixture was kept at the same temperature for 2 h and poured in cold water (150 ml). The formed oil was crystallized in 5–10 min of stirring, and the white precipitate was filtered, washed thoroughly with water and dried on a filter to afford chlorohydrazone **9d** as a white solid (2.2 g, 91%).

Synthesis of *tert*-butyl 2-(2-bromoethylidene)hydrazine-1-carboxylate (9e)

A mixture of bromoacetaldehyde diethyl acetal (5 g, 25.4 mmol), water (30 ml), and sulfuric acid (3 drops) was refluxed until clear (1–2 h). The reaction mixture was cooled on an ice bath and a solution of *tert*-butyl carbazate (2 g, 15.1 mmol) in methanol (15 ml) was added. After 10 min, 50 ml of water were added, the formed precipitate was filtered, washed thoroughly with water, and dried on a filter to afford bromohydrazone **9e** as a white solid (2.35 g, 78%).

Synthesis of trishydrazones 3d and 3e

To a stirred solution of **9d** or **9e** (5 mmol) in MeOH (25 ml) aqueous ammonia (25–28%, 5 ml) was added. After 5 min, water (50 ml) was added and the precipitate was filtered, thoroughly

[1] A. N. Semakin, A. O. Kokuev, Y. V. Nelyubina, A. Y. Sukhorukov, P. A. Zhmurov, S. L. Ioffe, V. A. Tartakovsky, *Beilstein J. Org. Chem.* **2016**, 2471

[2] A. D. Dilman, A. A. Tishkov, I. M. Lyapkalo, S. L. Ioffe, Yu. A. Strelenko, V. A. Tartakovsky, *Synthesis*, **1998**, 181

[3] A. N. Semakin, A. Yu. Sukhorukov, S. L. Ioffe, V. A. Tartakovsky *Synthesis*, **2011**, 1403.

washed with water (**3d**) or water and MeOH (**3e**), and dried on a filter. Yields: 69% for **3d** and 78% for **3e**. NMR spectra of **3e** are in agreement with literature data.[1]

Synthesis of bishydrazone 11

To a suspension of *N*-benzyl bishydrazone **10** (1.35 g, 3.02 mmol) in MeOH (20 ml) was added 10% Pd/C (200 mg) under an argon atmosphere. The reaction vessel was evacuated and filled with hydrogen for 3 times (rubber balloon). The hydrogenation was carried under 1 bar of hydrogen during 2–3 h (NMR monitoring). The reaction mixture was centrifuged and the solution was concentrated in vacuo. The residue was triturated with MTBE, filtered, and dried in vacuo until constant weight to give bishydrazone **11** (875 mg, yield 81%) as a white solid.

Synthesis of bishydrazone-monooxime 5a

To a solution of bishydrazone **11** (690 mg, 1.93 mmol) in MeOH (6 ml) was added a solution of *N*-(prop-1-en-2-yl)-*O*-(trimethylsilyl)-*N*-((trimethylsilyl)oxy)hydroxylamine (**12**, 2.9 ml, 1M in CH_2Cl_2, 2.9 mmol) upon stirring. The reaction mixture was kept at rt for 48 h and volatile components were removed in vacuo. The residue was triturated with diethyl ether with several drops of MeOH, filtered, and the product was dried in vacuo until constant weight to give product **5a** (670 mg, yield 81%) as a white solid.

Synthesis of hydrazone-bisoxime 7a

To a solution of bisoxime **14** (950 mg, 5.97 mmol) in MeOH (10 ml) K_2CO_3 (1.24 g, 8.96 mmol) was added. Then, halohydrazone **9c** (1.54 g, 7.46 mmol) was added in one portion with vigorous stirring. The reaction mixture was stirred for 1 h and concentrated in vacuo. The residue was triturated with water, filtered, and washed with water and ether, and the product was dried off in vacuo until constant weight to give product **7a** (1.38 g, yield 71%) as a white solid.

Synthesis of TAADs 4a–e, 6a, and 8a

A solution or suspension of trisimine **3**, **5** or **7** (1 mmol) in MeOH (**3a**, **3c-e**, **5a**, **7a**) or water (**3b**) (5 ml) was refluxed (ca. 2 h) with monitoring by TLC or until complete dissolution (for **3a** and **3b**). The reaction mixture was concentrated in vacuo to dryness. The residue was triturated with ether, filtered, washed with diethyl ether, and dried in vacuo until constant weight. Yields: 78% for **4a**, 92% for **4b**, 79% for **4c**, 90% for **4d**, 75% for **4e**, 78% for **6a**, 89% for **8a**.

Synthesis of TAAD 8b

To a solution of bisoxime **14** (470 mg, 2.96 mmol) in MeOH (5 ml) was added K_2CO_3 (614 mg, 4.45 mmol). Then, chlorohydrazone **9d** (890 mg, 3.70 mmol) was added in one portion with vigorous stirring. The reaction mixture was stirred for 1 h and concentrated in vacuo. To the residue was added water (25 ml) and EtOAc (50 ml) and the mixture was stirred until dissolution of all solids. The organic phase was separated, dried with Na_2SO_4, and concentrated in vacuo. The residue was redissolved in MeOH (25 ml) and refluxed for 1 h. Then, volatiles were removed under reduced pressure, and the residue was triturated with diethyl ether, filtered, and dried in vacuo until constant weight to give TAAD **8b** (705 mg, yield 65%) as a pale yellow amorphous solid.

Deprotection of TAAD 8b

To a solution of **8b** (0.5 mmol) in MeOH (5 ml) was added 10% Pd/C (50 mg) under an argon atmosphere. The reaction vessel was evacuated and filled with hydrogen for 3 times (rubber balloon). The hydrogenation was carried under 1 bar of hydrogen for 30–40 min (TLC

monitoring). The reaction mixture was centrifuged and the solution was concentrated in vacuo. The residue was dried in vacuo until constant weight to afford TAAD **15**, which exists in a dynamic equilibrium with ring-chain isomers **16** and **17** (yield 87 mg, 85%).

Synthesis of azaoxaboradiadamantane 18

Dynamic mixture of **15**, **16**, and **17** from the previous procedure (85 mg, 0.37 mmol) was dissolved in MeOH (1.5 ml) and phenylboronic acid (70 mg, 0.57 mmol) was added. The reaction mixture was kept in refrigerator for 4 days and concentrated in vacuo. The residue was washed with diethyl ether and dried in vacuo until constant weight to give boronate **18** (yield 115 mg, 99%) as a white solid.

Synthesis of TAAD hydrochlorides 4a·HCl and 4c·HCl

To a solution of TAADs **4a** or **4c** (1 mmol) in MeOH (5 ml) concentrated aqueous hydrochloric acid (1.0 ml of 1.00 M solution) was slowly added. The reaction mixture was concentrated in vacuo at 20 °C and the residue was dried in vacuo until constant weight to give corresponding hydrochlorides **4a**·HCl and **4c**·HCl in a quantitative yield (99%).

Synthesis of quaternary salts Bn-4c, Bn-4e, Bn-6a and Bn-8a

To a solution of TAADs **4c–e**, **6a** or **8a** (1 mmol) in MeOH (4 ml) was added benzyl chloride (150 μl, 1.3 mmol). The reaction mixture was kept at rt for 1 day and concentrated in vacuo to dryness. The residue was triturated with diethyl ether, filtered, washed with diethyl ether, and dried at 70–80 °C in vacuo until constant weight. Yields: 86% for **Bn-4c**, 82% for **Bn-4e**, 85% for **Bn-6a**, 96% for **Bn-8a**.

Bromide salt **Bn-4c**(bromide) for X-ray diffraction analysis was prepared by the same procedure using benzyl bromide instead of benzyl chloride in 92% yield.

Crystal solvates of **Bn-4c**(chloride) and **Bn-4c**(bromide) with two molecules of methanol were prepared by a gentle drying the corresponding crude products at 20 °C in vacuo or in a dessicator over $CaCl_2$.

Synthesis of TAADs 19–21

A solution/suspension of **Bn-4c**(chloride), **Bn-4e**, **Bn-6a**, or **Bn-8a** (0.3 mmol) in water (4 ml) was refluxed for 4 h, then concentrated and dried in vacuo (0.1–0.5 Torr, 70 °C) to give the corresponding products **19c**, **19e**, **20**, or **21** in a quantitative yield (99%).

Acidic deprotection of Bn-4c to 19c·3HCl·2/3H$_2$O

To a concentrated aqueous hydrochloric acid (2 ml) cooled to −20 °C was added chloride salt **Bn-4c** (160 mg, 0.25 mmol). The reaction mixture was kept at same temperature for 0.5 h and then allowed to slowly warm to −5 °C (approx. 1 h) until complete dissolution of the solid. The resulting solution was frozen in liquid nitrogen. Upon slow thawing of the solution, vacuum (0.1–0.3 Torr) was applied to remove volatiles, that gave salt **19c**·3HCl·2/3H_2O as a white solid (yield 117 mg, 99%). *Compound* ***19c****·3HCl·2/3H$_2$O is unstable and should be stored in a refrigerator.*

Acidic deprotection of Bn-4e to 19e·3HCl·1.5H$_2$O

To concentrated aqueous hydrochloric acid (2 ml) cooled to 0 °C (ice bath) was added **Bn-4e** (153 mg, 0.25 mmol). The reaction mixture was kept at the same temperature for 1 h and the crystalline precipitate was filtered, washed with cold hydrochloric acid (conc., 2 × 1 ml) and

dried in vacuo until constant weight to give salt **19e**·3HCl·1.5H_2O as a white solid (82 mg, yield 73%).

Acidic deprotection of Bn-6a to 20·2HCl·2H_2O

To concentrated aqueous hydrochloric acid (2 ml) cooled to 0 °C (ice bath) was added **Bn-6a** (140 mg, 0.25 mmol). The reaction mixture was kept at the same temperature for 1 h and evaporated in vacuo (0.1–0.3 Torr, 10–20 °C) to dryness to give salt **20**·2HCl·2H_2O as a pale yellow solid (yield 115 mg, 99%).

Acidic deprotection of Bn-8a to 21·HCl·2H_2O

To concentrated aqueous hydrochloric acid (4 ml) cooled to 0 °C (ice bath) was added **Bn-8a** (400 mg, 0.88 mmol). The reaction mixture was kept at rt for 3 h. The crystalline precipitate was filtered, washed with EtOH (abs.) and dried in vacuo until constant weight to give salt **21**·HCl·2H_2O as a white solid (265 mg, yield 70%).

Conversion of Bn-4c to ozatriazaadamantane 22

To concentrated aqueous hydrochloric acid (2 ml) cooled to 0 °C (ice bath) was added **Bn-4c** (164 mg, 0.25 mmol). The reaction mixture was kept in a refrigerator (0–5 °C) overnight. The formed crystals were quickly filtered (while solution cold) to afford hydrazine dihydrochloride (16 mg, 60%). The filtrate was concentrated in vacuo at 10–20 °C until dryness; the residue was dissolved in absolute EtOH (4 ml) and filtered. The filtrate was concentrated in vacuo at 20 °C and dried in vacuo until constant weight to give product **22** as a white solid (yield 72 mg, 70%). *Compound **22** is unstable and should be stored in a refrigerator.*

Conversion of hydrochloric salt 19c·3HCl·2/3H_2O into free trishydrazine 19c

To a solution of **19c**·3HCl·2/3H_2O (120 mg, 0.25 mmol) in EtOH (abs., 4 ml) was added $NaHCO_3$ (42 mg, 0.5 mmol). The reaction mixture was stirred for 0.5 h, centrifuged and the solution was concentrated in vacuo. The residue was dried in vacuo until constant weight to give trishydrazine **19c** as a white solid (yield 82 mg, 92%).

Synthesis of TAAD 23

A solution/suspension of **Bn-8a** (250 mg, 0.55 mmol) and Zn dust (6–9 μm, 1.5 g) in water (10 ml) was refluxed for 4.5 h (control by ^{1}H NMR). Then, the reaction mixture was centrifuged and the solid was washed with water (4 ml). The combined solutions were concentrated in vacuo, the residue was dried in vacuo until constant weight to give TAAD **23** as a white solid (yield 167 mg, 94%).

Competition experiments

TAAD **4c** was dissolved in an equimolar mixture of methanol/water (*experiment 1*), methanol/*tert*-butanol (*experiment 2*) or water/*tert*-butanol (*experiment 3*) at rt. The obtained solutions were subjected to isothermal evaporation at room temperature. The resulting solids were dried in vacuo and analyzed by ^{1}H NMR in $CDCl_3$. The sample obtained in experiment 1 was identical to the initial TAAD **4c**. Samples obtained in *experiments 2 and 3* showed the presence of *tert*-butanol (1–2 equiv) and a characteristic picture for inclusion complexes of TAAD suggesting the formation of *t*-BuOH@**4c**. No methanol signal was detected in the sample from *experiment 2*.

Characterization of compounds, copies of NMR and FTIR spectra

Benzyl 2-(1-chloropropan-2-ylidene)hydrazine-1-carboxylate (9d)

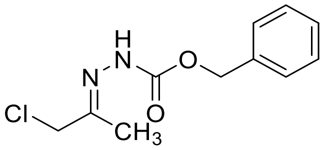

White solid, mp = 85-91 °C, mixture of *E*/*Z* isomers in ratio 15:1.

^{1}H NMR (300 MHz, $CDCl_3$): *E*-isomer, δ = 1.90 (s, 3 H, C*H*$_3$), 4.16 (s, 2 H, C*H*$_2$), 5.22 (s, 2 H, PhC*H*$_2$), 7.3-7.4 (m, 5 H, *Ph*), 7.8-8.4 (br, 1 H, N*H*); selected signals of *Z*-isomer, δ = 2.12 (s, 3 H, C*H*$_3$), 3.95 (s, 2 H, C*H*$_2$).

^{13}C NMR (75 MHz, $CDCl_3$): δ = 13.1 (*C*H$_3$), 48.6 (*C*H$_2$), 67.7 (Ph*C*H$_2$), 128.5, 128.6 (*o*,*m*,*p*-*Ph*) and 135.6 (*i*-*Ph*), 147.8 (*C*=N), 153.7 (*C*=O).

HRMS: Calcd for $C_{11}H_{13}ClN_2O_2Na^+$ [M+Na$^+$] m/z: 263.0558 and 265.0529. Found: 263.0559 and 265.0532.

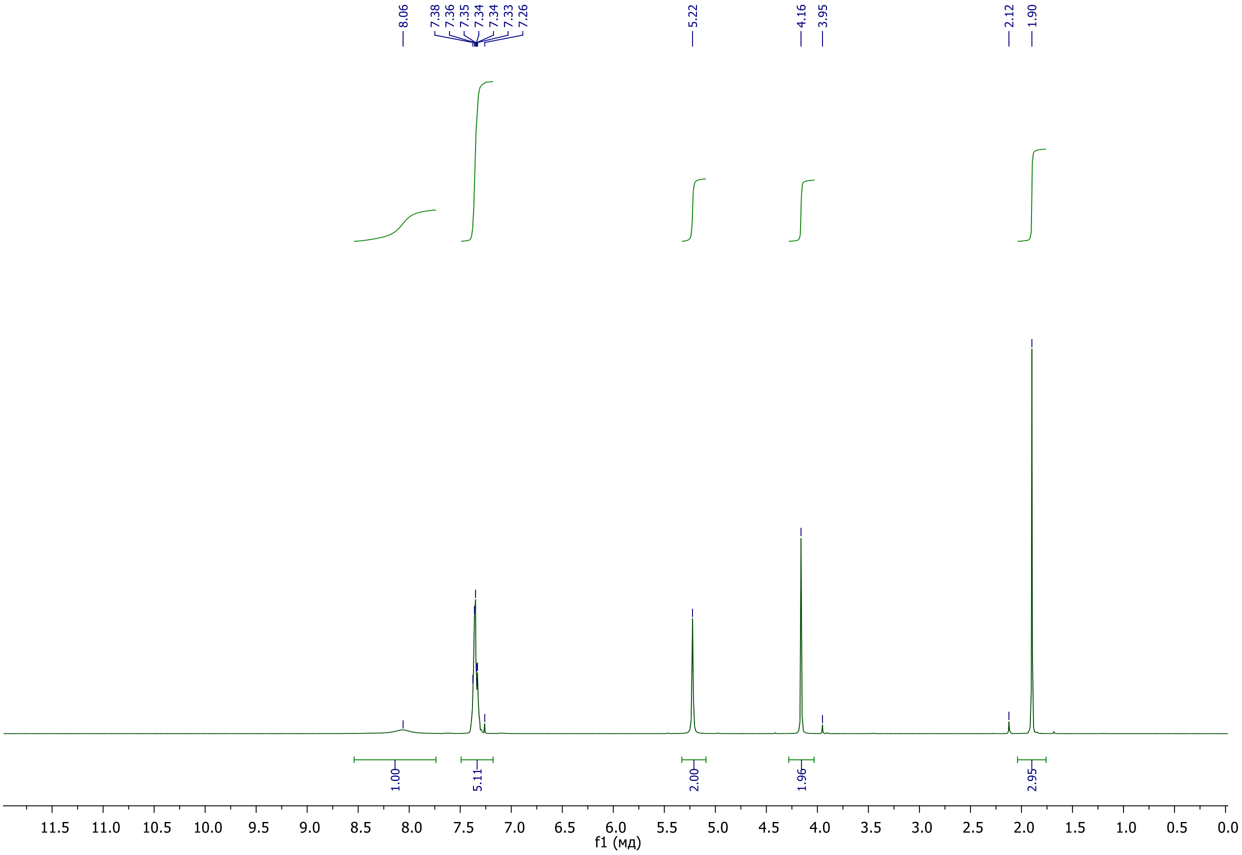

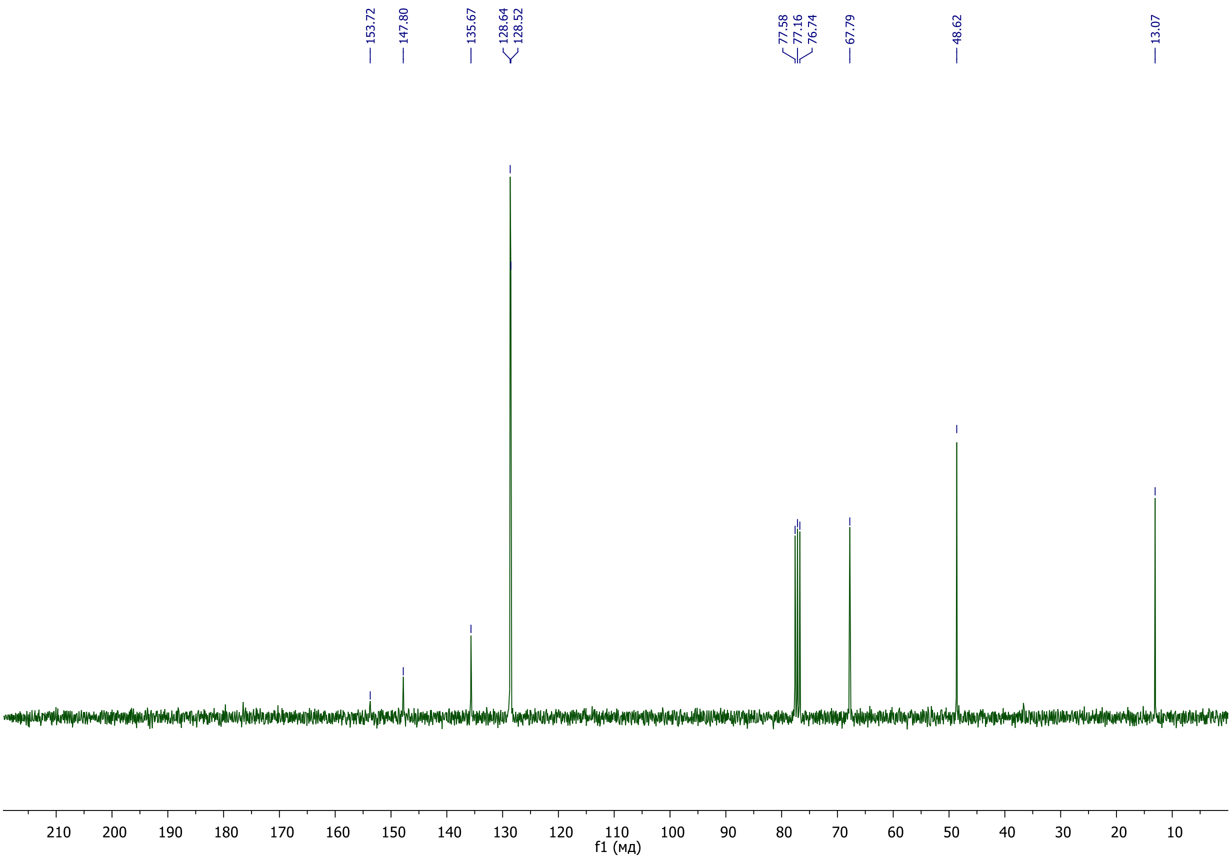
153.72
147.80
135.67
128.64
128.52
77.58
77.16
76.74
67.79
48.62
13.07
210 200 190 180 170 160 150 140 130 120 110 100 90 80 70 60 50 40 30 20 10
f1 (мд)

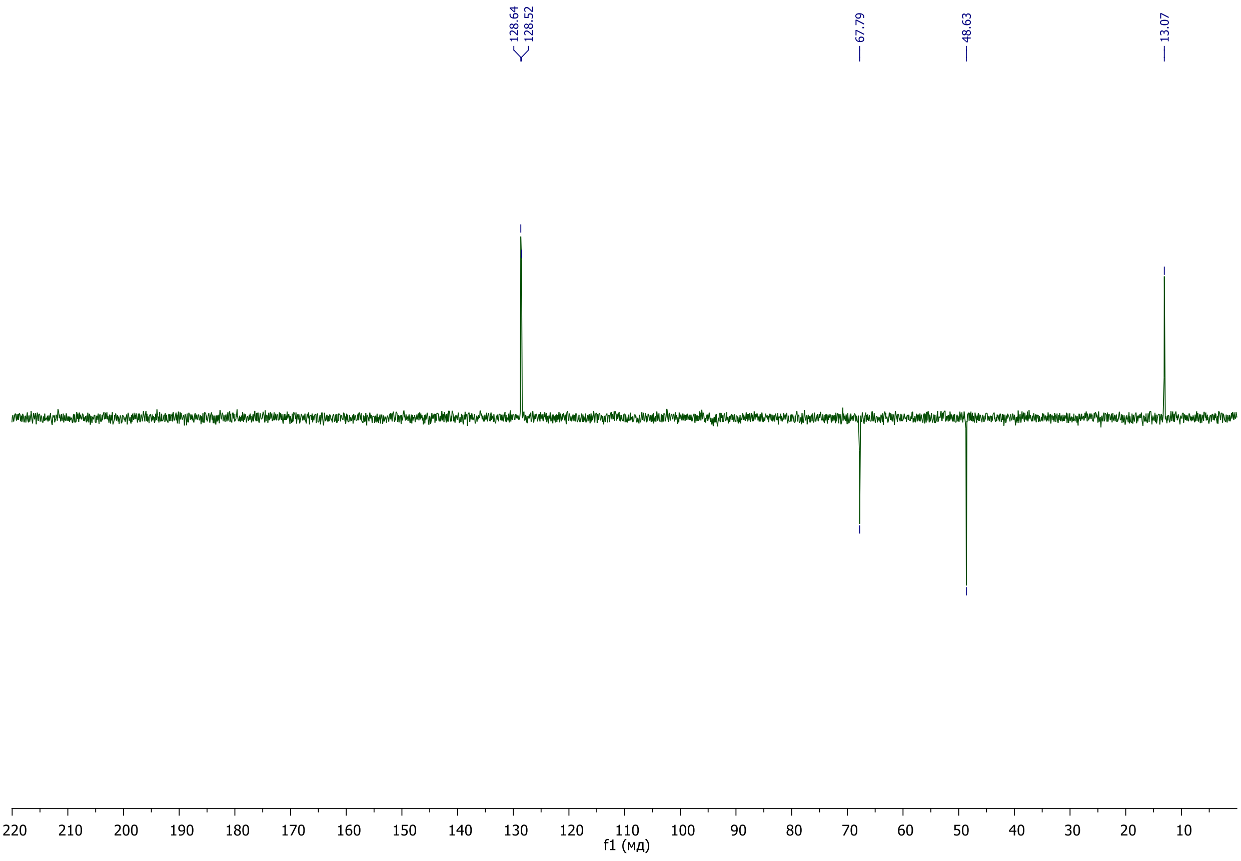
128.64
128.52
67.79
48.63
13.07
220 210 200 190 180 170 160 150 140 130 120 110 100 90 80 70 60 50 40 30 20 10
f1 (мд)

tert-Butyl 2-(2-bromoethylidene)hydrazine-1-carboxylate (9e)

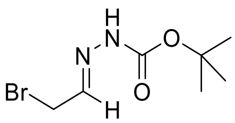

White solid, mp = 72-77 °C

^{1}H NMR (300 MHz, $CDCl_3$): δ = 1.48 (s, 9 H, C(C*H*$_3$)$_3$), 4.04 (d, *J* = 6.0 Hz, 2 H, C*H*$_2$), 7.32 (t, *J* = 6.0 Hz, 1 H), 8.61 (s, 1 H).

^{13}C NMR (75 MHz, $CDCl_3$): δ = 28.3 (C(*C*H$_3$)$_3$), 29.9 (*C*H$_2$), 81.8 (*C*(CH$_3$)$_3$), 140.5 (*C*=N), 152.6 (*C*=O).

HRMS: Calcd for $C_7H_{13}BrN_2O_2Na^+$ [M+Na$^+$] m/z: 259.0053 and 261.0032. Found: 259.0057 and 261.0070.

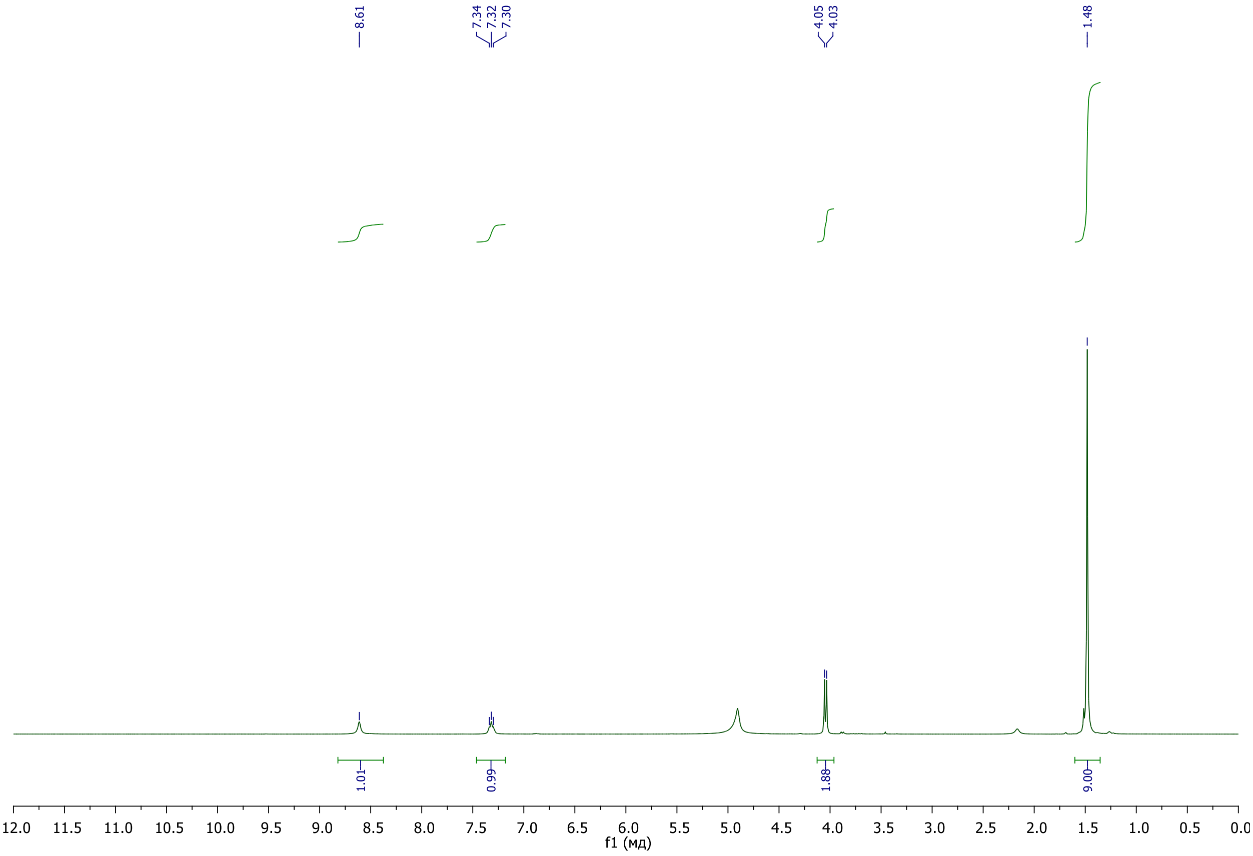

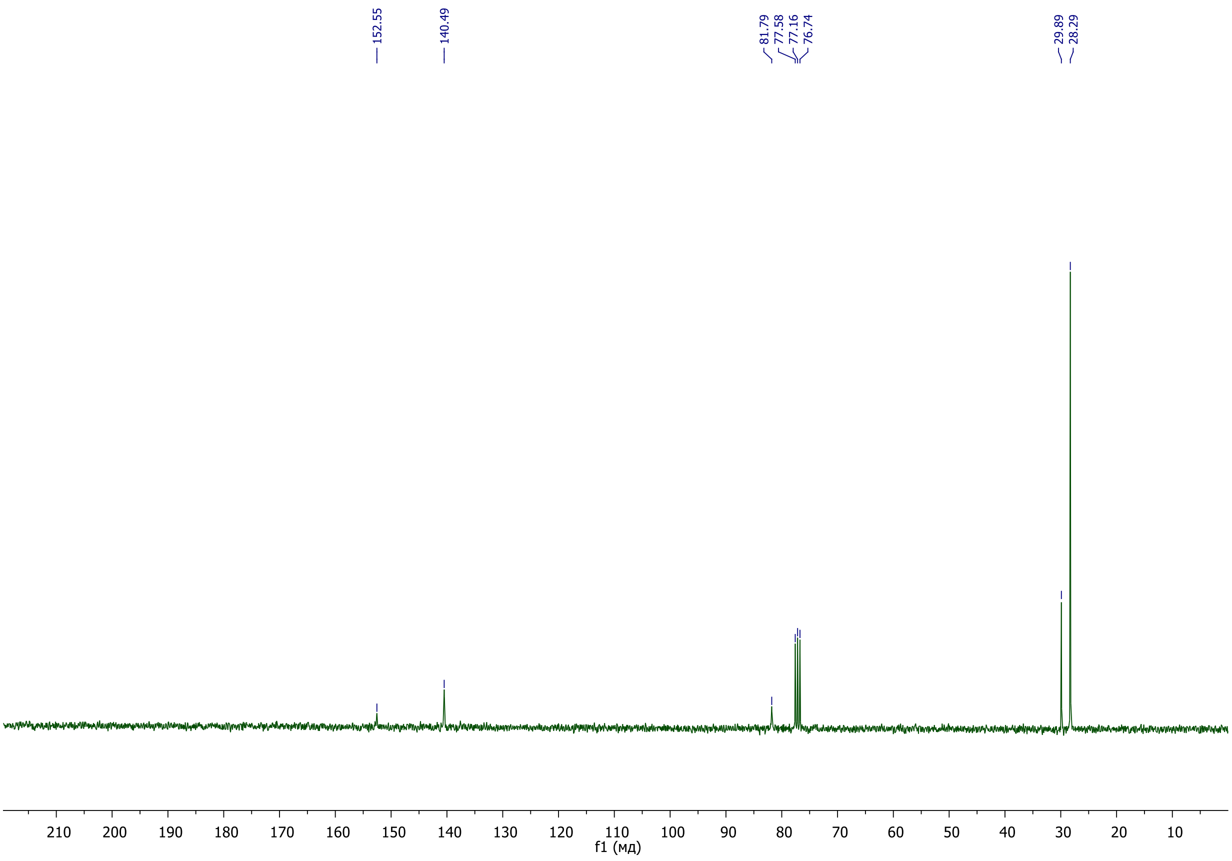

152.55
140.49
81.79
77.58
77.16
76.74
29.89
28.29
210 200 190 180 170 160 150 140 130 120 110 100 90 80 70 60 50 40 30 20 10
f1 (мд)

Bishydrazone 11

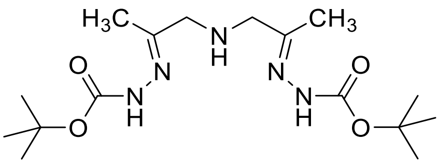

White solid, mp = 175-177 °C. Mixture of isomers and/or rotamers.

^{1}H NMR (300 MHz, CD_3OD): main isomer, δ = 1.53 (s, 18 H, 2 C(C*H*$_3$)$_3$), 1.89 (s, 6 H, C*H*$_3$), 3.33 (s, 4 H, 2 C*H*$_2$); signals of minor isomers/rotamers, δ = 1.50 (s, C(C*H*$_3$)$_3$), 1.90 and 1.97 (2 s, 2 C*H*$_3$), 3.47 (s, C*H*$_2$).

^{13}C NMR (75 MHz, CD_3OD): main isomer, δ = 14.8 (2 *C*H$_3$), 28.6 (2 C(*C*H$_3$)$_3$), 55.3 (2 *C*H$_2$), 81.6 (2 *C*(CH$_3$)$_3$), 153.3 and 156.0 (2 *C*=N and 2 *C*=O).

HRMS: Calcd for $C_{16}H_{31}N_5O_4Na^+$ [M+Na$^+$] m/z: 380.2268. Found: 380.2259.

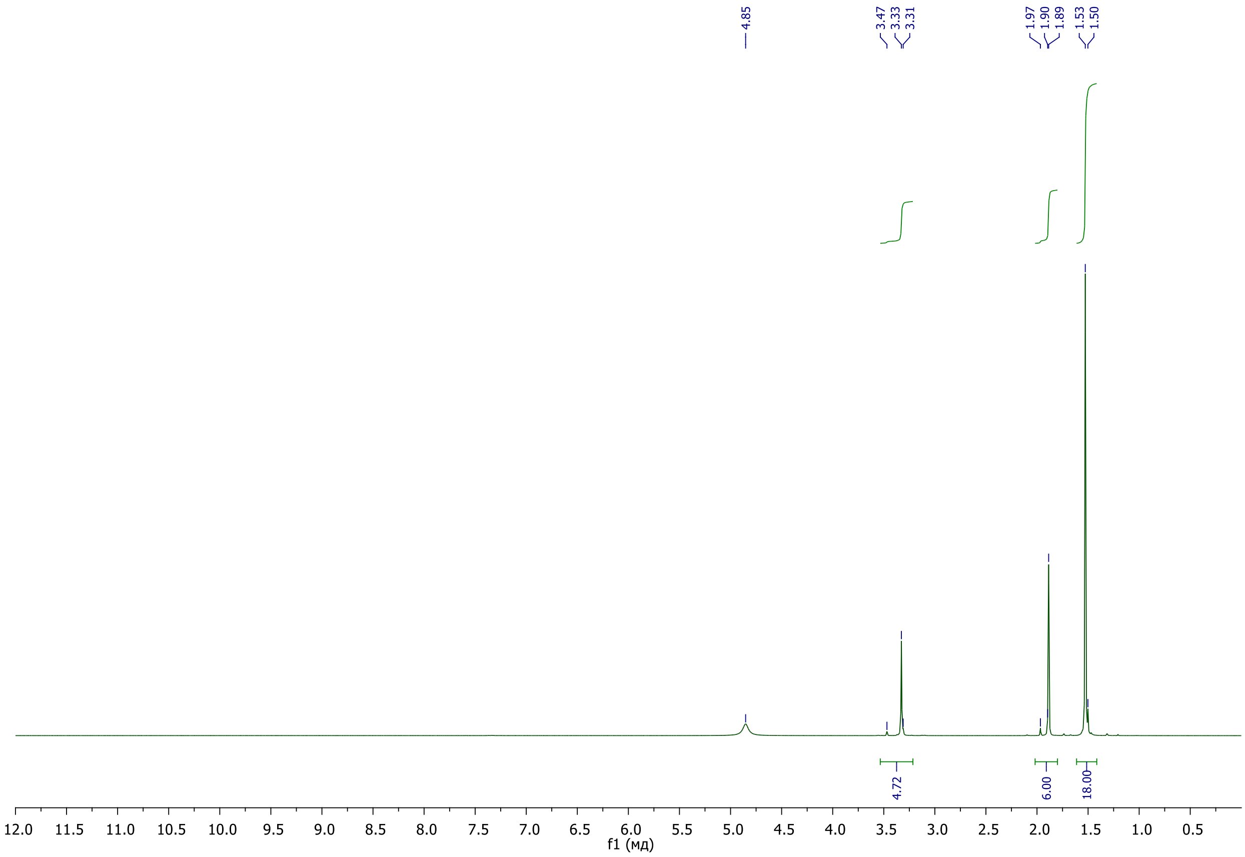

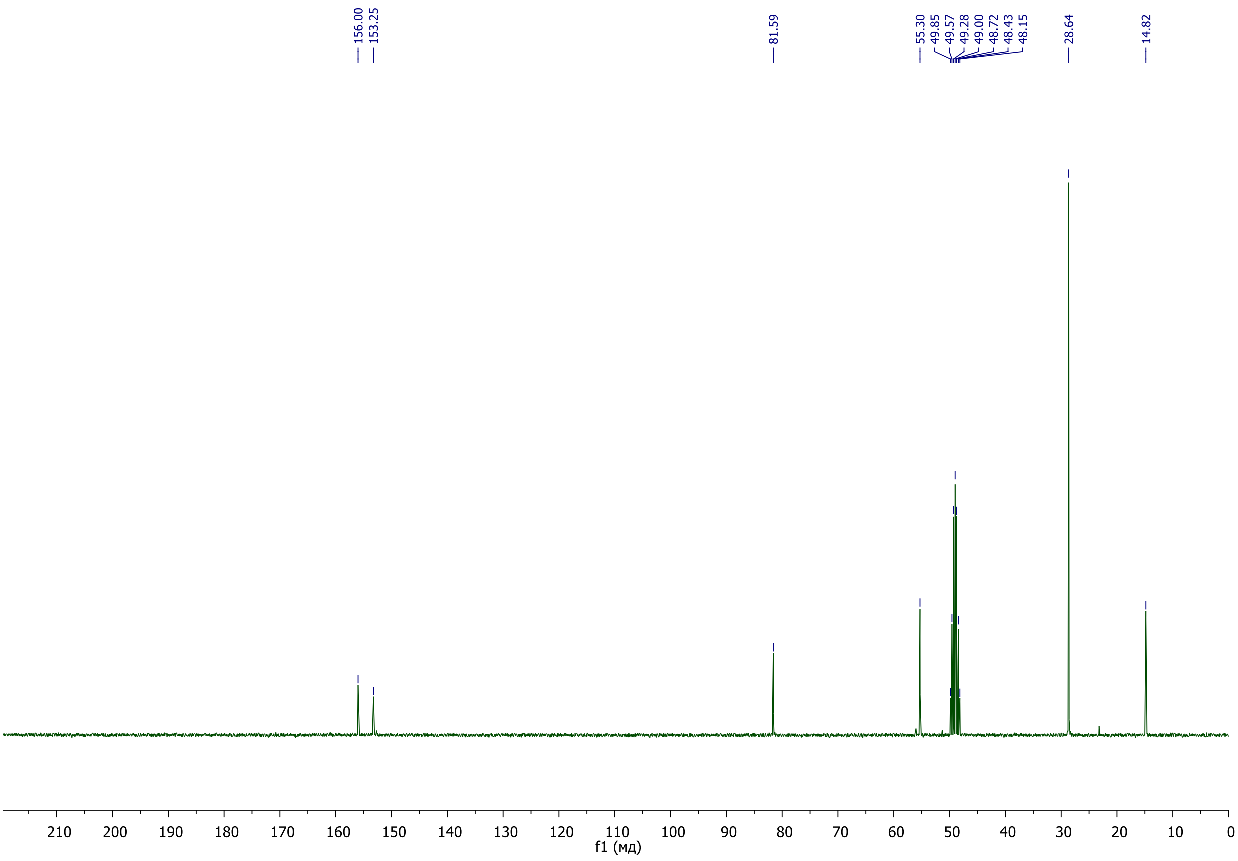
156.00
153.25
81.59
55.30
49.85
49.57
49.28
49.00
48.72
48.43
48.15
28.64
14.82
210 200 190 180 170 160 150 140 130 120 110 100 90 80 70 60 50 40 30 20 10 0
f1 (мд)

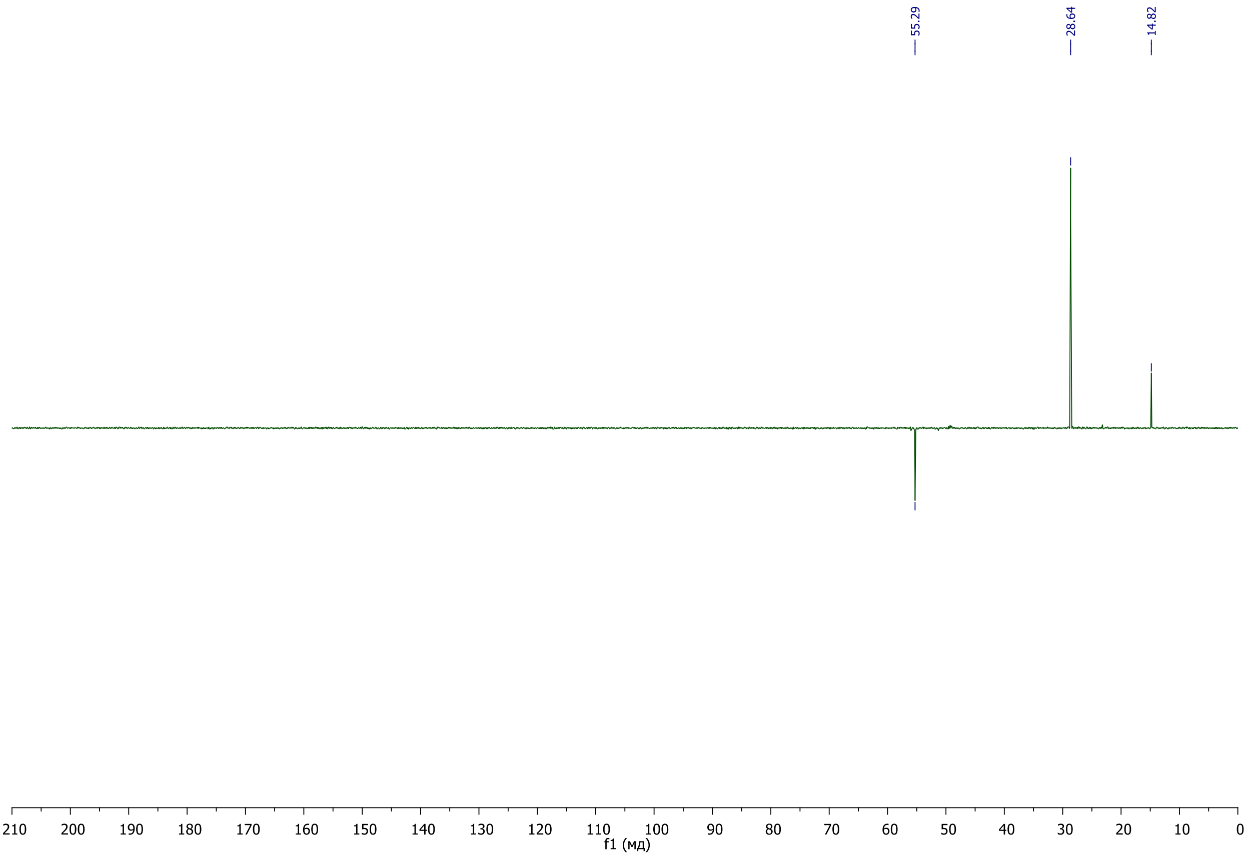
55.29
28.64
14.82
210 200 190 180 170 160 150 140 130 120 110 100 90 80 70 60 50 40 30 20 10 0
f1 (мд)

Trishydrazone 3a

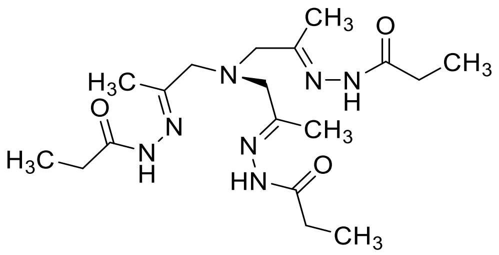

White solid, mp = 209-218 °C.

Due to extremely low solubility in all available solvents ($CDCl_3$, DMSO-d6, D_2O, CD_3OD) NMR spectra could not be recorded.

FTIR (KBr): 934 (w), 1015 (w), 1093 (m), 1219 (s), 1276 (w), 1360 (w), 1446 (w), 1548 (s, $\nu_{C=N}$), 1672 (s, $\nu_{C=O}$), 2829 (w, ν_{C-H}), 3040 (m, ν_{C-H}), 3204 (br, ν_{N-H}), 3200-3600 (br, ν_{N-H}).

HRMS: Calcd for $C_{18}H_{33}N_7O_3Na^+$ $[M+Na^+]$ m/z: 418.2537. Found: 418.2533.

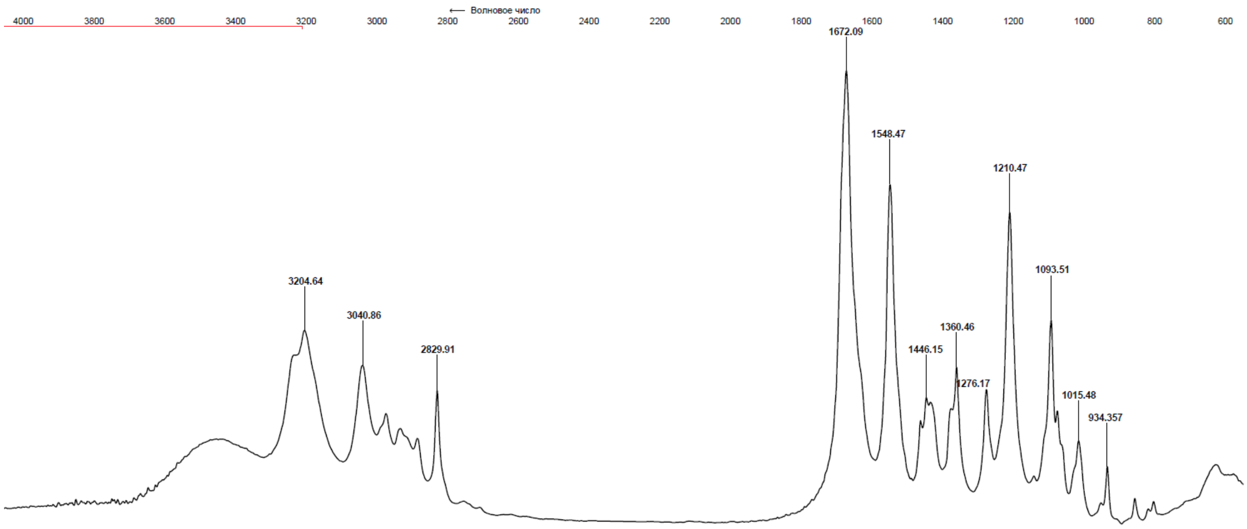

Trishydrazone 3d

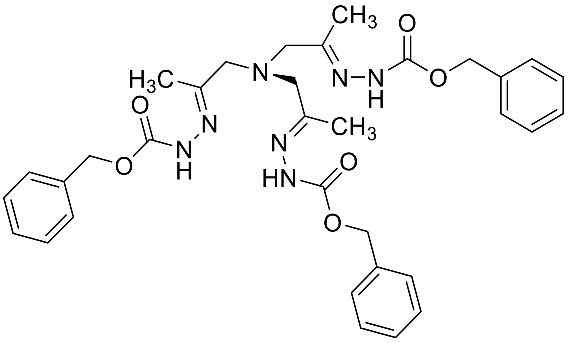

White amorphous solid, mp = 100-110 °C (starts softening at 70 °C). Mixture of isomers and/or rotamers.

^{1}H NMR (300 MHz, DMSO-d6): main isomer, δ = 1.83 (s, 9 H, 3 C*H$_3$*), 2.98 (s, 6 H, 2 C*H$_2$*), 5.13 (s, 6 H, 3 PhC*H$_2$*), 7.2-7.5 (m, 15 H, 3 *Ph*), 9.91 (s, 3 H, 3 N*H*); selected signals of minor isomers/rotamers, δ = 3.04, 3.17 and 3.21 (C*H$_3$*), 9.98 and 10.96 (N*H*).

^{13}C NMR (75 MHz, DMSO-d6): main isomer, δ = 14.6 (3 CH_3), 59.9 (3 *C*H$_2$), 65.7 (3 Ph*C*H$_2$), 127.5, 128.0 and 128.3 (3 *o,m,p-Ph*), 136.6 (3 *i-Ph*), 152.4 and 154.0 (3 *C*=N and 3 *C*=O); selected signals of minor isomers/rotamers, δ = 14.9 and 23.3 (*C*H$_3$), 53.4 (*C*H$_2$).

HRMS: Calcd for $C_{33}H_{39}N_7O_6Na^+$ [M+Na$^+$] m/z: 652.2854. Found: 652.2840.

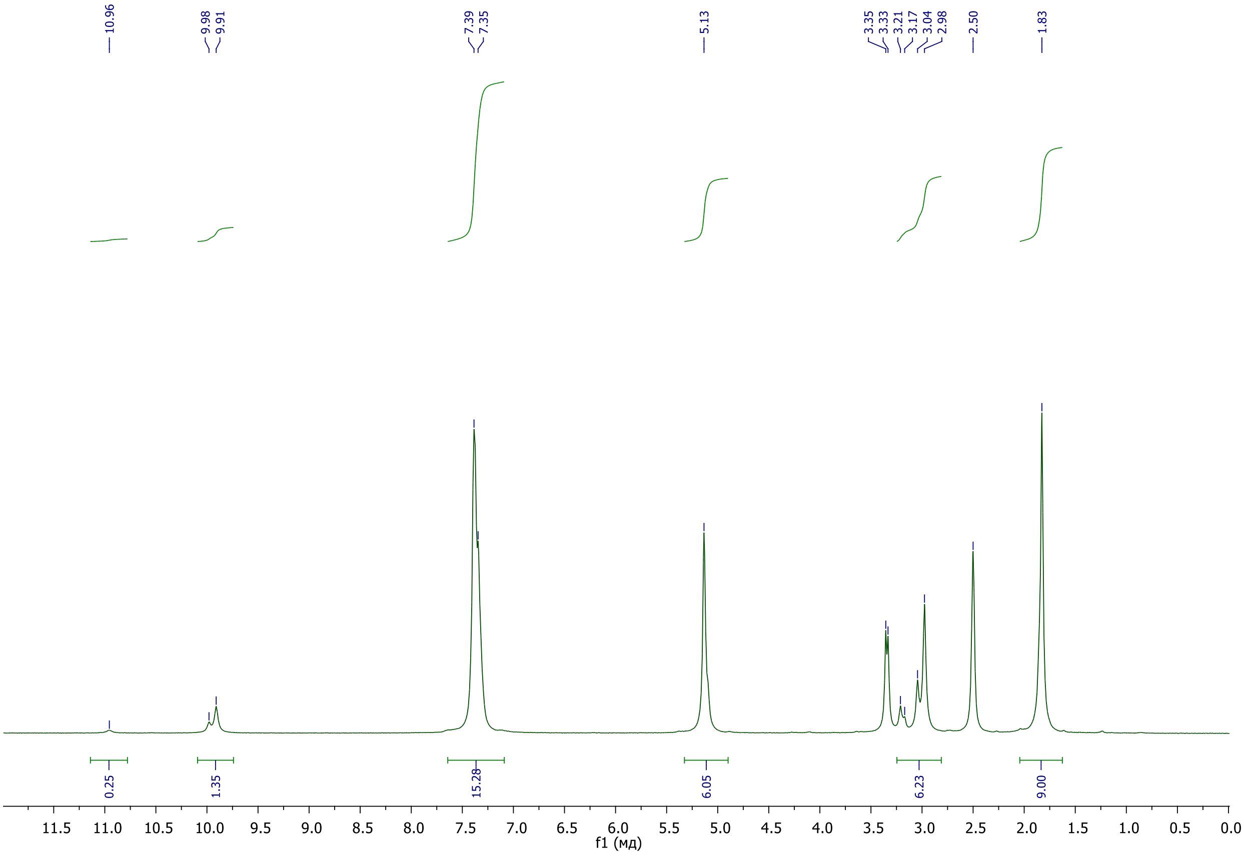

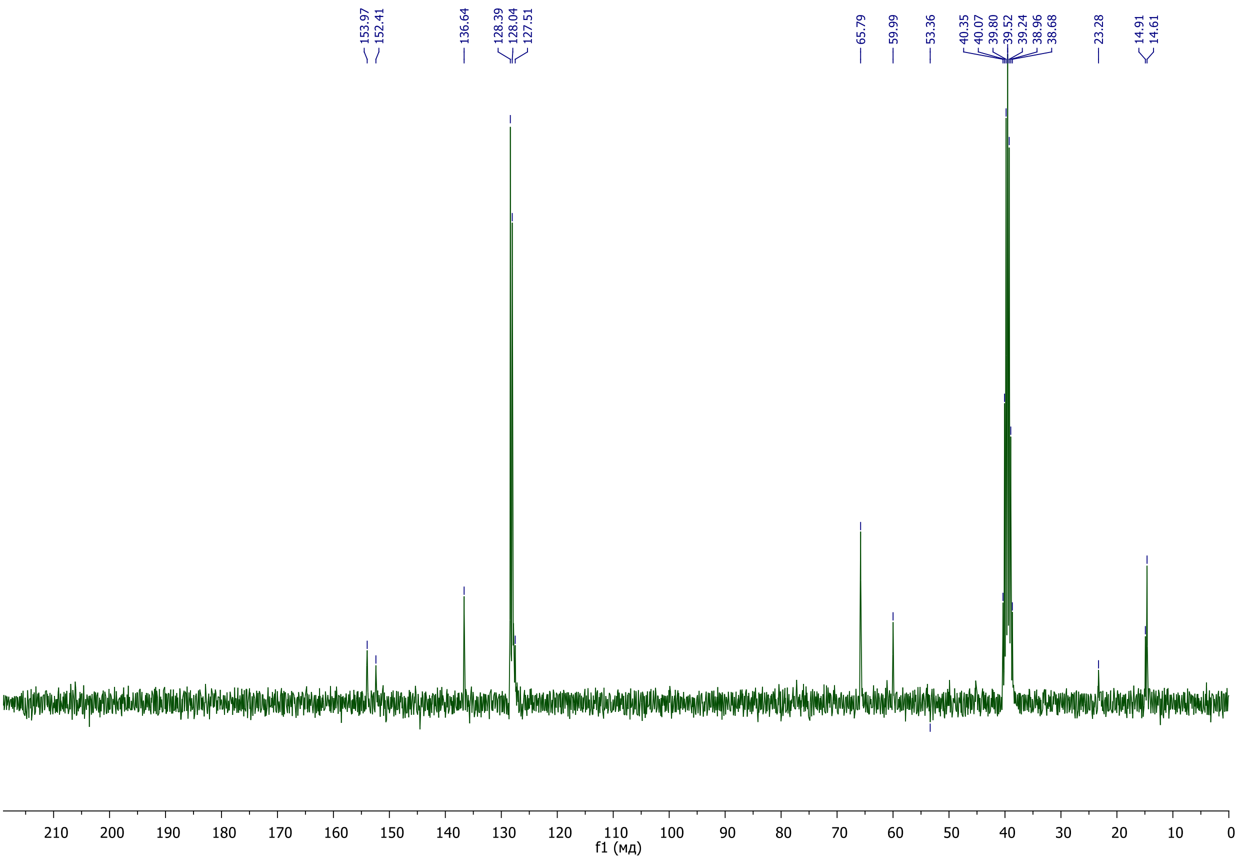
153.97
152.41
136.64
128.39
128.04
127.51
65.79
59.99
53.36
40.35
40.07
39.80
39.52
39.24
38.96
38.68
23.28
14.91
14.61
f1 (мд)

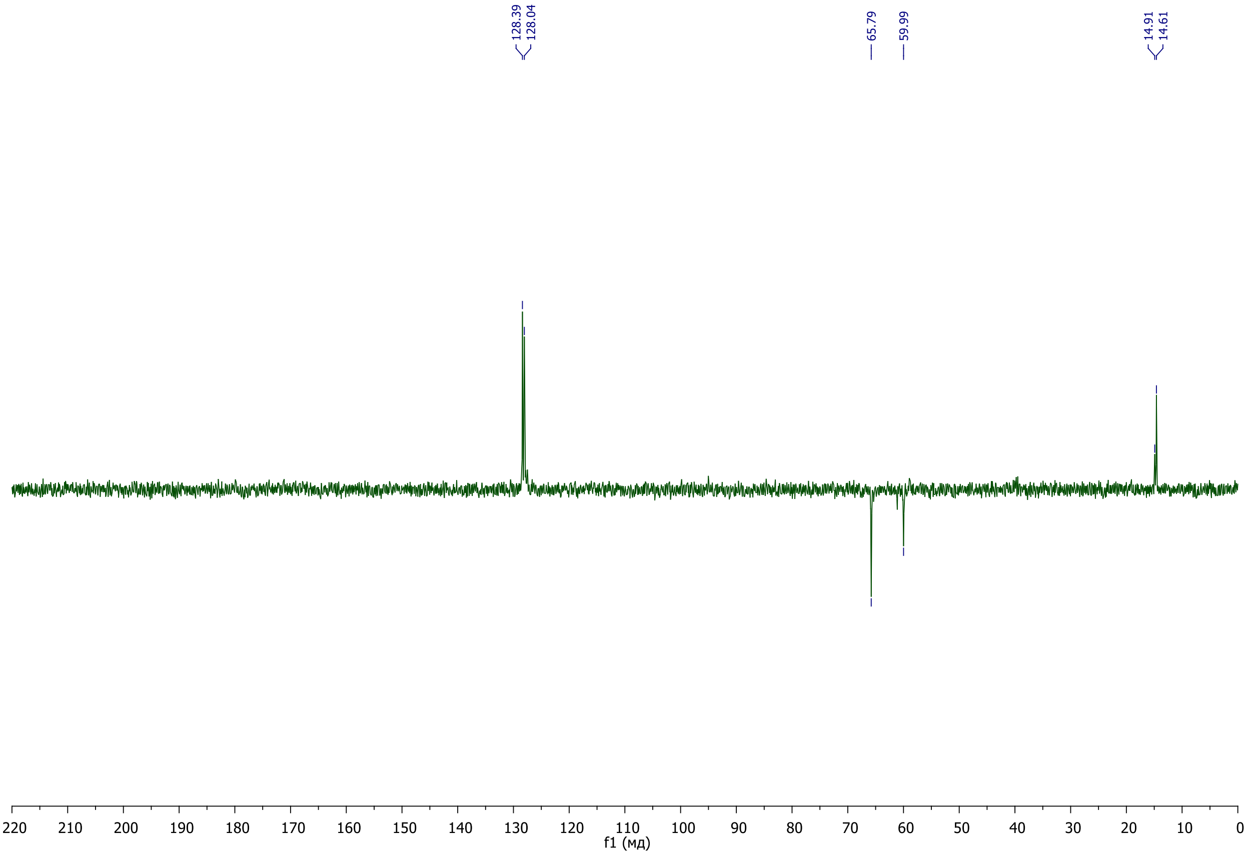
128.39
128.04
65.79
59.99
14.91
14.61
f1 (мд)

Mixed oxime-hydrazone 5a

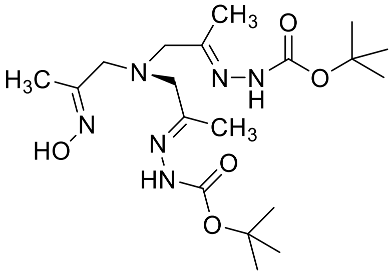

White solid, mp = 136-141 °C. Mixture of isomers and/or rotamers.

^{1}H NMR (300 MHz, DMSO-d6, 297 K): δ = 1.43 (s, 18 H, 2 C(C*H*$_3$)$_3$), 1.75 (s, 3 H, C*H*$_3$), 1.80 (s, 6 H, 2 C*H*$_3$) 2.93 (s, 6 H, 2 C*H*$_2$ and C*H*$_2$), 9.46 (s, 2 H, 2 N*H*), 10.60 (s, 1 H, O*H*).

^{1}H NMR (300 MHz, DMSO-d6, 330 K): δ = 1.41 and 1.44 (2 s, 18 H, 2 C(C*H*$_3$)$_3$), 1.77, 1.82 and 1.86 (3 s, 9 H, 2 C*H*$_3$ and C*H*$_3$), 2.9-3.1 (m, 6 H, 2 C*H*$_2$ and C*H*$_2$), 9.26, 10.45 and 10.60 (3 s, 3 H, 2 N*H* and O*H*).

^{13}C NMR (75 MHz, DMSO-d6, 297 K): δ = 12.2 (*C*H$_3$), 14.4 (2 *C*H$_3$), 28.1 (2 C(*C*H$_3$)$_3$), 57.3 (*C*H$_2$), 60.0 (2 *C*H$_2$), 79.0 (2 *C*(CH$_3$)$_3$). Signals of *C*=O, *C*=N and *C*=N are not observed due to broadening.

^{13}C NMR (75 MHz, DMSO-d6, 330 K): δ = 11.9, 12.0, 14.1, 14.5 and 23.2 (2 *C*H$_3$ and *C*H$_3$), 27.8 and 27.9 (2 C(*C*H$_3$)$_3$), 55.4, 57.2, 58.3, 59.9 and 60.8 (2 *C*H$_2$ and *C*H$_2$), 151.4, 152.7, 152.8 and 153.3 (2 *C*=O, 2 *C*=N and *C*=N).

HRMS: Calcd for $C_{19}H_{36}N_6O_5Na^+$ [M+Na$^+$] m/z: 451.2639. Found: 451.2633.

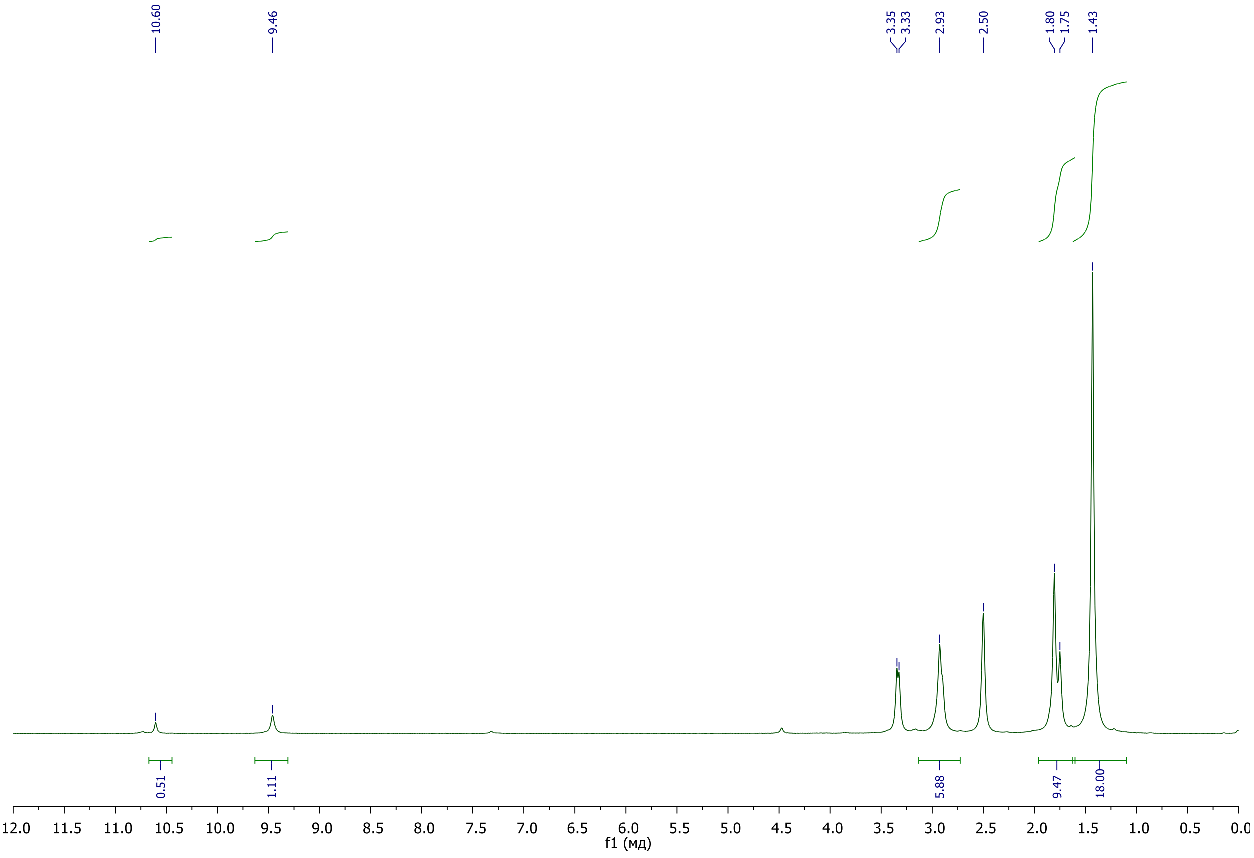

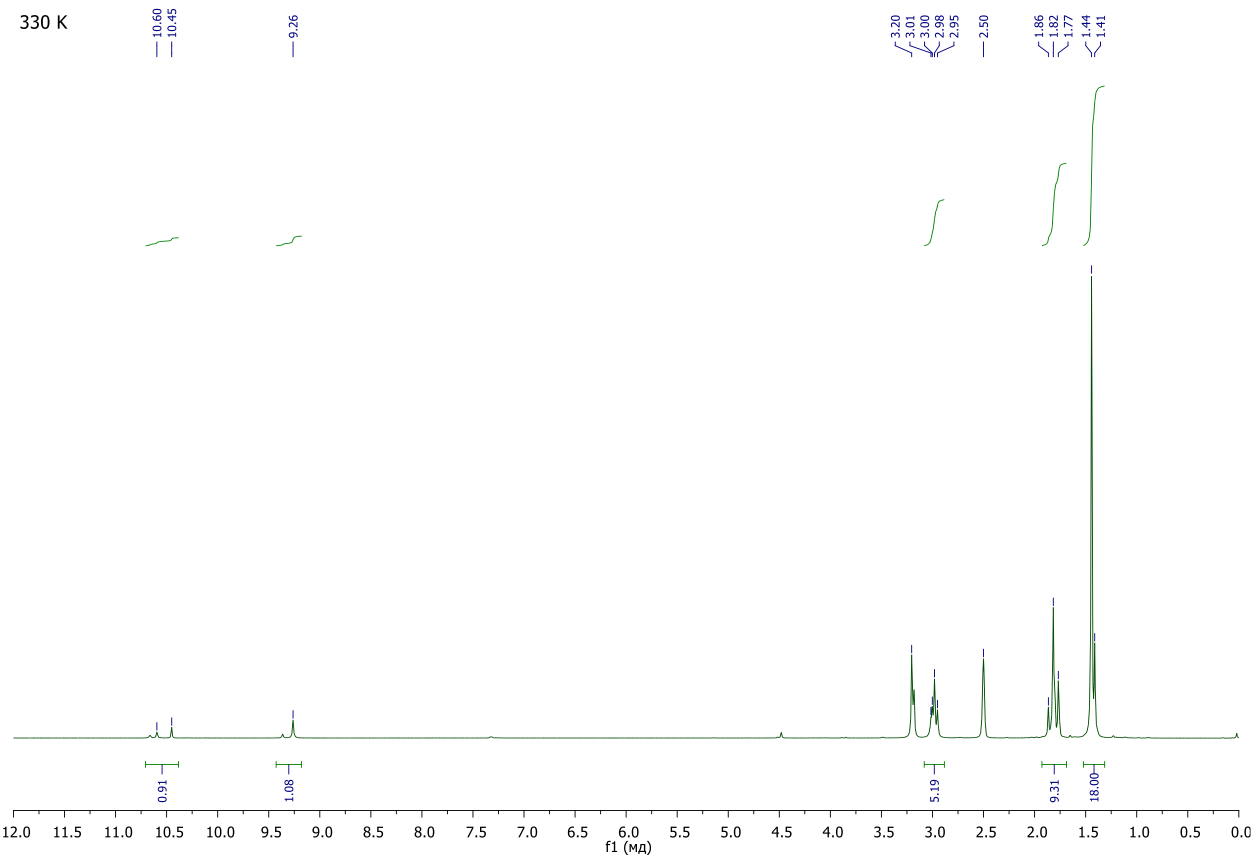
330 K
10.60
10.45
9.26
3.20
3.01
3.00
2.98
2.95
2.50
1.86
1.82
1.77
1.44
1.41
0.91
1.08
5.19
9.31
18.00
12.0 11.5 11.0 10.5 10.0 9.5 9.0 8.5 8.0 7.5 7.0 6.5 6.0 5.5 5.0 4.5 4.0 3.5 3.0 2.5 2.0 1.5 1.0 0.5 0.0
f1 (мд)

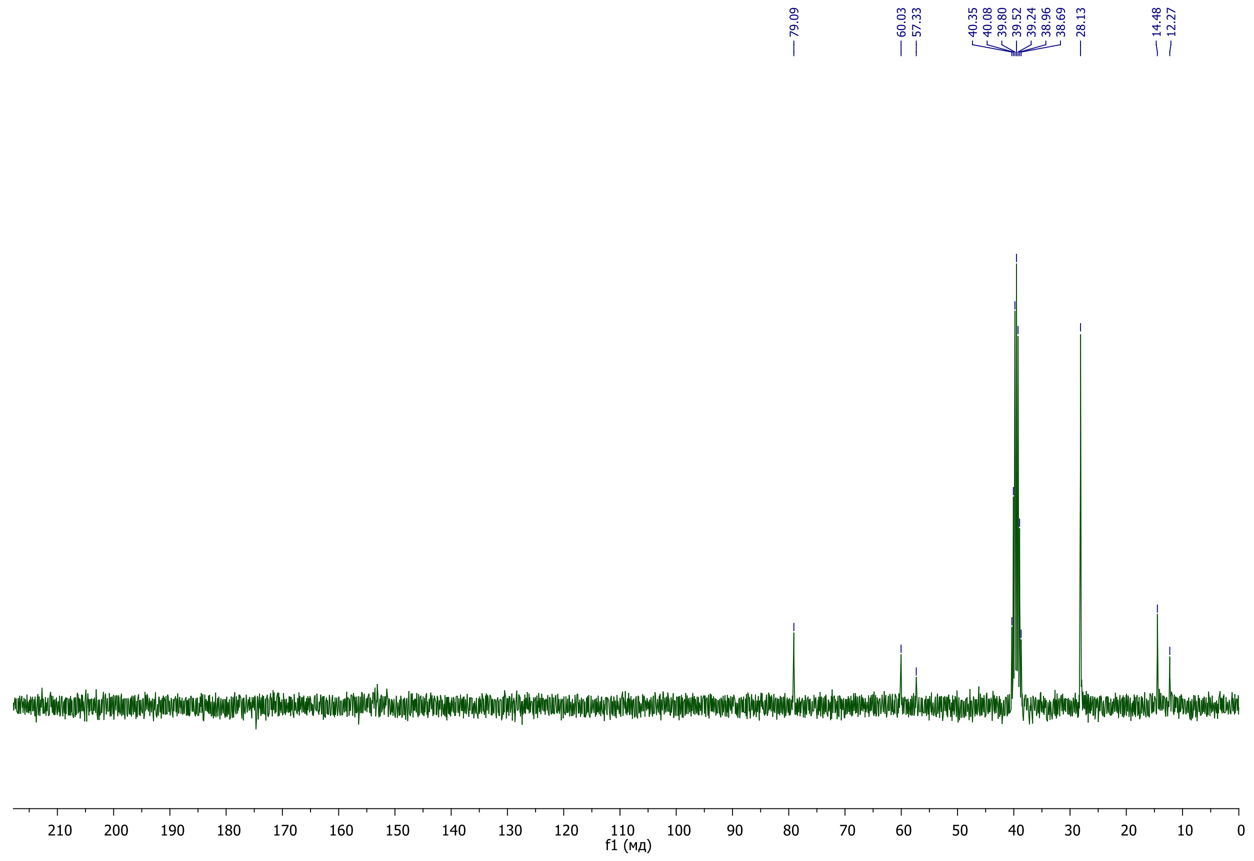
79.09
60.03
57.33
40.35
40.08
39.80
39.52
39.24
38.96
38.69
28.13
14.48
12.27
210 200 190 180 170 160 150 140 130 120 110 100 90 80 70 60 50 40 30 20 10 0
f1 (мд)

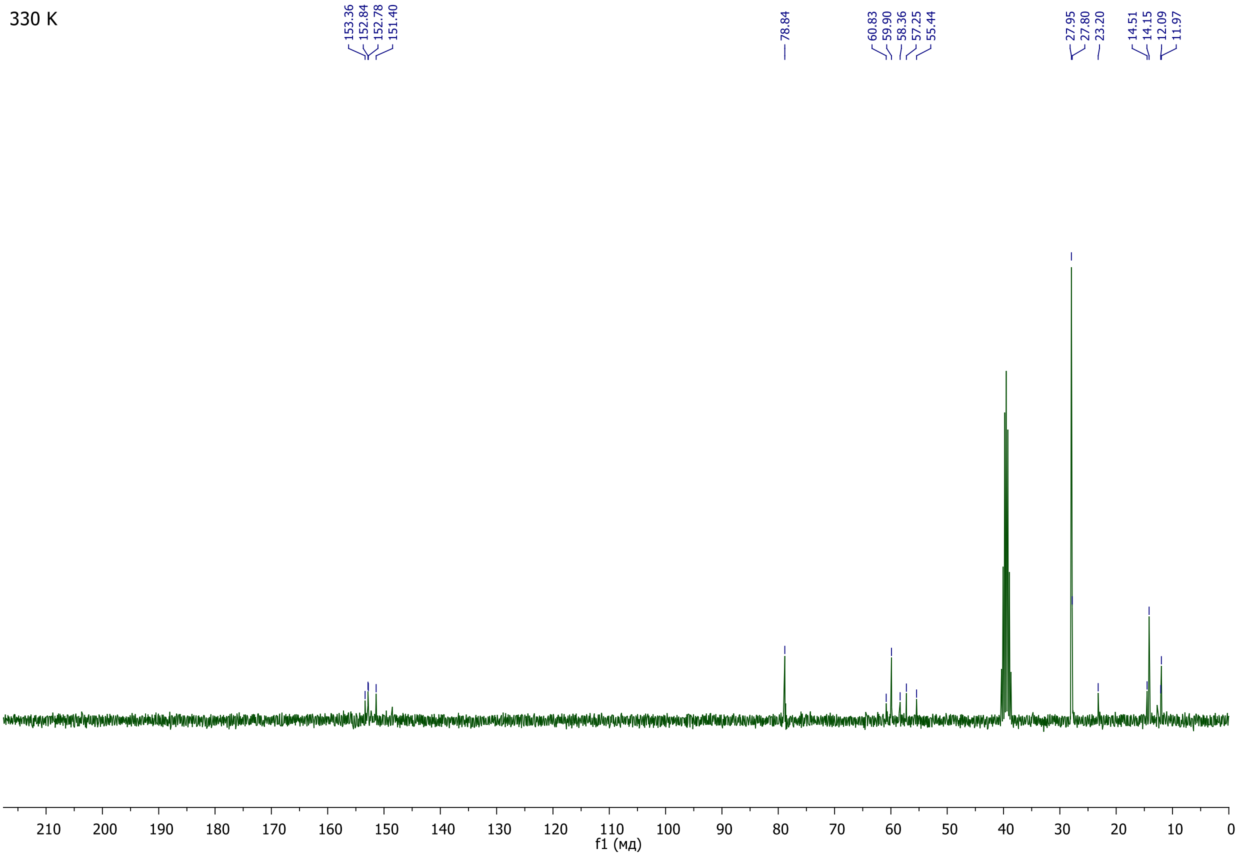

330 K
153.36
152.84
152.78
151.40
78.84
60.83
59.90
58.36
57.25
55.44
27.95
27.80
23.20
14.51
14.15
12.09
11.97
210 200 190 180 170 160 150 140 130 120 110 100 90 80 70 60 50 40 30 20 10 0
f1 (мд)

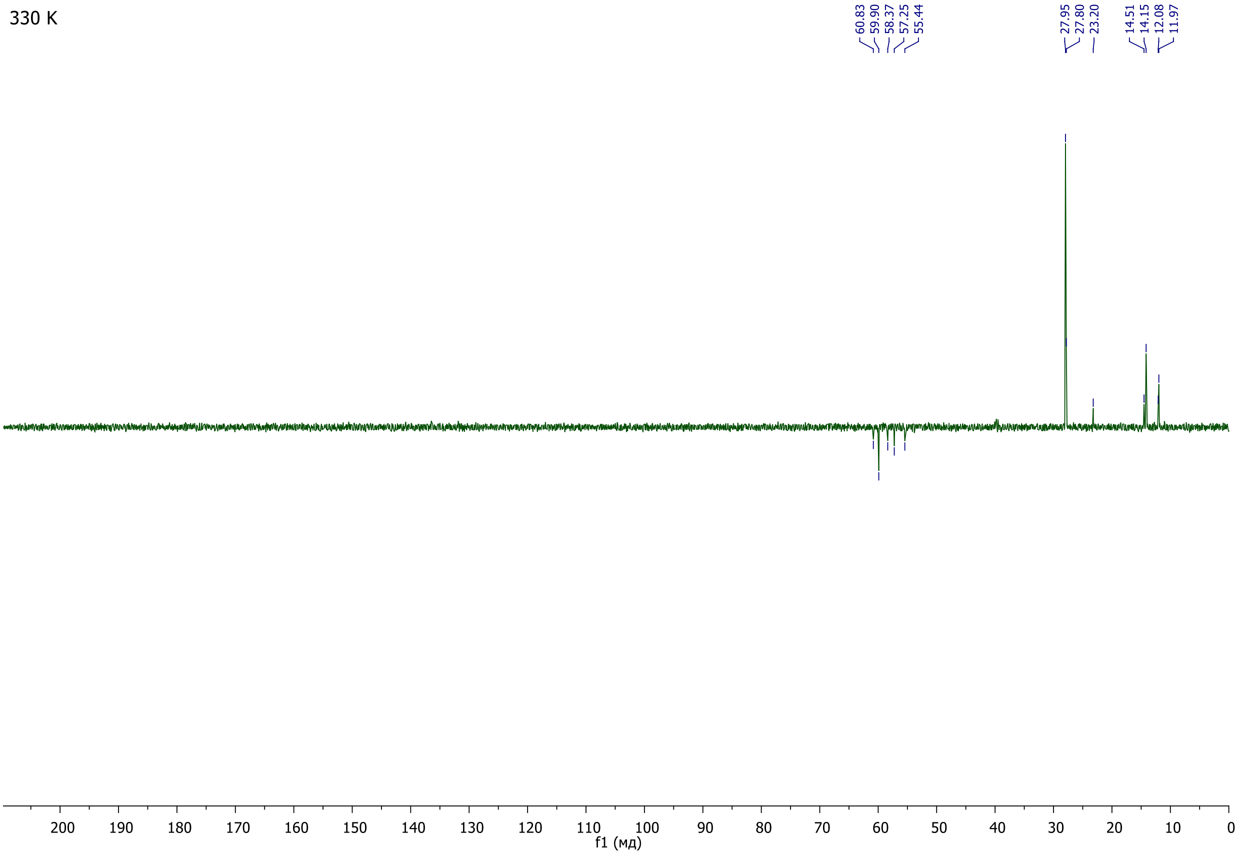

330 K
60.83
59.90
58.37
57.25
55.44
27.95
27.80
23.20
14.51
14.15
12.08
11.97
200 190 180 170 160 150 140 130 120 110 100 90 80 70 60 50 40 30 20 10 0
f1 (мд)

Mixed oxime-hydrazone 7a

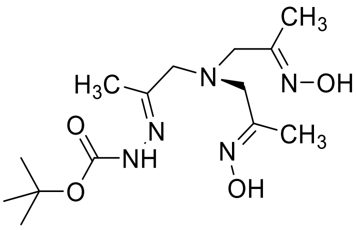

White solid, mp = 189-193 °C. Mixture of isomers and/or rotamers.

^{1}H NMR (300 MHz, DMSO-d6): main isomer, δ = 1.43 (s, 9 H, C(C*H*$_3$)$_3$), 1.75 (s, 6 H, 2 C*H*$_3$), 1.80 (s, 3 H, C*H*$_3$), 2.90 (s, 4 H, 2 C*H*$_2$), 2.93 (s, 2 H, C*H*$_2$), 9.46 (s, 1 H, N*H*), 10.61 (s, 2 H, 2 O*H*); selected signals of minor isomers, δ = 1.39 (C(C*H*$_3$)$_3$), 1.80 and 1.86 (2 C*H*$_3$ and C*H*$_3$), 3.15 (2 C*H*$_2$ and C*H*$_2$).

^{13}C NMR (75 MHz, DMSO-d6): δ = main isomer, δ = 12.2 (2 *C*H$_3$), 14.4 (*C*H$_3$), 28.1 (C(*C*H$_3$)$_3$), 57.2 (2 *C*H$_2$), 60.0 (*C*H$_2$), 79.1 (*C*(CH$_3$)$_3$), 151.4, 153.1 and 153.6 (2 *C*=N, *C*=N and *C*=O); selected signals of minor isomers, δ = 12.4 and 23.6 (*C*H$_3$), 28.0 (C(*C*H$_3$)$_3$), 58.3 (*C*H$_2$), 152.4 (*C*=N or *C*=O).

HRMS: Calcd for $C_{15}H_{29}N_4O_4Na^+$ [(M-NH$_2$OH+CH$_3$OH)+Na$^+$] m/z: 352.2081. Found: 352.2087.

For $C_{14}H_{27}N_5O_4$ calcd: C 51.05%, H 8.26%, N 21.26%. Found: C 51.01%, H 8.04%, N 21.17%.

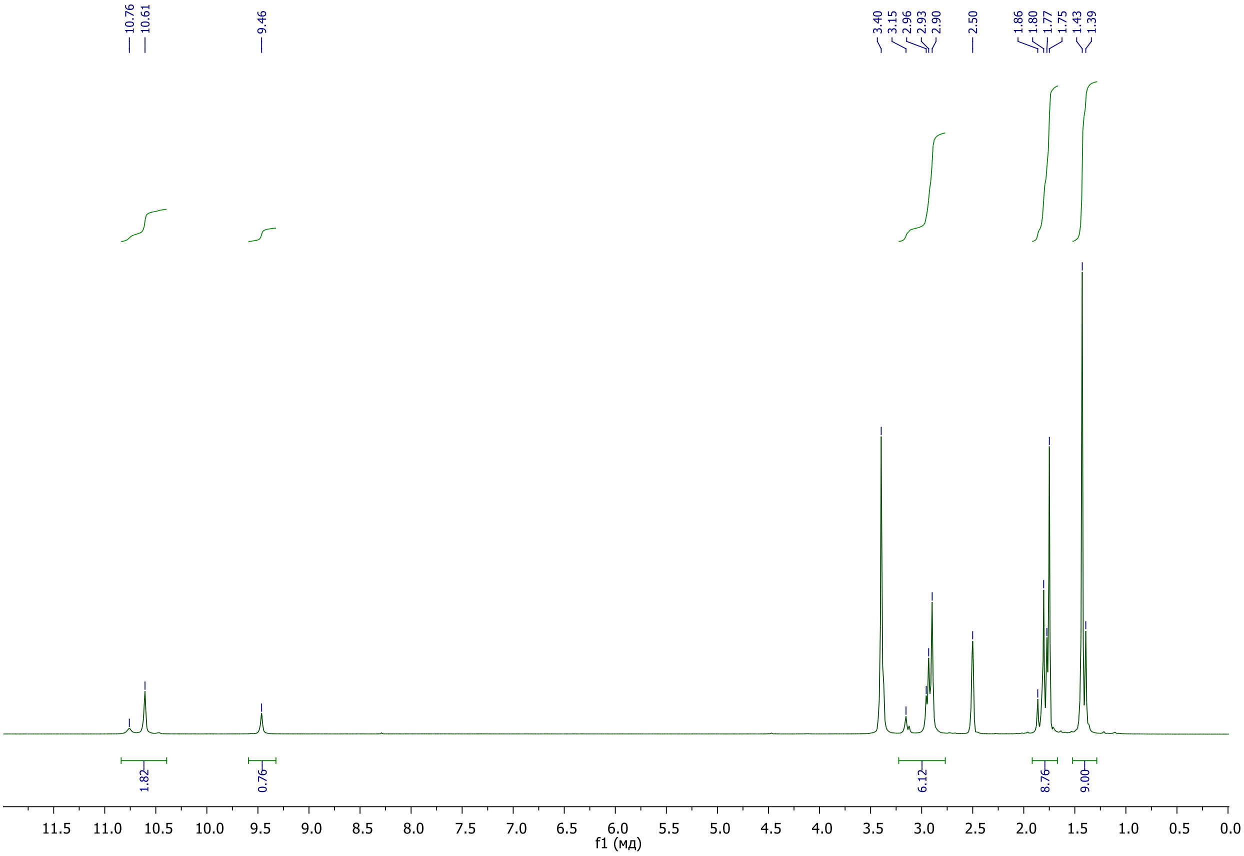

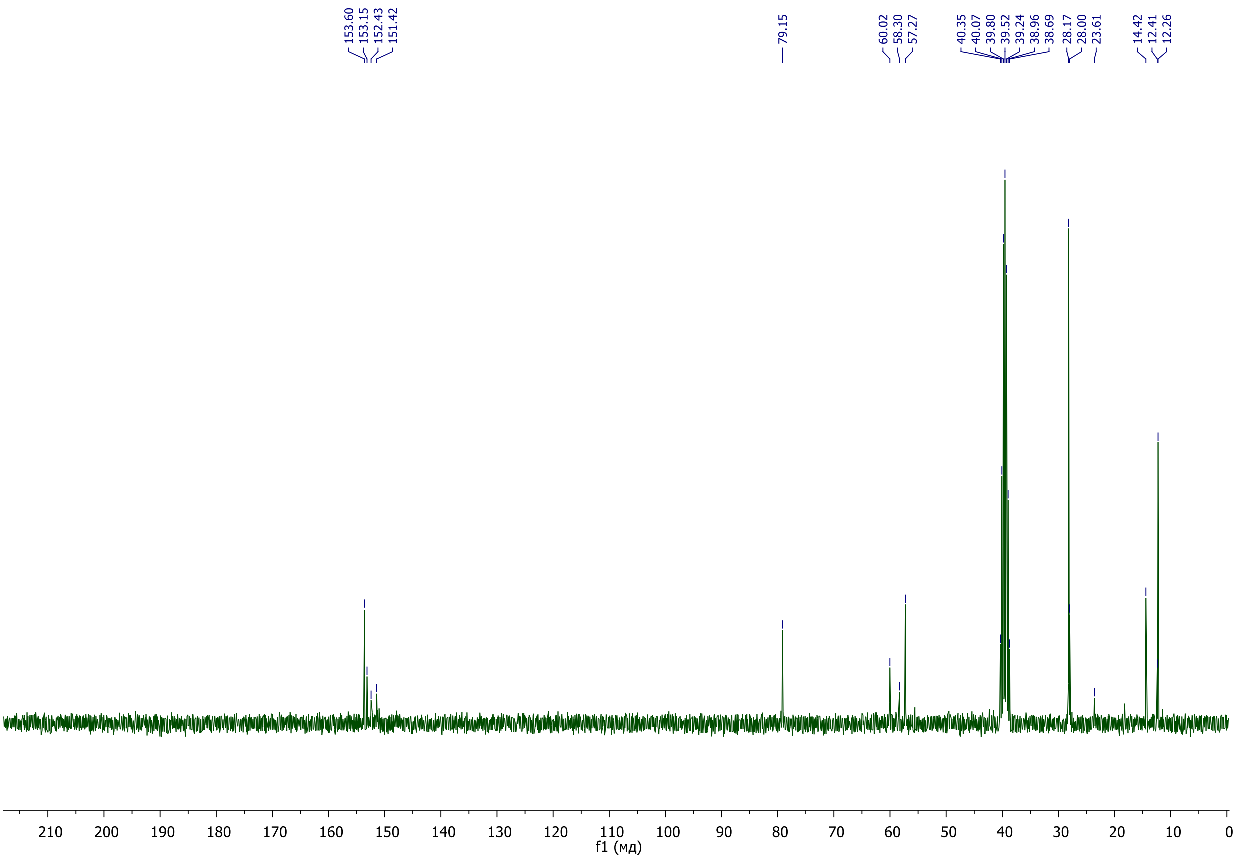
153.60
153.15
152.43
151.42
79.15
60.02
58.30
57.27
40.35
40.07
39.80
39.52
39.24
38.96
38.69
28.17
28.00
23.61
14.42
12.41
12.26
210 200 190 180 170 160 150 140 130 120 110 100 90 80 70 60 50 40 30 20 10 0
f1 (мд)

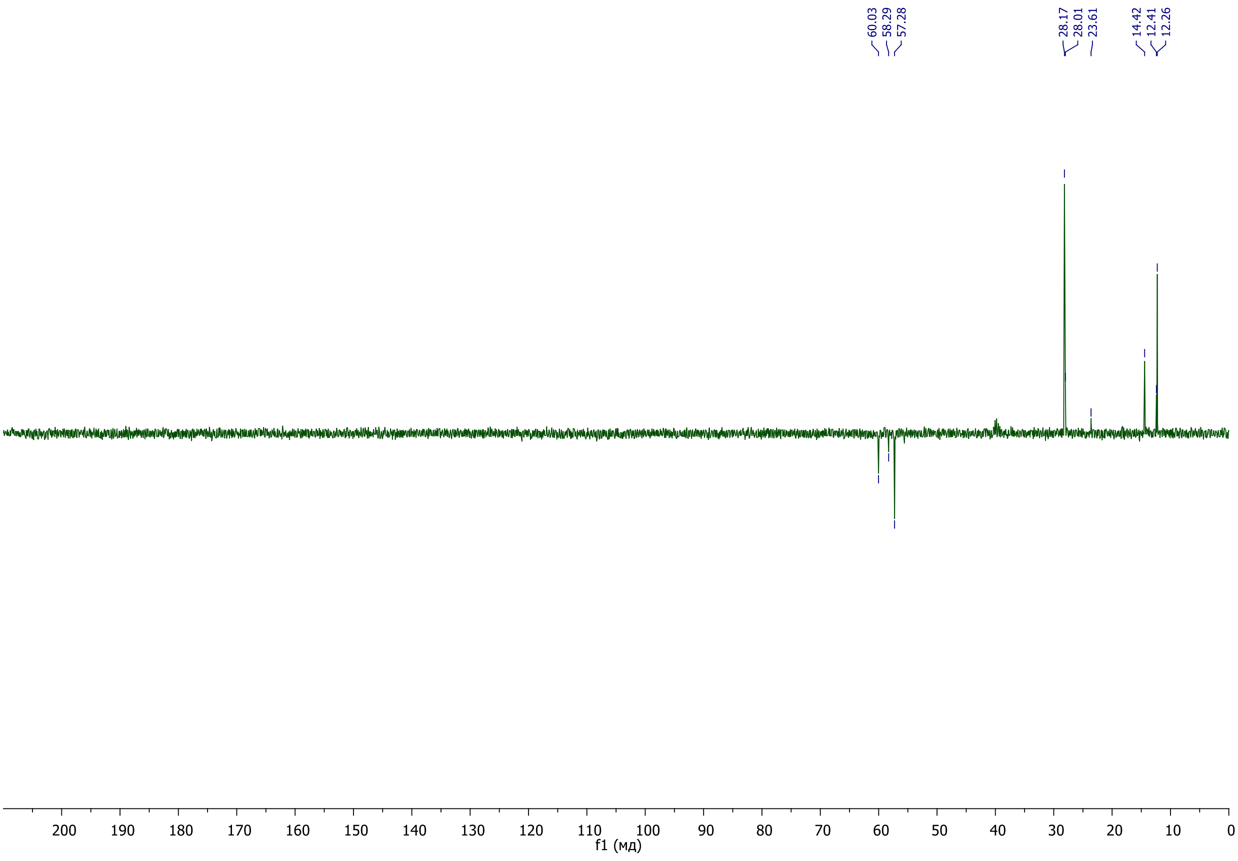
60.03
58.29
57.28
28.17
28.01
23.61
14.42
12.41
12.26
200 190 180 170 160 150 140 130 120 110 100 90 80 70 60 50 40 30 20 10 0
f1 (мд)

TAAD 4a

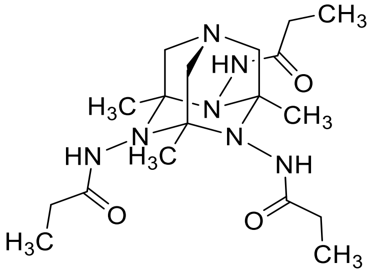

Pale yellow solid, mp = 169-173 °C

^{1}H NMR (300 MHz, D_2O): δ = 1.04 and 1.1-1.25 (s and m, 18 H, 3 C*H*$_3$ and 3 CH_2C*H*$_3$), 2.25-2.4 (m, 6 H, 3 C*H*$_2$$CH_3$), 3.12 and 3.34 (2 d, *J* = 14.1 Hz, 4 H, 3 C*H*$_2$), 3.32 (s, *2* H, C*H*$_2$).

^{13}C NMR (75 MHz, D_2O): δ = 9.5 and 9.9 (3 CH_2*C*H_3), 17.6 and 19.3 (3 *C*H_3), 27.3 (3 *C*$H_2$$CH_3$), 52.7 and 59.5 (3 *C*$H_2$), 73.7 and 7.38 (3 N*C*N), 177.8 and 179.0 (3 *C*=O).

HRMS: Calcd for $C_{18}H_{33}N_7O_3Na^+$ [M+Na^+] m/z: 418.2537. Found: 418.2534.

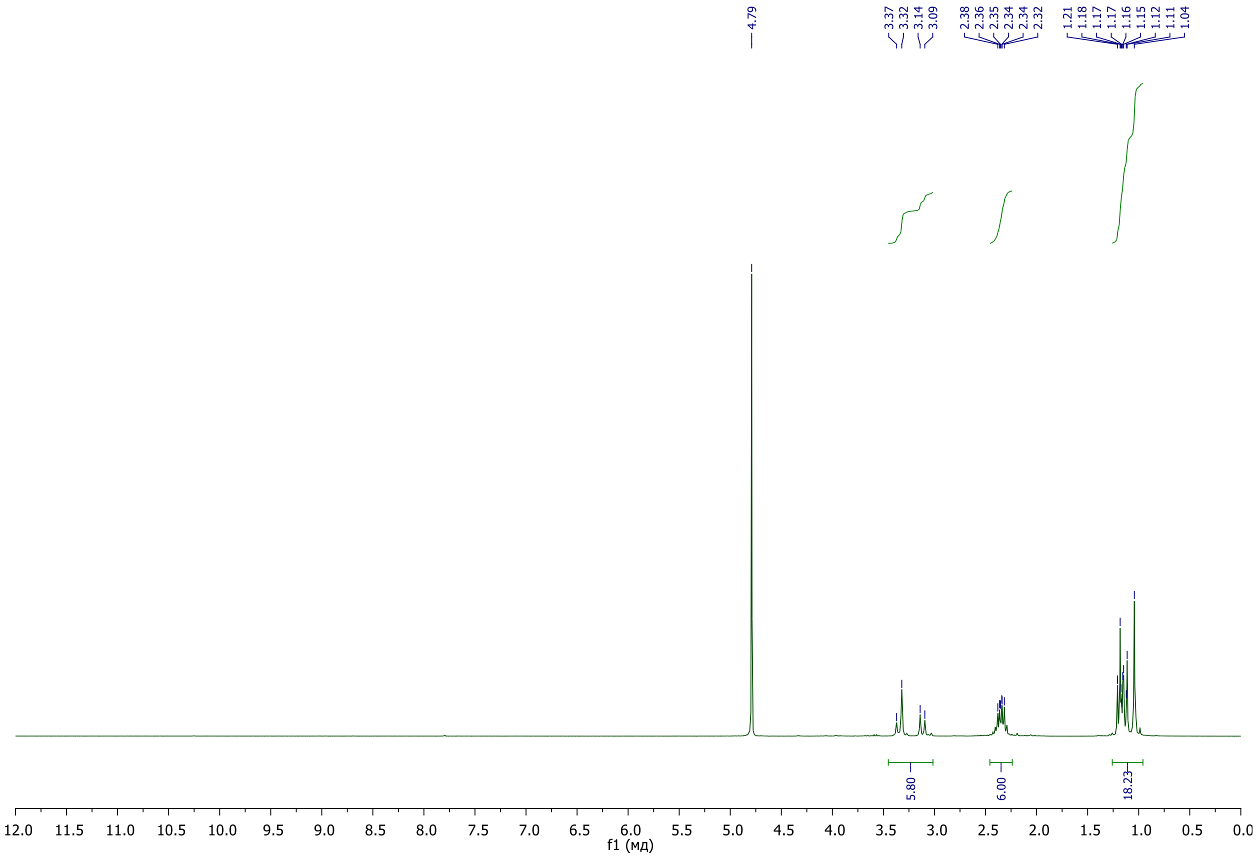

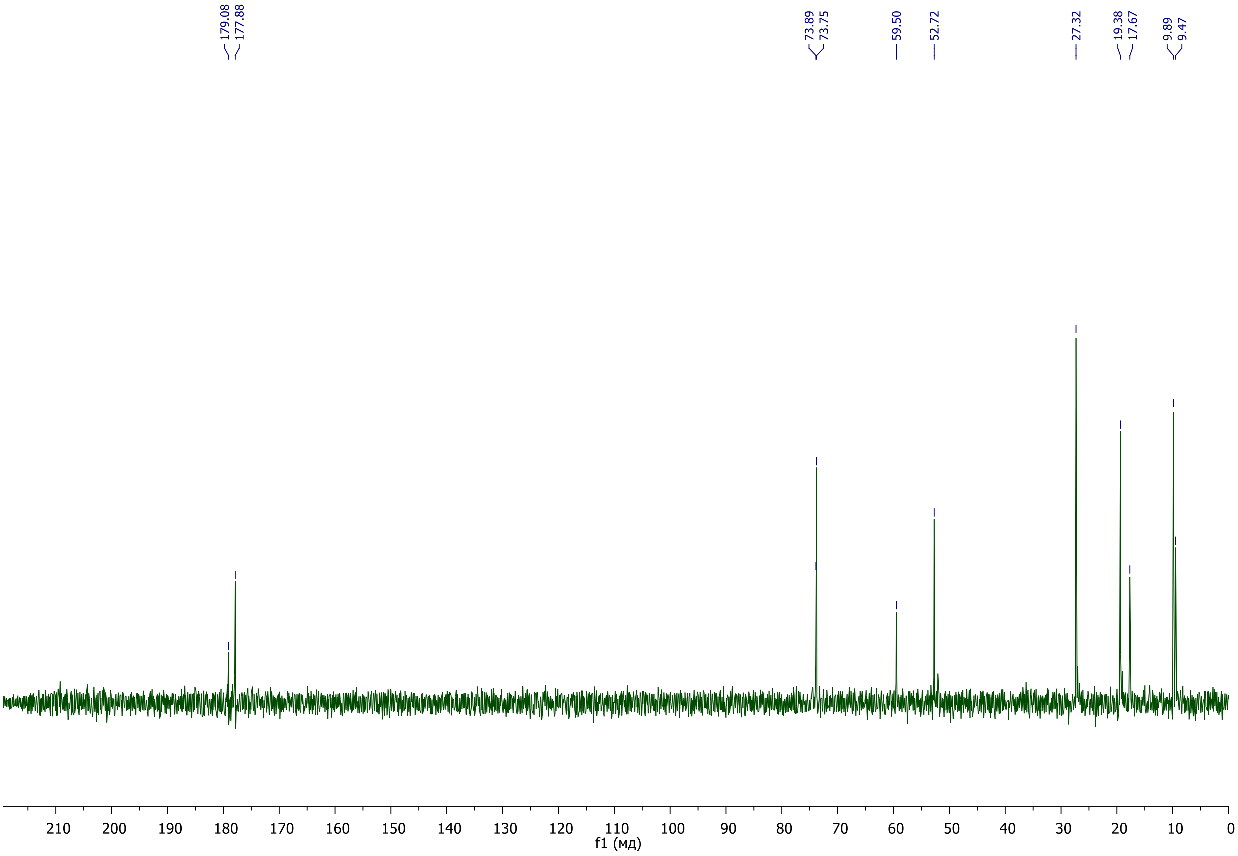
179.08
177.88
73.89
73.75
59.50
52.72
27.32
19.38
17.67
9.89
9.47
210 200 190 180 170 160 150 140 130 120 110 100 90 80 70 60 50 40 30 20 10 0
f1 (мд)

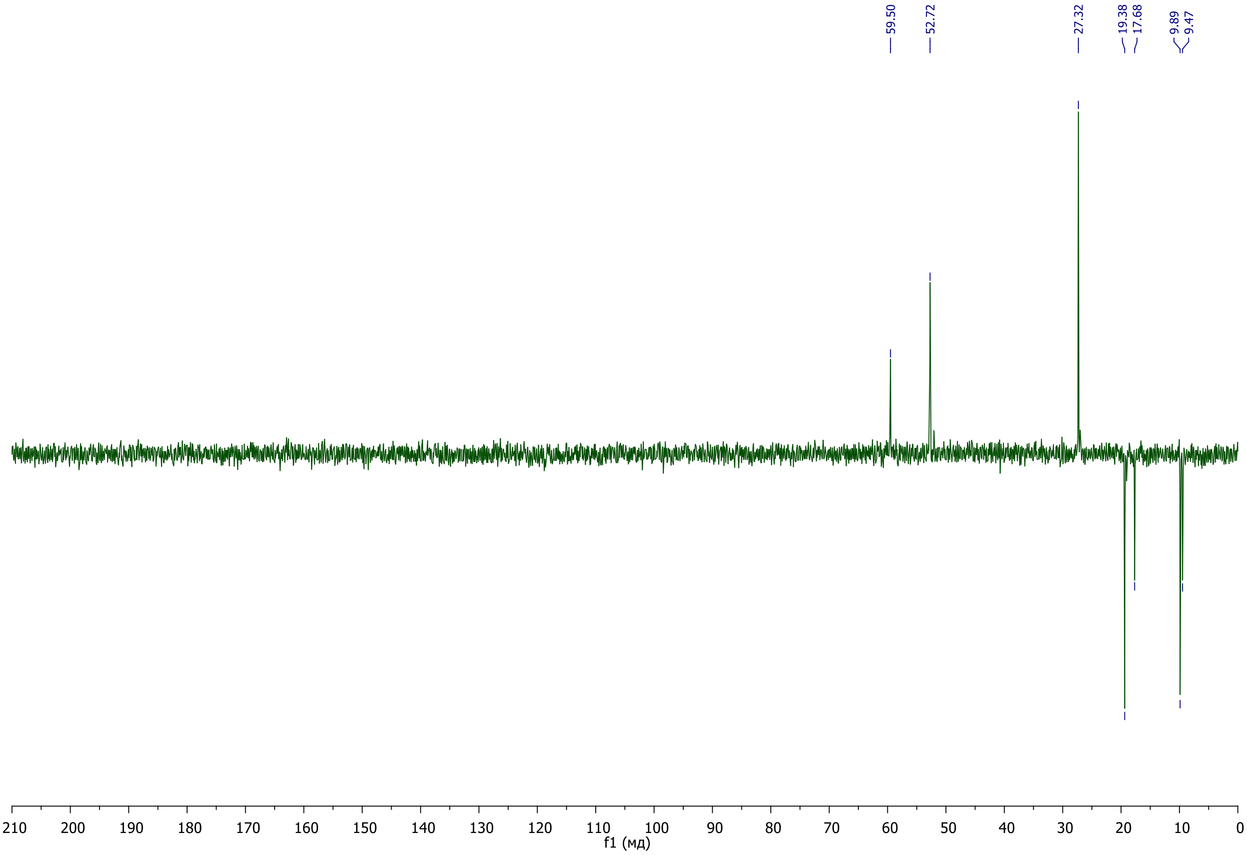
59.50
52.72
27.32
19.38
17.68
9.89
9.47
210 200 190 180 170 160 150 140 130 120 110 100 90 80 70 60 50 40 30 20 10 0
f1 (мд)

TAAD 4b

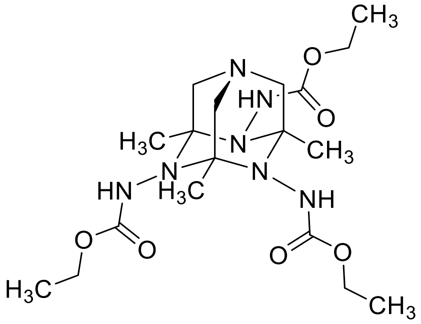

Pale yellow solid.

^{1}H NMR in DMSO-d_6 and melting point in accordance with literature data.[1]

^{1}H NMR (300 MHz, D_2O): δ = 1.07 and 1.20 (2 s, 6 H and 3 H, 3 CH_3), 1.28 (m, 9 H, 3 CH_2CH_3), 3.07 and 3.26 (2 d, J = 13.5 Hz, 4 H, 2 CH_2), 3.28 (s, 2 H, CH_2), 4.17 (q, J = 6.8 Hz, 6 H, 3 CH_2CH$_3$).

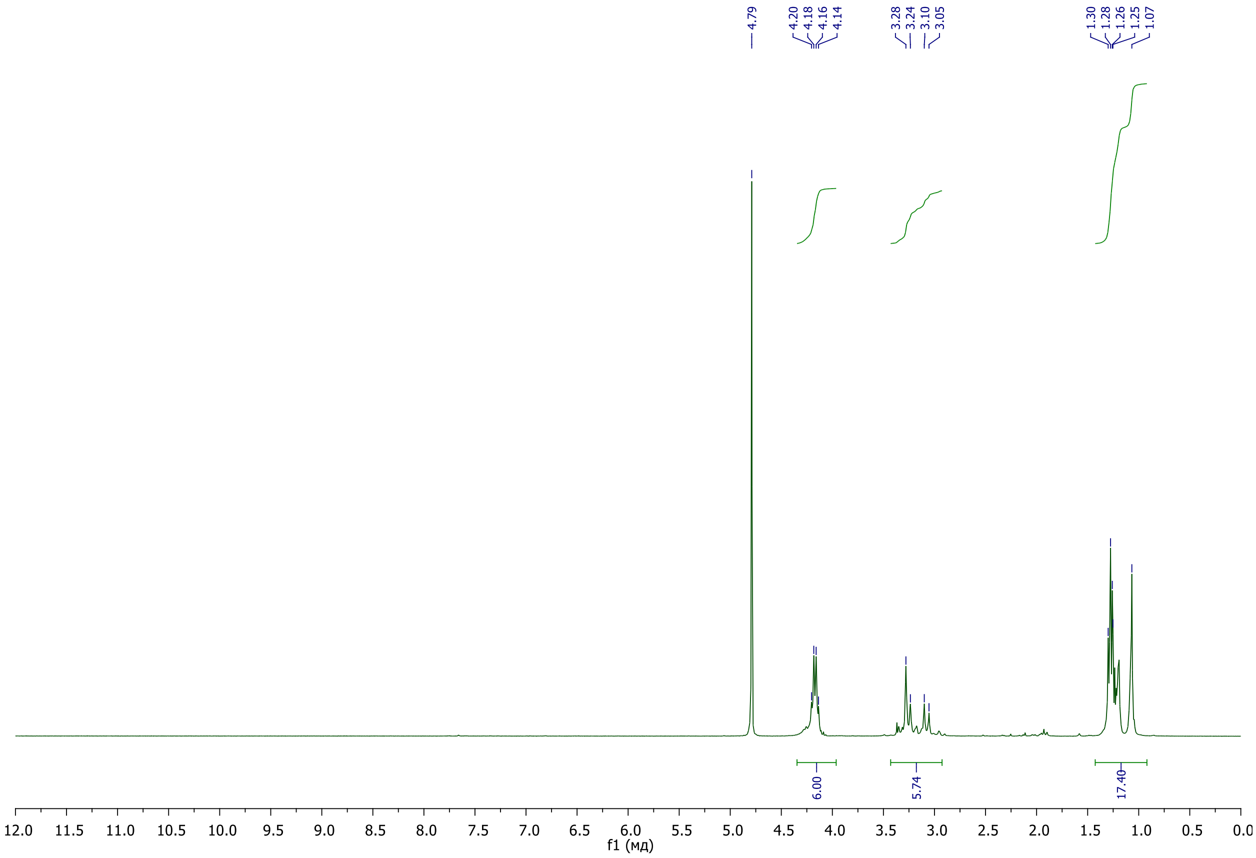

TAAD 4c

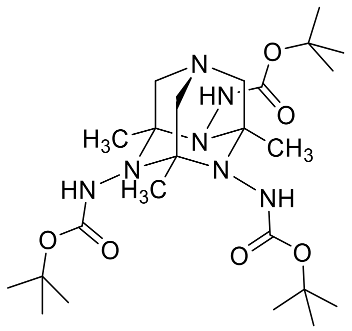

White solid, mp = 238-240 °C (with dec.)

^{1}H NMR (300 MHz, CD_3OD): δ = 0.99 and 1.15 (2 s, 6 H and 3 H, 3 CH_3), 1.51 (s, 27 H, 3 C(CH_3)$_3$), 3.03 and 3.26 (2 d, J = 13.7 Hz, 4 H, 2 CH_2), 3.29 (s, 2 H, CH_2), 9.10 (s, 3 H, 3 NH).

^{13}C NMR (75 MHz, CD_3OD): δ = 18.8 and 20.1 (3 CH$_3$), 28.6 (3 C(CH$_3$)$_3$), 54.7 and 62.1 (3 CH$_2$), 75.0 and 75.7 (3 NCN), 80.9 and 81.9 (3 C(CH$_3$)$_3$), 159.5 and 160.1 (3 C=O).

HRMS: Calcd for $C_{24}H_{46}N_7O_6^+$ [M+H$^+$] m/z: 528.3504. Found: 528.3498.

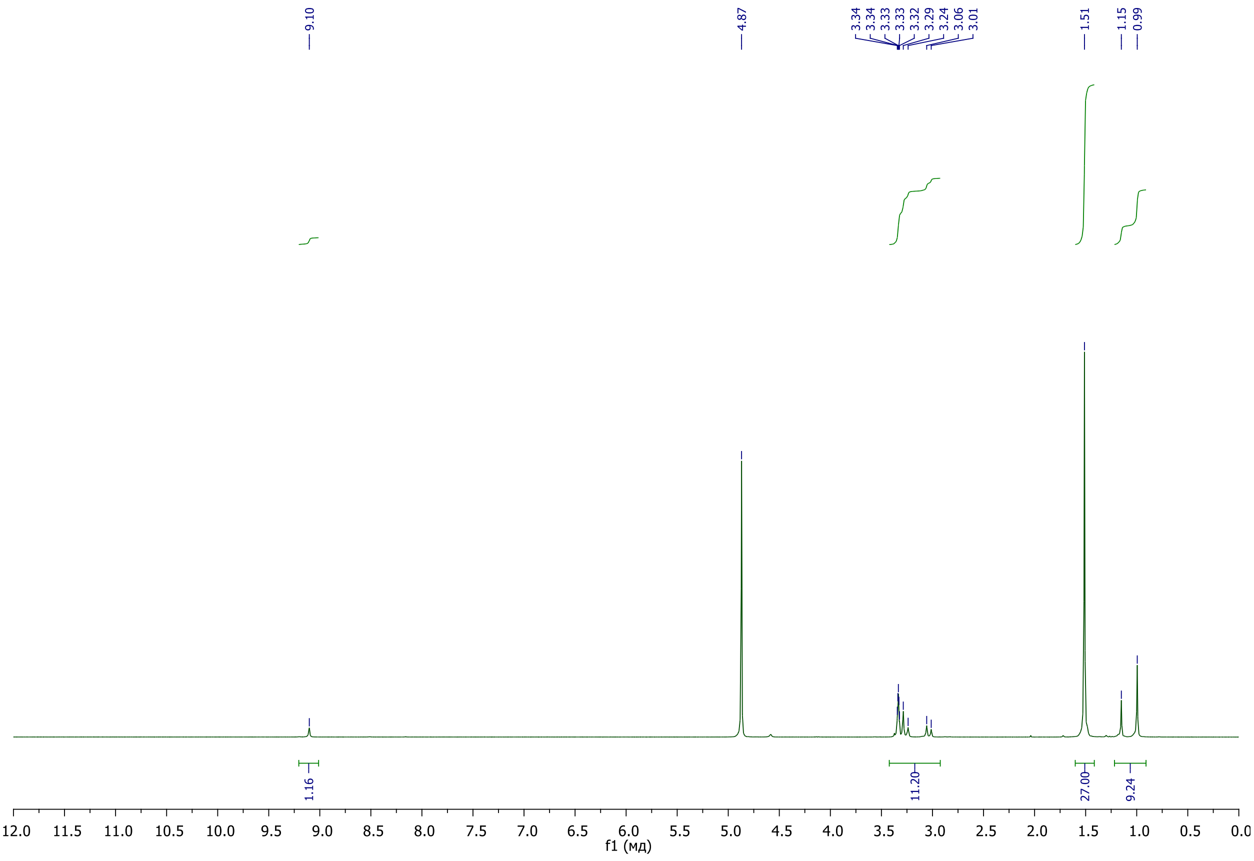

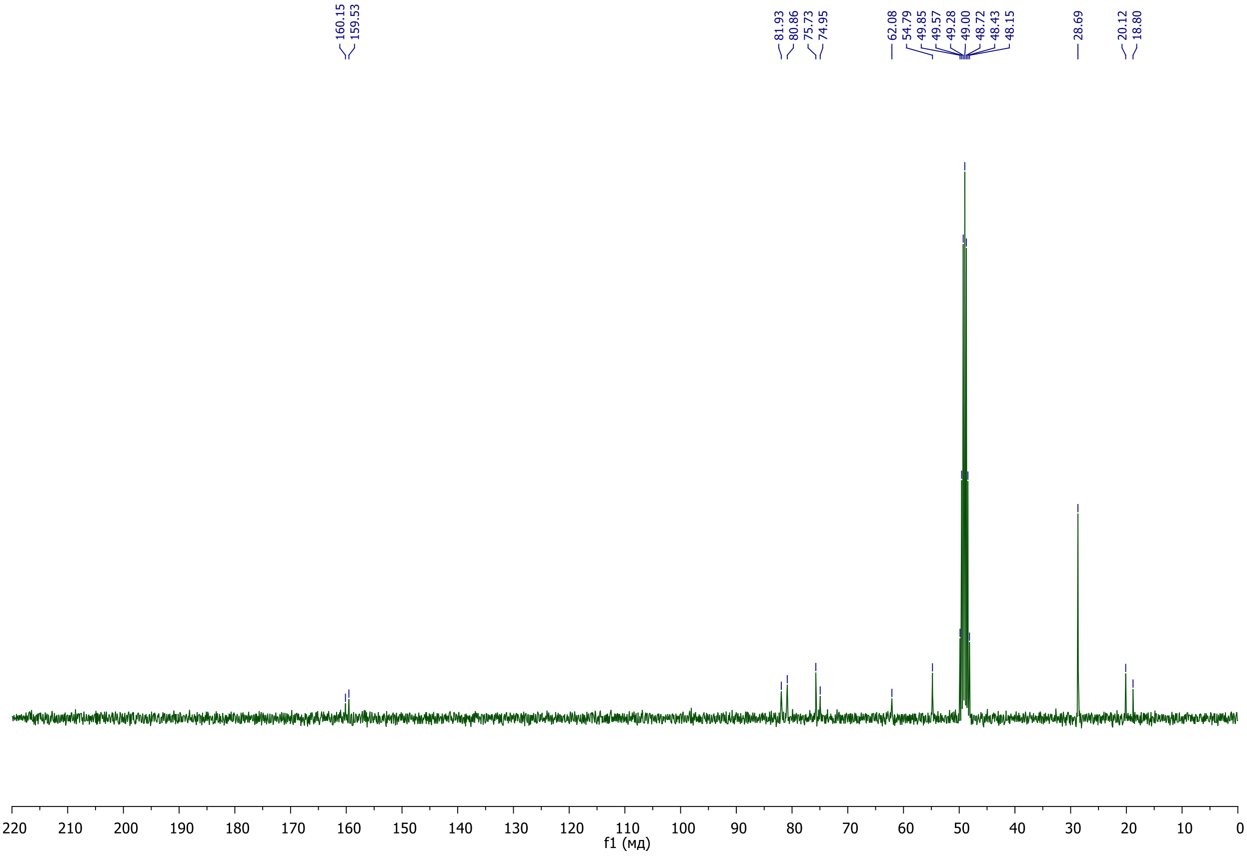
160.15
159.53
81.93
80.86
75.73
74.95
62.08
54.79
49.85
49.57
49.28
49.00
48.72
48.43
48.15
28.69
20.12
18.80
f1 (мд)

TAAD salt 4c·× *t*-BuOH

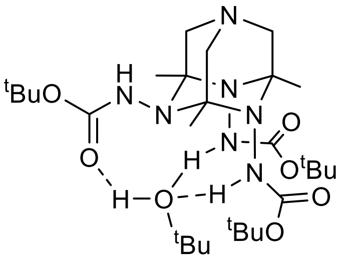

(obtained in competition experiments with *tert*-butanol)

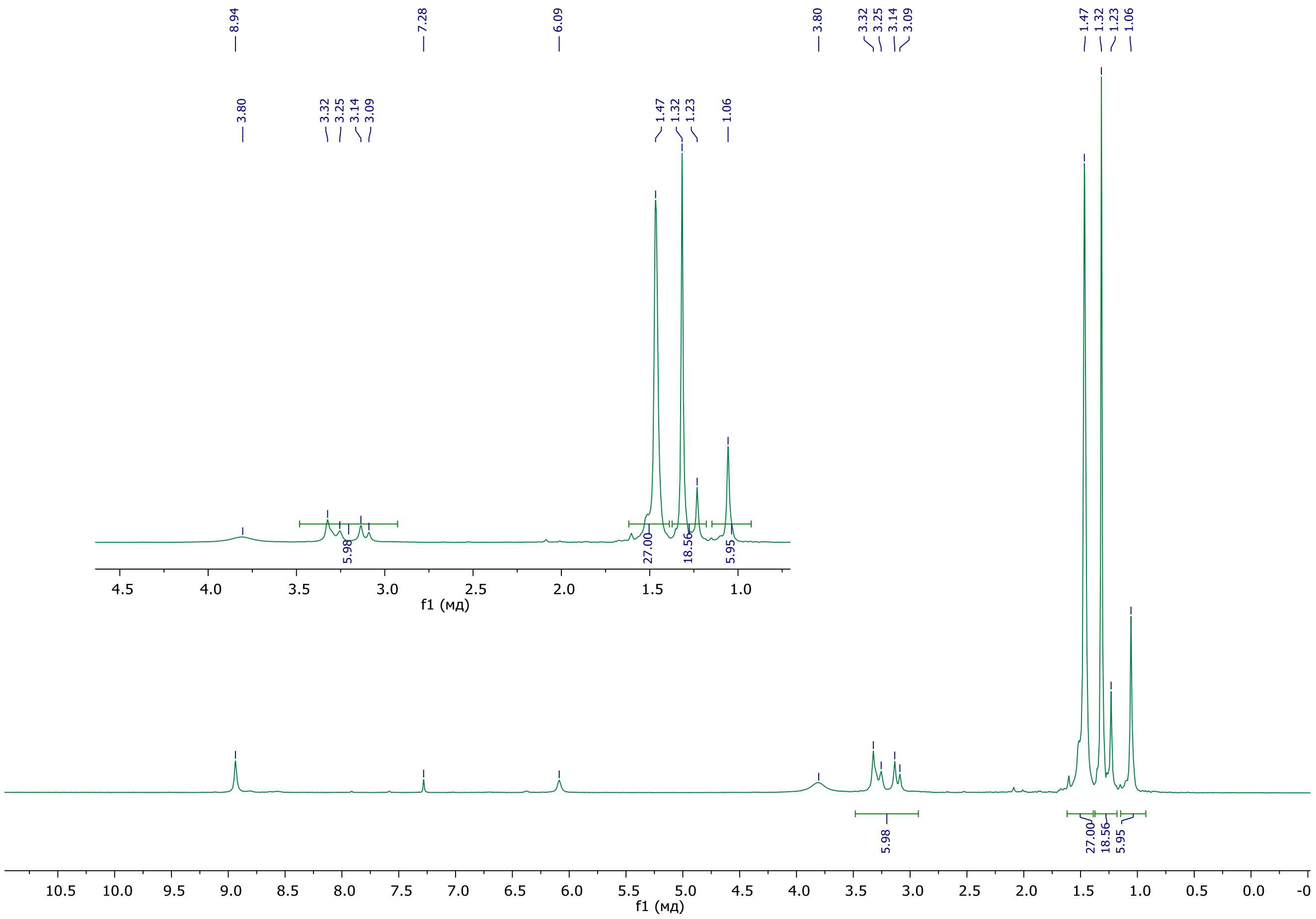

TAAD 4d

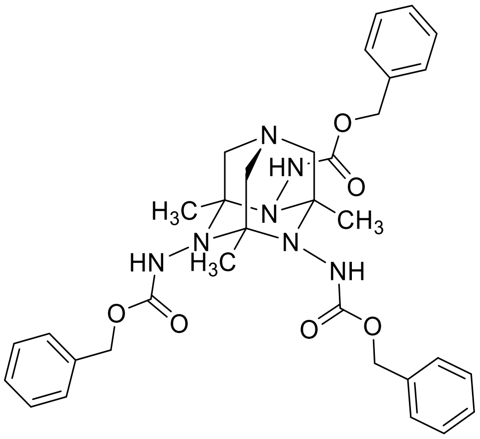

Pale yellow amorphous solid, starts softening at 72 °C, mp = 77-80 °C

^{1}H NMR (300 MHz, CD_3OD): δ = 0.94 and 1.11 (2 s, 9 H, 3 C*H*$_3$), 3.03 and 3.25 (2 d, J = 14.7 Hz, 2 H and 2 H, 2 C*H*$_2$), 3.27 (s, 2H, C*H*$_2$), 5.14-5.25 (m, 6 H, 3 PhC*H*$_2$), 7.2-7.4 (m, 15 H, 3 *Ph*).

^{13}C NMR (75 MHz, CD_3OD): δ = 18.8 and 20.3 (3 *C*H_3), 54.8 and 61.9 (3 *C*H_2), 67.8 and 68.4 (3 N*C*N), 75.2 and 75.7 (3 Ph*C*H_2), 128.6, 129.0, 129.1, 129.3, 129.4 and 129.5 (3 *o,m,p-Ph*), 137.6 and 138.1 (3 *i-Ph*), 159.7 and 160.7 (3 *C*=O).

HRMS: Calcd for $C_{33}H_{40}N_7O_6^+$ [M+H$^+$] m/z: 630.3035. Found: 630.3042.

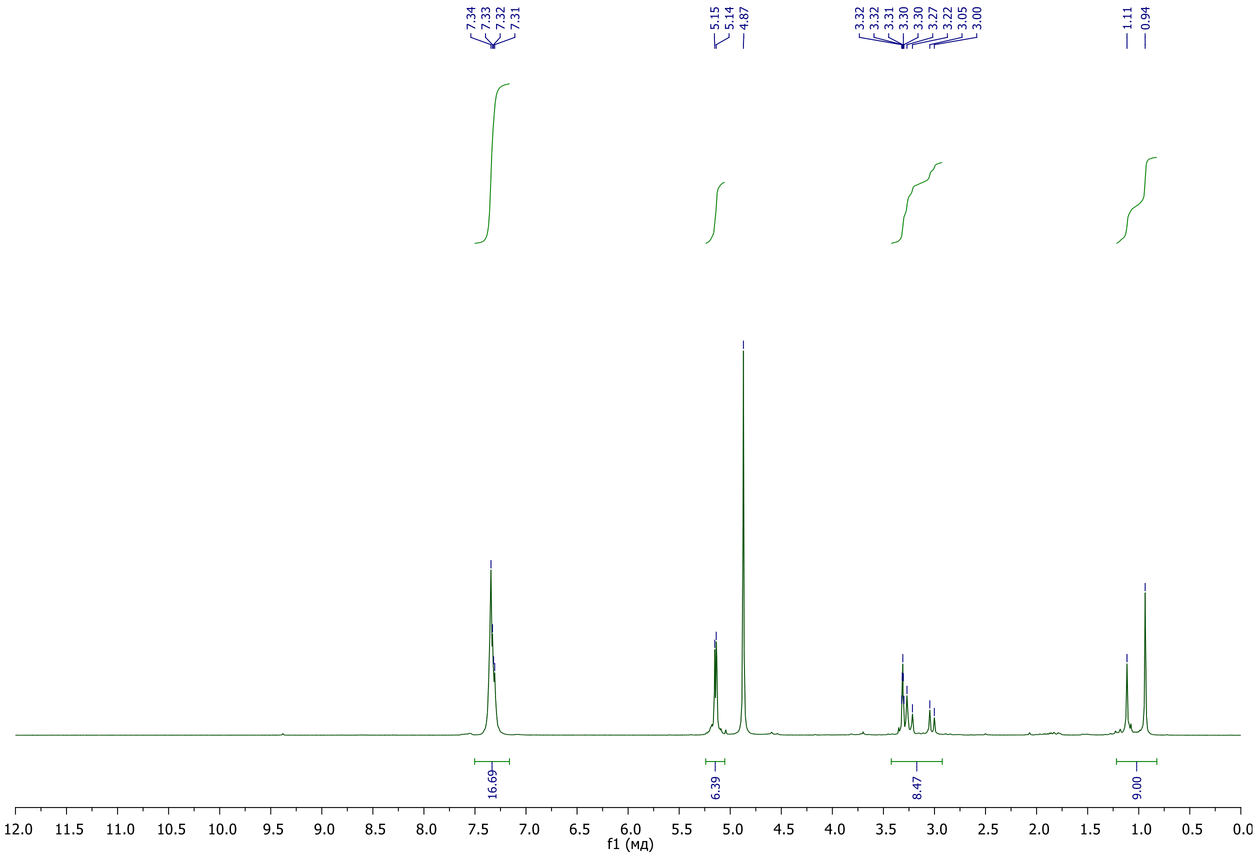

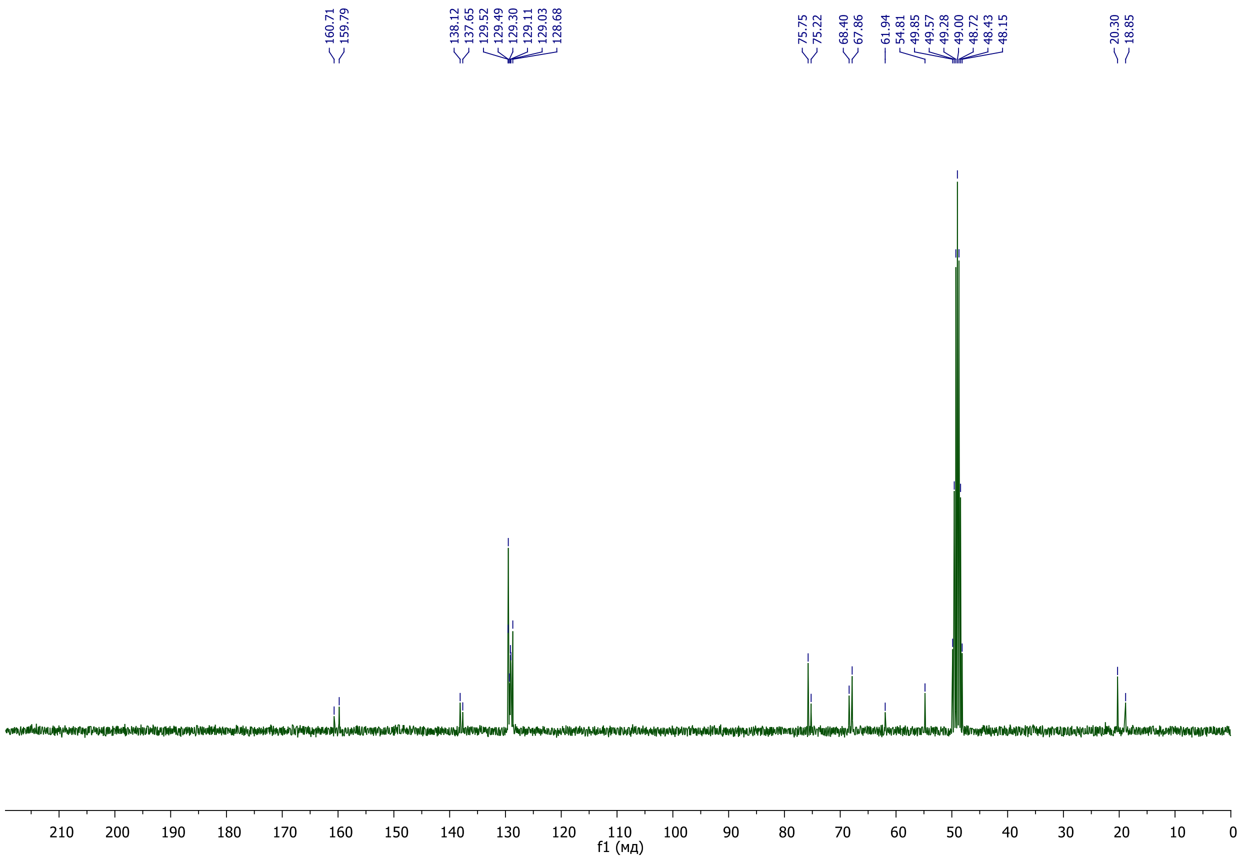
160.71
159.79
138.12
137.65
129.52
129.49
129.30
129.11
129.03
128.68
75.75
75.22
68.40
67.86
61.94
54.81
49.85
49.57
49.28
49.00
48.72
48.43
48.15
20.30
18.85
f1 (мд)

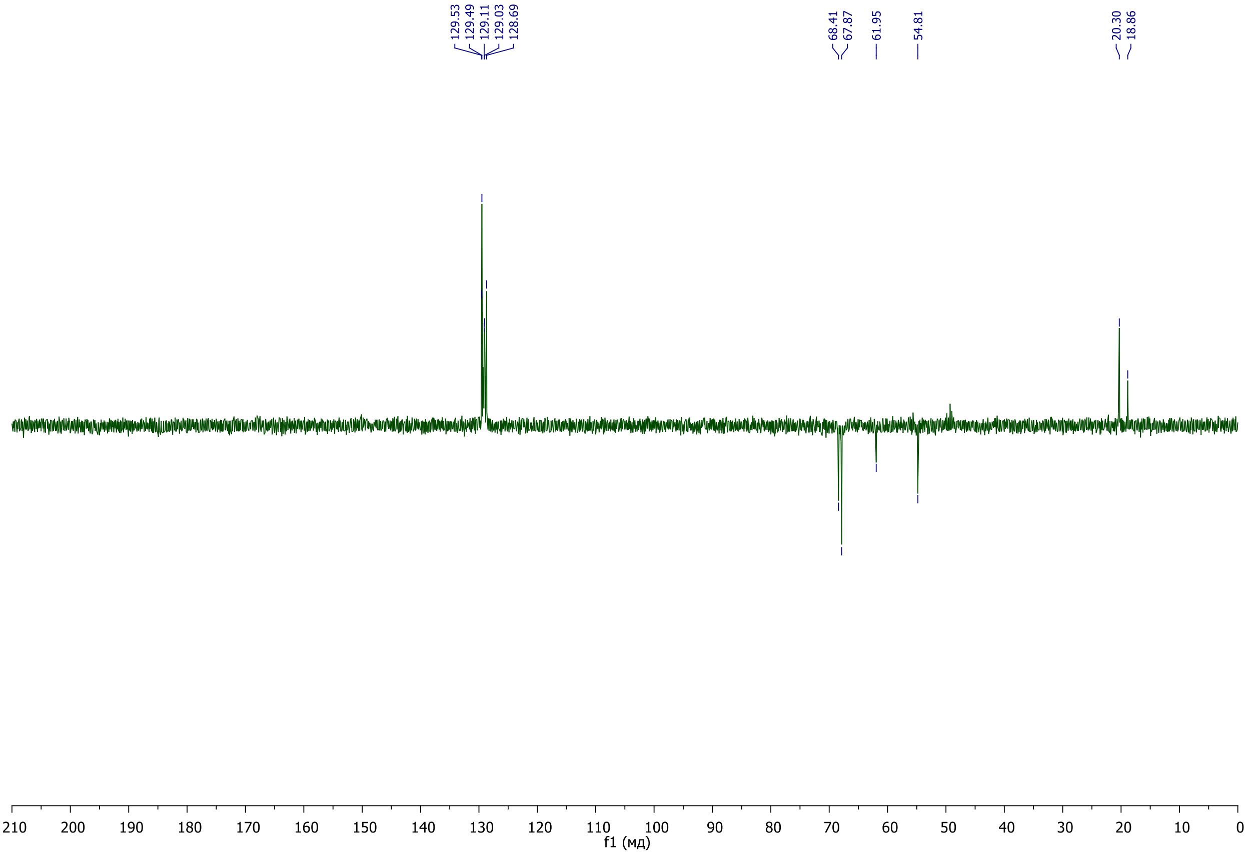
129.53
129.49
129.11
129.03
128.69
68.41
67.87
61.95
54.81
20.30
18.86
f1 (мд)

TAAD 4e

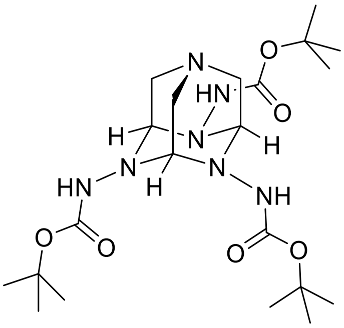

White solid, dec. 250-270 °C without melting.

^{1}H NMR (300 MHz, DMSO-d6): δ = 1.41 (s, 27 H, 3 C(C*H*$_3$)$_3$), 3.16 (s, 6 H, 3 C*H*$_2$), 3.40 (s, 3 H, 3 C*H*), 8.35 (s, 3 H, 3 N*H*).

^{13}C NMR (75 MHz, DMSO-d6): δ = 28.1 (3 C(*C*H$_3$)$_3$), 49.9 (3 *C*H$_2$), 73.2 (3 N*C*N), 78.9 (3 *C*(CH$_3$)$_3$), 154.2 (3 *C*=O).

HRMS: Calcd for $C_{21}H_{40}N_7O_6^+$ [M+H$^+$] m/z: 486.3035. Found: 486.3027.

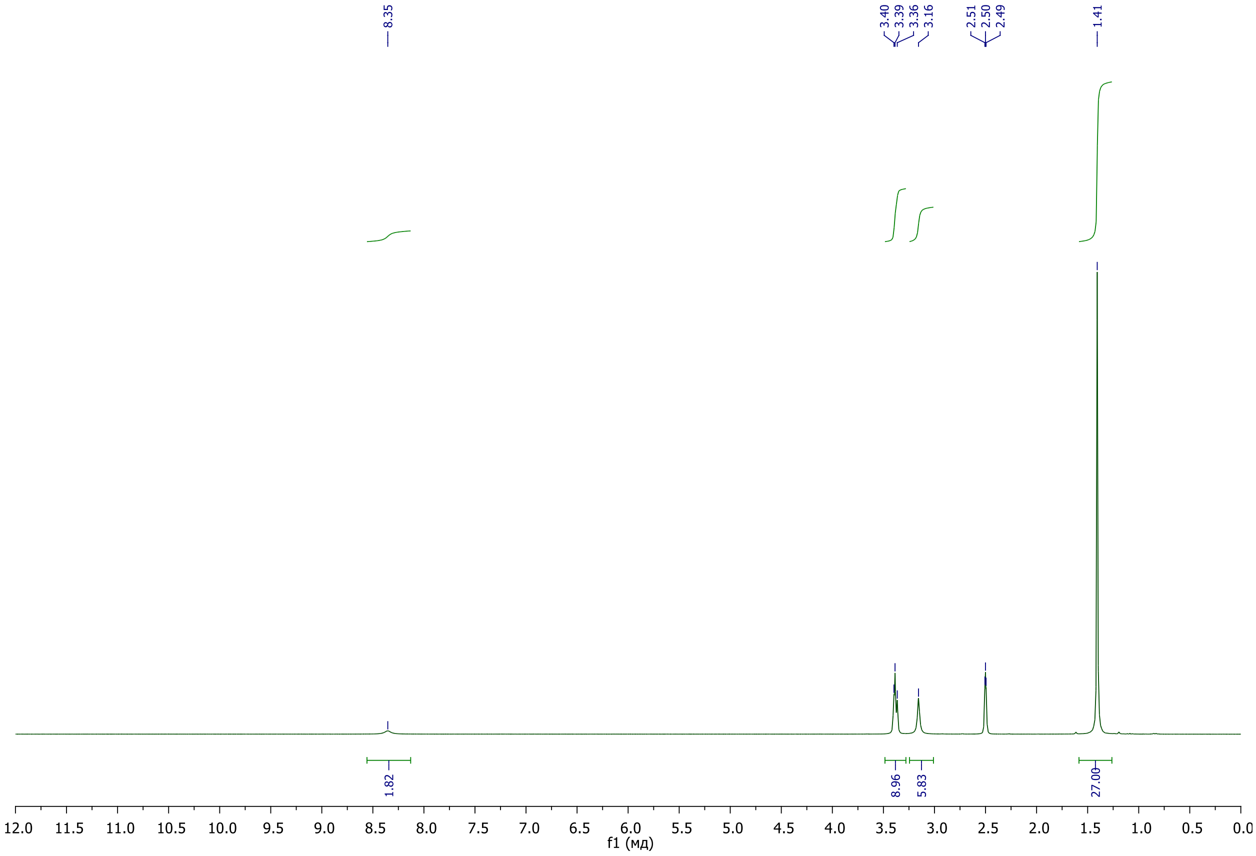

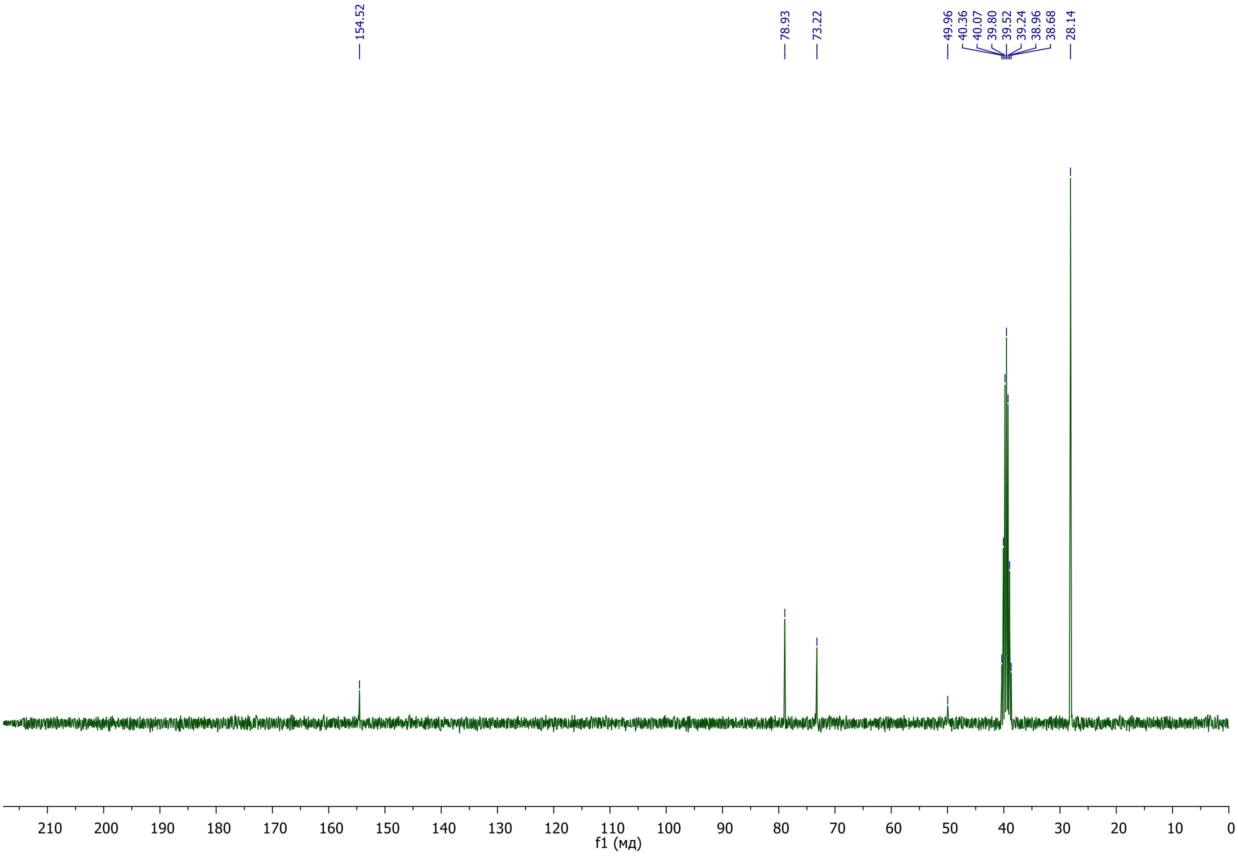
154.52
78.93
73.22
49.96
40.36
40.07
39.80
39.52
39.24
38.96
38.68
28.14
210 200 190 180 170 160 150 140 130 120 110 100 90 80 70 60 50 40 30 20 10 0
f1 (мд)

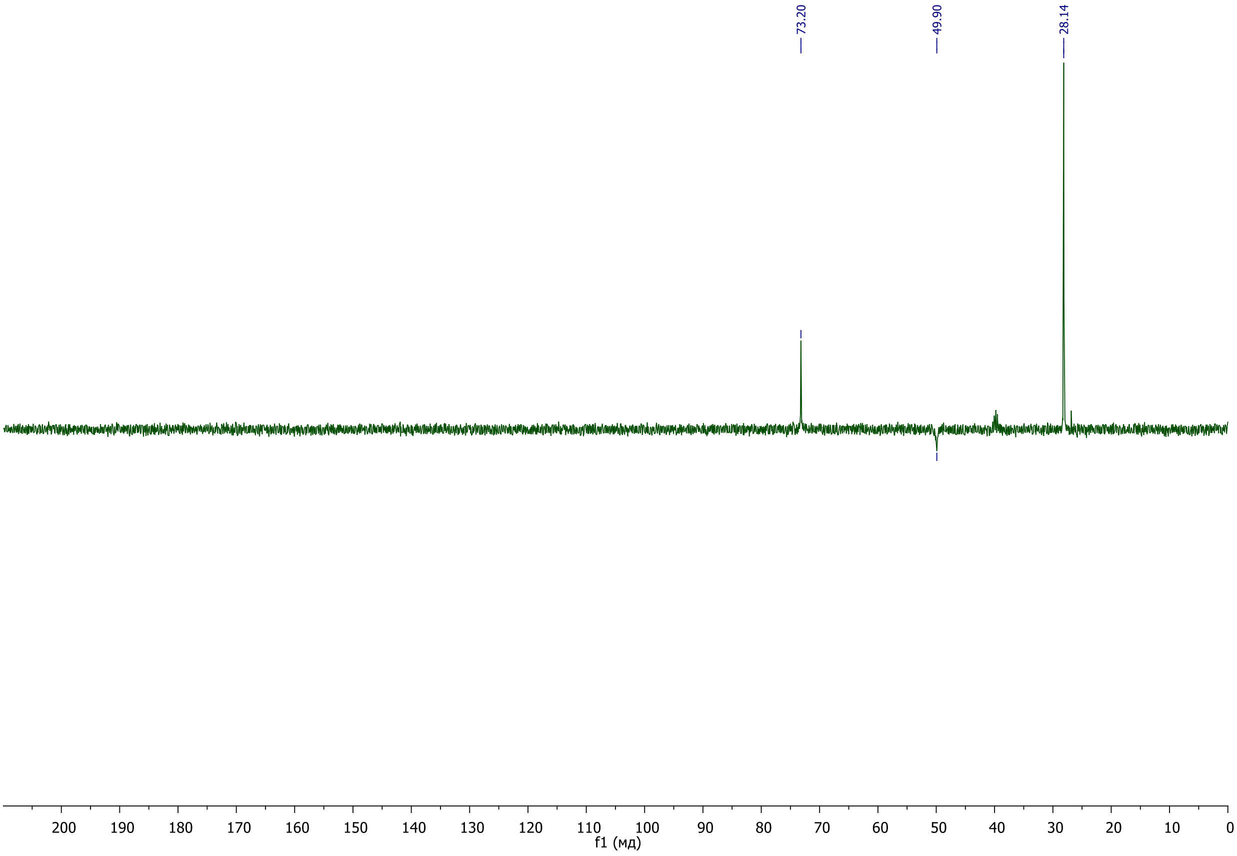
73.20
49.90
28.14
200 190 180 170 160 150 140 130 120 110 100 90 80 70 60 50 40 30 20 10 0
f1 (мд)

HSQC

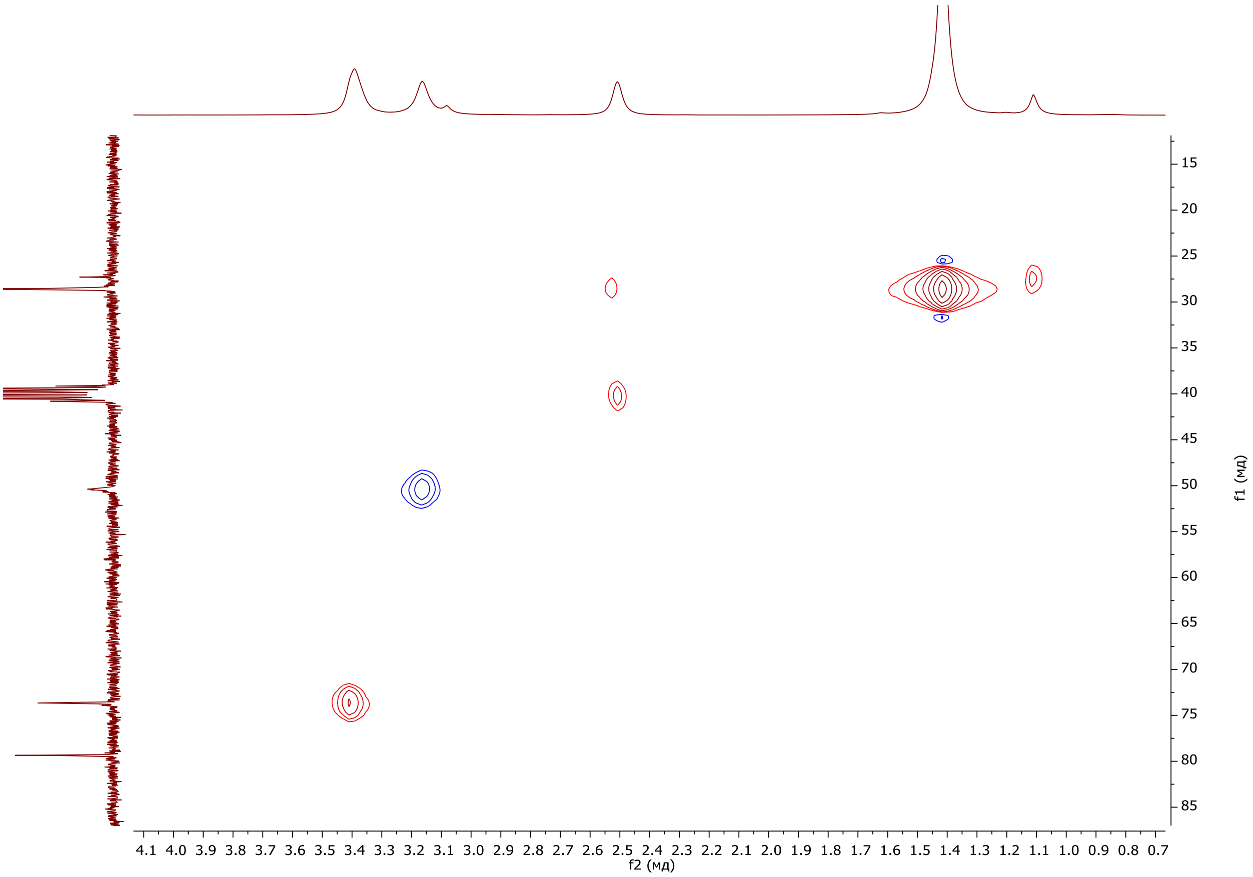

15
20
25
30
35
40
45
50
55
60
65
70
75
80
85
f1 (мд)
4.1 4.0 3.9 3.8 3.7 3.6 3.5 3.4 3.3 3.2 3.1 3.0 2.9 2.8 2.7 2.6 2.5 2.4 2.3 2.2 2.1 2.0 1.9 1.8 1.7 1.6 1.5 1.4 1.3 1.2 1.1 1.0 0.9 0.8 0.7
f2 (мд)

TAAD 6a

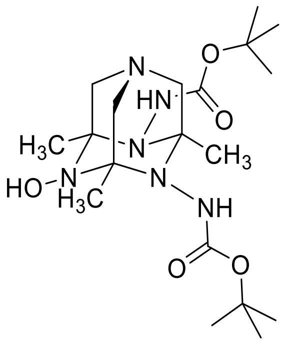

White solid, mp = 147-152 °C

^{1}H NMR (300 MHz, CD_3OD): δ = 1.01, 1.13, 1.18, 1.24 and 1.26 (5 br, 9 H, 2 CH_3 and CH_3), 1.48 and 1.52 (2 s, 18 H, 2 $C(CH_3)_3$), 2.95, 2.98, 3.2 and 3.26 (4 br, 6 H, 2 CH_2 and CH_2).

^{13}C NMR (75 MHz, CD_3OD): δ = 18.9 and 20.0 (2 CH_3 and CH_3), 28.6 and 28.7 (2 $C(CH_3)_3$), 55.0 (br, CH_2), 62.8 and 63.4 (2 CH_2), 75.0, 75.7, 76.1 and 76.3 (2 NCN and NCN), 80.5 and 80.9 (2 $C(CH_3)_3$), 158.9 and 159.5 (2 C=O).

HRMS: Calcd for $C_{19}H_{37}N_6O_5^+$ [M+H$^+$] m/z: 429.2831. Found: 429.2820.

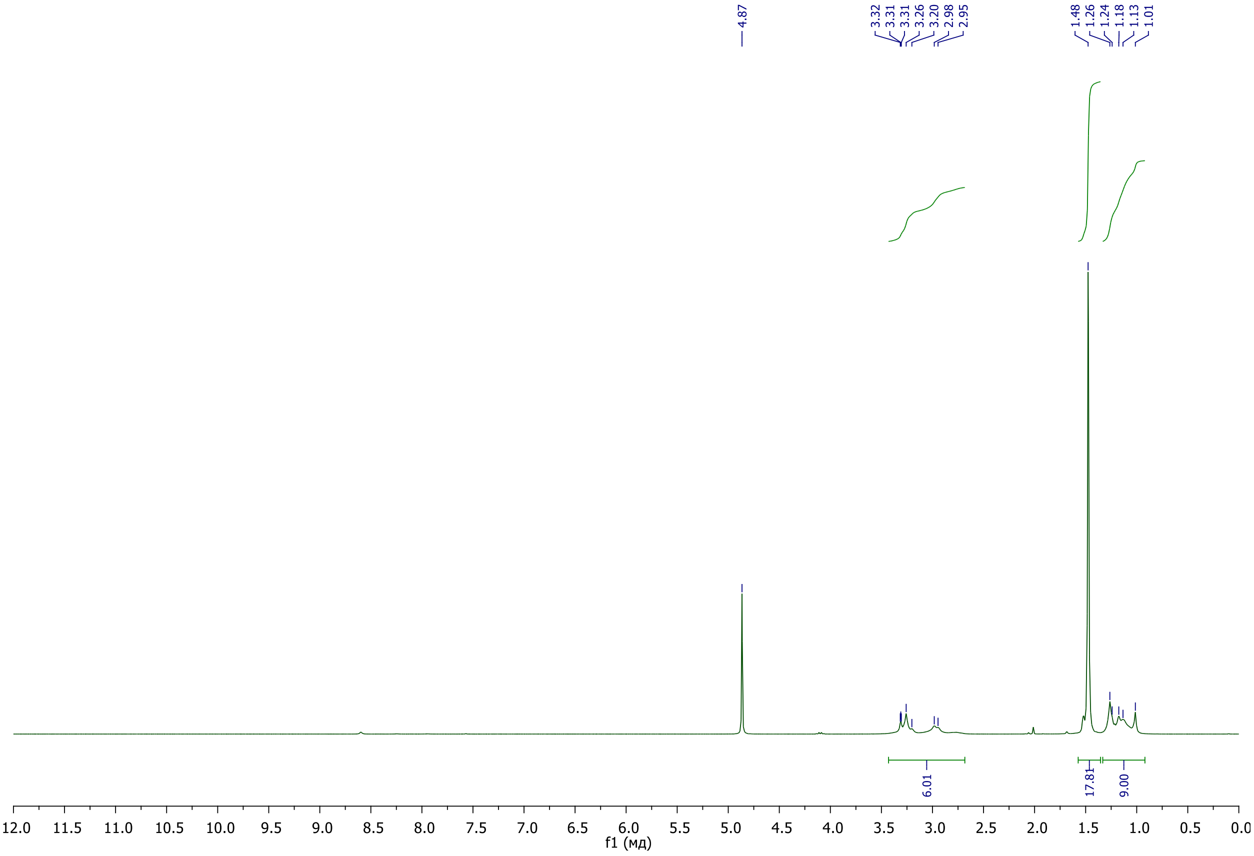

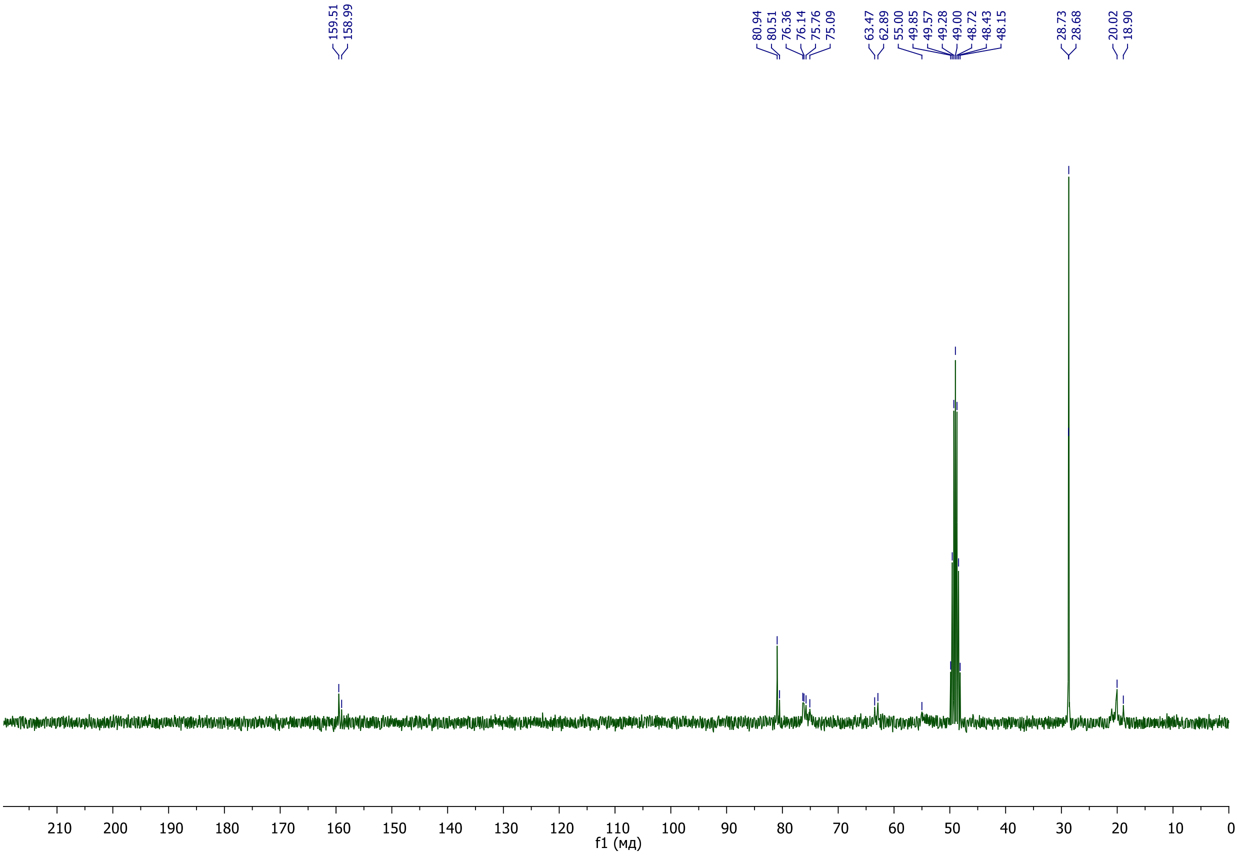
159.51
158.99
80.94
80.51
76.36
76.14
75.76
75.09
63.47
62.89
55.00
49.85
49.57
49.28
49.00
48.72
48.43
48.15
28.73
28.68
20.02
18.90
210 200 190 180 170 160 150 140 130 120 110 100 90 80 70 60 50 40 30 20 10 0
f1 (мд)

TAAD 8a

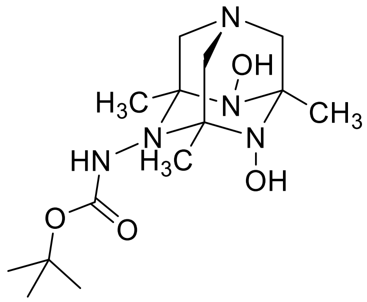

White solid, mp = 194-196 °C

^{1}H NMR (300 MHz, DMSO-d6): δ = 0.9-1.2 (s br, 6 H, 2 C*H*$_3$), 1.12 (s, 3 H, C*H*$_3$), 1.37 (s, 9 H, C(C*H*$_3$)$_3$), 2.5-3.3 (br, 6 H, 2 C*H*$_2$ and C*H*$_2$), 7.66 and 7.90 (2 br, 3 H, 2 O*H* and N*H*).

^{1}H NMR (300 MHz, DMSO-d6, 330K): δ = 1.08 (s br, 6 H, 2 C*H*$_3$), 1.11 (s, 3 H, C*H*$_3$), 1.38 (s, 9 H, C(C*H*$_3$)$_3$), 2.7-3.0 and 3.0-3.3 (2 br, 6 H, 2 C*H*$_2$ and C*H*$_2$), 7.59 and 7.86 (2 br, 3 H, 2 O*H* and N*H*).

^{13}C NMR (75 MHz, DMSO-d6, 330K): δ = 19.6 and 20.8 (2 *C*H$_3$ and *C*H$_3$), 27.9 (C(*C*H$_3$)$_3$), 50-60 (br, 2 *C*H$_2$ and *C*H$_2$), 73.0, 74.3 and 77.8 (2 N*C*N and N*C*N). Signals of *C*(CH$_3$)$_3$ and *C*=O was not observed at 1200 scans.

HRMS: Calcd for $C_{14}H_{28}N_5O_4^+$ [M+H$^+$] m/z: 330.2136. Found: 330.2137.

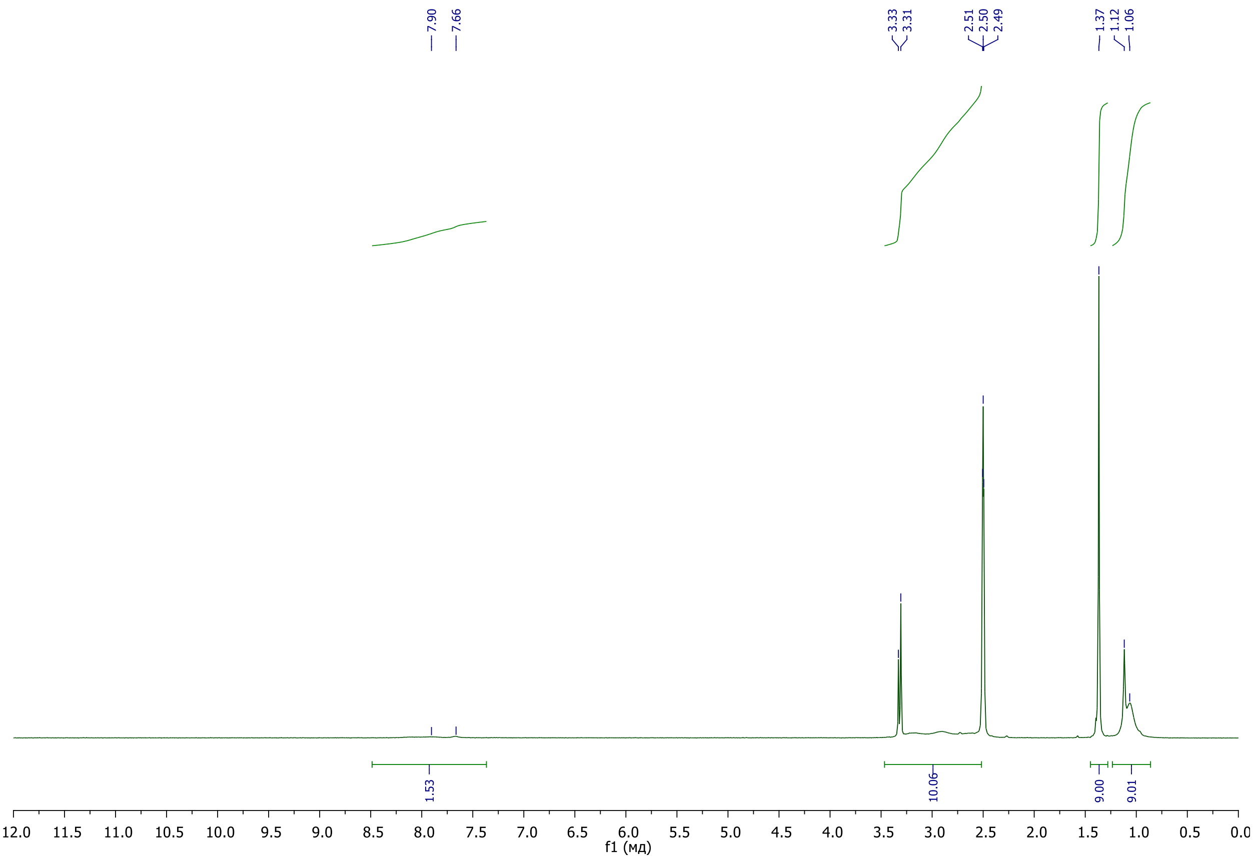

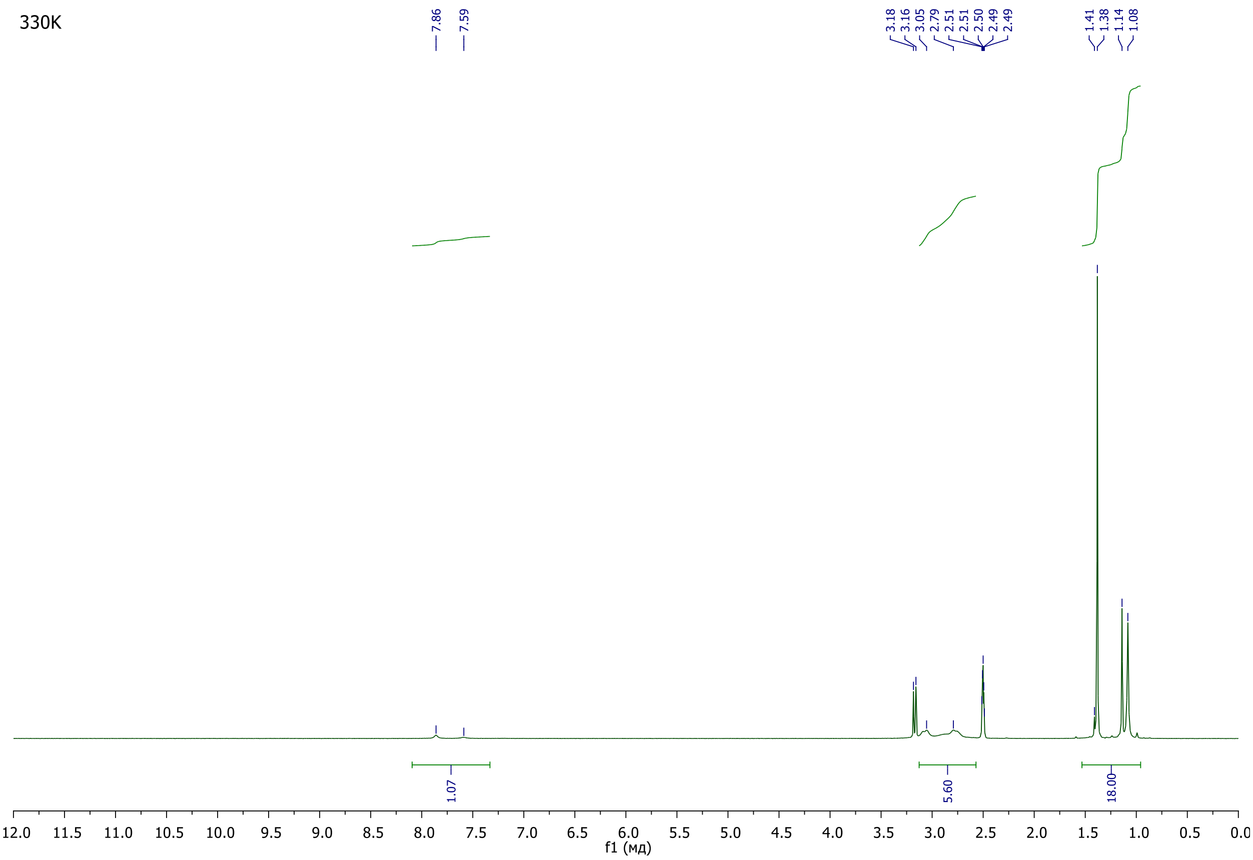
330K
7.86
7.59
3.18
3.16
3.05
2.79
2.51
2.50
2.49
2.49
1.41
1.38
1.14
1.08
1.07
5.60
18.00
f1 (мд)

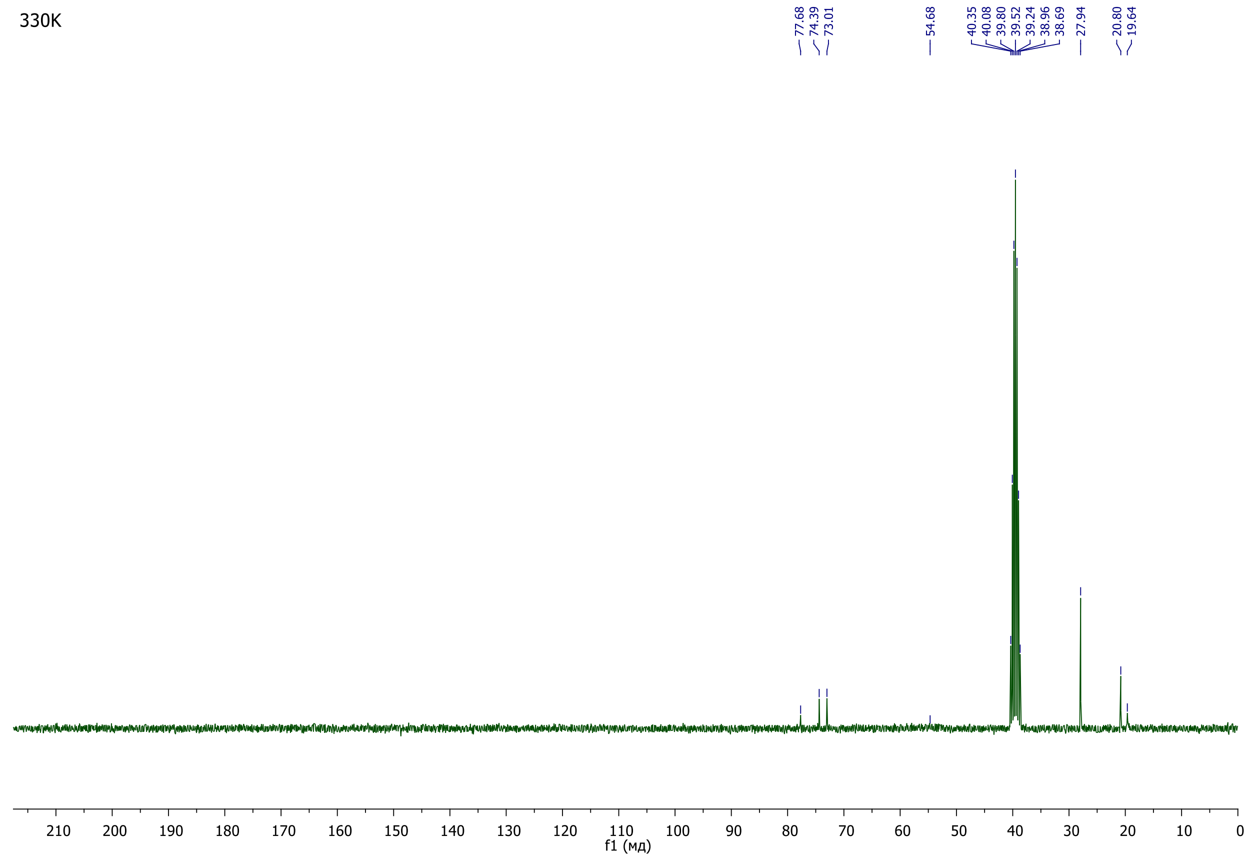
330K
77.68
74.39
73.01
54.68
40.35
40.08
39.80
39.52
39.24
38.96
38.69
27.94
20.80
19.64
f1 (мд)

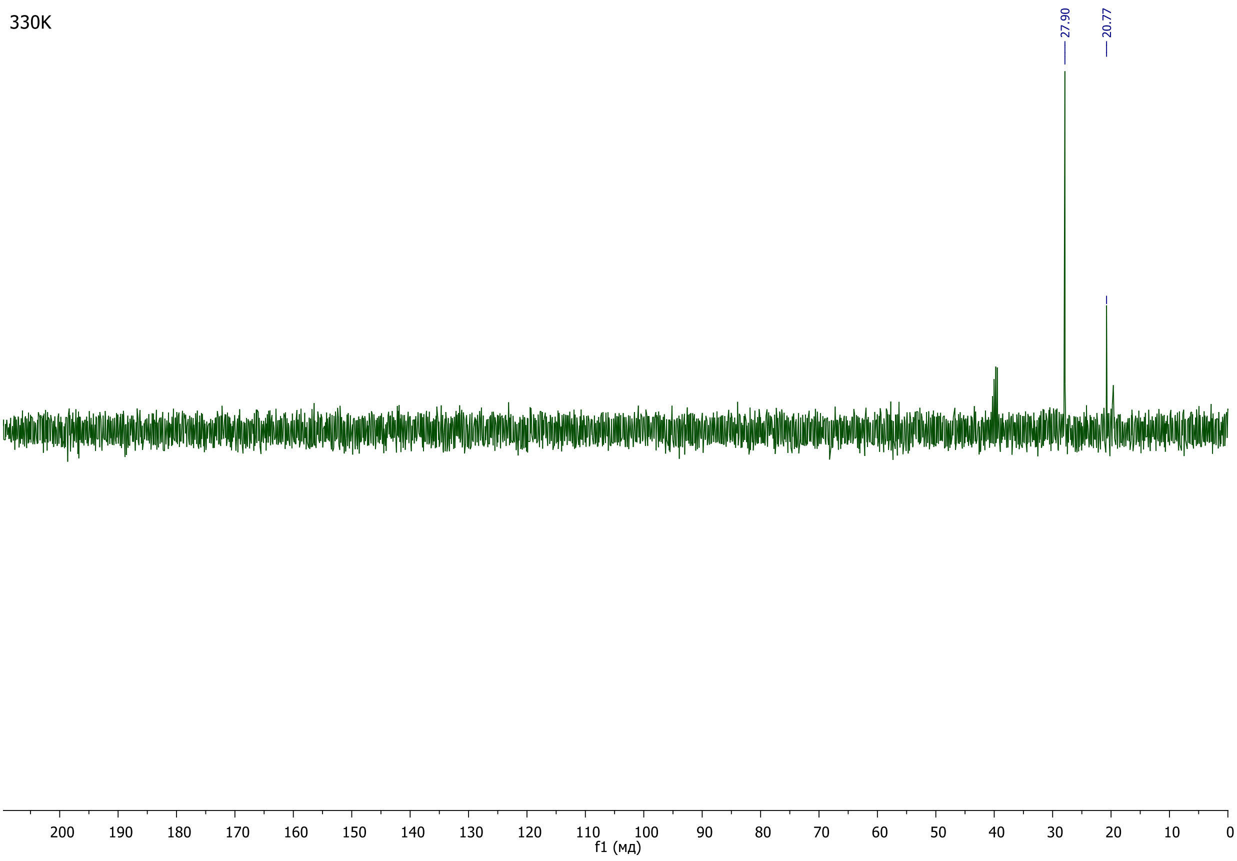

330K
27.90
20.77
200 190 180 170 160 150 140 130 120 110 100 90 80 70 60 50 40 30 20 10 0
f1 (мд)

TAAD 8b

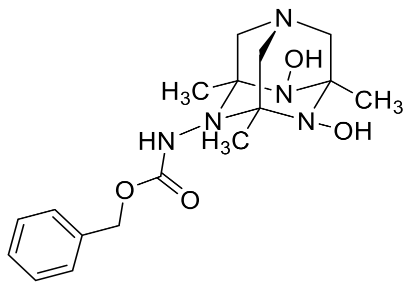

Pale yellow amorphous solid, starts softening at 120 °C, mp = 145-151 °C

^{1}H NMR (300 MHz, DMSO-d6, 330K): δ = 1.09 (s, 6 H, 2 C*H$_3$*), 1.15 (s, 3 H, C*H$_3$*), 2.2-3.0 and 3.0-3.2 (2 br, 6 H, 2 C*H$_2$* and C*H$_2$*), 5.06 (s, 2 H, PhC*H$_2$*), 7.2-7.4 (m, 5 H, *Ph*), 7.58 (br, 1 H, N*H*), 7.94 (br, 2 H, 2 O*H*).

^{13}C NMR (75 MHz, DMSO-d6, 330K): δ = 19.7 (C*H$_3$*), 20.8 (2 C*H$_3$*), 50-60 (br, 2 C*H$_2$* and C*H$_2$*), 65.1 (PhC*H$_2$*), 73.1 and 74.4 (2 N*C*N and N*C*N), 127.1, 127.5 and 128.1 (*o,m,p-Ph*), 136.9 (*i-Ph*), 155.3 (*C*=O).

HRMS: Calcd for $C_{17}H_{26}N_5O_4^+$ [M+H$^+$] m/z: 364.1979. Found: 364.1983.

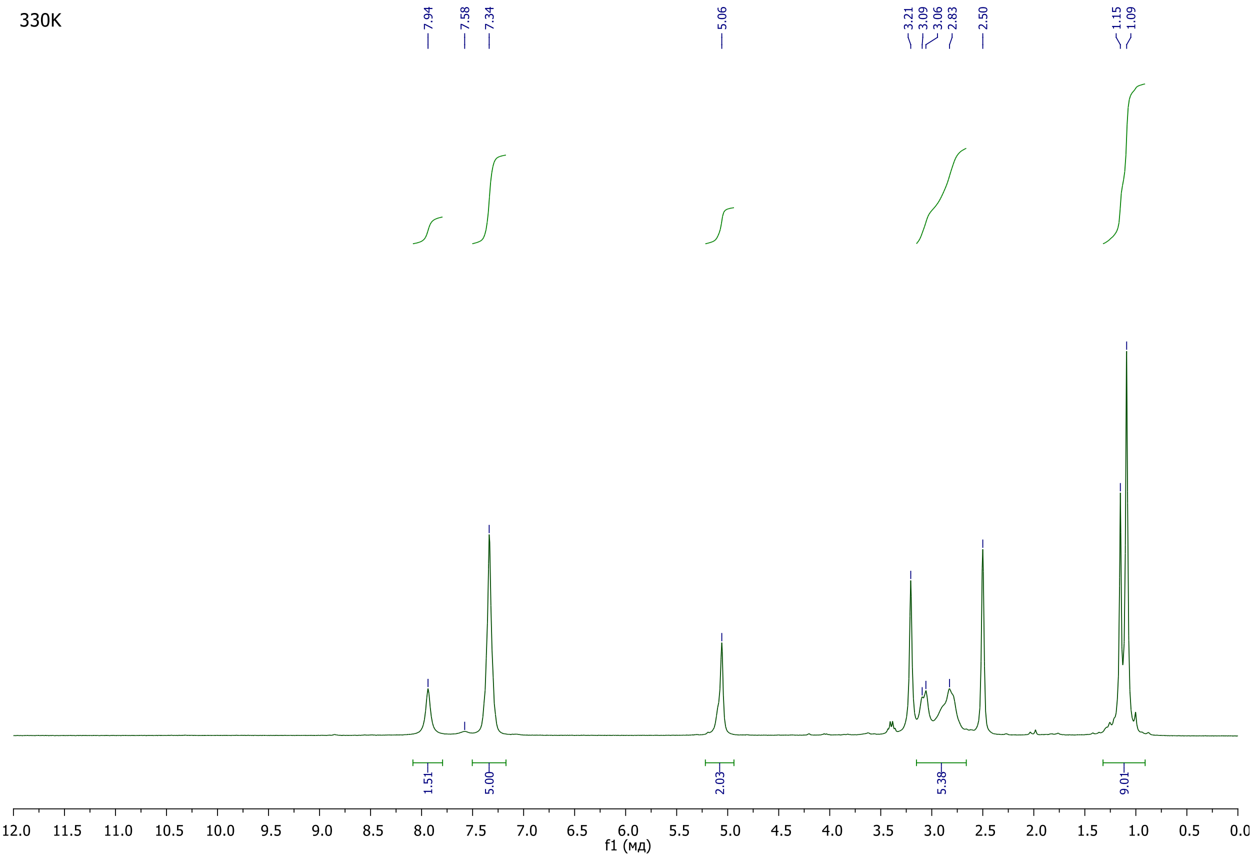

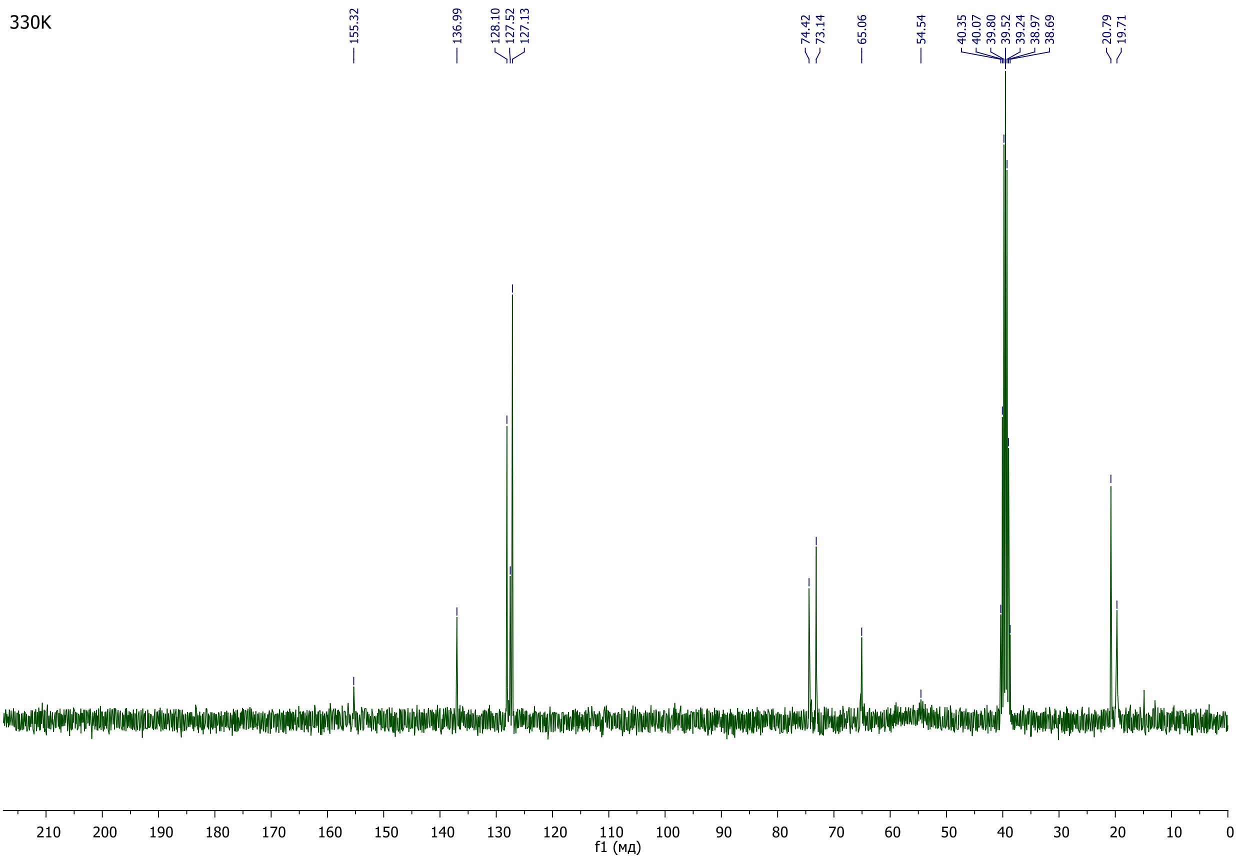
330K
155.32
136.99
128.10
127.52
127.13
74.42
73.14
65.06
54.54
40.35
40.07
39.80
39.52
39.24
38.97
38.69
20.79
19.71
210 200 190 180 170 160 150 140 130 120 110 100 90 80 70 60 50 40 30 20 10 0
f1 (мд)

TAAD salt 4a·HCl

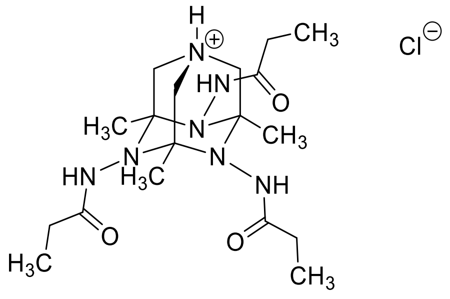

Pale yellow solid, mp = 122-129 °C with dec.

^{1}H NMR (300 MHz, D_2O): δ = 1.19 (m, 18 H, 3 C*H₃* and 3 CH_2CH_3), 2.36 (m, 6 H, 3 CH_2CH_3), 3.72 and 3.80 (2 d, *J* = 13.2 Hz, 4 H, 2 CH_2), 3.88 (s, 2 H, CH_2).

^{13}C NMR (75 MHz, D_2O): δ = 9.3 and 9.7 (3 CH_2CH_3), 17.5 and 19.2 (3 CH_3), 27.2 (3 CH_2CH_3), 51.1 and 57.6 (3 CH_2), 73.6 and 74.2 (2 N*C*N and N*C*N), 178.1 and 179.1 (3 C=O).

HRMS: Calcd for $C_{18}H_{34}N_7O_3^+$ [M-Cl$^-$] m/z: 396.2718. Found: 396.2720.

For $C_{18}H_{34}ClN_7O_3$ calcd: Cl 8.21%. Found: 8.05%; 8.12%.

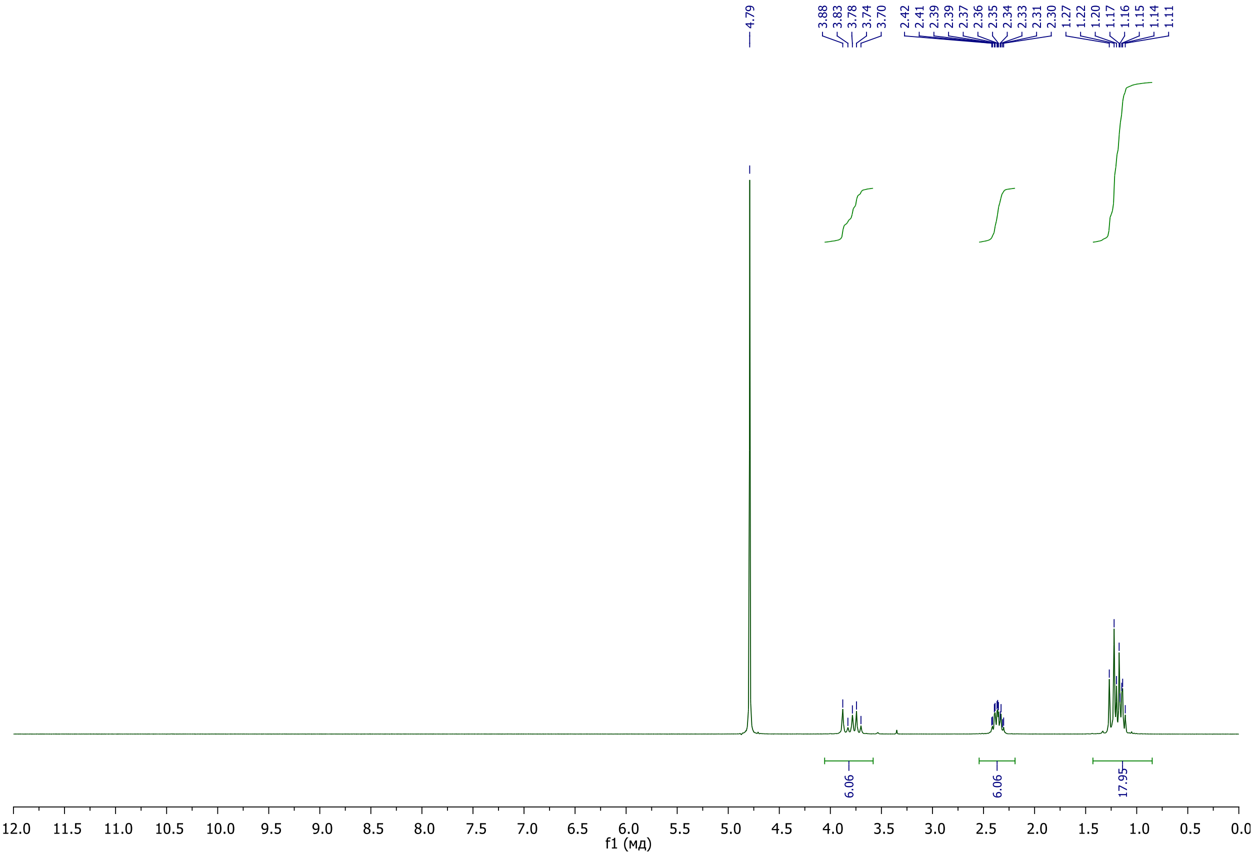

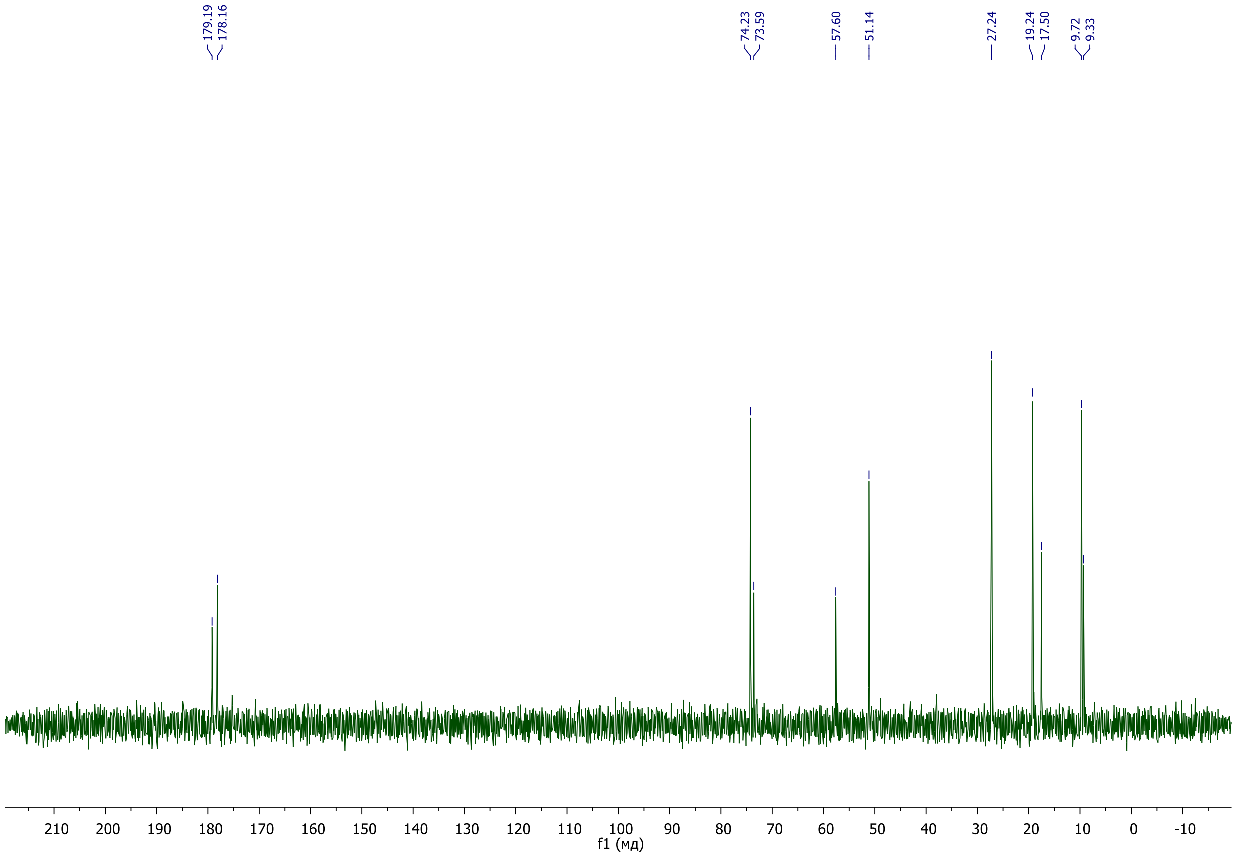
179.19
178.16
74.23
73.59
57.60
51.14
27.24
19.24
17.50
9.72
9.33
210 200 190 180 170 160 150 140 130 120 110 100 90 80 70 60 50 40 30 20 10 0 -10
f1 (мд)

TAAD salt 4c·HCl

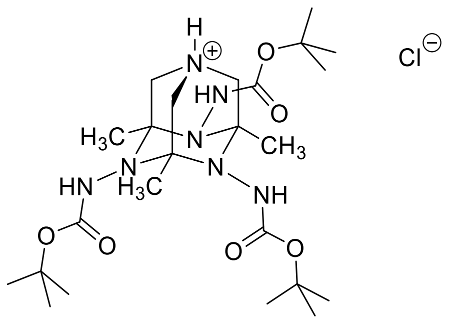

White solid, mp = 149-152 °C

^{1}H NMR (300 MHz, D_2O): δ = 1.24 and 1.34 (2 s, 9 H, 3 C*H*$_3$), 1.47 and 1.48 (2 s, 27 H, 3 C(C*H*$_3$)$_3$), 3.68 and 3.82 (2 s, 6 H, 3 C*H*$_2$).

^{13}C NMR (75 MHz, D_2O): δ = 17.7 and 19.0 (3 *C*H$_3$), 27.5 (3 C(*C*H$_3$)$_3$), 51.3 and 58.0 (3 *C*H$_2$), 73.4 and 74.7 (3 N*C*N), 82.4 and 82.7 (3 *C*(CH$_3$)$_3$), 158.3 (3 *C*=O).

HRMS: Calcd for $C_{24}H_{46}N_7O_6^+$ [M-Cl$^-$] m/z: 528.3504. Found: 528.3500.

For $C_{24}H_{46}ClN_7O_6$ calcd: Cl 6.28%. Found: 6.19%; 6.02%.

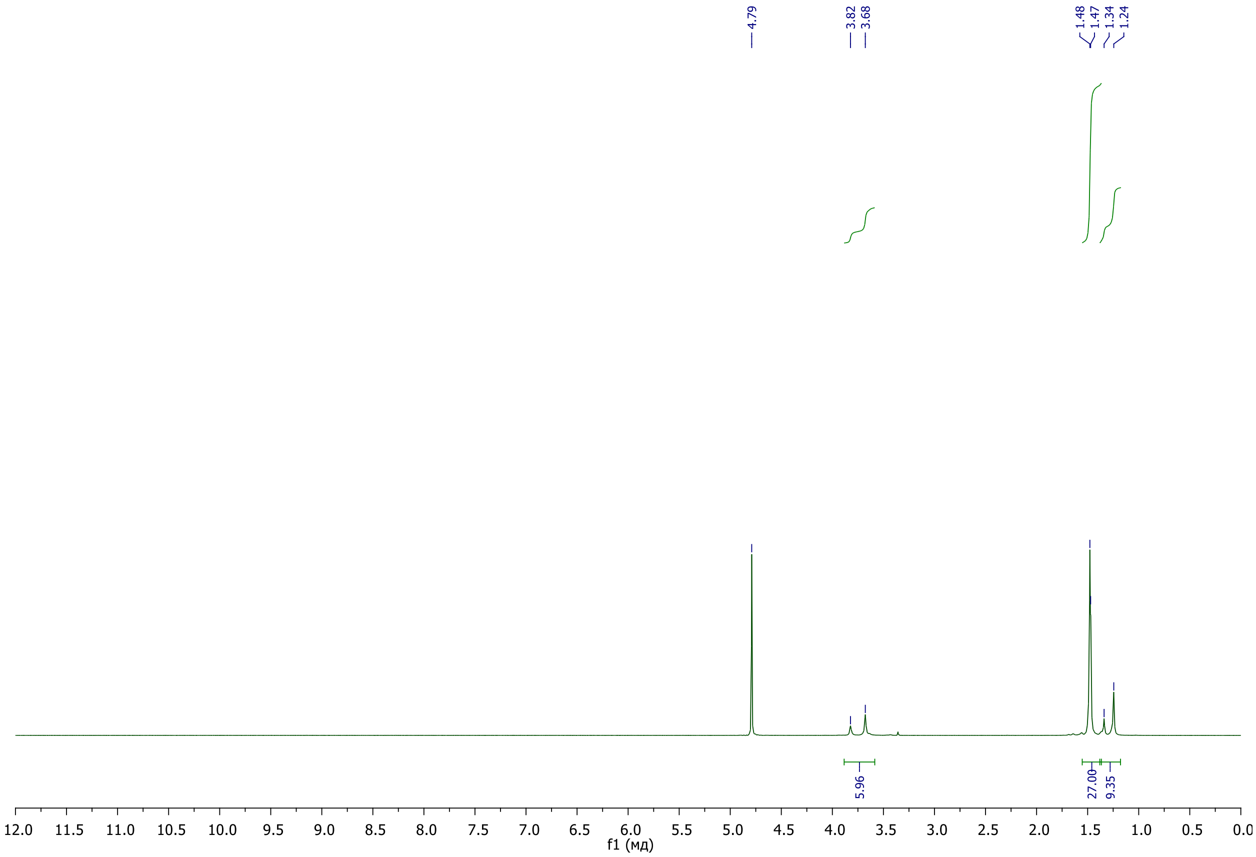

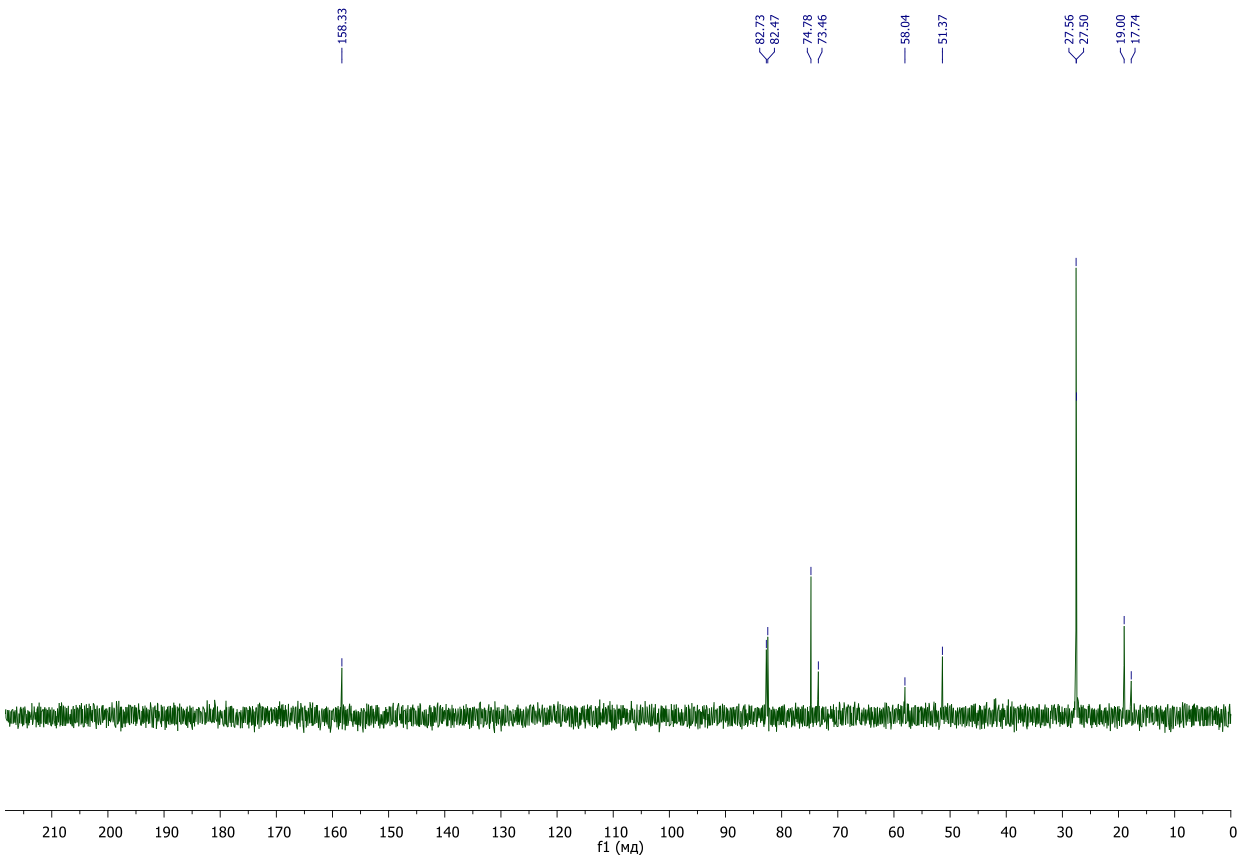
158.33
82.73
82.47
74.78
73.46
58.04
51.37
27.56
27.50
19.00
17.74
210 200 190 180 170 160 150 140 130 120 110 100 90 80 70 60 50 40 30 20 10 0
f1 (мд)

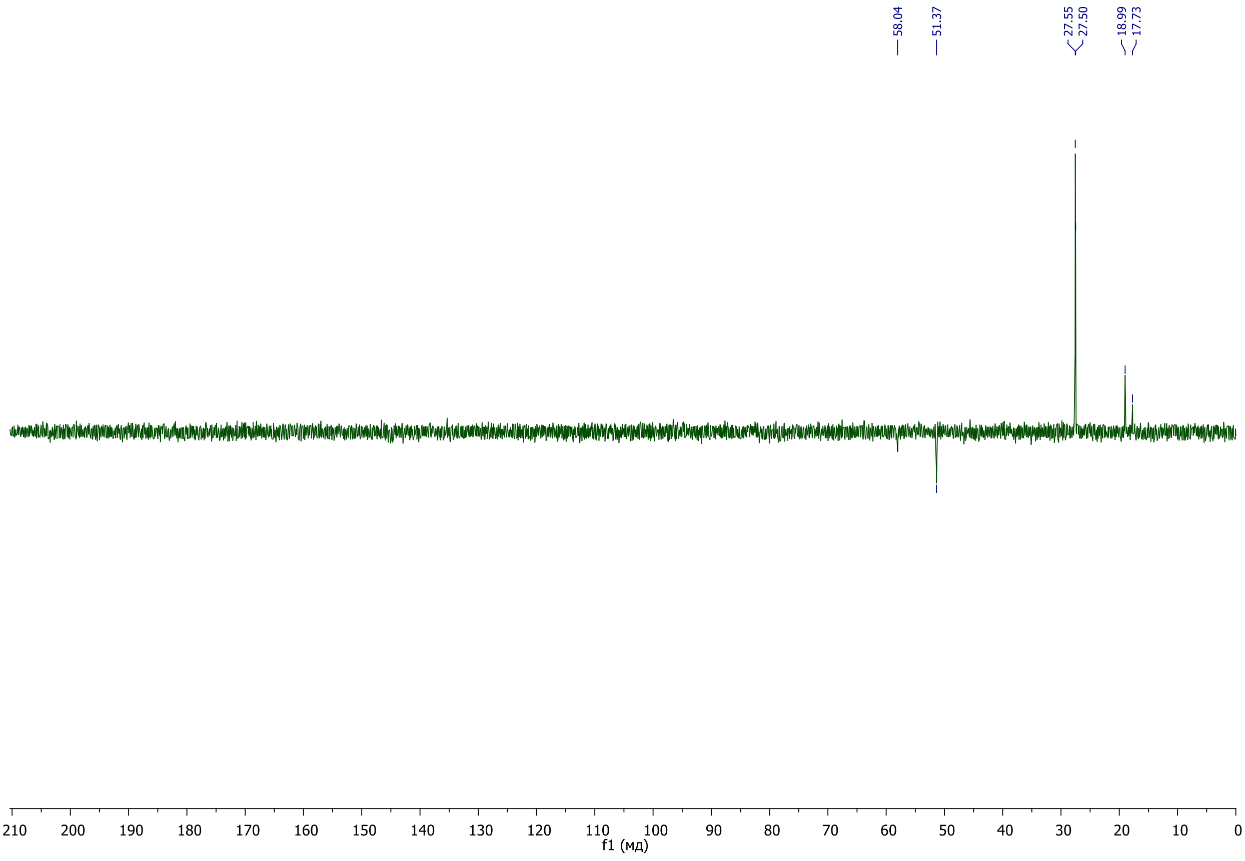
58.04
51.37
27.55
27.50
18.99
17.73
210 200 190 180 170 160 150 140 130 120 110 100 90 80 70 60 50 40 30 20 10 0
f1 (мд)

TAAD quaternary chloride salt Bn-4c·2CH_3OH

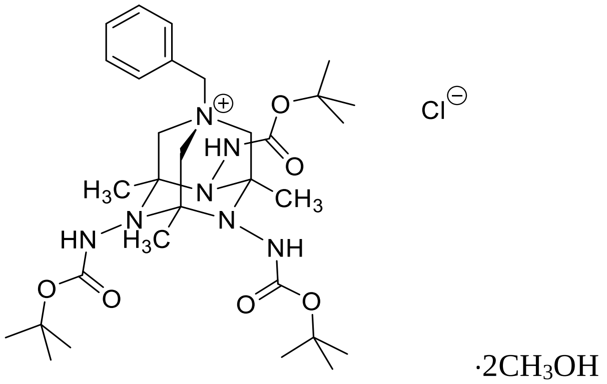

White solid, mp = 192-196 °C (with dec.)

^{1}H NMR (300 MHz, CD_3OD): δ = 1.18 and 1.23 (2 s, 9 H, 3 CH_3), 1.48 and 1.50 (2 s, 27 H, 3 C(CH_3)$_3$), 3.35 (s, 6 H, 2 CH_3OH), 3.68 and 4.02 (2 d, J = 12.3 Hz, 4 H, 2 CH_2), 3.77 (s, 2 H, CH_2), 4.62 (s, 2 H, PhCH_2), 7.59 (m, 5 H, *Ph*), 8.48 and 9.13 (2 s, 3 H, 3 N*H*).

^{13}C NMR (75 MHz, CD_3OD): δ = 18.6 and 19.8 (3 *C*H_3), 28.5 (s, 3 C(*C*H_3)$_3$), 49.8 (2 *C*H_3OH), 59.4 and 64.8 (3 CH_2), 71.1 (PhCH_2), 76.9 and 74.6 (3 N*C*N), 81.9 and 83.0 (3 *C*(CH_3)$_3$), 126.3 (*i-Ph*), 130.5, 132.2 and 134.4 (*o*,*m*,*p-Ph*), 159.0 and 159.7 (3 *C*=O).

For $C_{31}H_{52}ClN_7O_6$•2CH_3OH calcd: Cl 4.94%. Found: 4.85%; 4.92%.

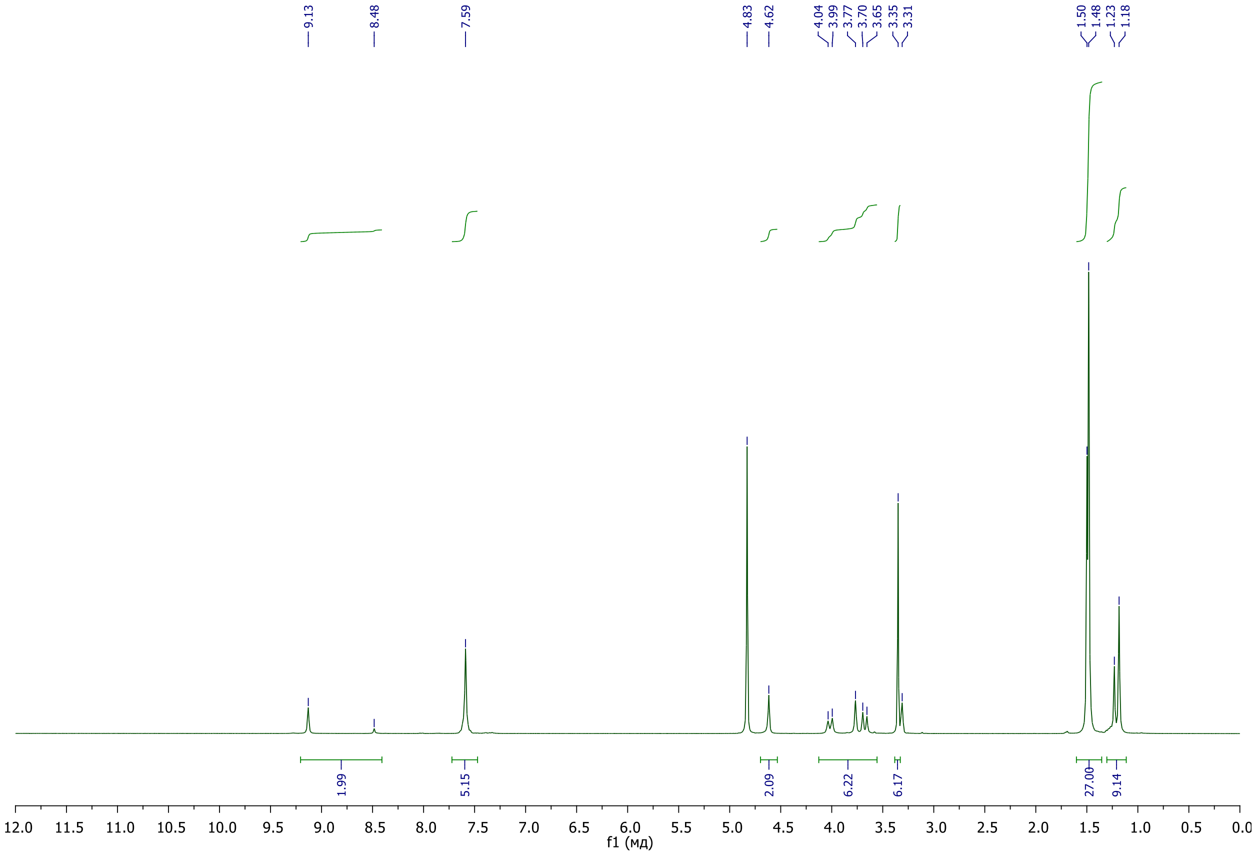

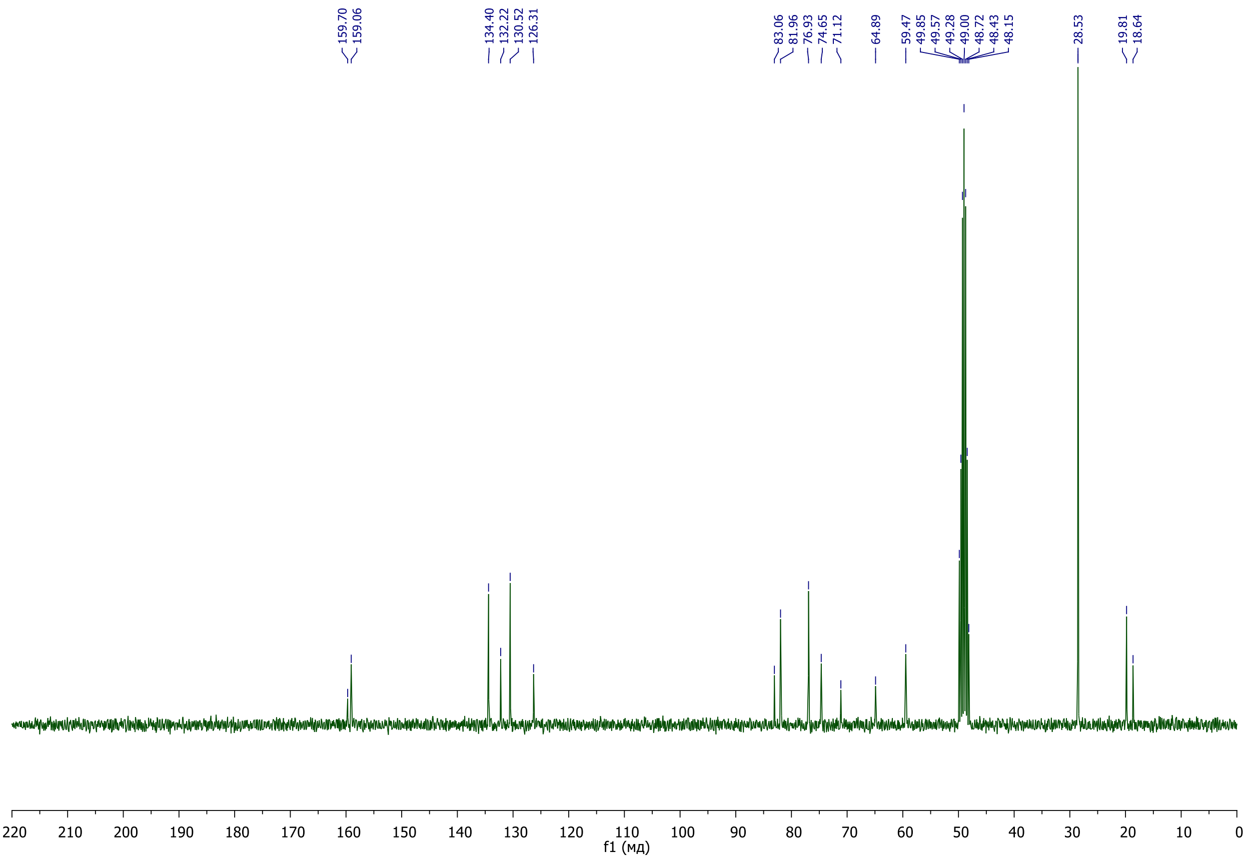
159.70
159.06
134.40
132.22
130.52
126.31
83.06
81.96
76.93
74.65
71.12
64.89
59.47
49.85
49.57
49.28
49.00
48.72
48.43
48.15
28.53
19.81
18.64
220 210 200 190 180 170 160 150 140 130 120 110 100 90 80 70 60 50 40 30 20 10 0
f1 (мд)

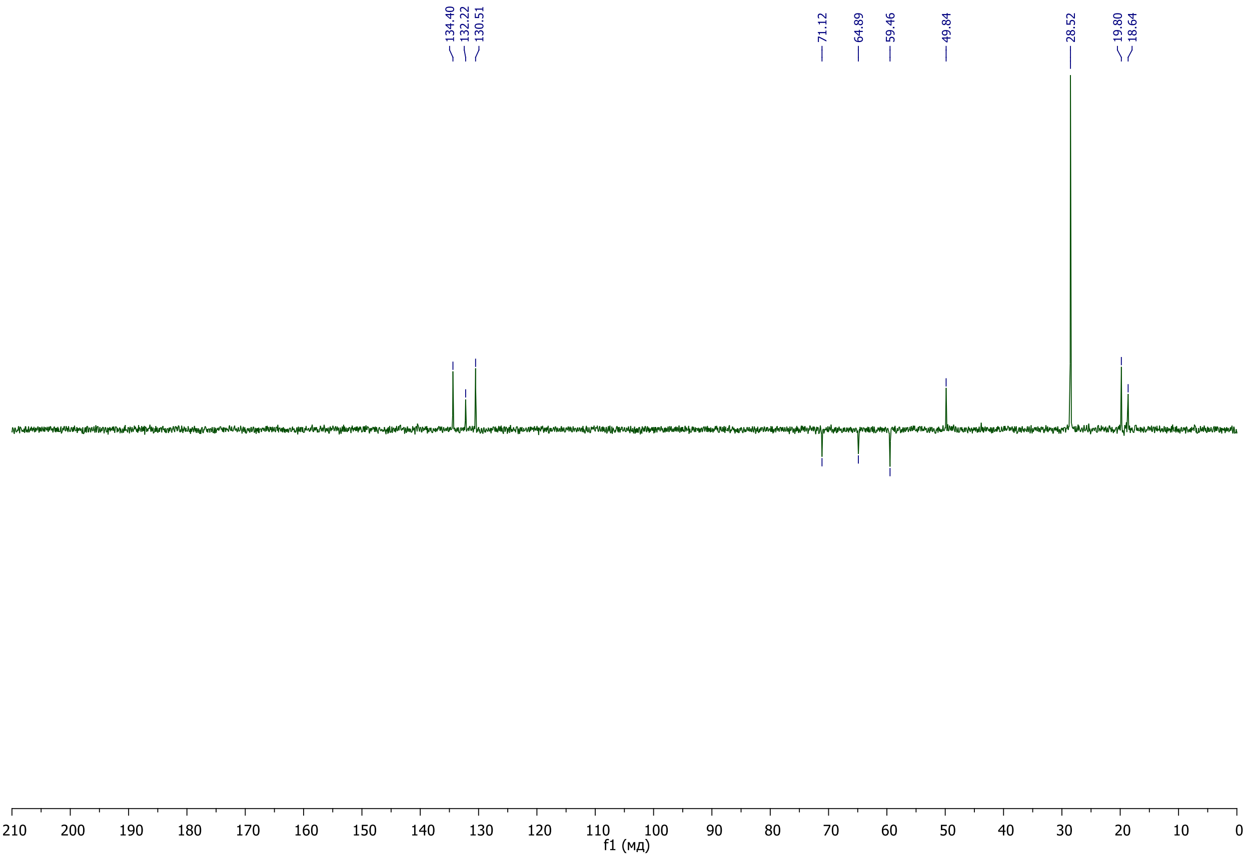
134.40
132.22
130.51
71.12
64.89
59.46
49.84
28.52
19.80
18.64
210 200 190 180 170 160 150 140 130 120 110 100 90 80 70 60 50 40 30 20 10 0
f1 (мд)

TAAD quaternary chloride salt Bn-4c

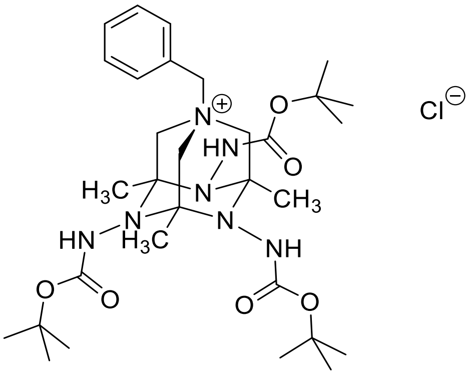

White solid, 192-196 °C (with dec.)

^{1}H NMR (300 MHz, CD_3OD): δ = 1.18 and 1.23 (2 s, 9 H, 3 C*H*$_3$), 1.48 and 1.50 (2 s, 27 H, 3 C(C*H*$_3$)$_3$), 3.68 and 3.95 (2 d, *J* = 12.3 Hz, 4 H, 2 C*H*$_2$), 3.77 (s, 2 H, C*H*$_2$), 4.62 (s, 2 H, PhC*H*$_2$), 7.58 (m, 5 H, *Ph*), 9.12 (s, 3 H, 3 N*H*).

^{13}C NMR (75 MHz, CD_3OD): δ = 18.6 and 19.8 (3 *C*H$_3$), 28.5 (s, 3 C(*C*H$_3$)$_3$), 59.4 and 64.9 (3 C*H*$_2$), 71.1 (PhC*H*$_2$), 74.6 and 76.9 (3 N*C*N), 81.9 and 83.0 (3 *C*(CH$_3$)$_3$), 126.3 (*i-Ph*), 130.5, 132.2 and 134.4 (*o,m,p-Ph*), 159.0 and 159.7 (3 *C*=O).

HRMS: Calcd for $C_{31}H_{52}N_7O_6^+$ [M-Cl$^-$] m/z: 618.3974. Found: 618.3957.

For $C_{31}H_{52}ClN_7O_6$ calcd: Cl 5.42%. Found: 5.55%; 5.63%.

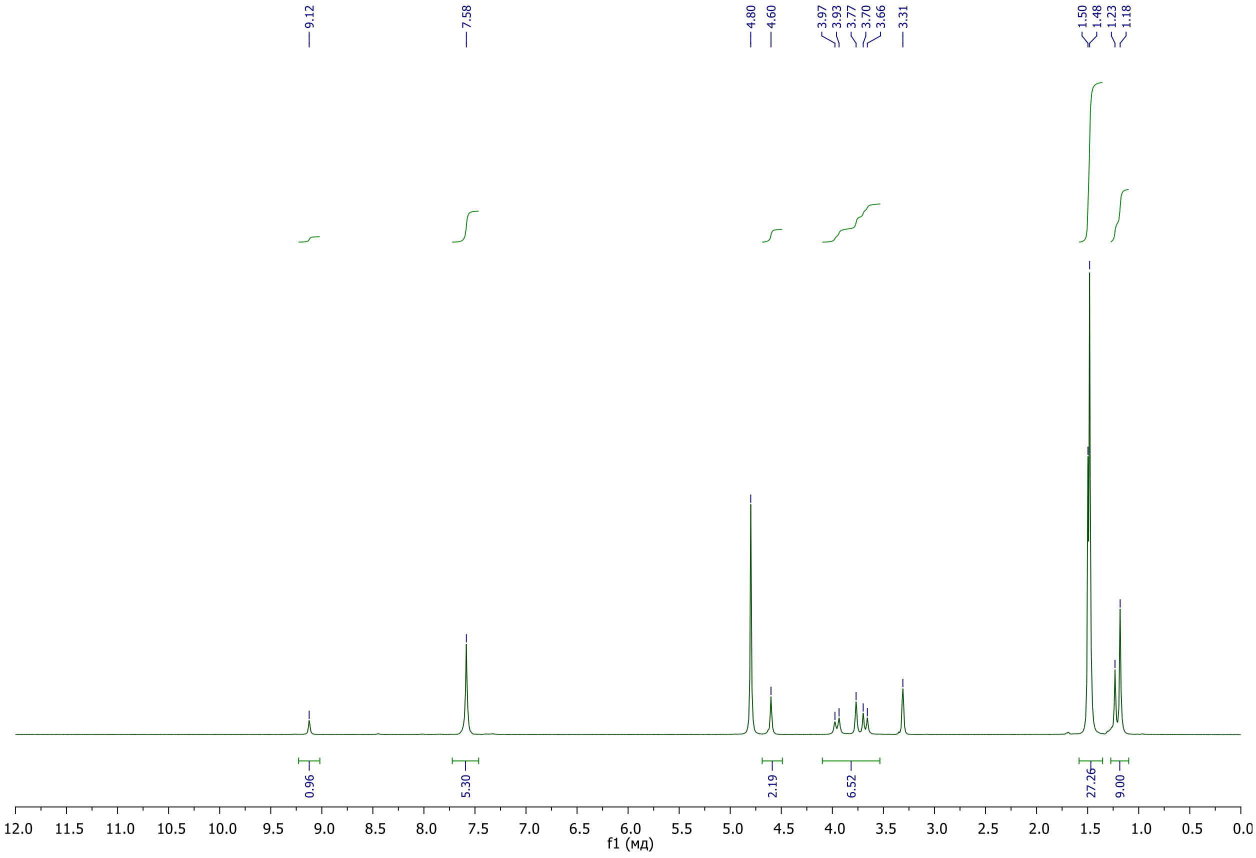

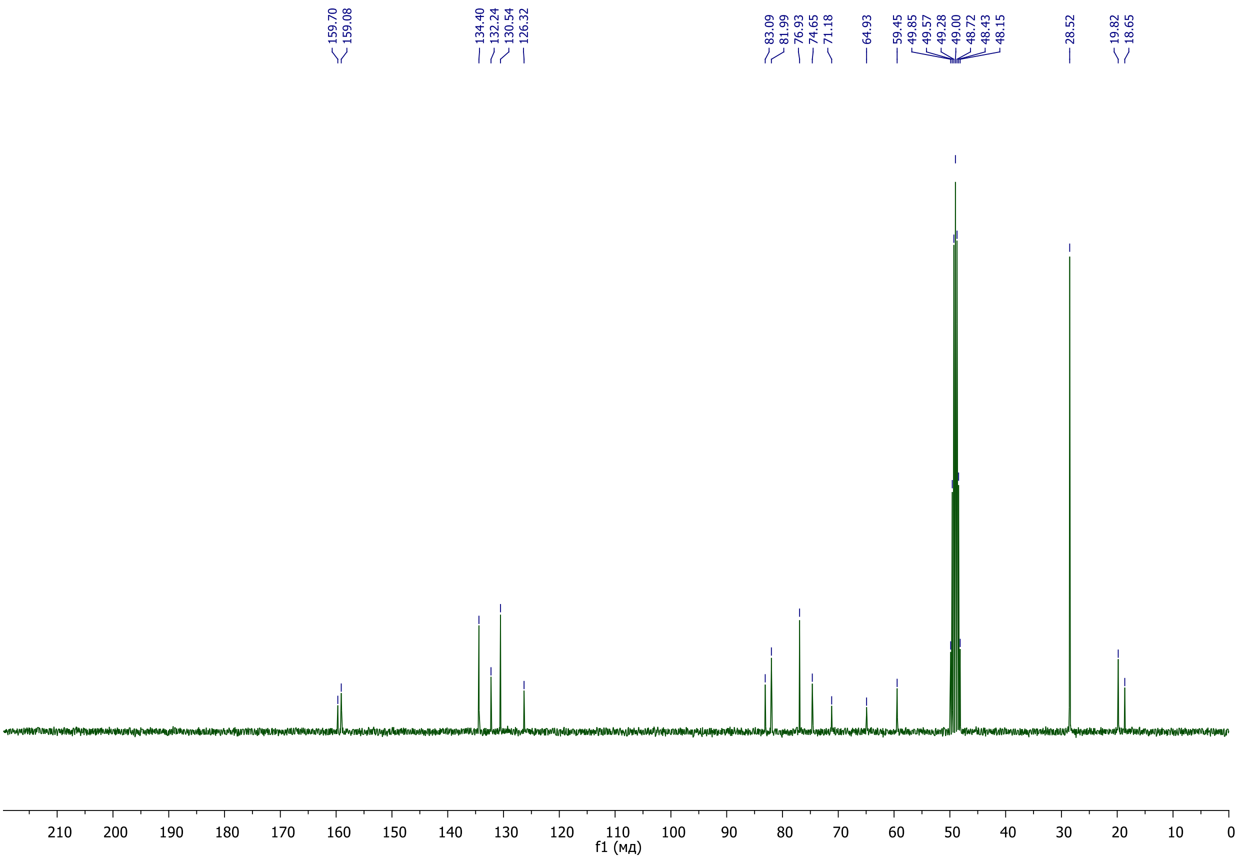
159.70
159.08
134.40
132.24
130.54
126.32
83.09
81.99
76.93
74.65
71.18
64.93
59.45
49.85
49.57
49.28
49.00
48.72
48.43
48.15
28.52
19.82
18.65
210 200 190 180 170 160 150 140 130 120 110 100 90 80 70 60 50 40 30 20 10 0
f1 (мд)

TAAD quaternary bromide salt Bn-4c·2CH3OH

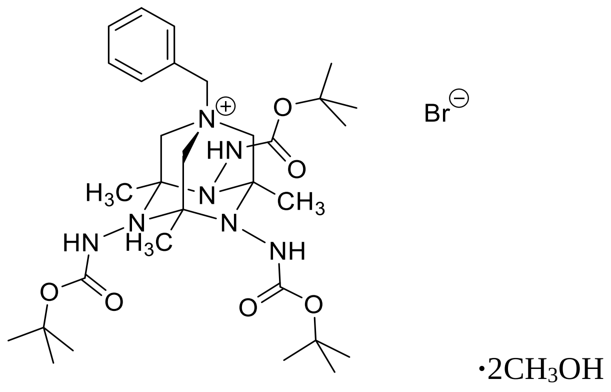

White solid, 177-182 °C with dec.

^{1}H NMR (300 MHz, CD_3OD): δ = 1.18 and 1.23 (2 s, 9 H, 3 CH_3), 1.48 and 1.50 (2 s, 27 H, 3 C(CH_3)$_3$), 3.35 (2 CH_3OH), 3.68 and 4.03 (2 d, *J* = 12.3 Hz, 4 H, 2 CH_2), 3.77 (s, 2 H, CH_2), 4.63 (s, 2 H, PhCH_2), 7.59 (m, 5 H, *Ph*), 8.37 and 9.13 (s, 3 H, 3 N*H*).

^{13}C NMR (75 MHz, CD_3OD): δ = 18.6 and 19.8 (3 *C*H$_3$), 28.5 (s, 3 C(*C*H$_3$)$_3$), 49.8 (2 *C*H$_3$OH), 59.4 and 64.9 (3 C*H*$_2$), 70.9 (PhC*H*$_2$), 76.9 and 74.6 (3 N*C*N), 81.9 and 83.0 (3 *C*(CH$_3$)$_3$), 126.3 (*i-Ph*), 130.5, 132.2 and 134.4 (*o,m,p-Ph*), 159.0 and 159.7 (3 *C*=O).

For $C_{31}H_{52}BrN_7O_6 \cdot 2CH_3OH$ calcd: Br 10.48%. Found: 10.58%; 10.60%.

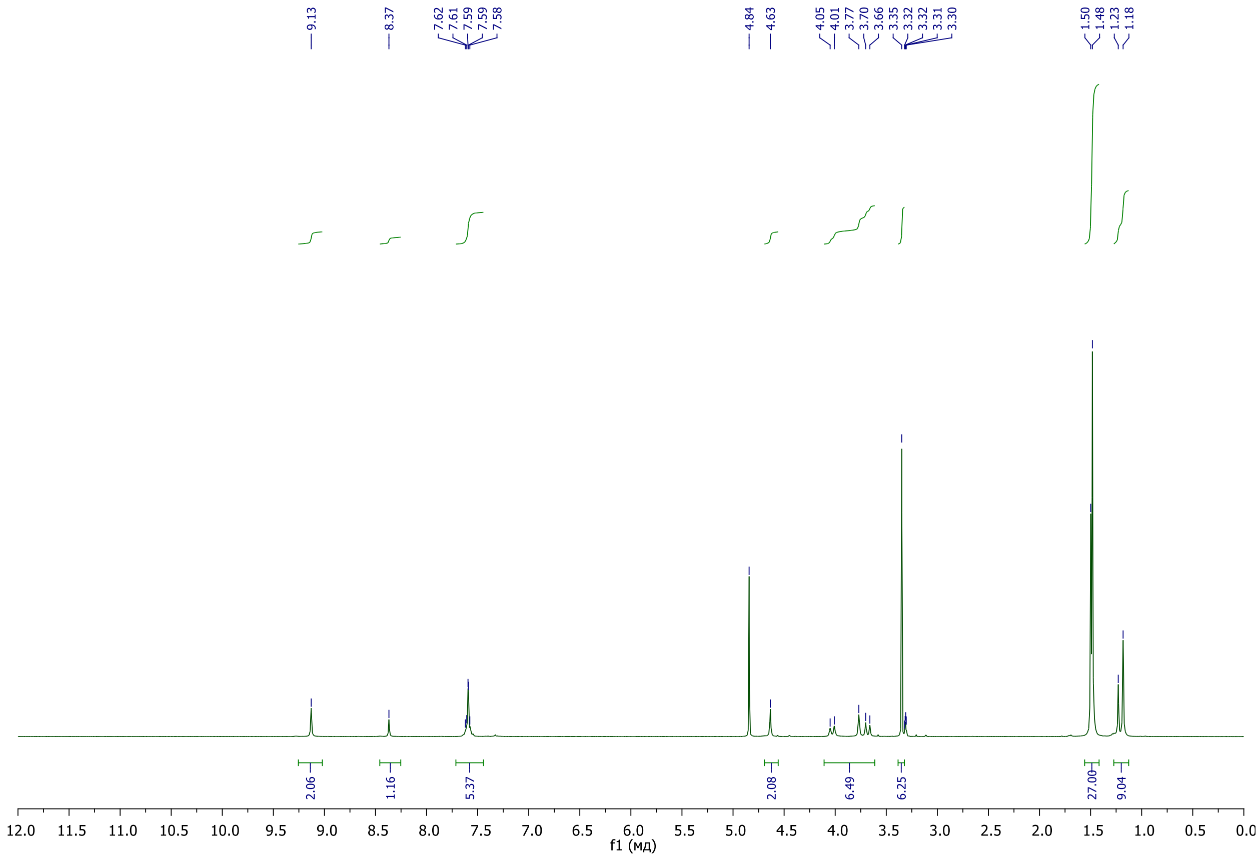

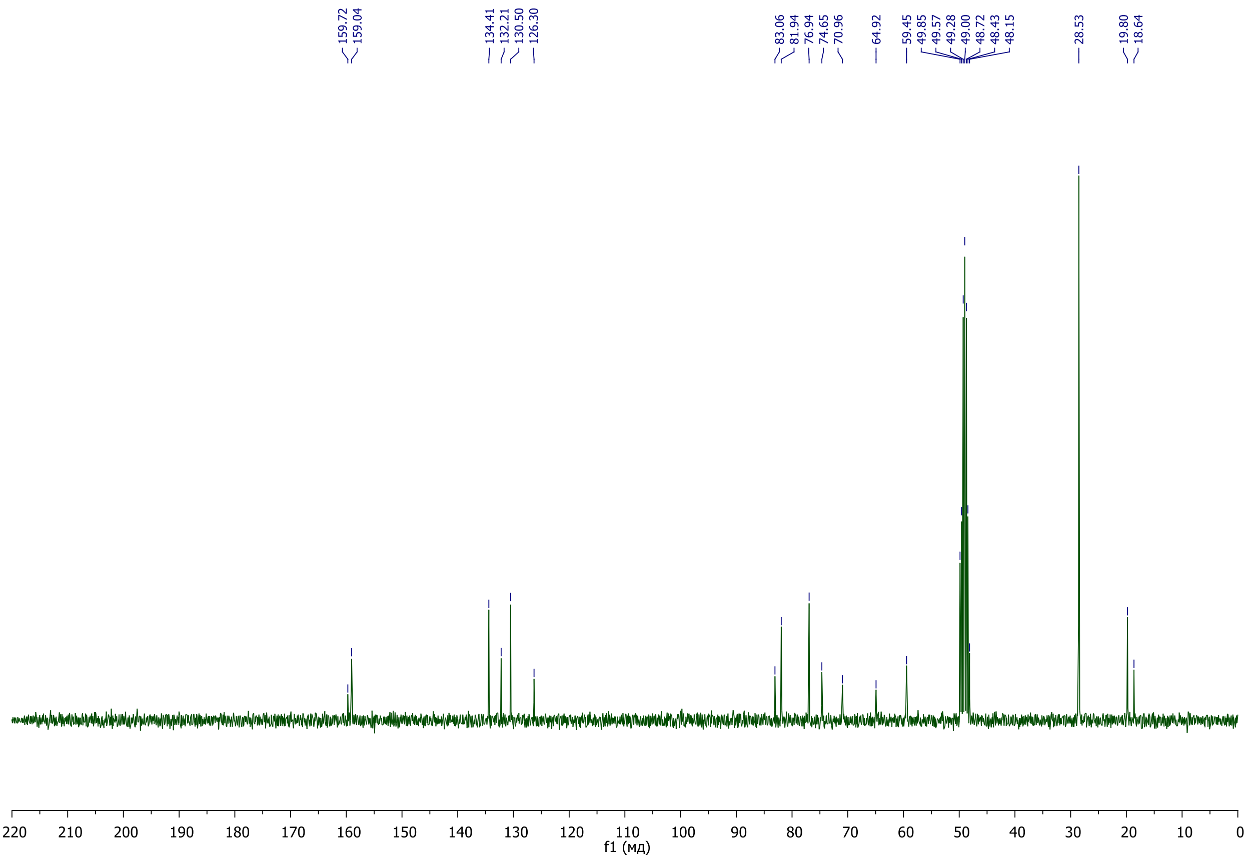
159.72
159.04
134.41
132.21
130.50
126.30
83.06
81.94
76.94
74.65
70.96
64.92
59.45
49.85
49.57
49.28
49.00
48.72
48.43
48.15
28.53
19.80
18.64
f1 (мд)

HSQC

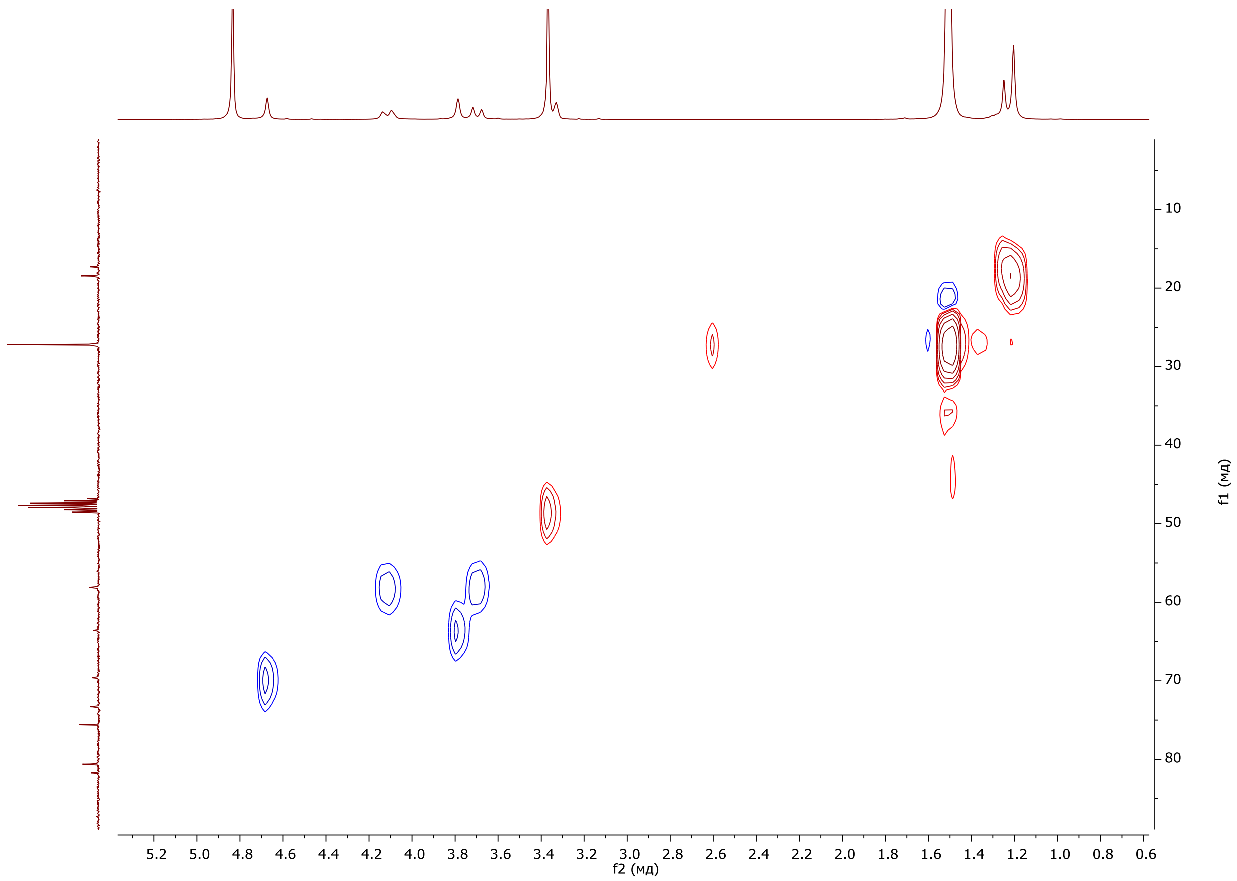
f2 (мд)
f1 (мд)

TAAD quaternary bromide salt Bn-4c

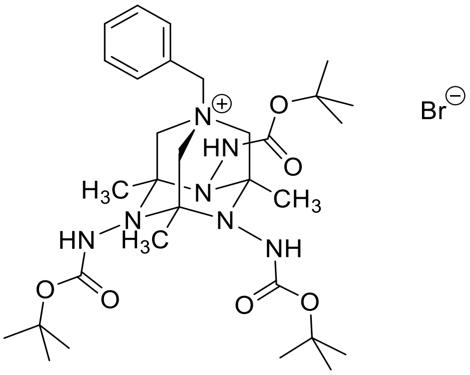

White solid, 177-184 °C with dec.

^{1}H NMR (300 MHz, CD_3OD): δ = 1.18 and 1.23 (2 s, 9 H, 3 C*H*$_3$), 1.48 and 1.50 (2 s, 27 H, 3 C(C*H*$_3$)$_3$), 3.69 and 3.93 (2 d, *J* = 12.3 Hz, 4 H, 2 C*H*$_2$), 3.77 (s, 2 H, C*H*$_2$), 4.61 (s, 2 H, PhC*H*$_2$), 7.59 (m, 5 H, *Ph*).

^{13}C NMR (75 MHz, CD_3OD): δ = 18.6 and 19.8 (3 *C*H$_3$), 28.5 (s, 3 C(*C*H$_3$)$_3$), 59.4 and 65.0 (3 C*H*$_2$), 71.1 (PhC*H*$_2$), 74.6 and 76.9 (3 N*C*N), 82.0 and 83.0 (3 *C*(CH$_3$)$_3$), 126.3 (*i-Ph*), 130.5, 132.2 and 134.4 (*o,m,p-Ph*), 159.0 and 159.6 (3 *C*=O).

HRMS: Calcd for $C_{31}H_{52}N_7O_6^+$ [M-Br$^-$] m/z: 618.3974. Found: 618.3976.

For $C_{31}H_{52}BrN_7O_6$ calcd: Br 11.44%. Found: 11.11%; 11.23%.

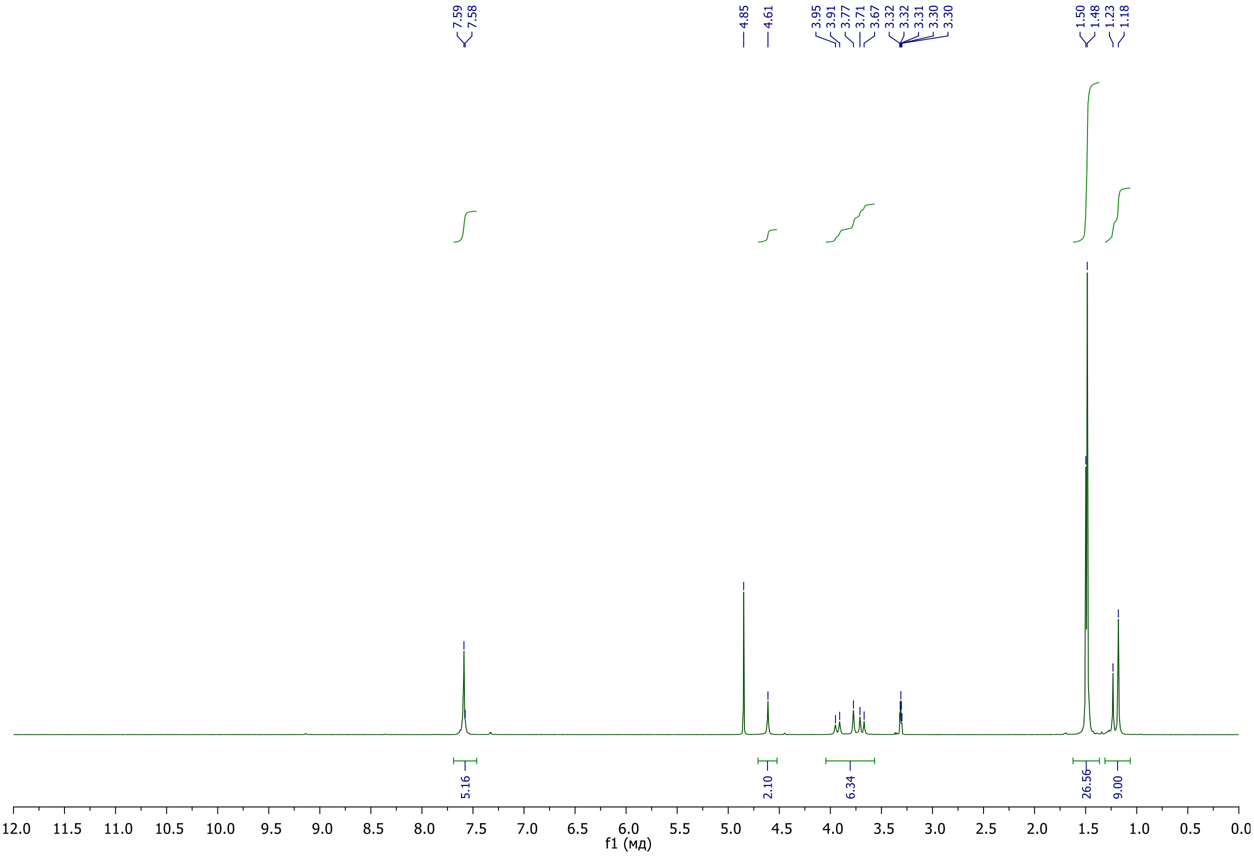

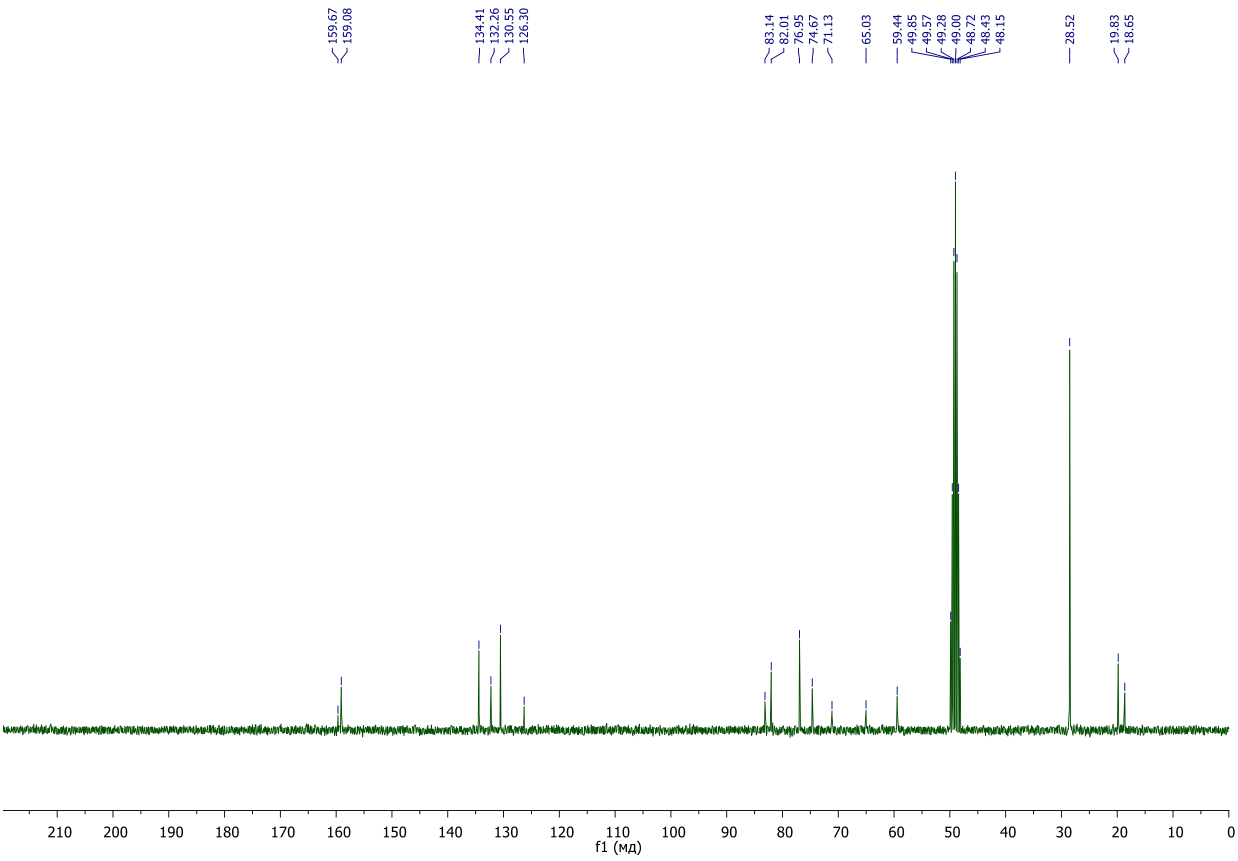
159.67
159.08
134.41
132.26
130.55
126.30
83.14
82.01
76.95
74.67
71.13
65.03
59.44
49.85
49.57
49.28
49.00
48.72
48.43
48.15
28.52
19.83
18.65
210 200 190 180 170 160 150 140 130 120 110 100 90 80 70 60 50 40 30 20 10 0
f1 (мд)

TAAD quaternary salt Bn-4e

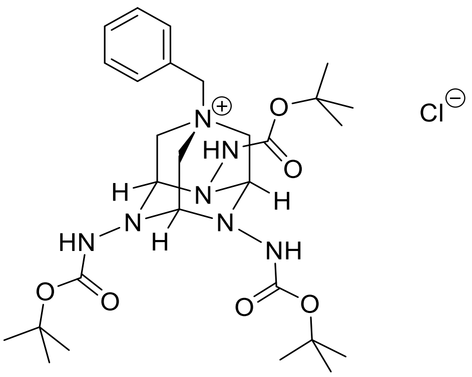

Pale yellow amorphous solid, mp = 176-182 °C

^{1}H NMR (300 MHz, DMSO-d6, 330 K): δ = 1.41 (s, 27 H, 3 C(C*H*$_3$)$_3$), 3.95 (s, 6 H, 3 C*H*$_2$), 4.08 (s, 3 H, 3 C*H*), 4.70 (s, 2 H, PhC*H*$_2$), 7.55 (m, 5 H, *Ph*), 8.50 (s, 3 H, 3 N*H*).

^{13}C NMR (75 MHz, DMSO-d6, 330 K): δ = 27.8 (3 *C*(CH$_3$)$_3$), 54.3 (3 *C*H$_2$), 67.8 (Ph*C*H$_2$), 71.7 (3 N*C*N), 79.1 (3 *C*(CH$_3$)$_3$), 125.4 (*i-Ph*), 128.8, 130.3 and 132.8 (*o,m,p-Ph*), 153.8 (3 *C*=O).

HRMS: Calcd for $C_{28}H_{46}N_7O_6^+$ [M-Cl$^-$] m/z: 576.3504. Found: 576.3496.

For $C_{28}H_{46}ClN_7O_6$ calcd: Cl 5.79%. Found: 5.55%; 5.75%.

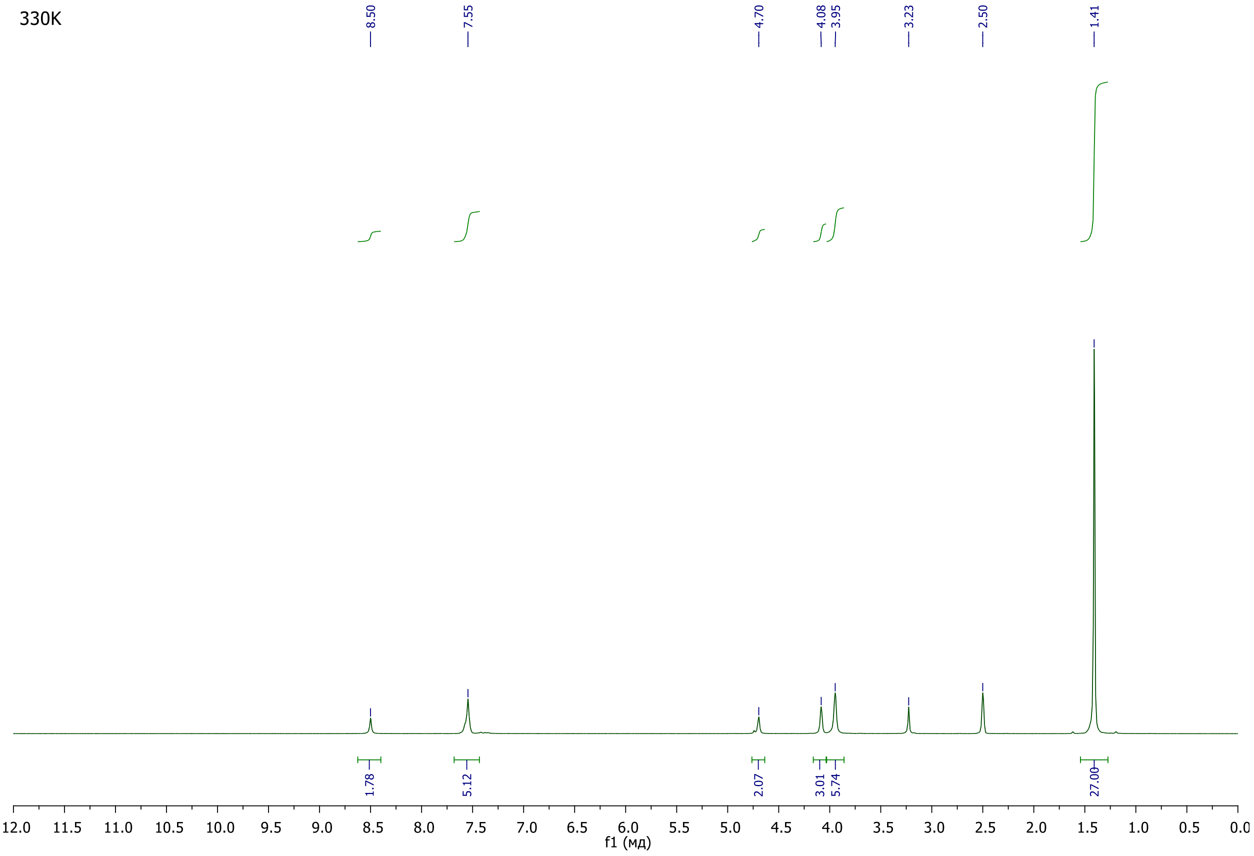

330K

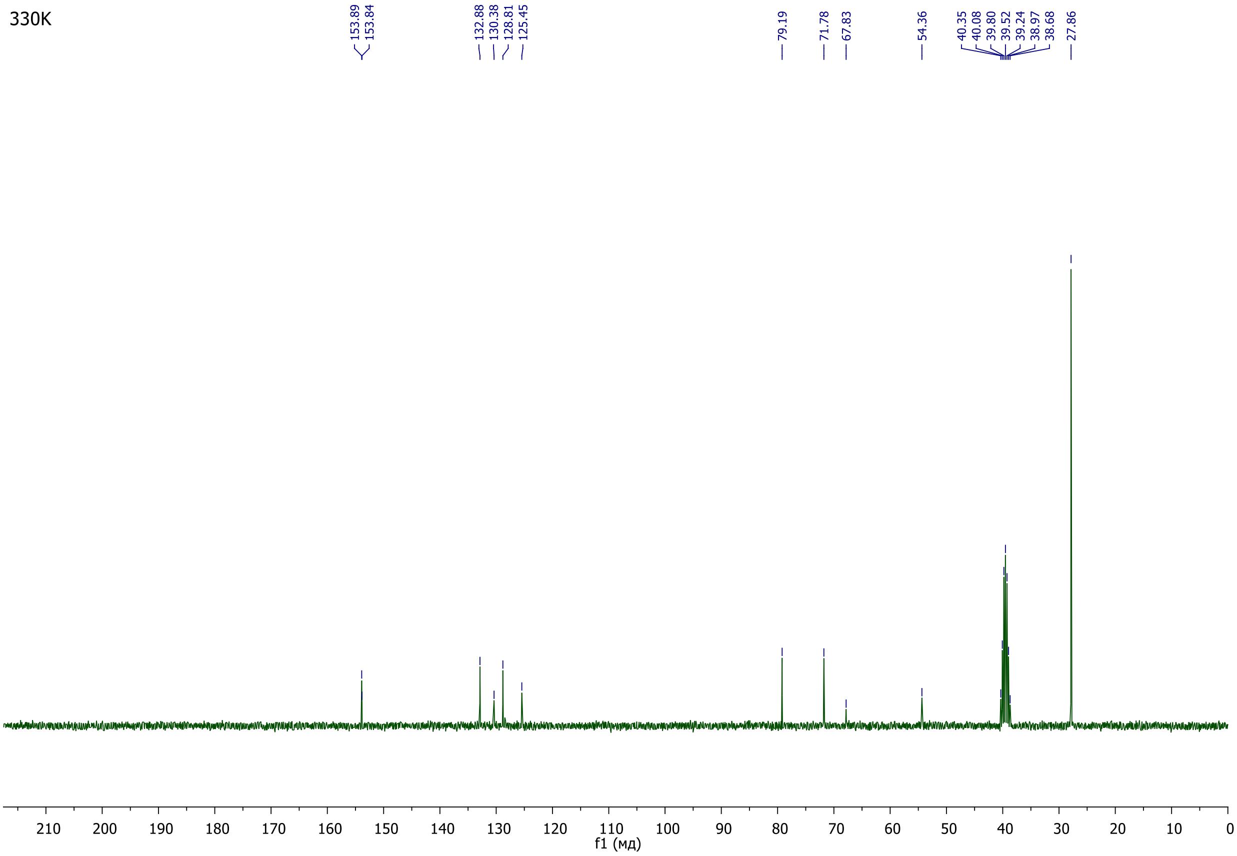

HSQC, 330K

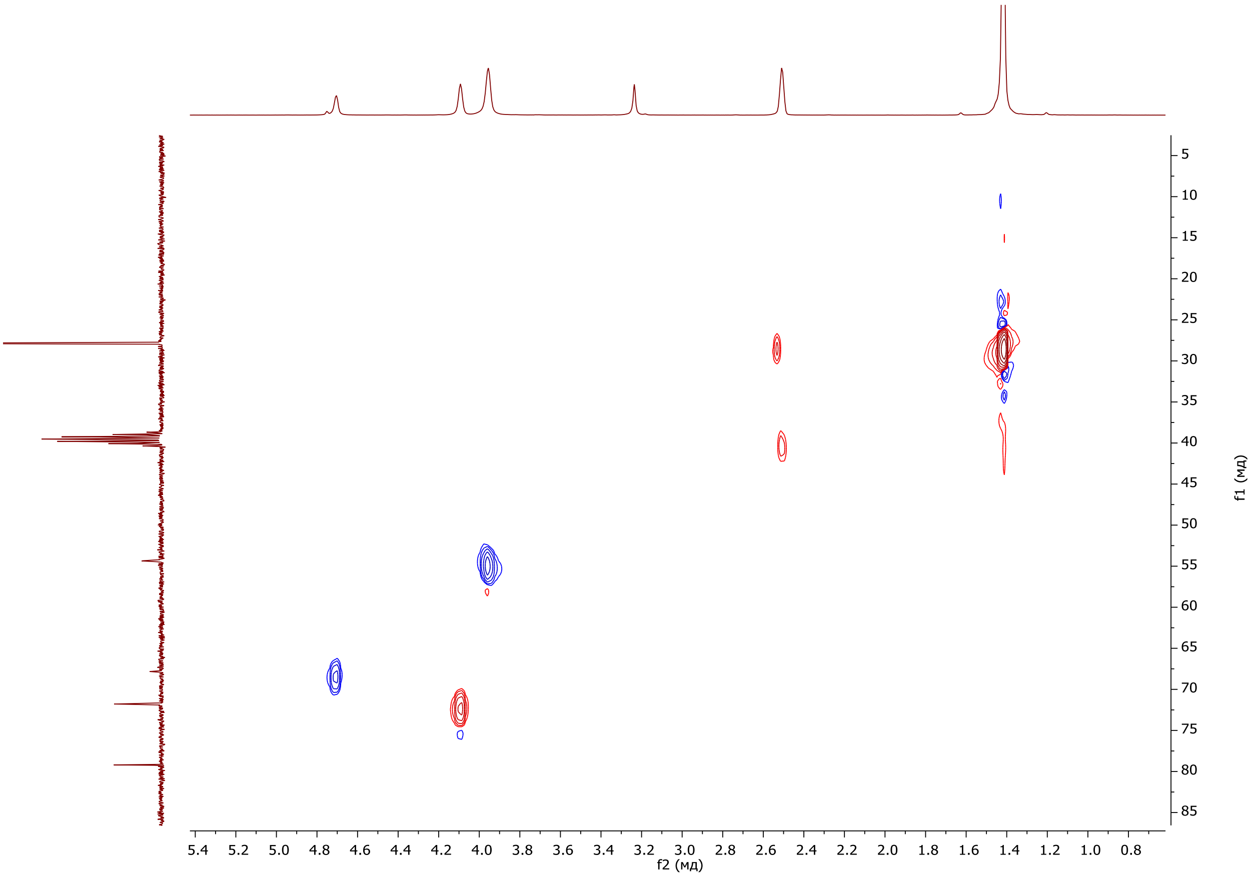

TAAD quaternary salt Bn-6a

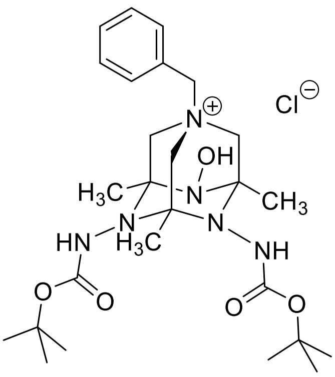

Pale grey amorphous solid, starts softening at 185 °C, mp = 200-206 °C (with dec.)

^{1}H NMR (300 MHz, CD_3OD): δ = 1.1-1.4 (br, 9 H, 2 C*H*$_3$ and C*H*$_3$), 1.46-1.52 (br s, 18 H, 2 C(C*H*$_3$)$_3$) 3.4-3.9 (m br, 6 H, 2 C*H*$_2$ and C*H*$_2$), 4.58 (s, 2 H, PhC*H*$_2$), 7.58 (m, 5 H, *Ph*).

^{13}C NMR (75 MHz, CD_3OD): δ = 18.5, 19.7 and 20.5 (br, 2 *C*H$_3$ and *C*H$_3$), 28.5 (2 C(*C*H$_3$)$_3$), 57-59 (br, 2 *C*H$_2$ and *C*H$_2$), 70.6 (br, Ph*C*H$_2$), 74-77 (2 br, 2 N*C*N and N*C*N), 81.6 and 81.9 (2 *C*(CH$_3$)$_3$), 126.3 and 126.8 (*i-Ph*), 130.4, 132.1, 134.2 and 134.4 (*o*,*m*,*p-Ph*), 158.8 and 159.2 (2 *C*=O).

HRMS: Calcd for $C_{26}H_{43}N_6O_5^+$ [M-Cl$^-$] m/z: 519.3289. Found: 519.3279.

For $C_{26}H_{43}ClN_6O_5$ calcd: Cl 6.39%. Found: 6.26%; 6.24%.

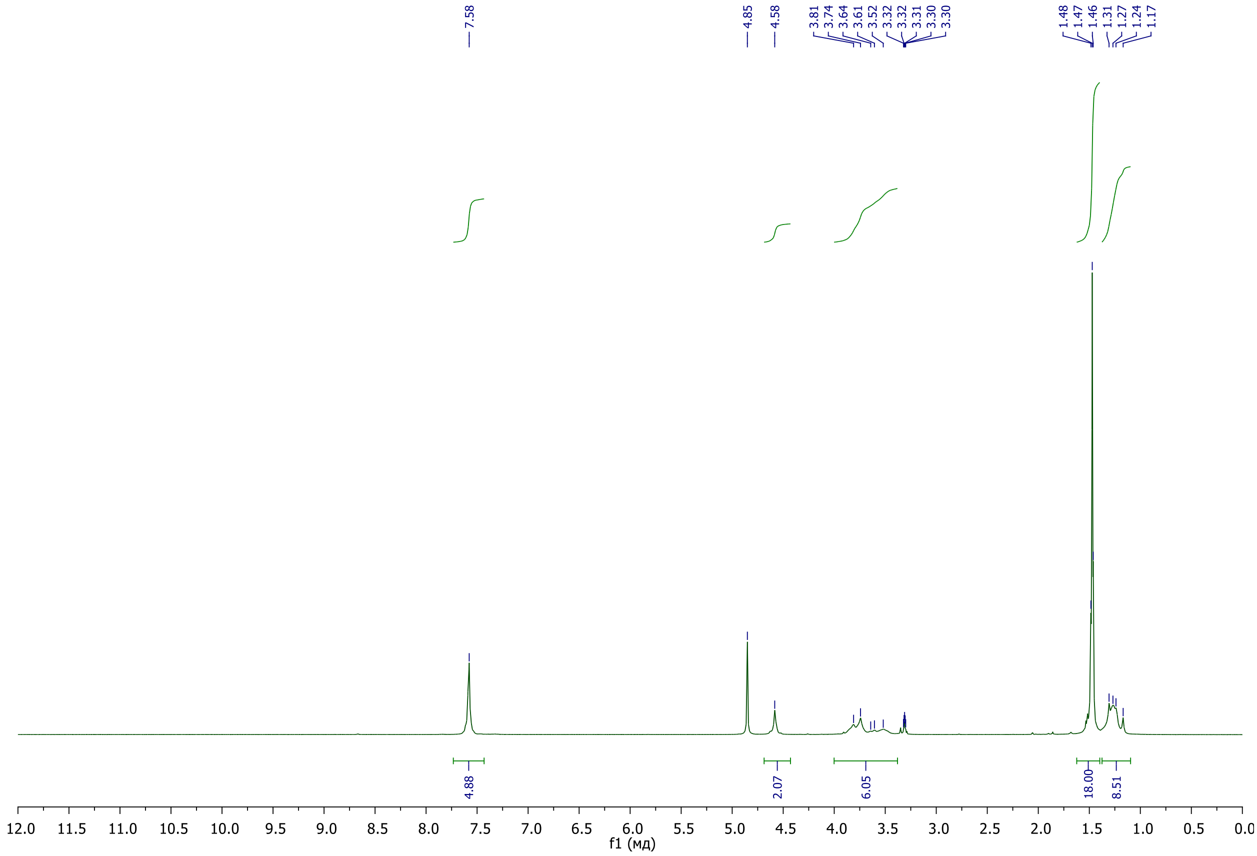

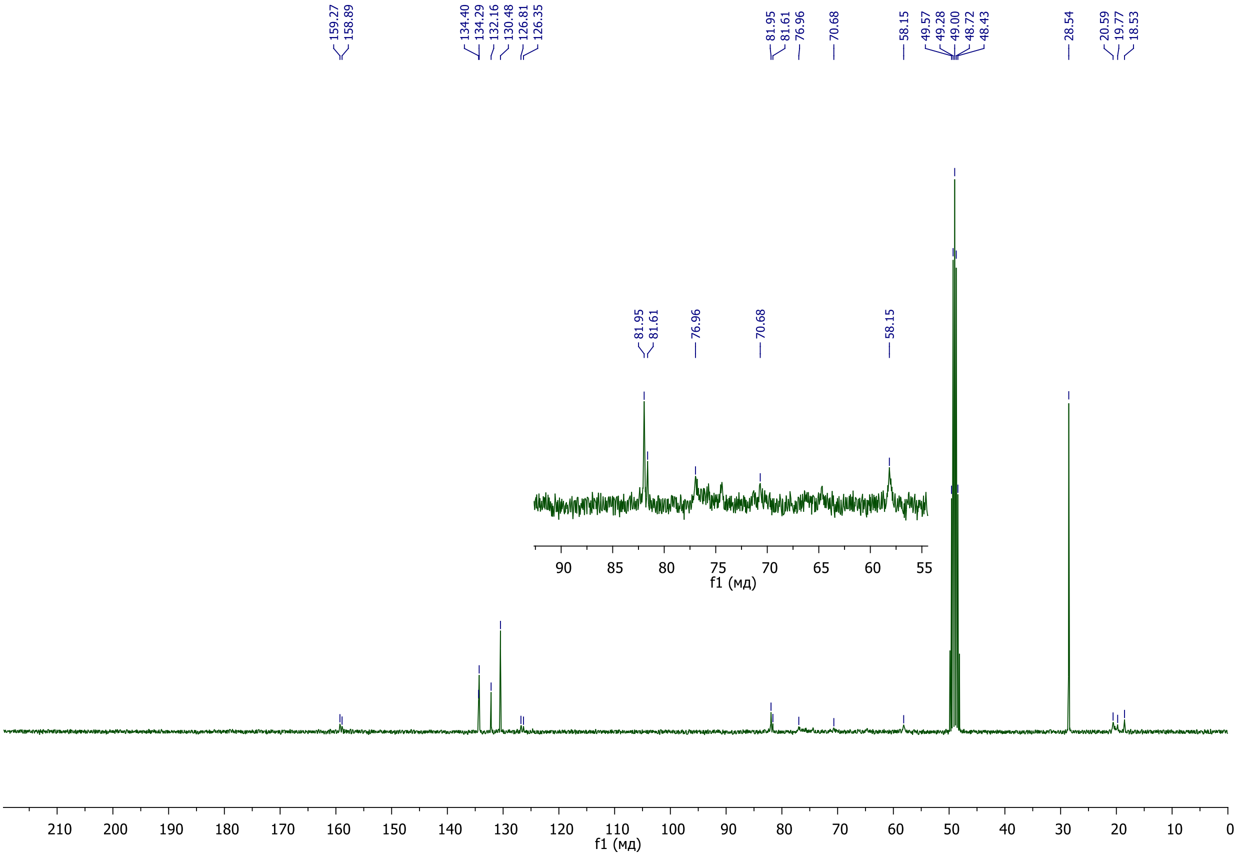
159.27
158.89
134.40
134.29
132.16
130.48
126.81
126.35
81.95
81.61
76.96
70.68
58.15
49.57
49.28
49.00
48.72
48.43
28.54
20.59
19.77
18.53
f1 (мд)

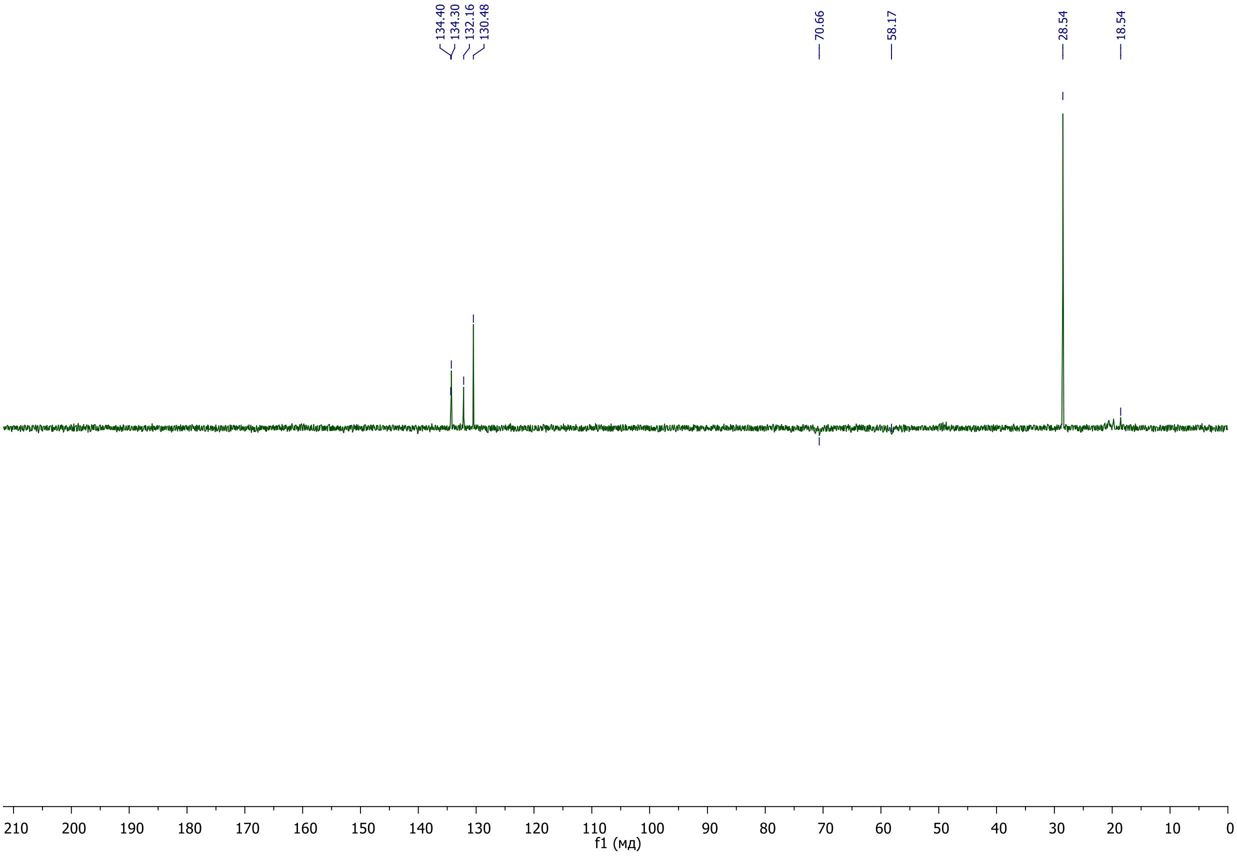
134.40
134.30
132.16
130.48
70.66
58.17
28.54
18.54
f1 (мд)

TAAD quaternary salt Bn-8a

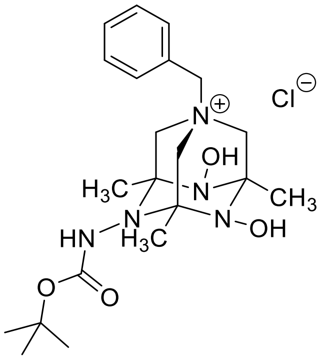

White amorphous solid, starts softening at 208 °C, mp = 230-235 °C (with dec.)

^{1}H NMR (300 MHz, CD_3OD): δ = 1.29 (s, 6 H, 2 C*H*$_3$), 1.41 (s, 3 H, C*H*$_3$), 1.45 (s, 9 H, C(C*H*$_3$)$_3$), 3.44 and 3.58 (2 m, 4 H, 2 C*H*$_2$), 3.78 (d, *J* = 12 Hz, 2 H, C*H*$_2$), 4.63 (s, 2 H, PhC*H*$_2$), 7.65 (m, 5 H, Ph).

^{13}C NMR (75 MHz, CD_3OD): δ = 20.3 and 21.2 (2 *C*H$_3$ and *C*H$_3$), 28.5 (C(*C*H$_3$)$_3$), 55-60 (br, 2 *C*H$_2$ and *C*H$_2$), 70.4 (Ph*C*H$_2$), 75.8 and 78.6 (2 br, 2 N*C*N and N*C*N), 81.8 (*C*(CH$_3$)$_3$), 126.8 (*i-Ph*), 130.3, 132.0 and 134.3 (*o,m,p-Ph*), 158.3 (*C*=O).

HRMS: Calcd for $C_{21}H_{34}N_5O_4^+$ [M-Cl$^-$] m/z: 420.2605. Found: 420.2601.

For $C_{21}H_{34}ClN_5O_4$ calcd: Cl 7.78%. Found: 7.44%; 7.52%.

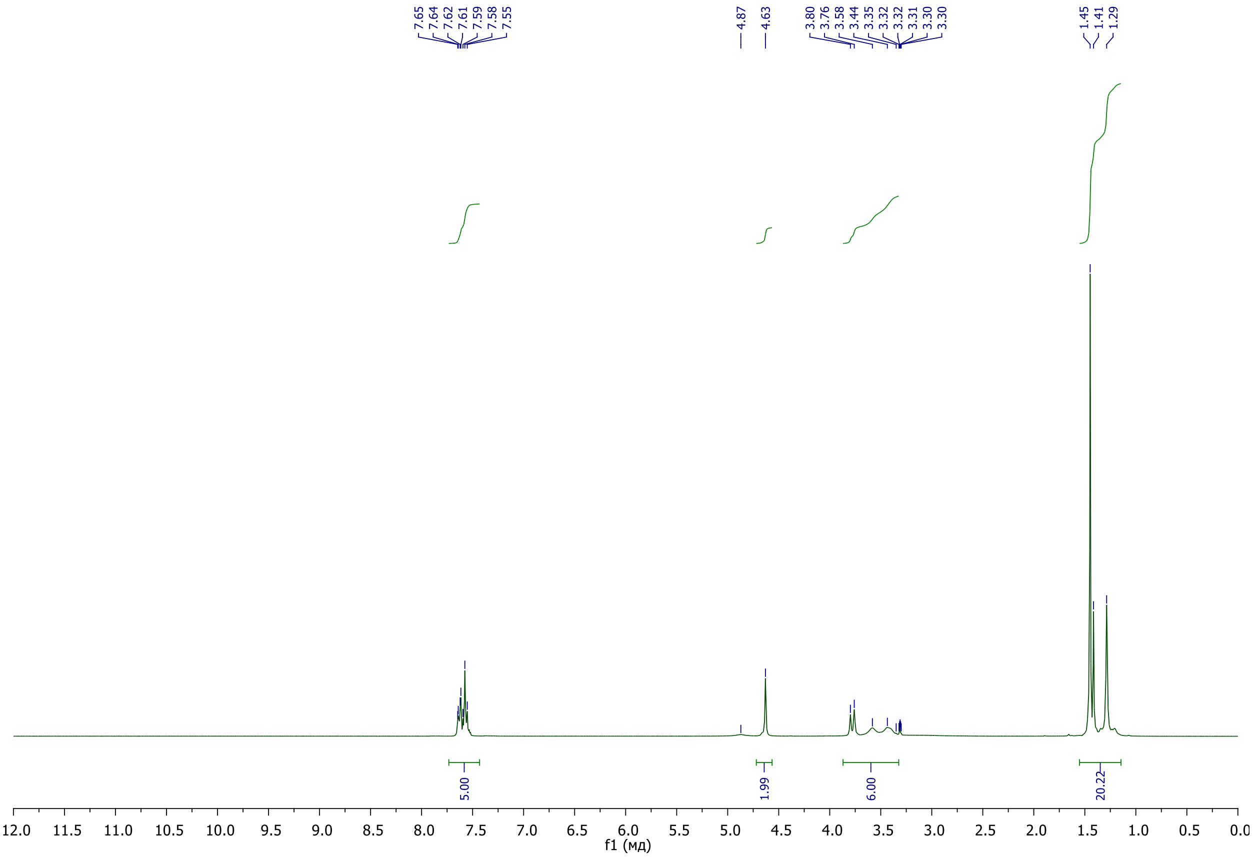

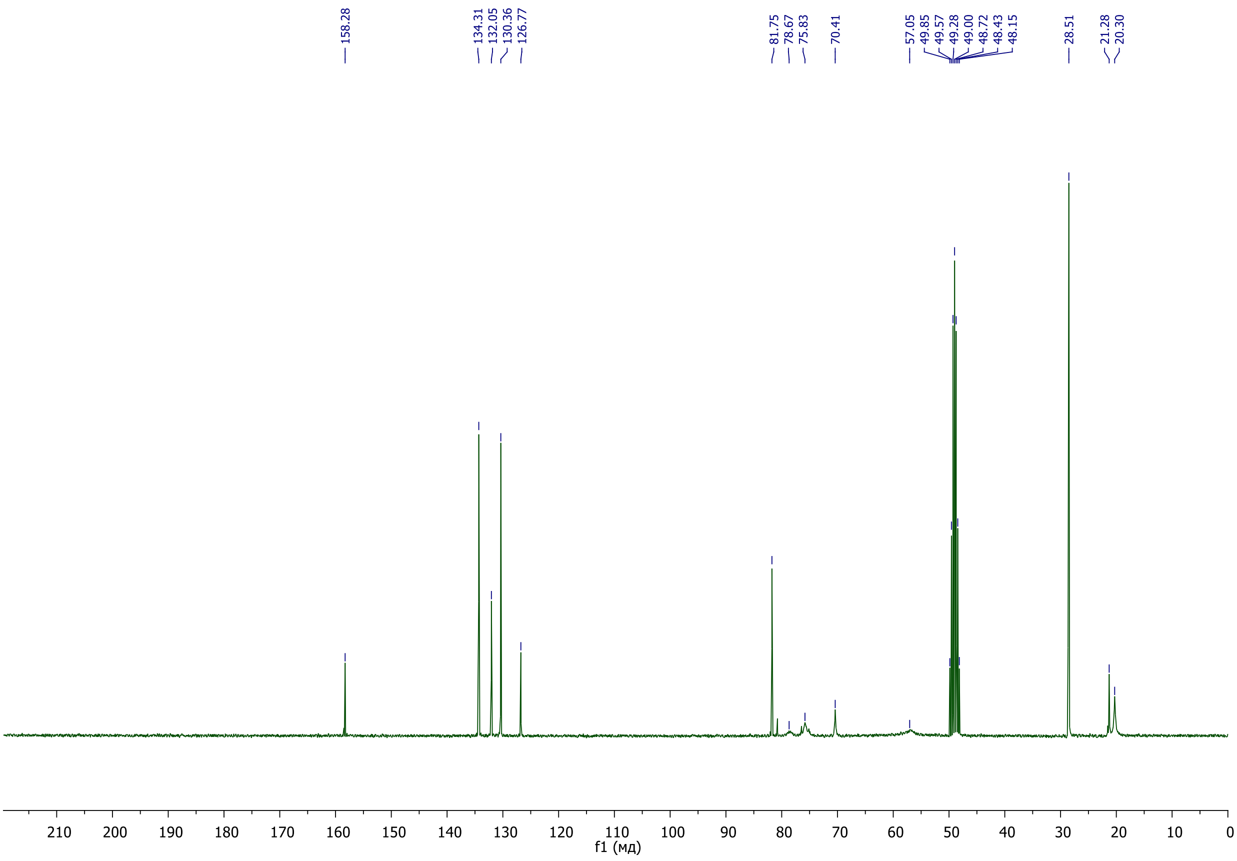
158.28
134.31
132.05
130.36
126.77
81.75
78.67
75.83
70.41
57.05
49.85
49.57
49.28
49.00
48.72
48.43
48.15
28.51
21.28
20.30
210
200
190
180
170
160
150
140
130
120
110
100
90
80
70
60
50
40
30
20
10
0
f1 (мд)

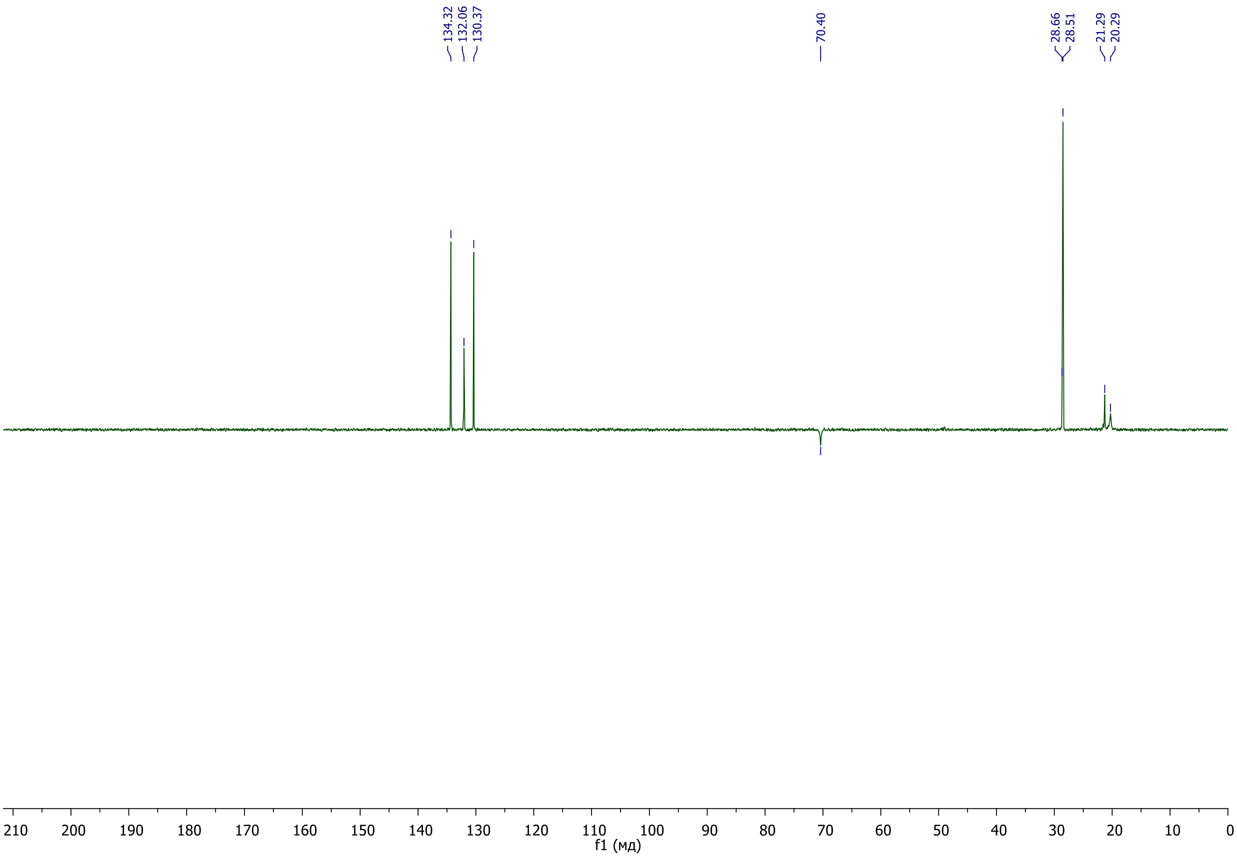
134.32
132.06
130.37
70.40
28.66
28.51
21.29
20.29
210
200
190
180
170
160
150
140
130
120
110
100
90
80
70
60
50
40
30
20
10
0
f1 (мд)

Dynamic mixture of TAAD 15 and ring-chain isomers 16 and 17

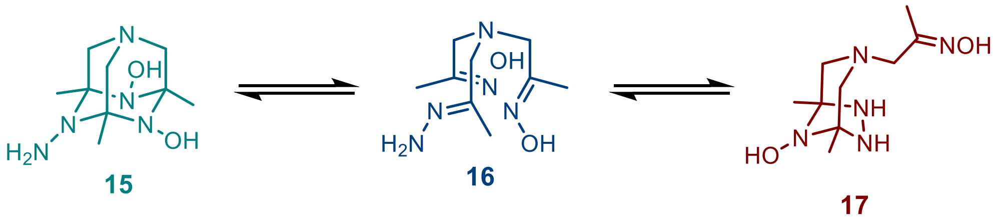

White amorphous solid
HRMS: Calcd for $C_9H_{20}N_5O_2^+$ [M+H$^+$] m/z: 230.1612. Found: 230.1621.

Ratio **15**/**16**/**17** is solvent-dependable and changes with time

Characteristic signals of **15**:
^{1}H NMR (300 MHz, DMSO-d6): δ = 1.0-1.2 (br, 9 H, 2 CH_3 and CH_3), 2.7-3.3 (br, 6 H, 2 CH_2 and CH_2), 7.5-8.2 (br, NH_2 and 2 O*H*).
^{13}C NMR (75 MHz, DMSO-d6): δ = 21.7 (br, 2 *C*H_3 and *C*H_3), 52-56 and 60-65 (2 br, 2 *C*H_2 and *C*H_2), 73.2 and 74.9 (2 N*C*N and N*C*N).
^{1}H NMR (300 MHz, D_2O): δ = 1.2-1.4 (br, 9 H, 2 CH_3 and CH_3), 2.7-3.3 (br, 6 H, 2 CH_3 and CH_3).
^{13}C NMR (75 MHz, D_2O): δ = 19.7 (br, 2 *C*H_3 and *C*H_3), 50-65 (br, 2 *C*H_2 and *C*H_2), 74.0 and 75.1 (2 N*C*N and N*C*N).

Characteristic signals of **16**:
^{1}H NMR (300 MHz, DMSO-d6): δ = 1.66 and 1.75 (2 s, 9 H, 2 CH_3 and CH_3), 2.84 and 2.86 (2 s, 6 H, 2 CH_2 and CH_2), 10.4-10.8 (br, 2OH).
^{13}C NMR (75 MHz, DMSO-d6): δ = 12.6 and 12.8 (2 *C*H_3 and *C*H_3), 57.5 and 60.7 (2 *C*H_2 and *C*H_2), 145.2 and 154.1 (2 *C*=N and *C*=N).

Characteristic signals of **17**:
^{1}H NMR (300 MHz, D_2O): δ = 1.24 (s, 6 H, 2 CH_3), 1.85 (s, 3 H, CH_3), 2.28 and 2.68 (2 d, J = 12 Hz, 4 H, 2 CH_2), 2.96 (s, 2 H, CH_2).
^{13}C NMR (75 MHz, D_2O): δ = 12.2 (*C*H_3), 16.7 (2 *C*H_3), 58.6 and 61.5 (2 *C*H_2 and *C*H_2), 83.3 (2 N*C*N), 158.6 (*C*=N).

^{1}H and ^{13}C NMR spectra in DMSO-d_6 immediately after preparation

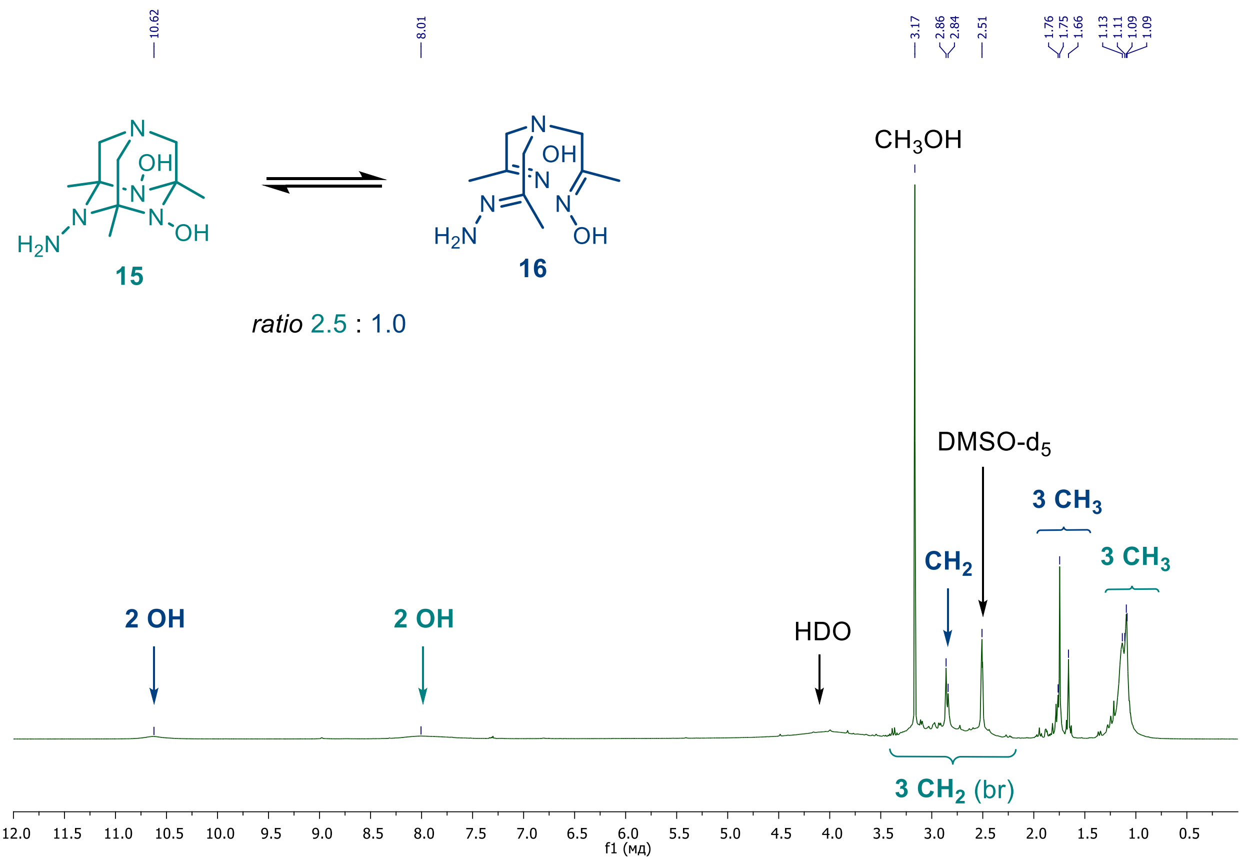

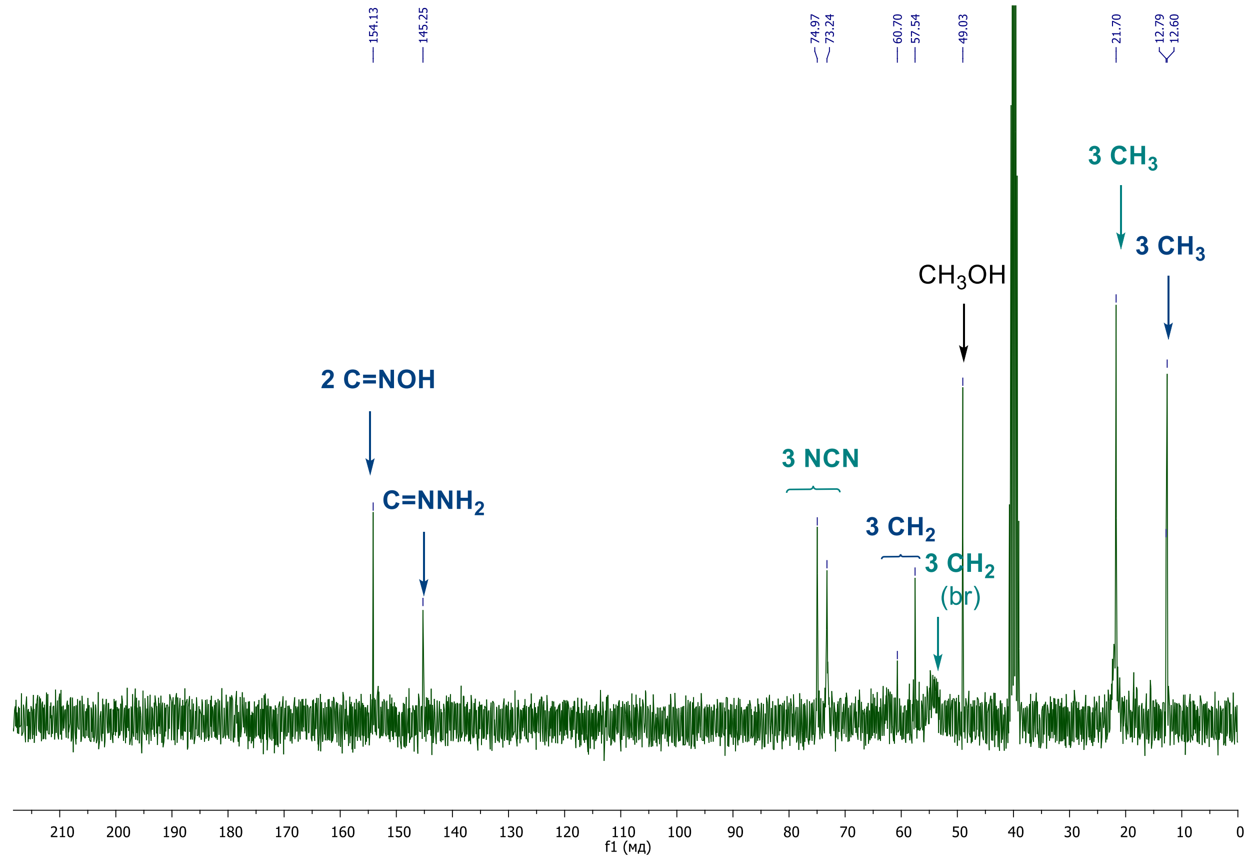

^{1}H NMR spectra in D_2O immediately after preparation

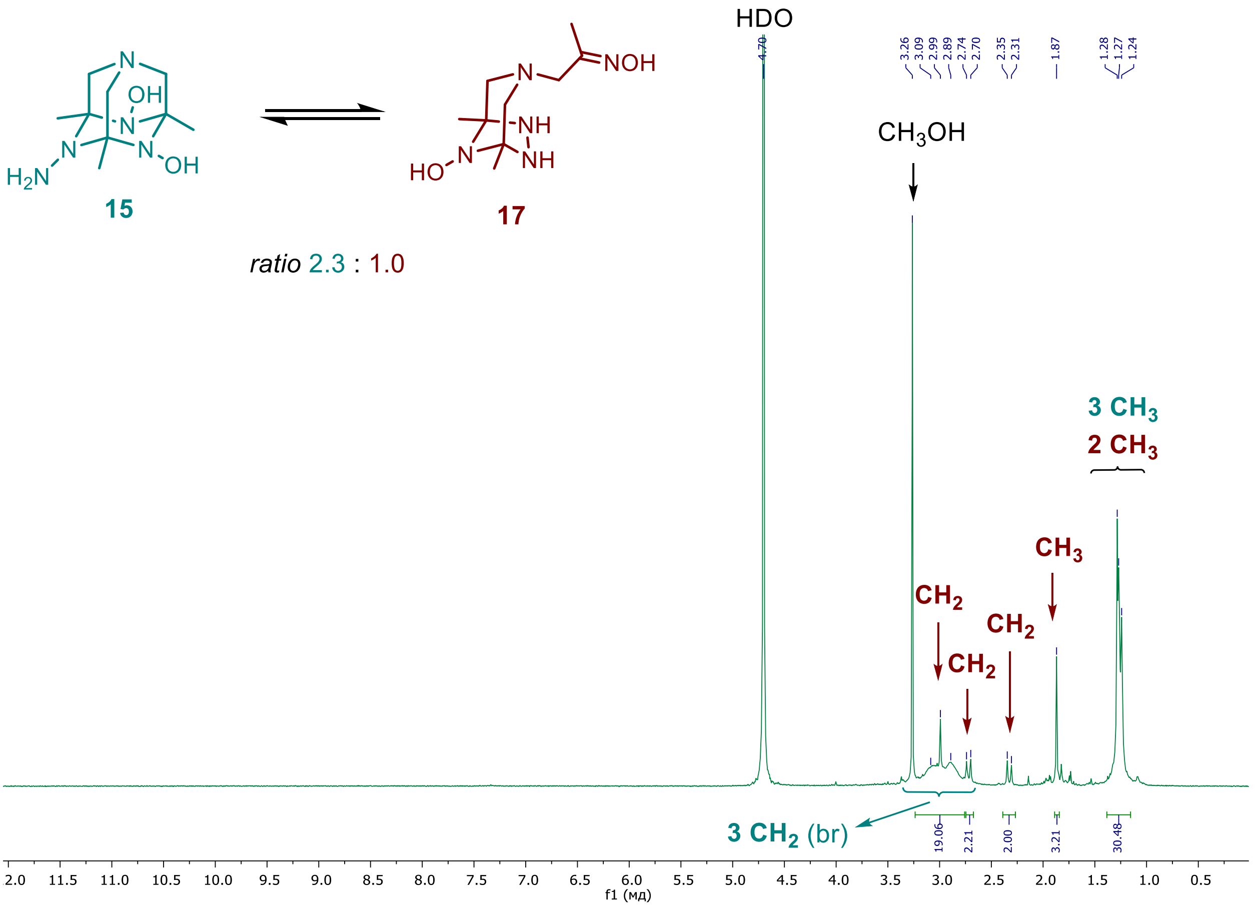

^{1}H NMR spectra in D_2O after 5 d at rt.

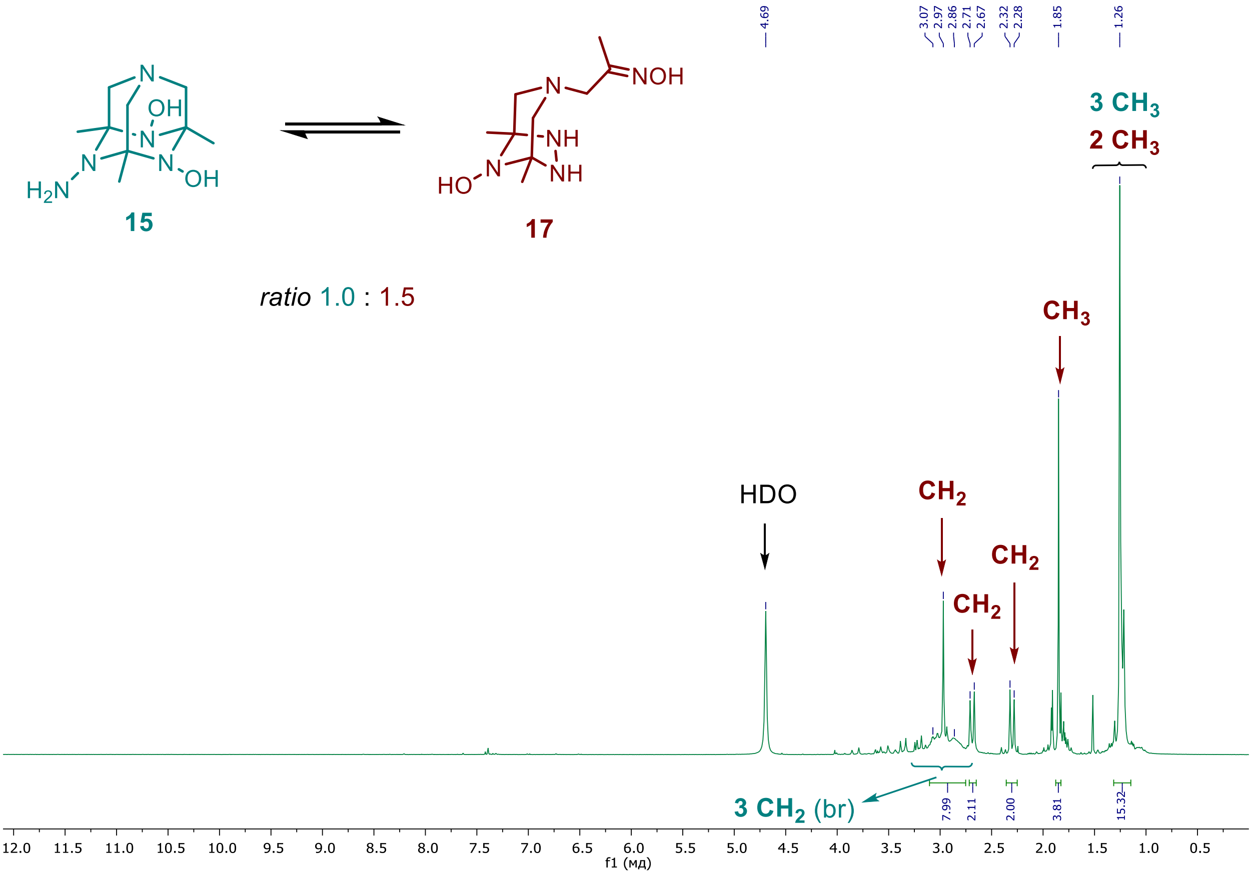

^{1}H and ^{13}C NMR spectra in D_2O after ca. 12 h at rt.

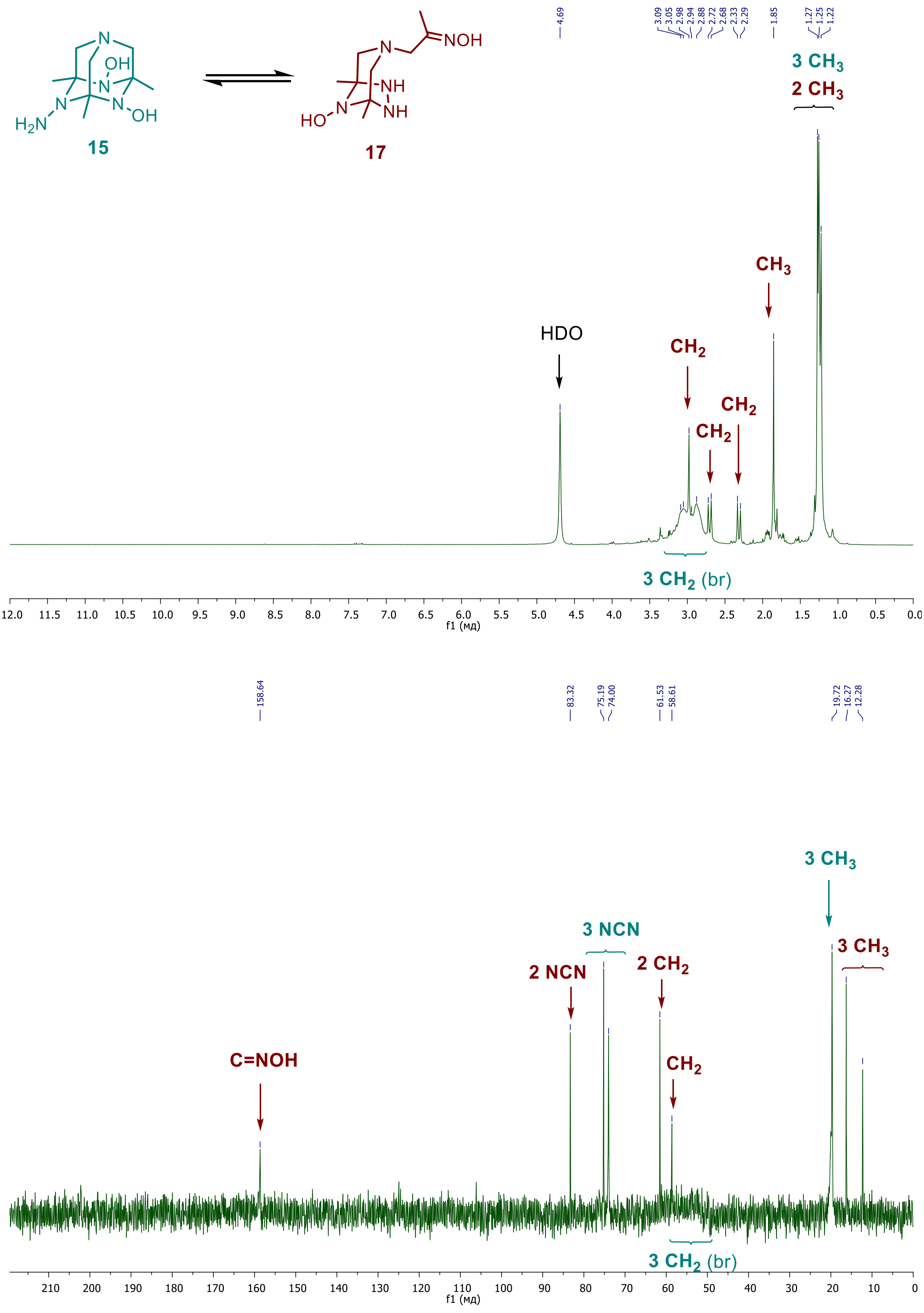

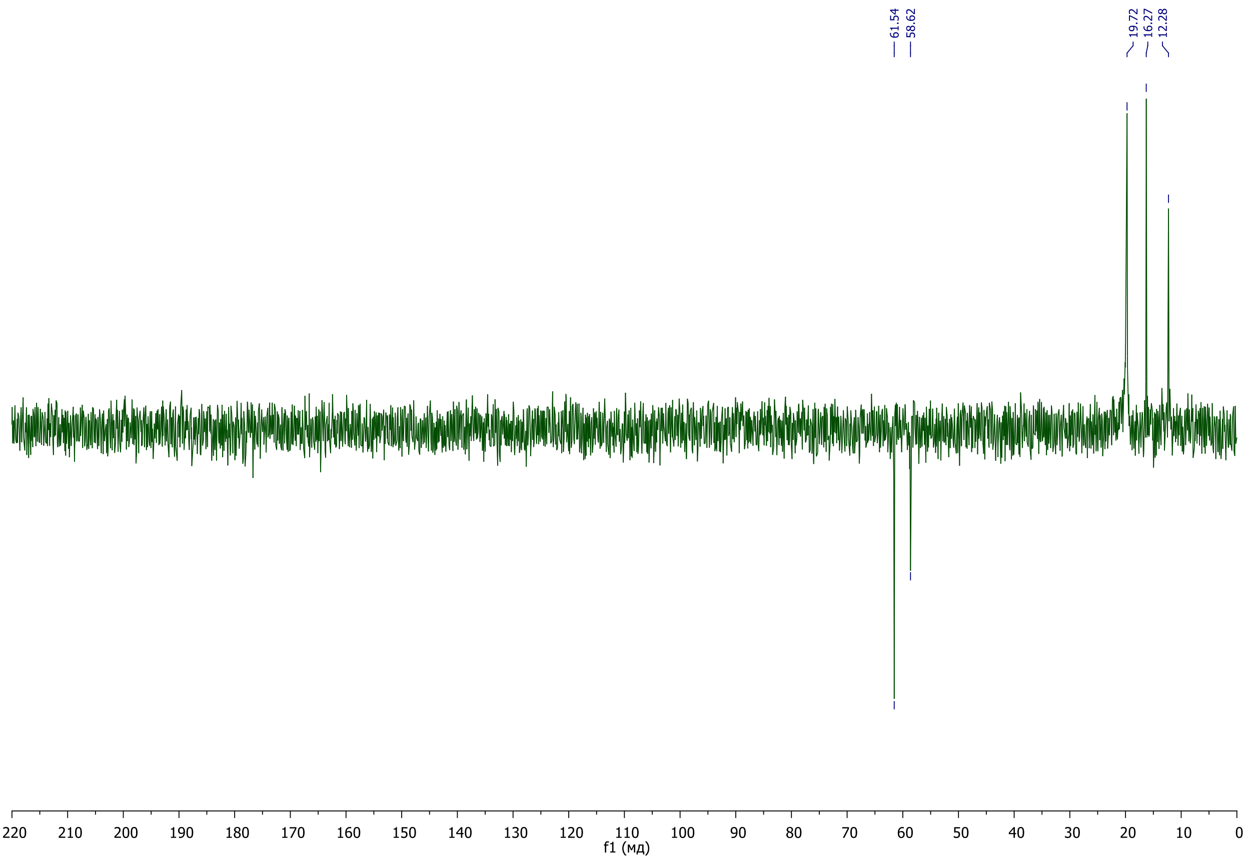

61.54
58.62
19.72
16.27
12.28
220
210
200
190
180
170
160
150
140
130
120
110
100
90
80
70
60
50
40
30
20
10
0
f1 (мд)

HSQC

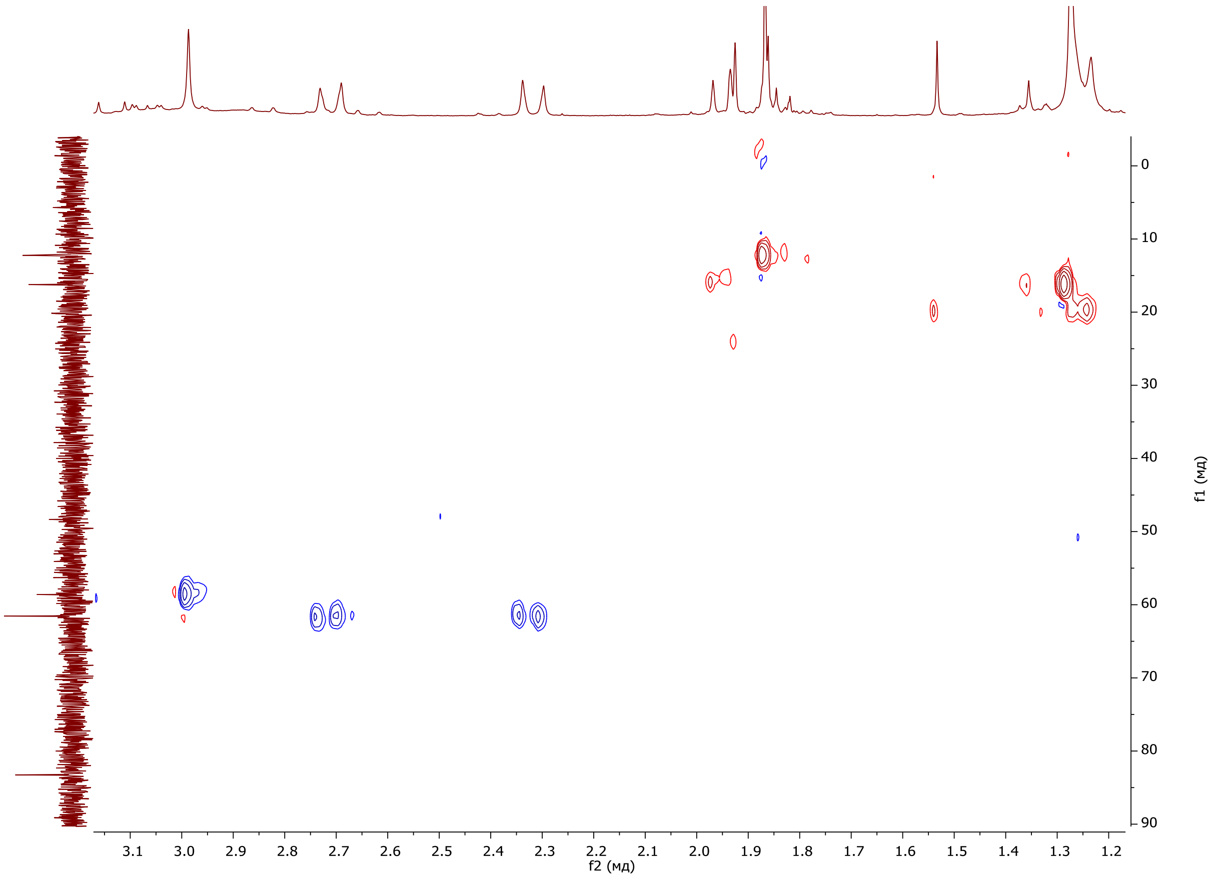

3.1
3.0
2.9
2.8
2.7
2.6
2.5
2.4
2.3
2.2
2.1
2.0
1.9
1.8
1.7
1.6
1.5
1.4
1.3
1.2
f2 (мд)
f1 (мд)

i

Azaoxaboradiadamantane 18

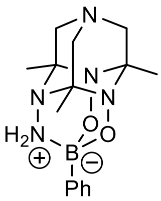

White solid, mp = 125-131 °C (with dec.)

^{1}H NMR (300 MHz, D_2O): δ = 1.61 (s, 3 H, C*H₃*), 1.65 (s, 6 H, 2 C*H₃*), 3.14 (m, 6 H, 2 C*H₂* and C*H₂*), 7.39 and 7.49 (2 m, 5 H, *Ph*).

^{13}C NMR (75 MHz, D_2O): δ = 19.8 (2 *C*H_3), 20.0 (*C*H_3), 59.4 (2 *C*H_2), 59.8 (*C*H_2), 72.4 (2 N*C*N), 73.0 (N*C*N), 127.9, 128.5 and 130.7 (*o*,*m*,*p*-*Ph*). *C*-B signal is not observed.

^{11}B NMR (96 MHz, D_2O): 0.33 (br).

HRMS: Calcd for $C_{15}H_{23}BN_5O_2^+$ [M+H$^+$] m/z: 316.1942. Found: 316.1941.

Calcd for $C_{15}H_{21}BN_5O_2^-$ [M-H$^+$] m/z: 314.1797. Found: 314.1795.

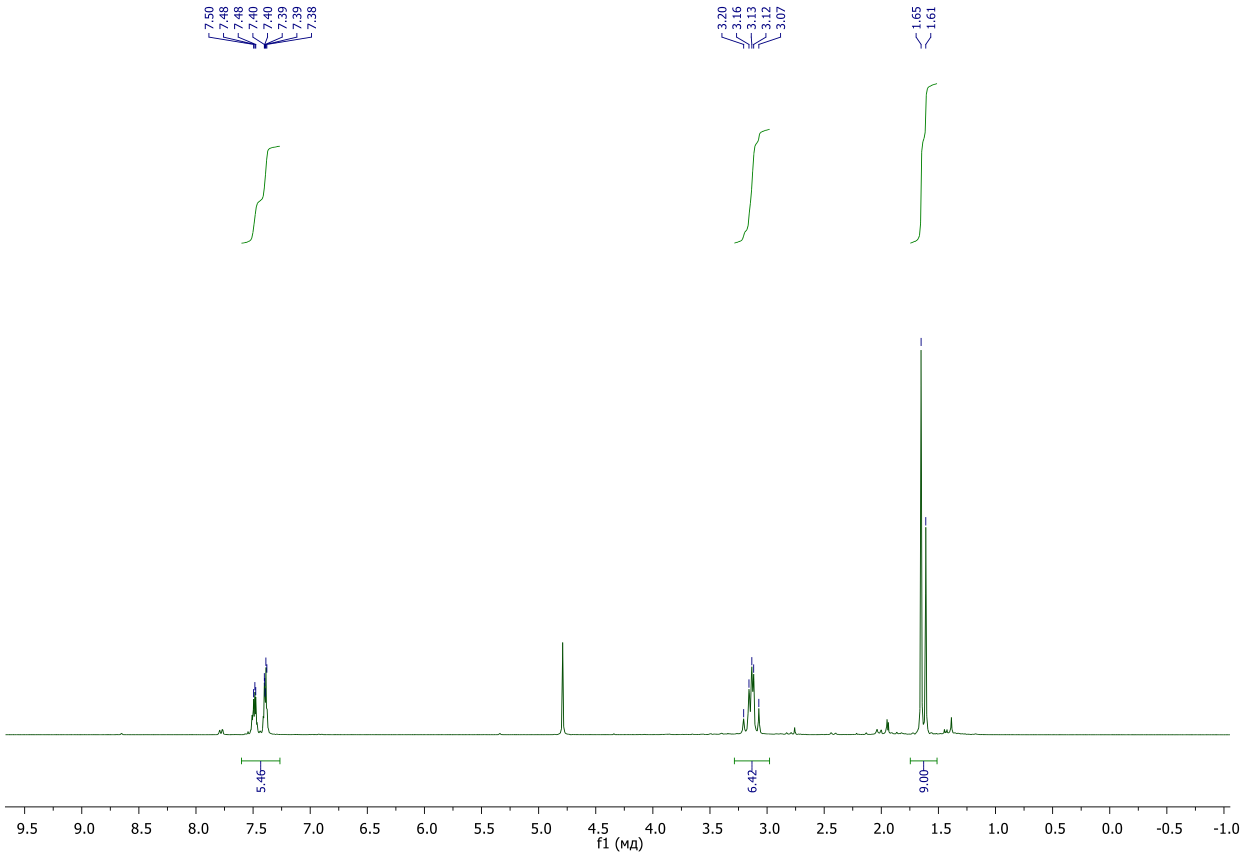

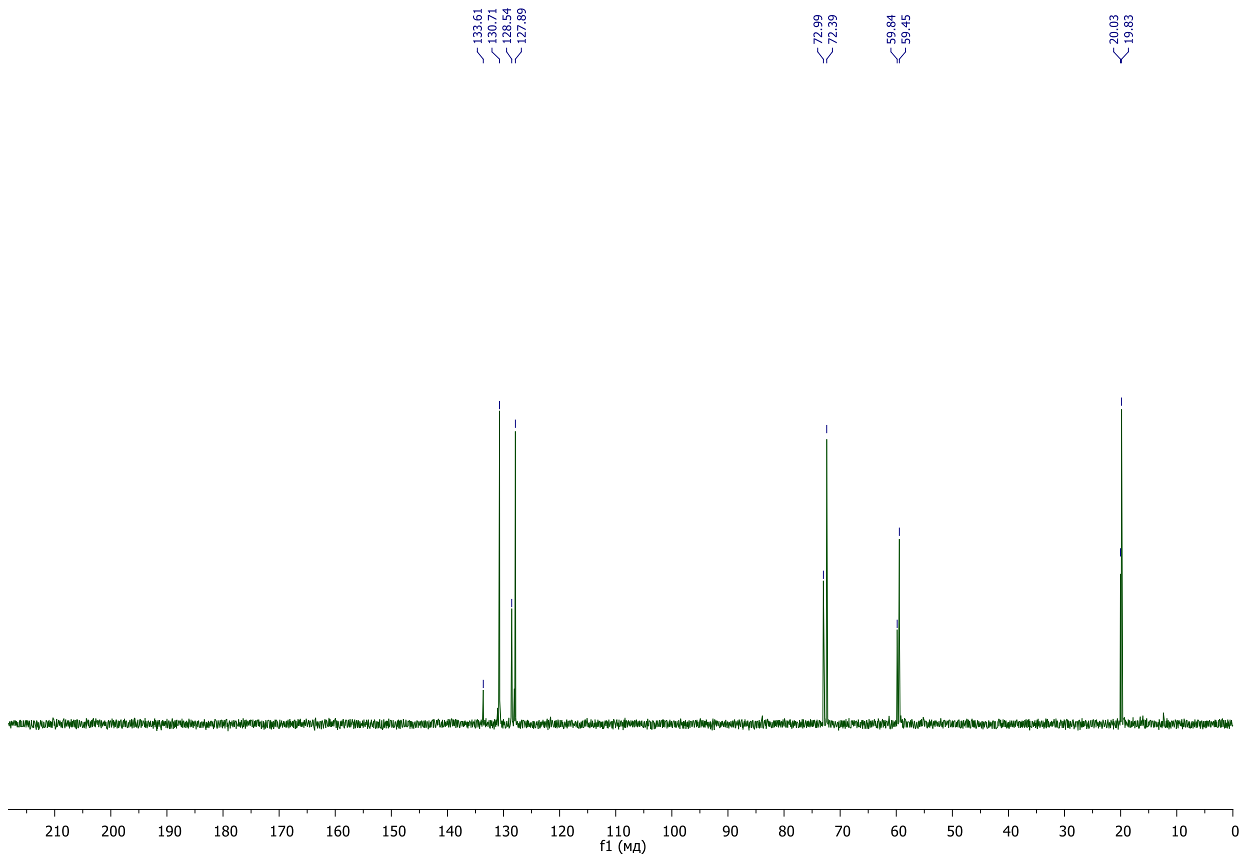
133.61
130.71
128.54
127.89
72.99
72.39
59.84
59.45
20.03
19.83
f1 (мд)

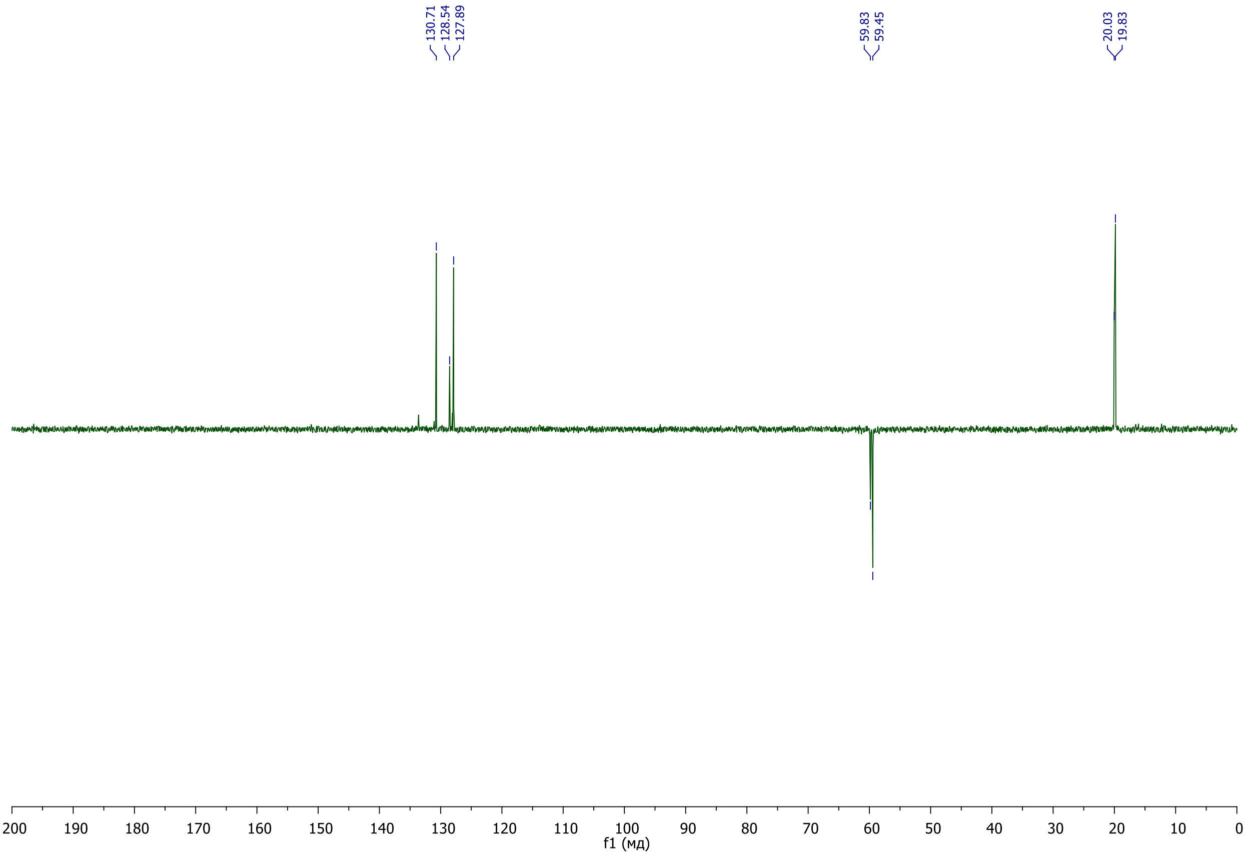
130.71
128.54
127.89
59.83
59.45
20.03
19.83
f1 (мд)

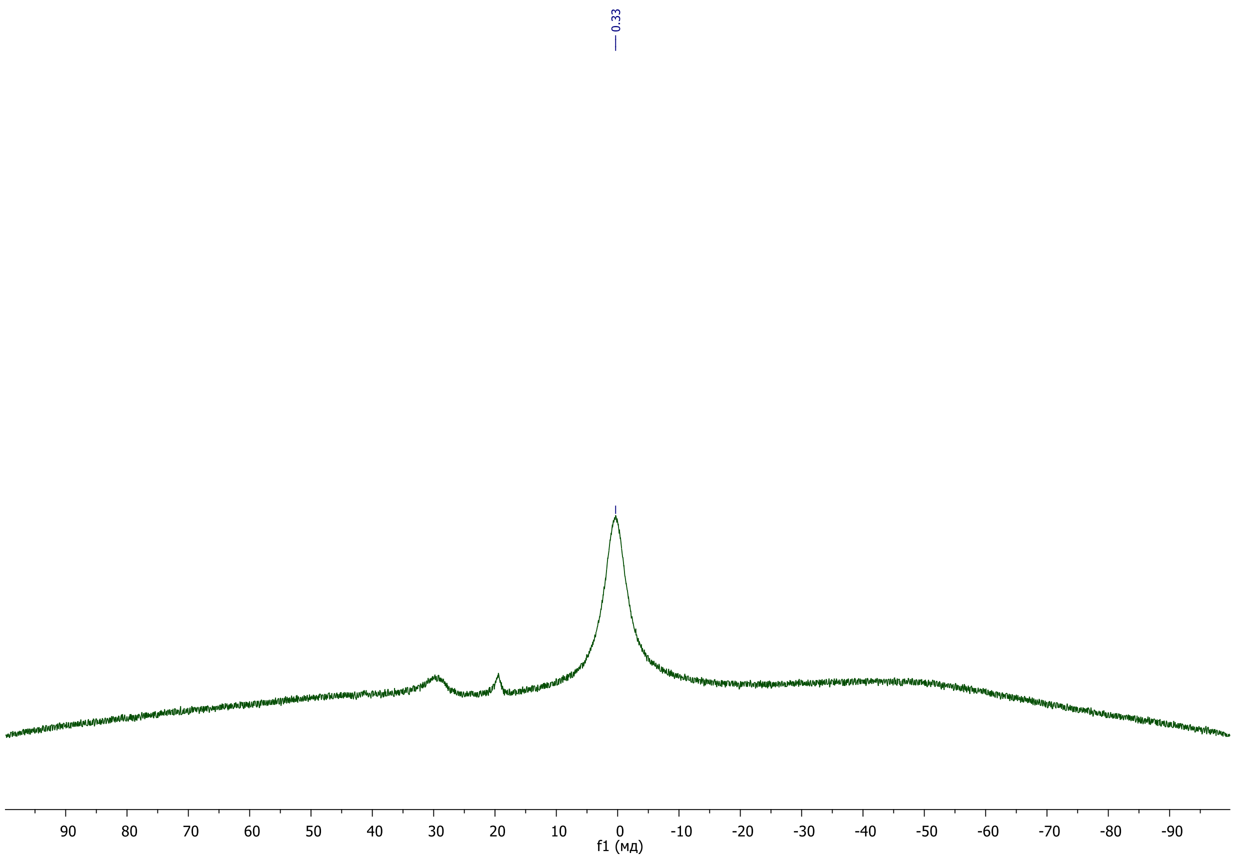

0.33
90
80
70
60
50
40
30
20
10
0
-10
-20
-30
-40
-50
-60
-70
-80
-90
f1 (мд)

TAAD 19c

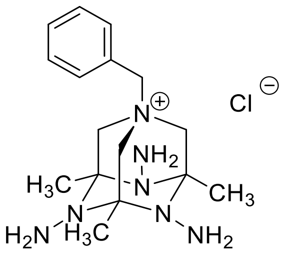

White solid, mp = 165-172 °C (with dec.)
^{1}H NMR (300 MHz, D_2O): δ = 1.44 (s, 9 H, 3 C*H*$_3$), 3.59 (s, 6 H, 3 C*H*$_2$), 4.57 (s, 2 H, PhC*H*$_2$), 7.5-7.7 (m, 5 H, *Ph*).
^{13}C NMR (75 MHz, D_2O): δ = 21.2 (3 C*H*$_3$), 58.2 (3 C*H*$_2$), 70.0 (PhC*H*$_2$), 74.5 (3 N*C*N), 124.5 (*i-Ph*), 129.4, 131.2 and 133.1 (*o,m,p-Ph*).
HRMS: Calcd for $C_{16}H_{28}N_7^+$ [M-Cl$^-$] m/z: 318.2401. Found: 318.2411.

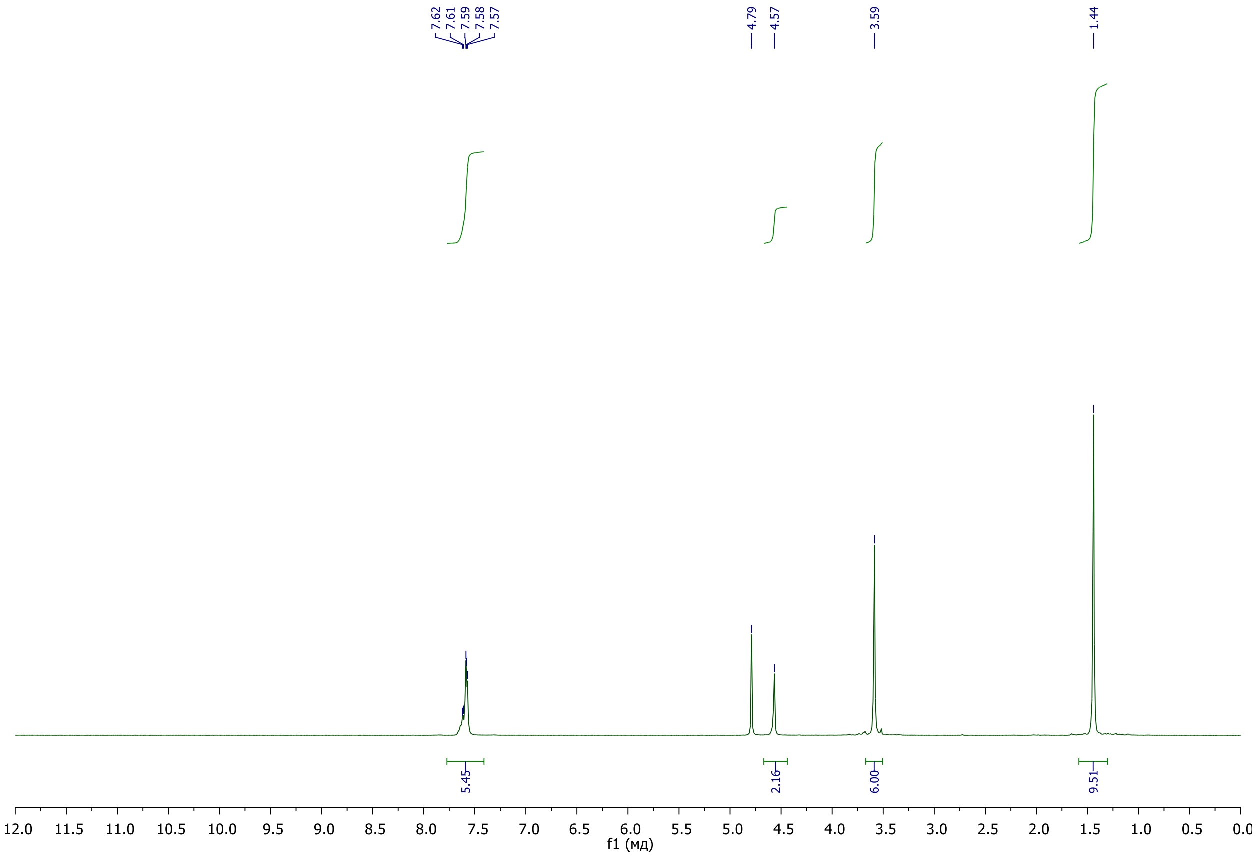

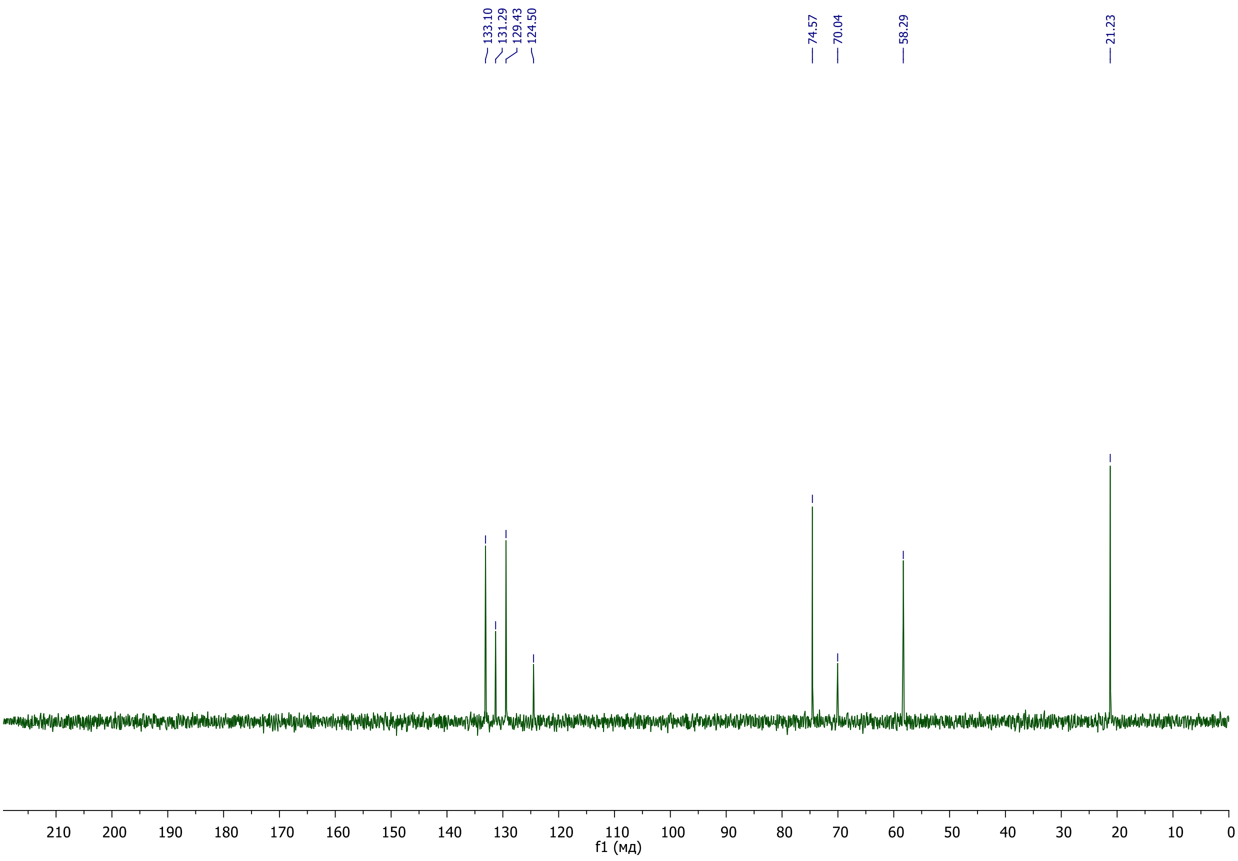
133.10
131.29
129.43
124.50
74.57
70.04
58.29
21.23
210 200 190 180 170 160 150 140 130 120 110 100 90 80 70 60 50 40 30 20 10 0
f1 (мд)

TAAD 19e

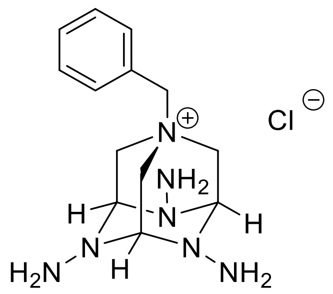

White solid, softening at 130 °C, decomposition 190-200 °C.

^{1}H NMR (300 MHz, D_2O): δ = 3.75 (s, 6 H, 3 C*H*$_2$), 4.55 (s, 3 H, 3 C*H*), 4.20 (s, 2 H, PhC*H*$_2$), 7.5-7.6 (m, 5 H, *Ph*).

^{13}C NMR (75 MHz, D_2O): δ = 54.9 (3 C*H*$_2$), 70.1 (PhC*H*$_2$), 74.1 (3 *C*H), 124.6 (*i-Ph*), 129.4, 131.2 and 133.0 (*o,m,p-Ph*).

HRMS: Calcd for $C_{13}H_{22}N_7^+$ [M-Cl$^-$] m/z: 276.1931. Found: 276.1937.

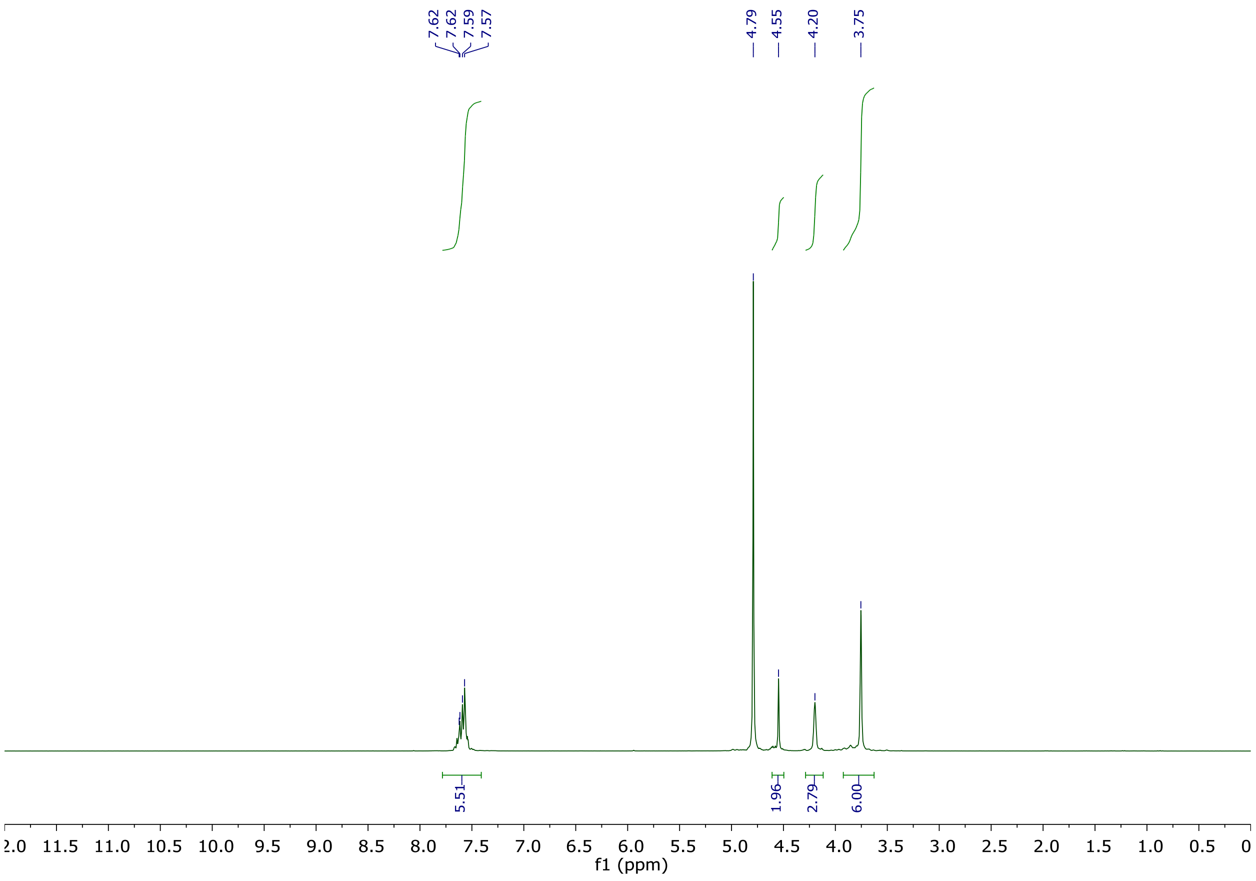

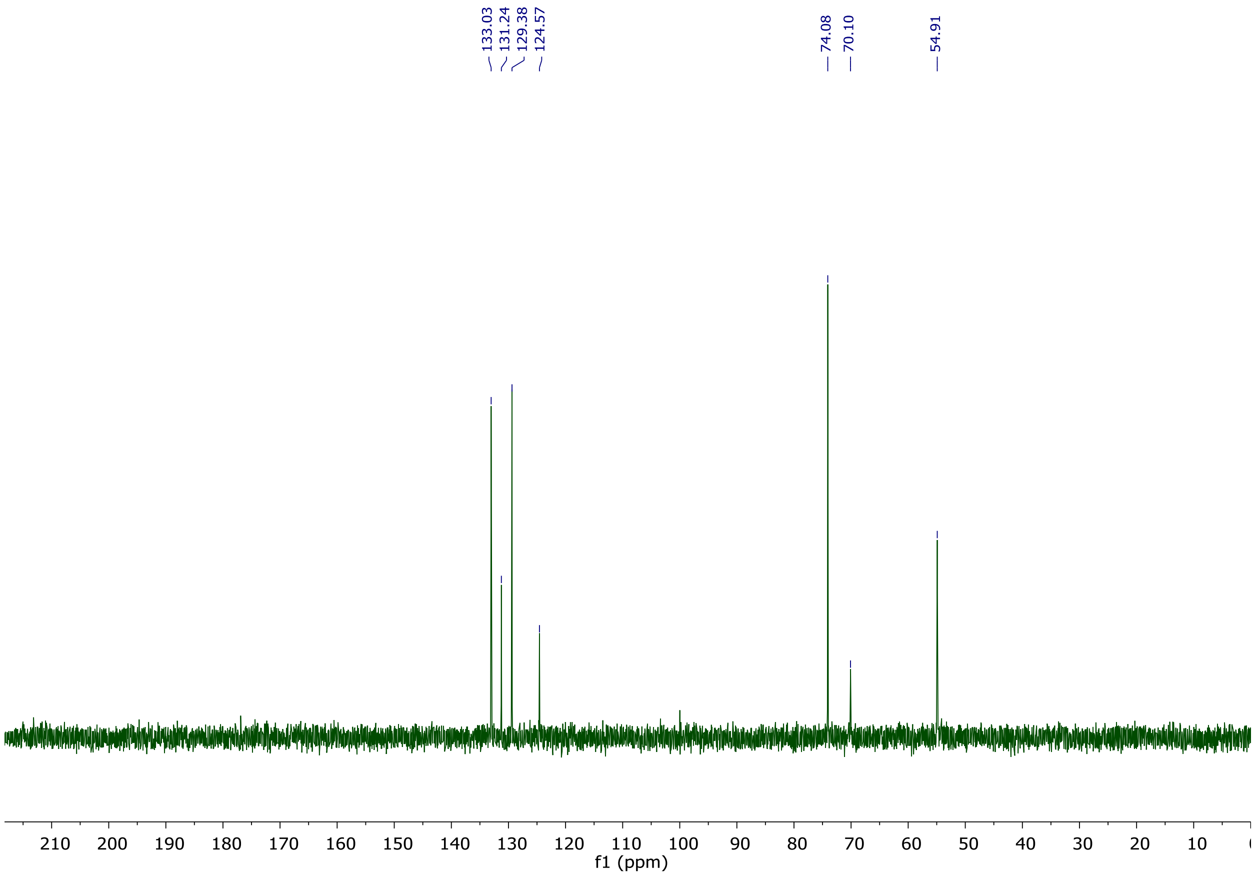
133.03
131.24
129.38
124.57
74.08
70.10
54.91
210 200 190 180 170 160 150 140 130 120 110 100 90 80 70 60 50 40 30 20 10
f1 (ppm)

Compound 19c·3HCl·2/3 H_2O

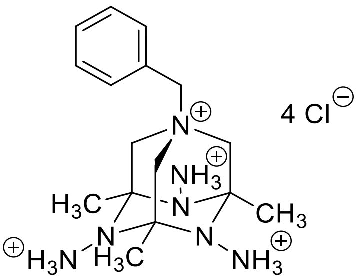

White solid, mp = 123-133 °C (with dec.)

^{1}H NMR (300 MHz, D_2O): δ = 1.53 (s, 9 H, 3 C*H*$_3$), 3.76 (s, 6 H, 3 C*H*$_2$), 4.59 (s, 2 H, PhC*H*$_2$), 7.5-7.6 (m, 5 H, *Ph*).

^{13}C NMR (75 MHz, D_2O): δ = 20.3 (3 C*H*$_3$), 58.2 (3 C*H*$_2$), 70.1 (PhC*H*$_2$), 71.4 (3 N*C*N), 123.8 (*i-Ph*), 129.5, 131.4 and 133.1 (*o*,*m*,*p-Ph*).

HRMS: Calcd for $C_{16}H_{28}N_7^+$ [M-3H$^+$-4Cl$^-$] m/z: 318.2401. Found: 318.2397.

For $C_{16}H_{31}Cl_4N_7$·2/3H_2O calcd: C 40.43%, H 6.86%, N 20.63%. Found: C 40.78%, H 6.87%, N 20.07%.

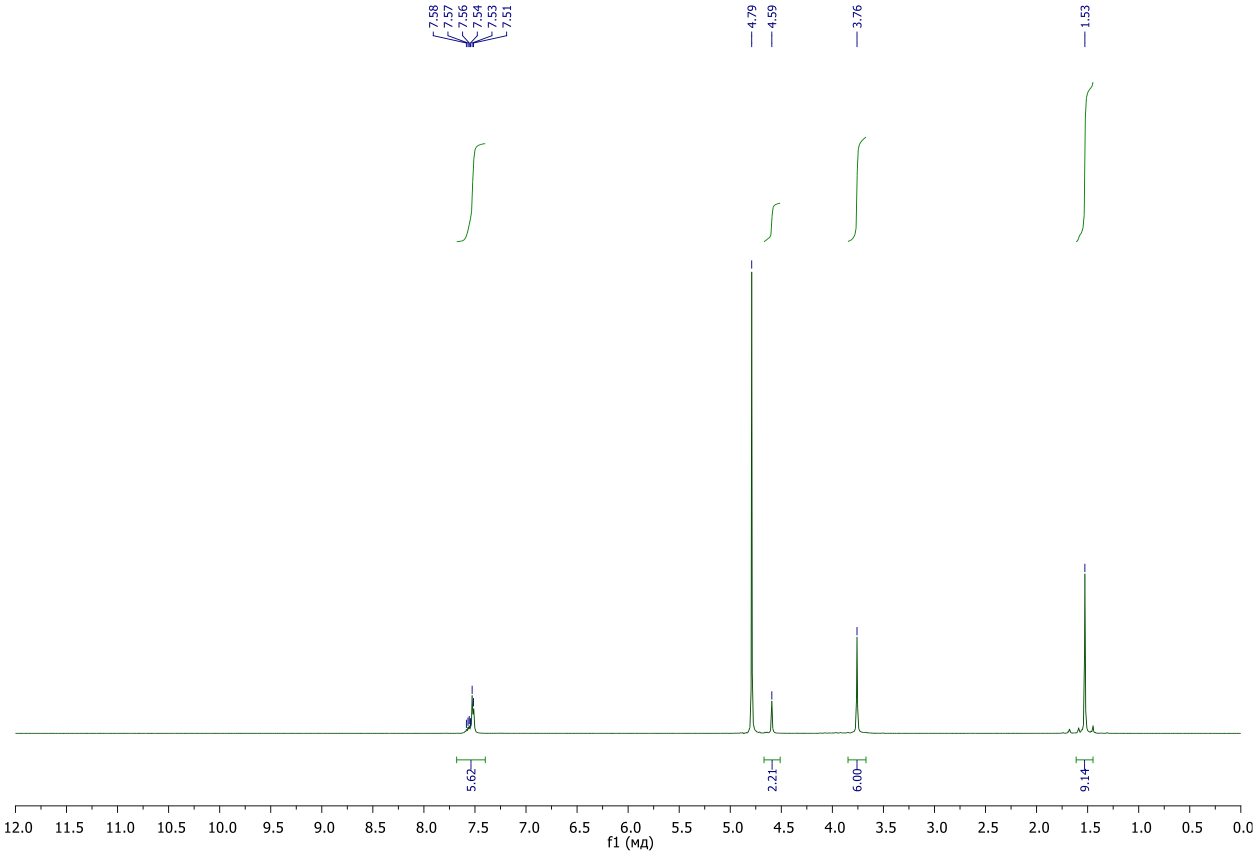

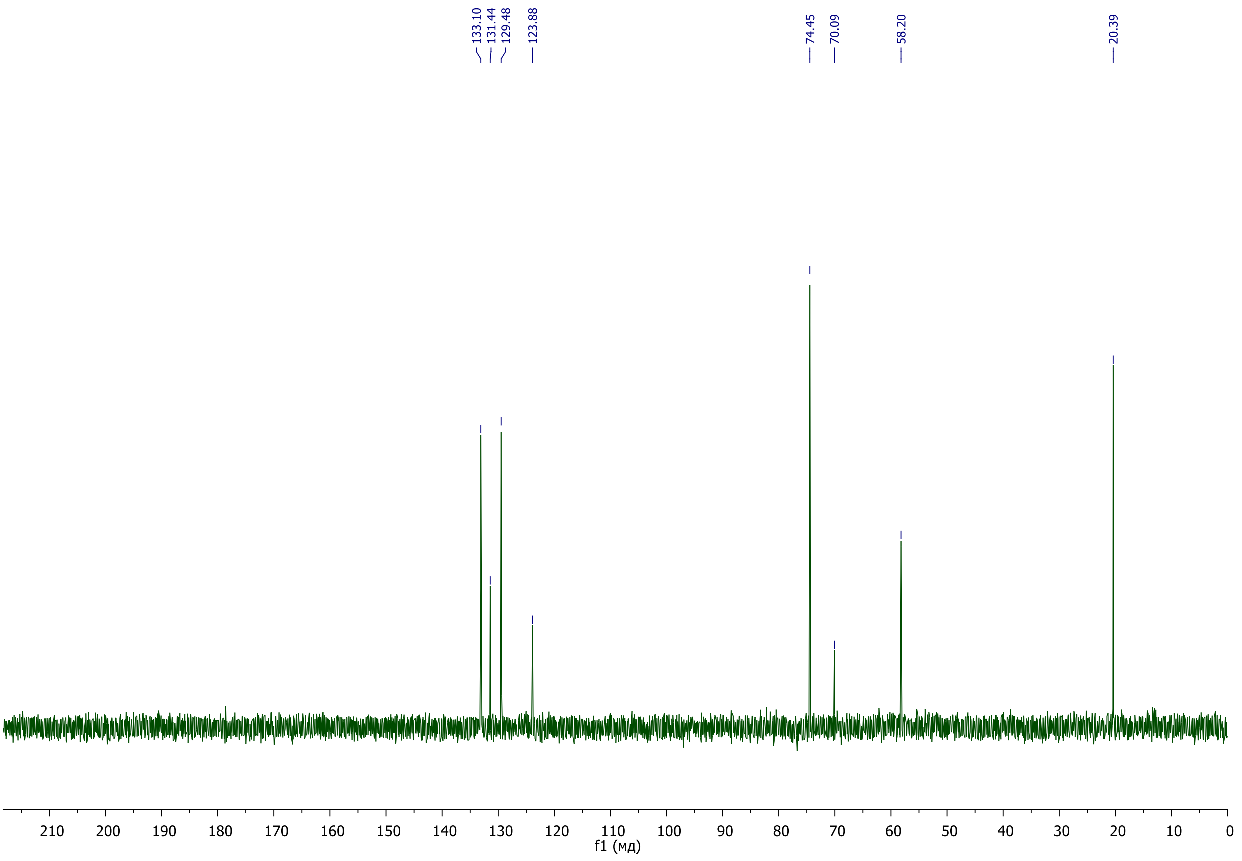
133.10
131.44
129.48
123.88
74.45
70.09
58.20
20.39
f1 (мд)

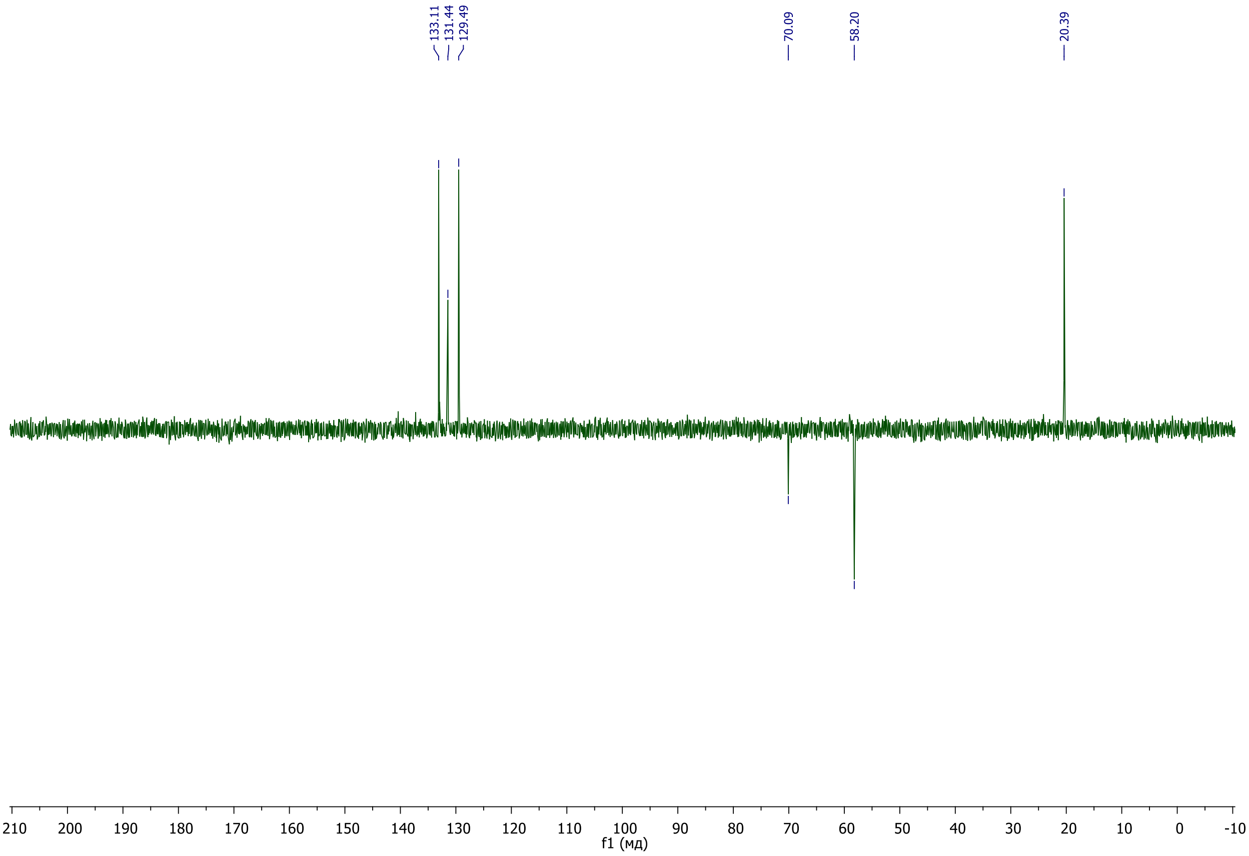
133.11
131.44
129.49
70.09
58.20
20.39
f1 (мд)

HSQC

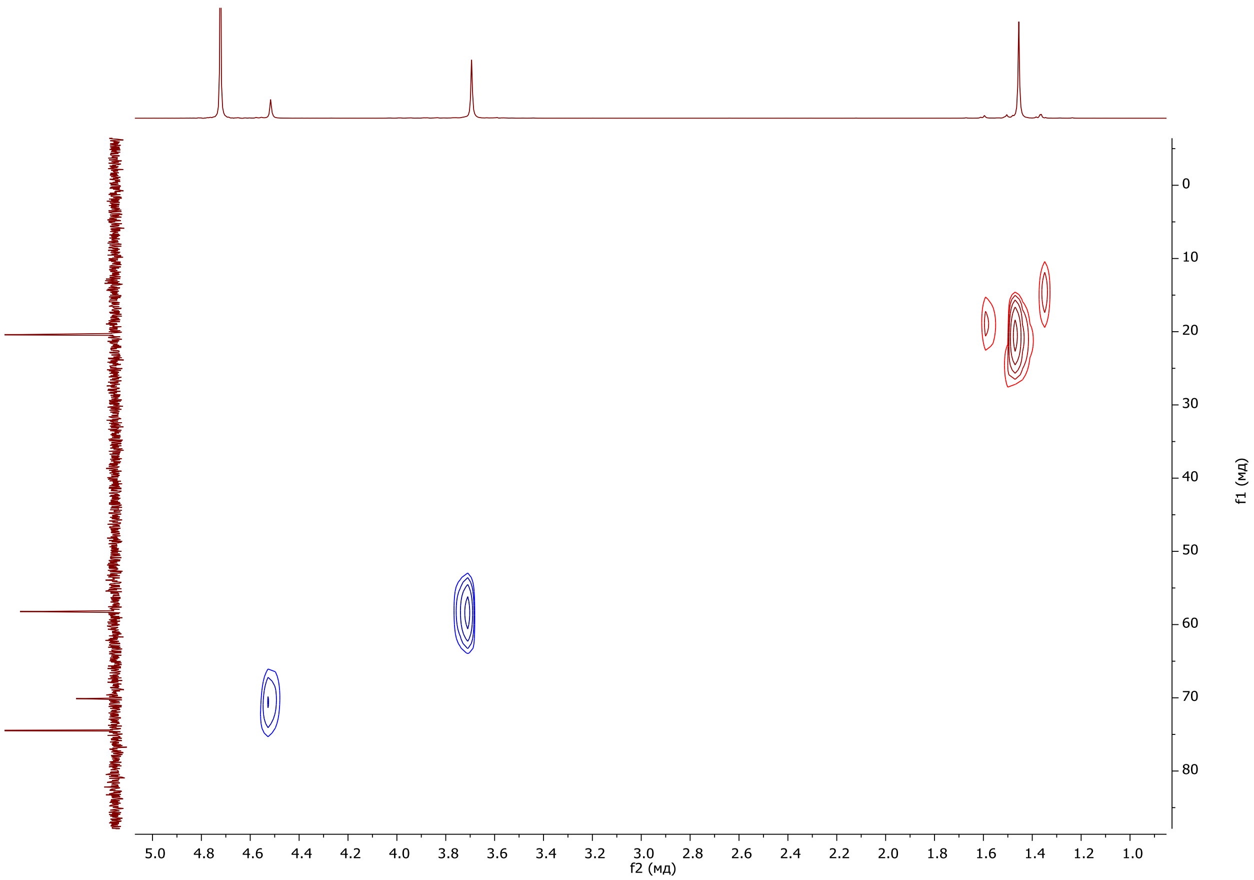

5.0
4.8
4.6
4.4
4.2
4.0
3.8
3.6
3.4
3.2
3.0
2.8
2.6
2.4
2.2
2.0
1.8
1.6
1.4
1.2
1.0
f2 (мд)
0
10
20
30
40
50
60
70
80
f1 (мд)

Compound 19e·3HCl·1.5 H_2O

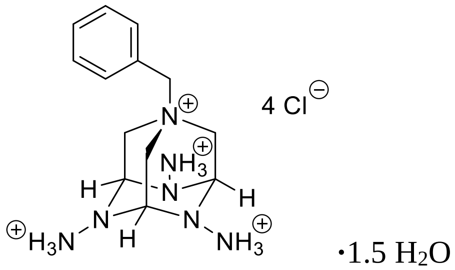

White solid, dec. at 155-165 °C without melting.

^{1}H NMR (300 MHz, D_2O): δ = 4.02 (s, 6 H, 3 C*H*$_2$), 4.65 (s, 2 H, PhC*H*$_2$), 4.73 (s, 3 H, 3 C*H*), 7.5-7.6 (m, 5 H, *Ph*).

^{13}C NMR (75 MHz, D_2O): δ = 54.9 (3 C*H*$_2$), 70.4 (PhC*H*$_2$), 70.9 (3 *C*H), 123.9 (*i-Ph*), 129.5, 131.5 and 133.0 (*o,m,p-Ph*).

HRMS: Calcd for $C_{13}H_{22}N_7$ [M-3H$^+$-4Cl$^-$] m/z: 276.1931. Found: 276.1935.

For $C_{13}H_{25}Cl_4N_7$•1.5 H_2O calcd: C 34.84%, H 6.30%, N 21.88%. Found: C 34.59%, H 6.01%, N 21.75%.

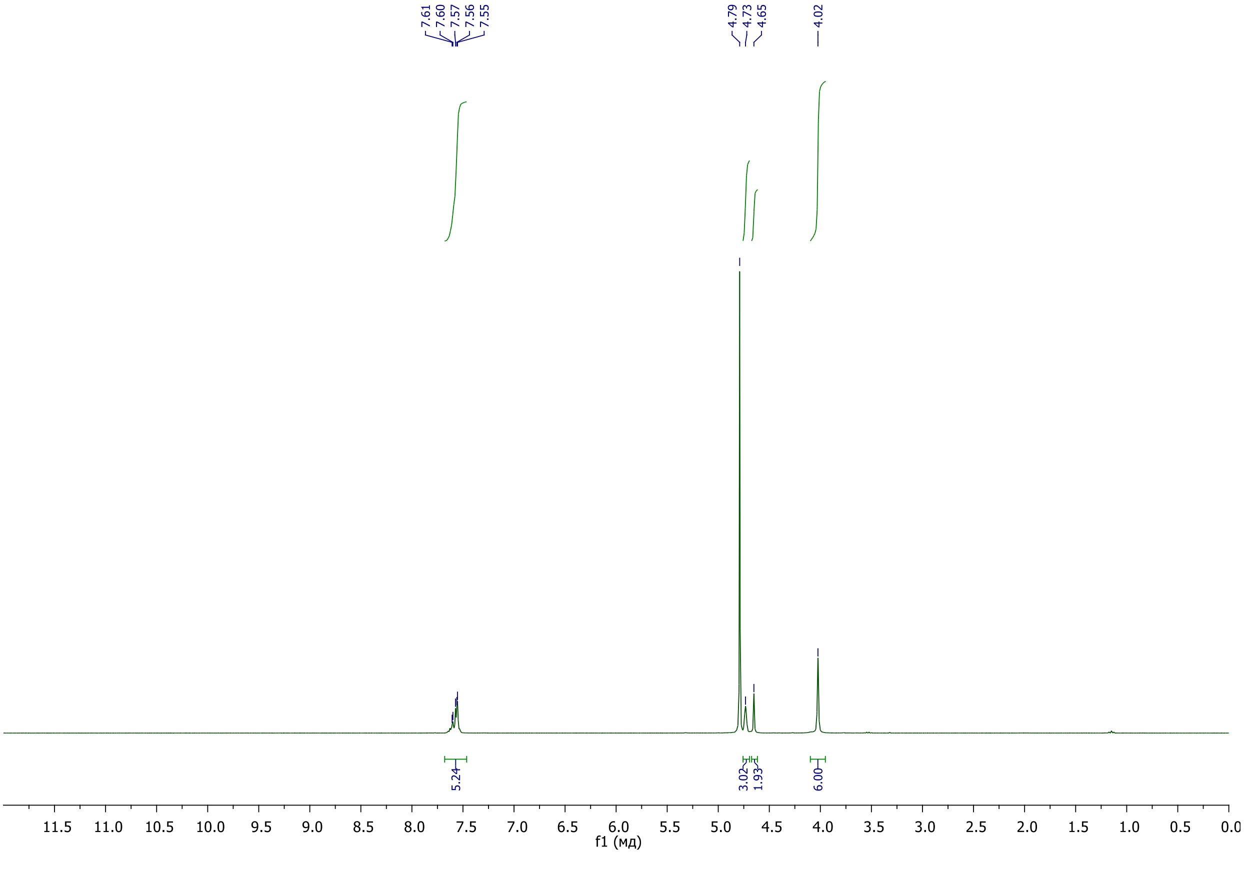

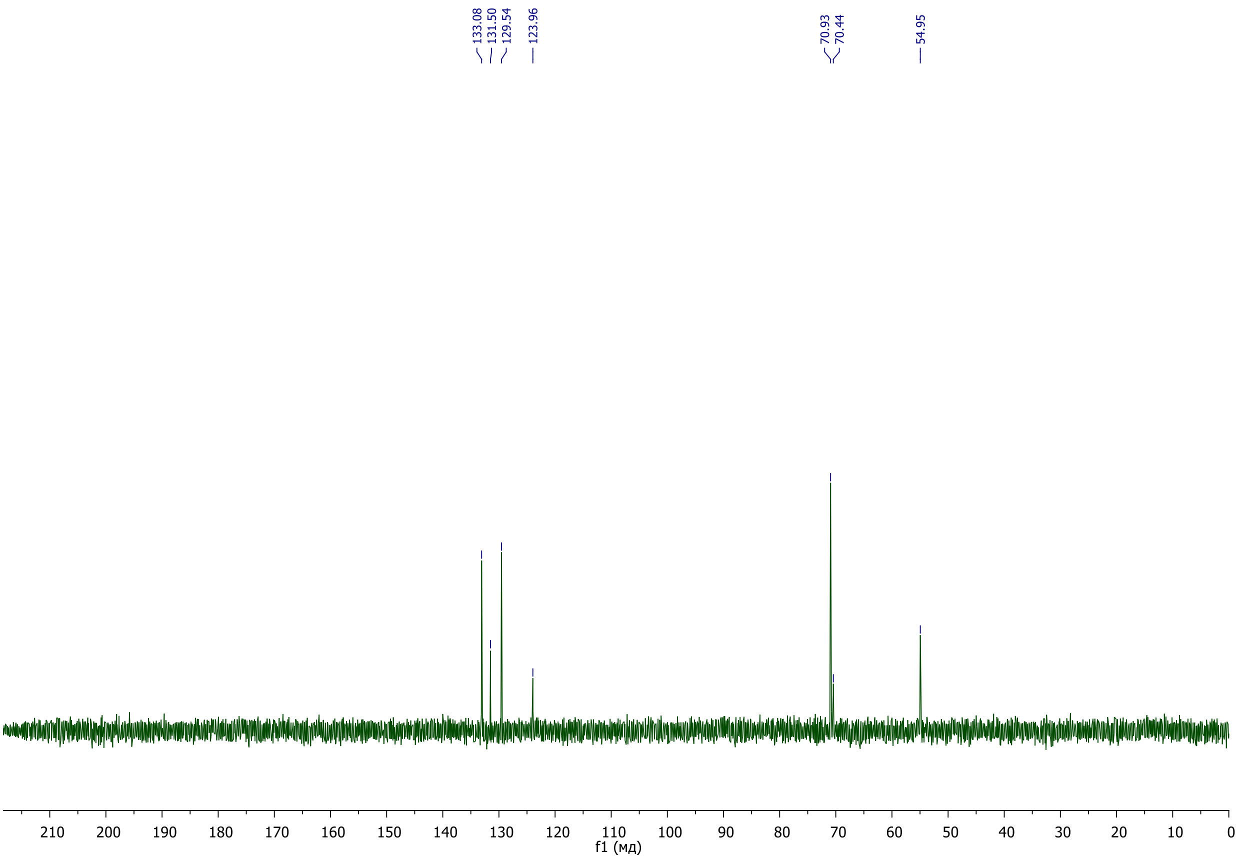
133.08
131.50
129.54
123.96
70.93
70.44
54.95
210 200 190 180 170 160 150 140 130 120 110 100 90 80 70 60 50 40 30 20 10 0
f1 (мд)

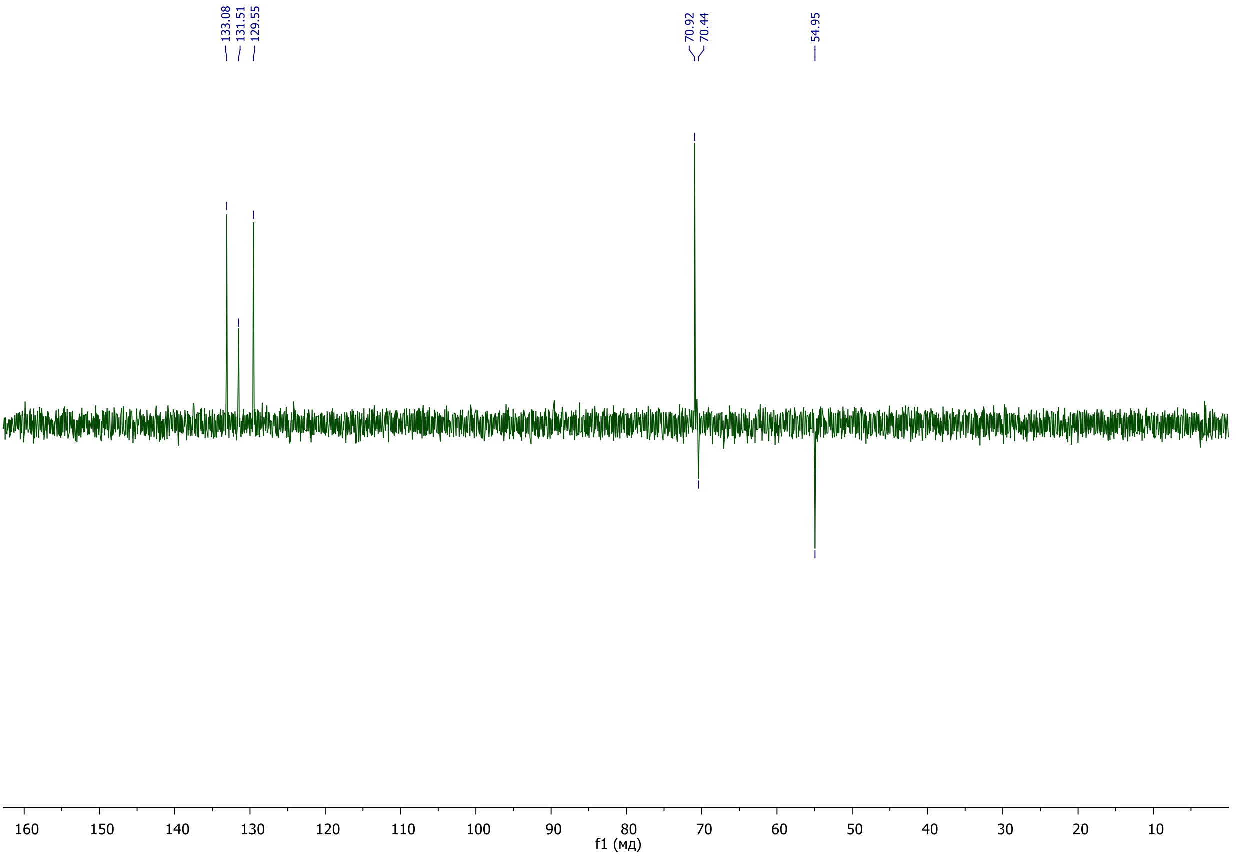
133.08
131.51
129.55
70.92
70.44
54.95
160 150 140 130 120 110 100 90 80 70 60 50 40 30 20 10
f1 (мд)

TAAD 20

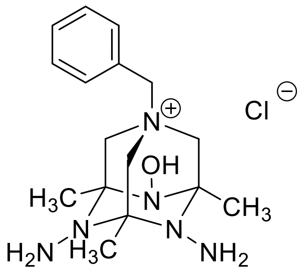

Pale yellow solid, mp = 157-165°C.

^{1}H NMR (300 MHz, D_2O): δ = 1.41 (s, 6 H, 2 C*H₃*), 1.44 (s, 3 H, C*H₃*), 3.43, 3.54 and 3.69 (3 br, 6 H, 2 C*H₂* and C*H₂*), 4.54 (s, 2 H, PhC*H₂*), 7.5-7.7 (m, 5 H, *Ph*).

^{13}C NMR (75 MHz, D_2O): δ = 20.5 (2 *C*H₃), 21.1 (*C*H₃), 53-56 and 58-60 (2 br, 2 *C*H₂ and *C*H₂), 69.9 (Ph*C*H₂), 73-75 and 75-77 (2 br, 2 N*C*N and N*C*N), 124.6 (*i-Ph*), 129.3, 131.2 and 133.0 (*o*,*m*,*p-Ph*).

HRMS: Calcd for $C_{16}H_{27}N_6O^+$ [M-Cl$^-$] m/z: 319.2241. Found: 319.2233.

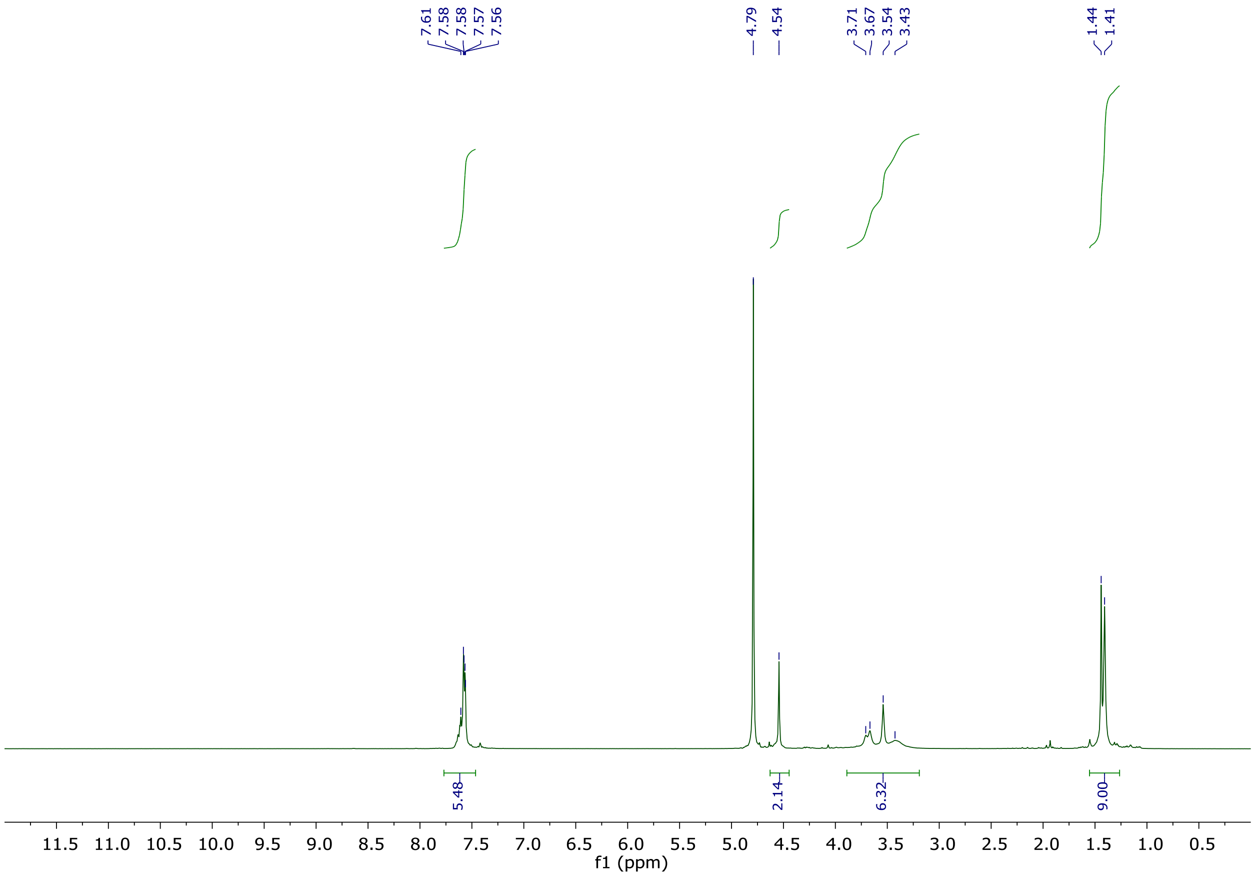

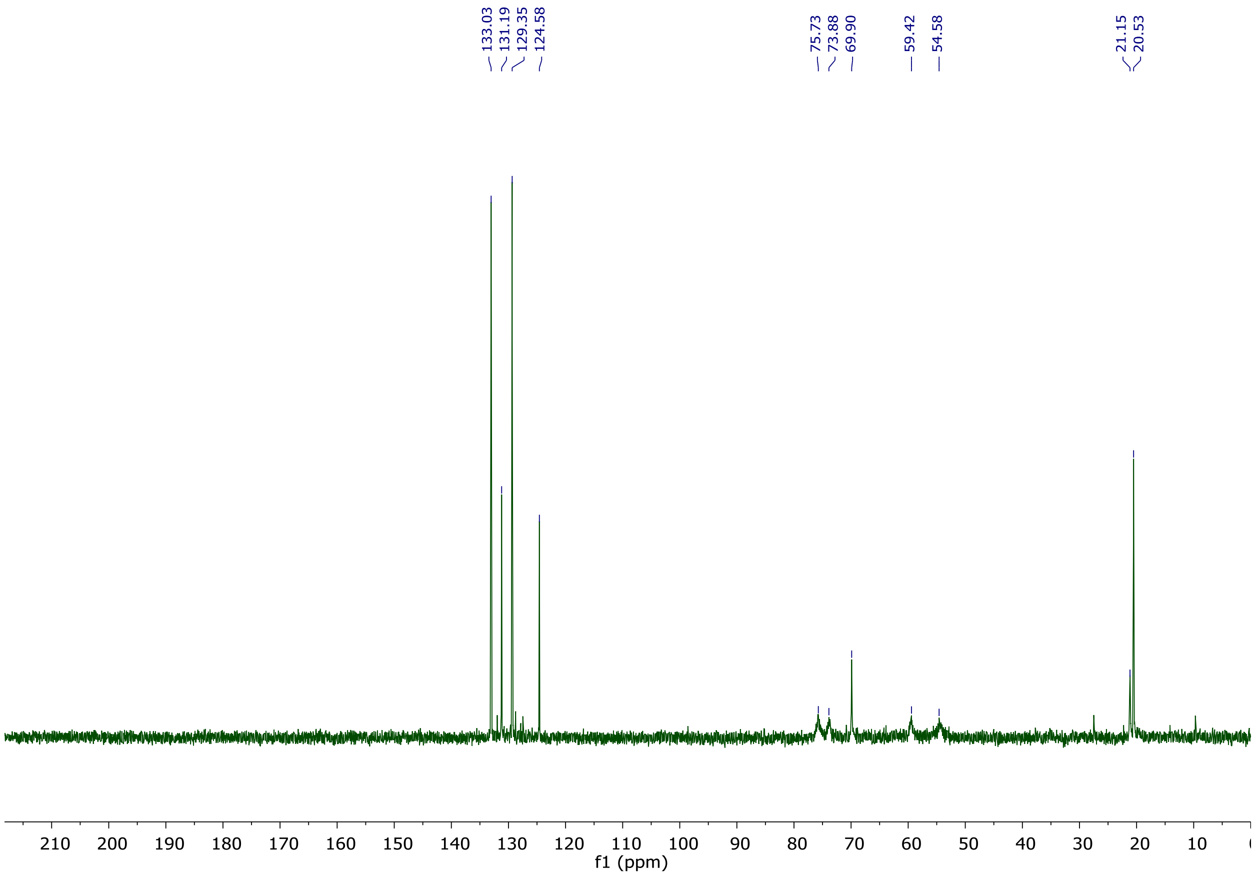
133.03
131.19
129.35
124.58
75.73
73.88
69.90
59.42
54.58
21.15
20.53
210 200 190 180 170 160 150 140 130 120 110 100 90 80 70 60 50 40 30 20 10
f1 (ppm)

Compound 20·2HCl·2H2O

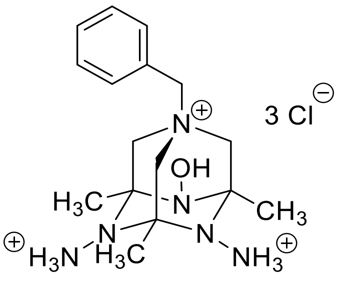

Pale yellow solid, mp = 145-150 °C (with dec.)

^{1}H NMR (300 MHz, D_2O): δ = 1.49 (s, 6 H, 2 C*H3*), 1.57 (s, 3 H, C*H3*), 3.4-3.6 (br, 6 H, 2 C*H2* and C*H2*), 4.58 (s, 2 H, PhC*H2*), 7.4-7.6 (m, 5 H, *Ph*).

^{13}C NMR (75 MHz, D_2O): δ = 19.8 (2 *C*H3), 20.4 (*C*H3), 55-65 (br, 2 *C*H2 and *C*H2), 69.7 (Ph*C*H2), 73.8 and 74.8 (2 N*C*N and N*C*N), 124.1 (*i-Ph*), 129.4, 131.4 and 133.0 (*o,m,p-Ph*).

HRMS: Calcd for $C_{16}H_{27}N_6O^+$ [M-2H$^+$-3Cl$^-$] m/z: 319.2241. Found: 319.2238.

For $C_{16}H_{29}Cl_3N_6O$·$2H_2O$ calcd: C 41.43%, H 7.17%, N 18.12%. Found: C 41.82%, H 6.93%, N 17.68%.

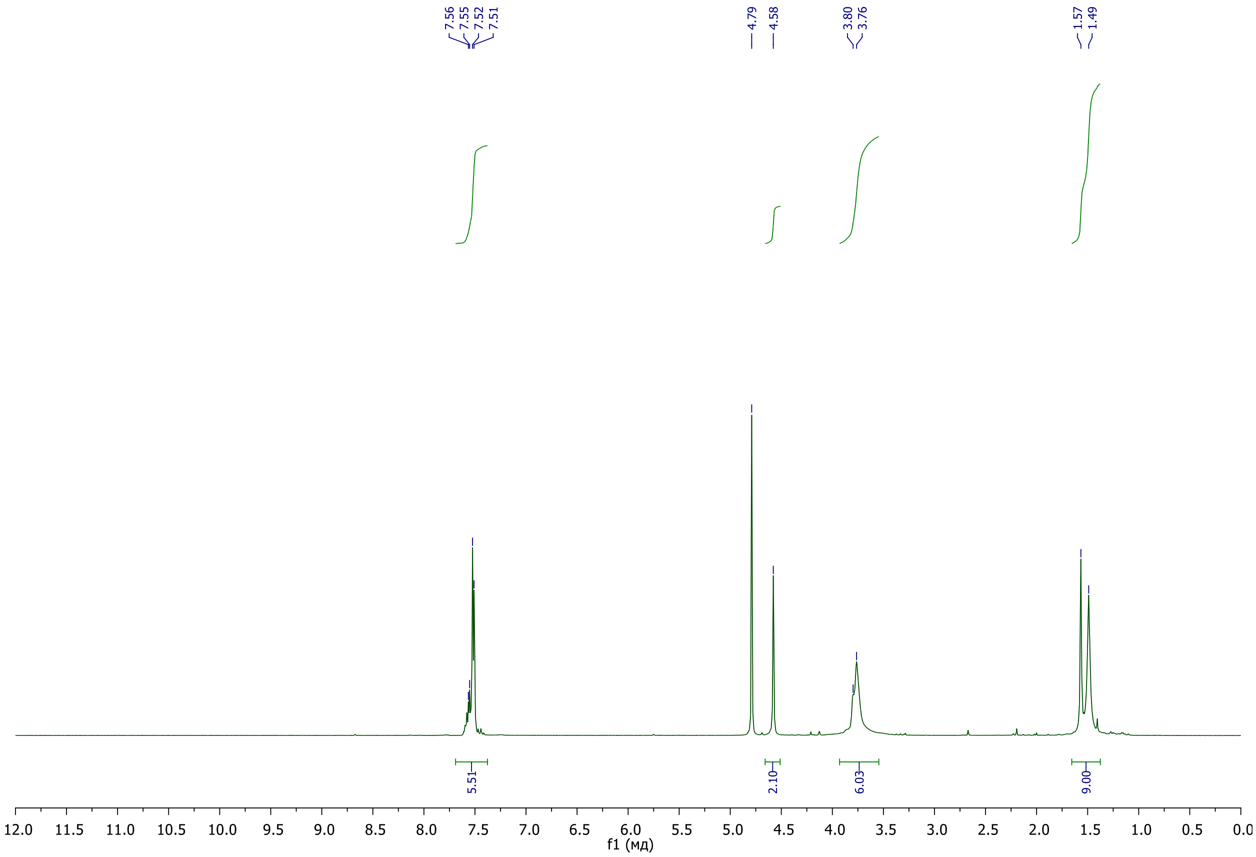

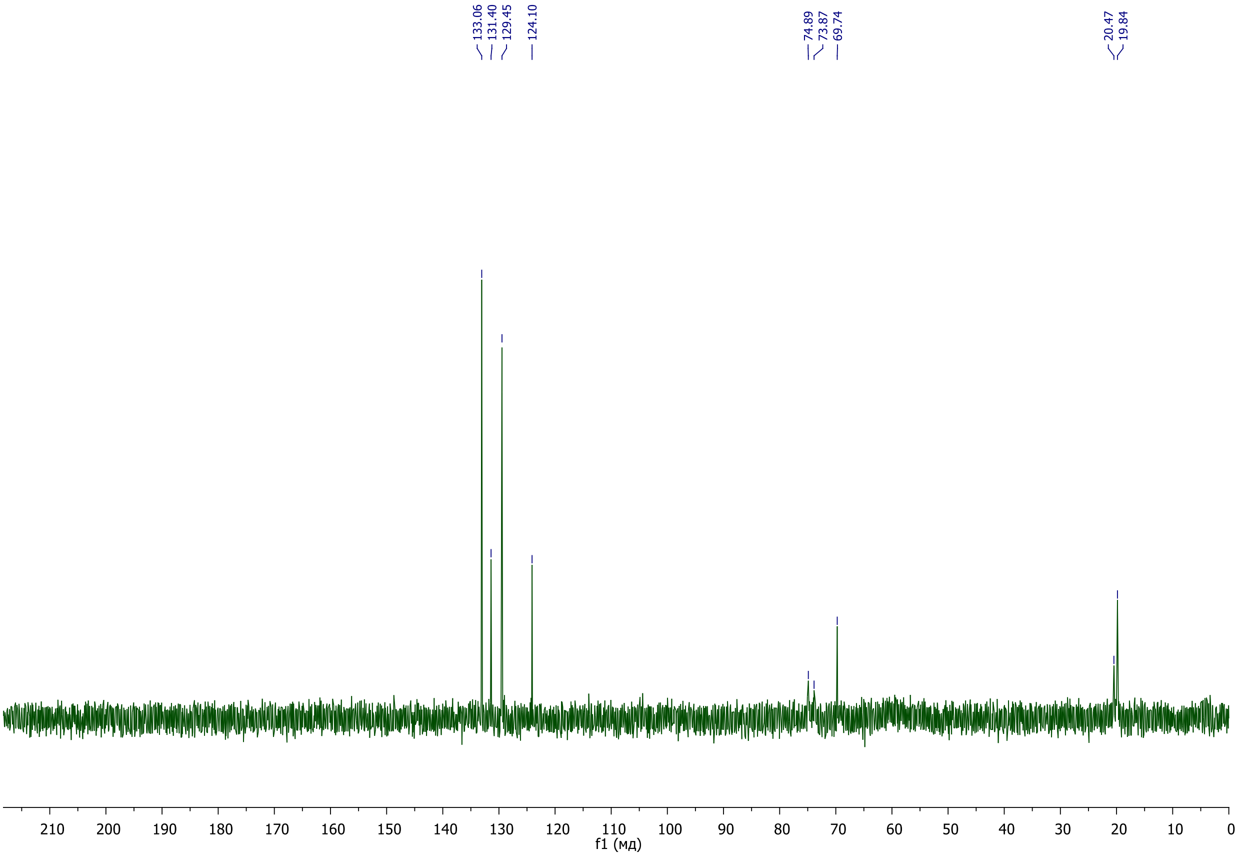
133.06
131.40
129.45
124.10
74.89
73.87
69.74
20.47
19.84
210 200 190 180 170 160 150 140 130 120 110 100 90 80 70 60 50 40 30 20 10 0
f1 (мд)

TAAD 21

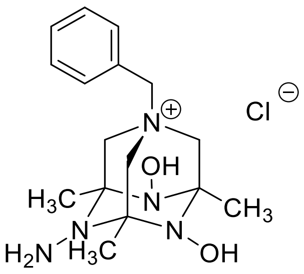

White solid, dec. >200 °C without melting.

^{1}H NMR (300 MHz, D_2O): δ = 1.39 (s, 3 H, C*H*$_3$), 1.42 (s, 6 H, 2 C*H*$_3$), 3.63 and 3.75 (2 d, *J* = 12 Hz, 4 H, 2 C*H*$_2$), 3.56 (s, 2 H, C*H*$_2$), 4.54 (s, 2 H, PhC*H*$_2$), 7.5-7.7 (m, 5 H, *Ph*).

^{13}C NMR (75 MHz, D_2O): δ = 19.8 (2 *C*H$_3$), 20.3 (*C*H$_3$), 55-60 (br, 2 *C*H$_2$ and *C*H$_2$) 69.7 (Ph*C*H$_2$), 74.4 and 77.0 (2 br, 2 N*C*N and N*C*N), 124.6 (*i-Ph*) 129.3, 131.3 and 133.0 (*o,m,p-Ph*). HRMS: Calcd for $C_{16}H_{26}N_5O_2^+$ [M-Cl$^-$] m/z: 320.2081. Found: 320.2088.

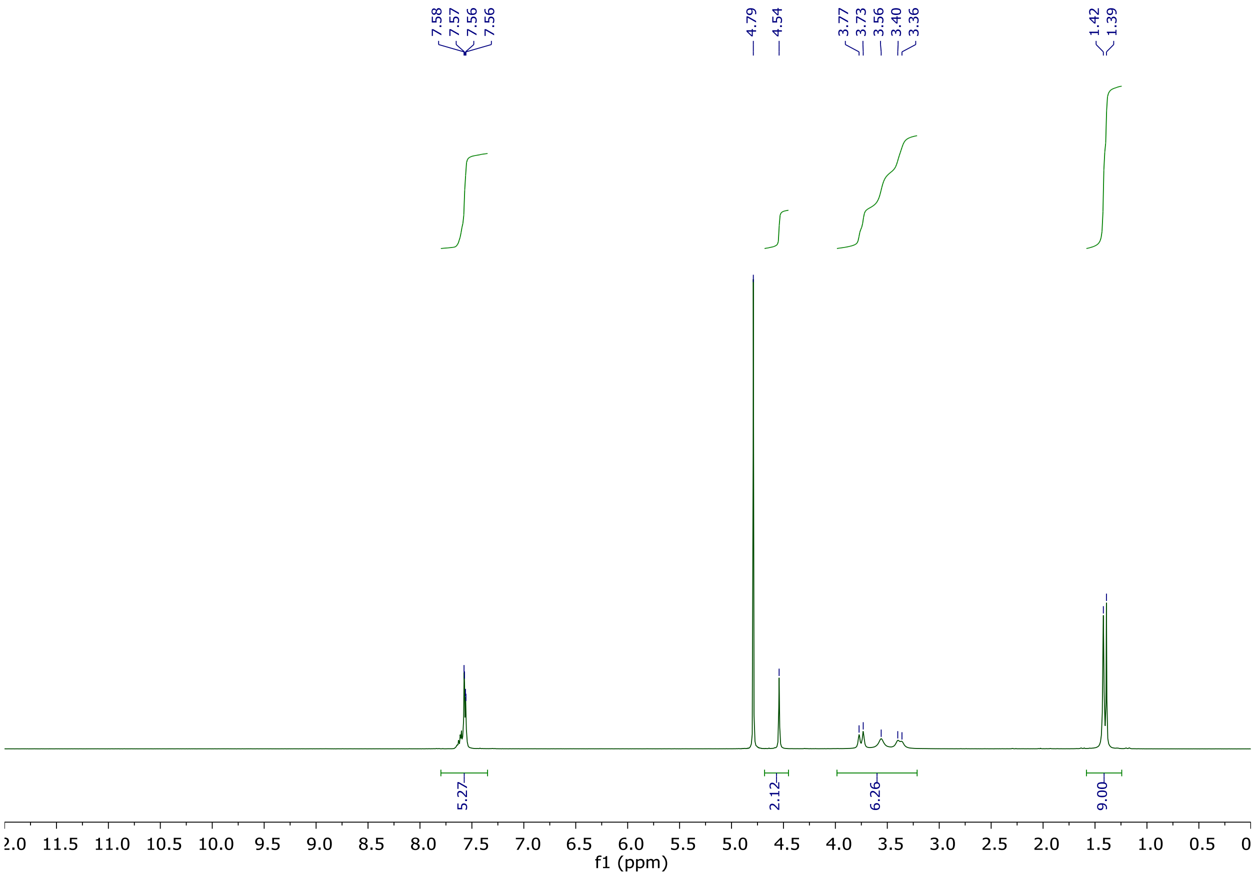

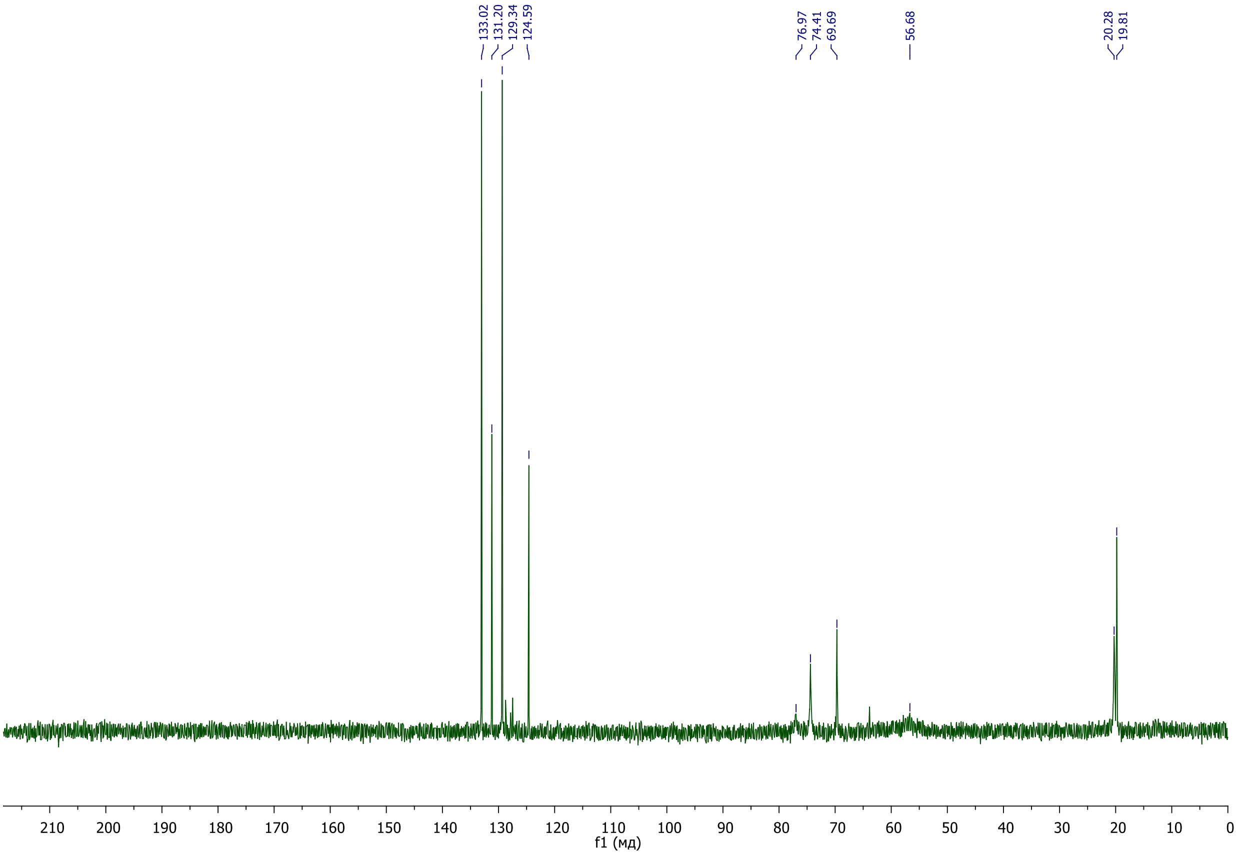
133.02
131.20
129.34
124.59
76.97
74.41
69.69
56.68
20.28
19.81
210 200 190 180 170 160 150 140 130 120 110 100 90 80 70 60 50 40 30 20 10 0
f1 (мд)

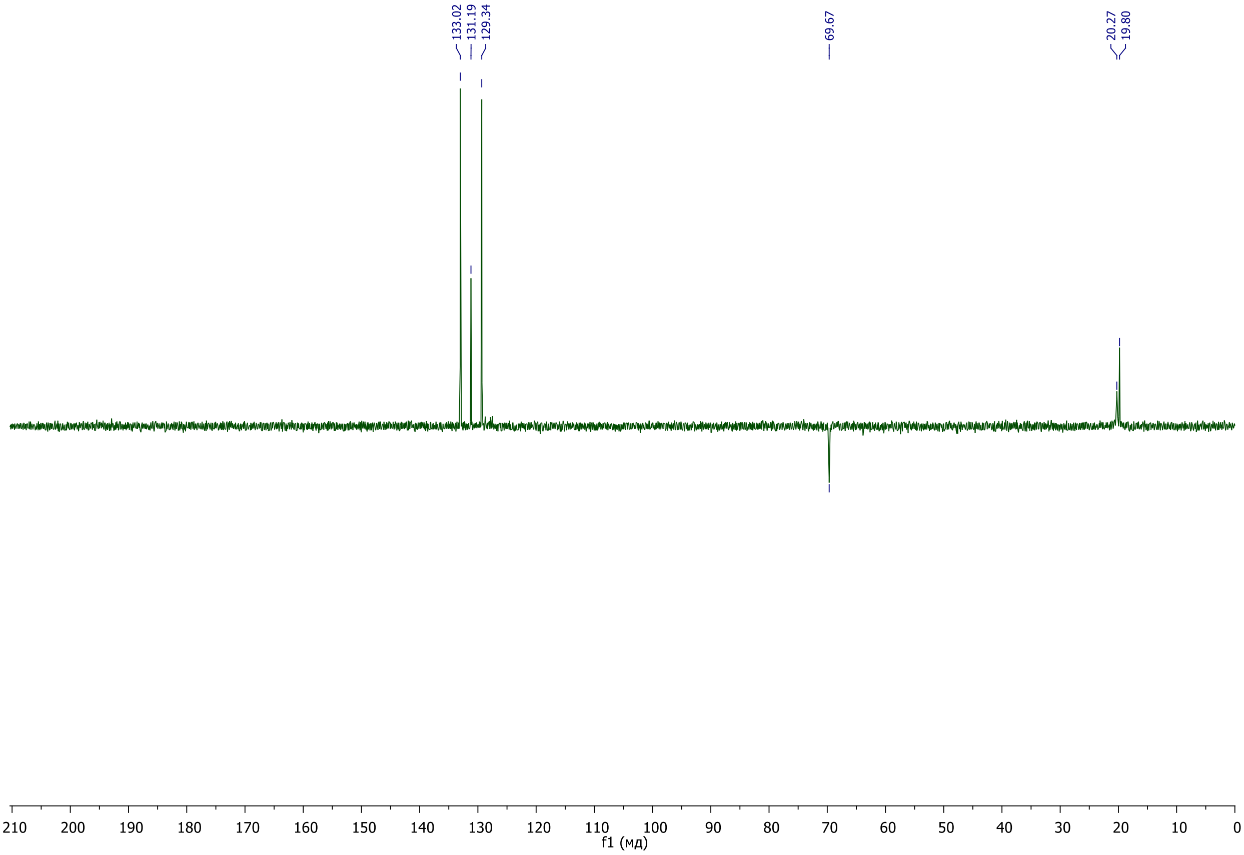
133.02
131.19
129.34
69.67
20.27
19.80
210 200 190 180 170 160 150 140 130 120 110 100 90 80 70 60 50 40 30 20 10 0
f1 (мд)

Compound 21·HCl·2H_2O

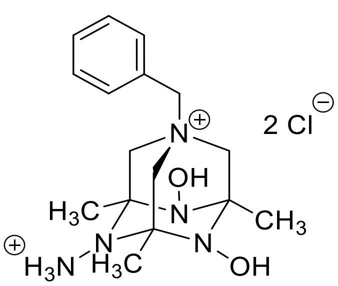

•2H_2O

White solid, mp = 170-174 °C with dec.

^1H NMR (300 MHz, D_2O): δ = 1.43 (s, 3 H, C*H*$_3$), 1.54 (s, 6 H, 2 C*H*$_3$), 3.67 and 3.84 (2 m, 6 H, 2 C*H*$_2$ and C*H*$_2$), 4.61 (s, 2 H, PhC*H*$_2$), 7.5-7.7 (m, 5 H, *Ph*).

^{13}C NMR (75 MHz, D_2O): δ = 19.7 (2 *C*H$_3$ and *C*H$_3$), 55-60 (br, 2 *C*H$_2$ and *C*H$_2$), 69.5 (Ph*C*H$_2$), 73.9 and 76.4 (2 N*C*N and N*C*N), 124.3 (*i-Ph*), 129.3, 131.3 and 133.0 (*o,m,p-Ph*).

HRMS: Calcd for $C_{16}H_{26}N_5O_2^+$ [M-H$^+$-2Cl$^-$] m/z: 320.2081. Found: 320.2082.

For $C_{16}H_{27}Cl_2N_5O_2$•2H_2O calcd: C 44.86%, H 7.29%, N 16.35%. Found: C 44.89%, H 7.36%, N 16.78%.

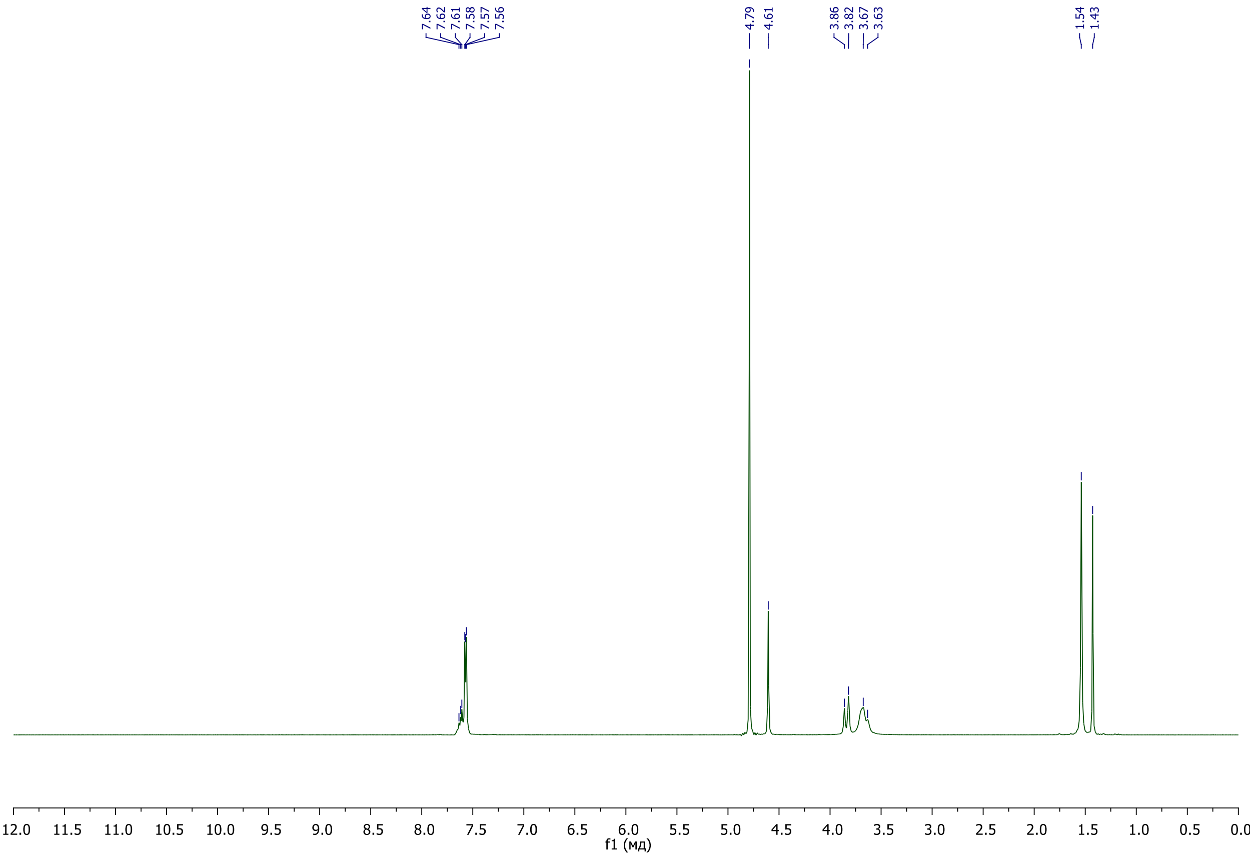

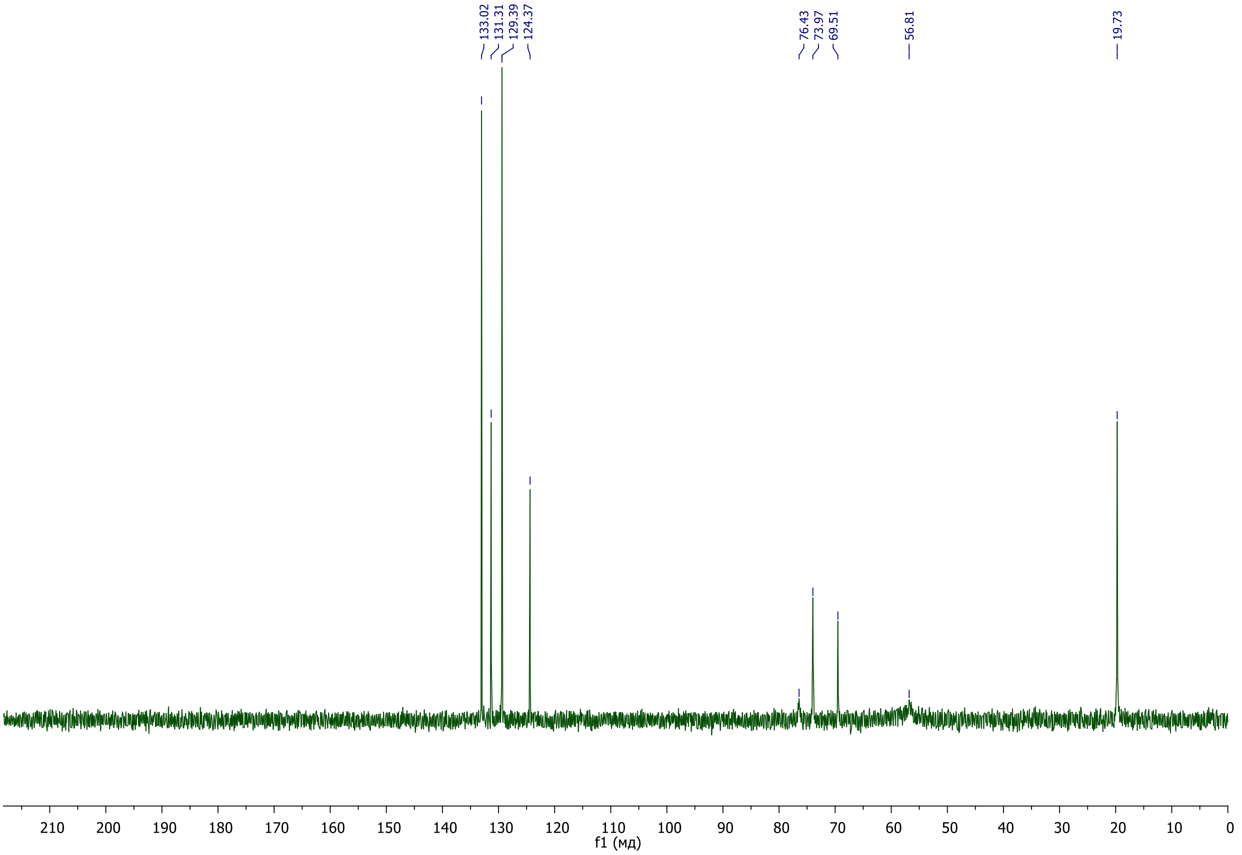
133.02
131.31
129.39
124.37
76.43
73.97
69.51
56.81
19.73
210 200 190 180 170 160 150 140 130 120 110 100 90 80 70 60 50 40 30 20 10 0
f1 (мд)

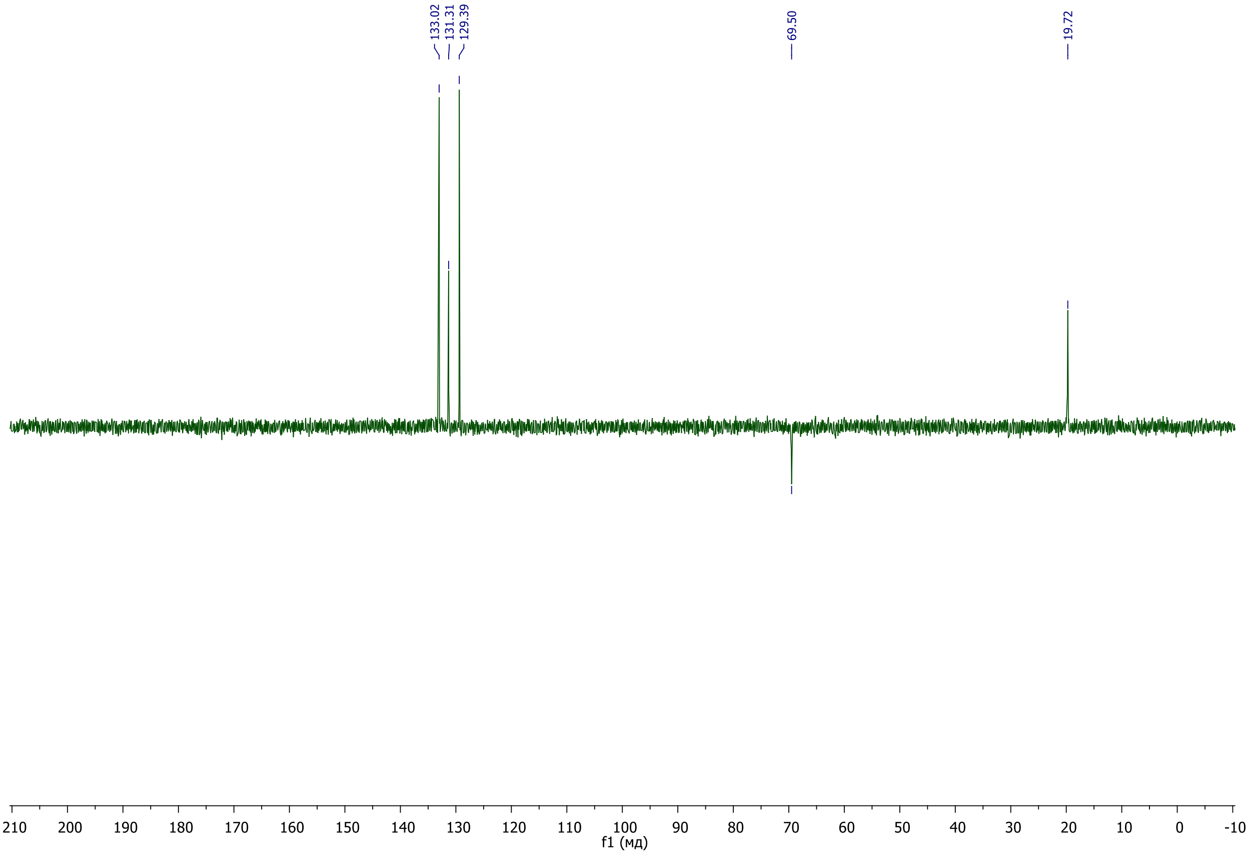
133.02
131.31
129.39
69.50
19.72
210 200 190 180 170 160 150 140 130 120 110 100 90 80 70 60 50 40 30 20 10 0 -10
f1 (мд)

HSQC

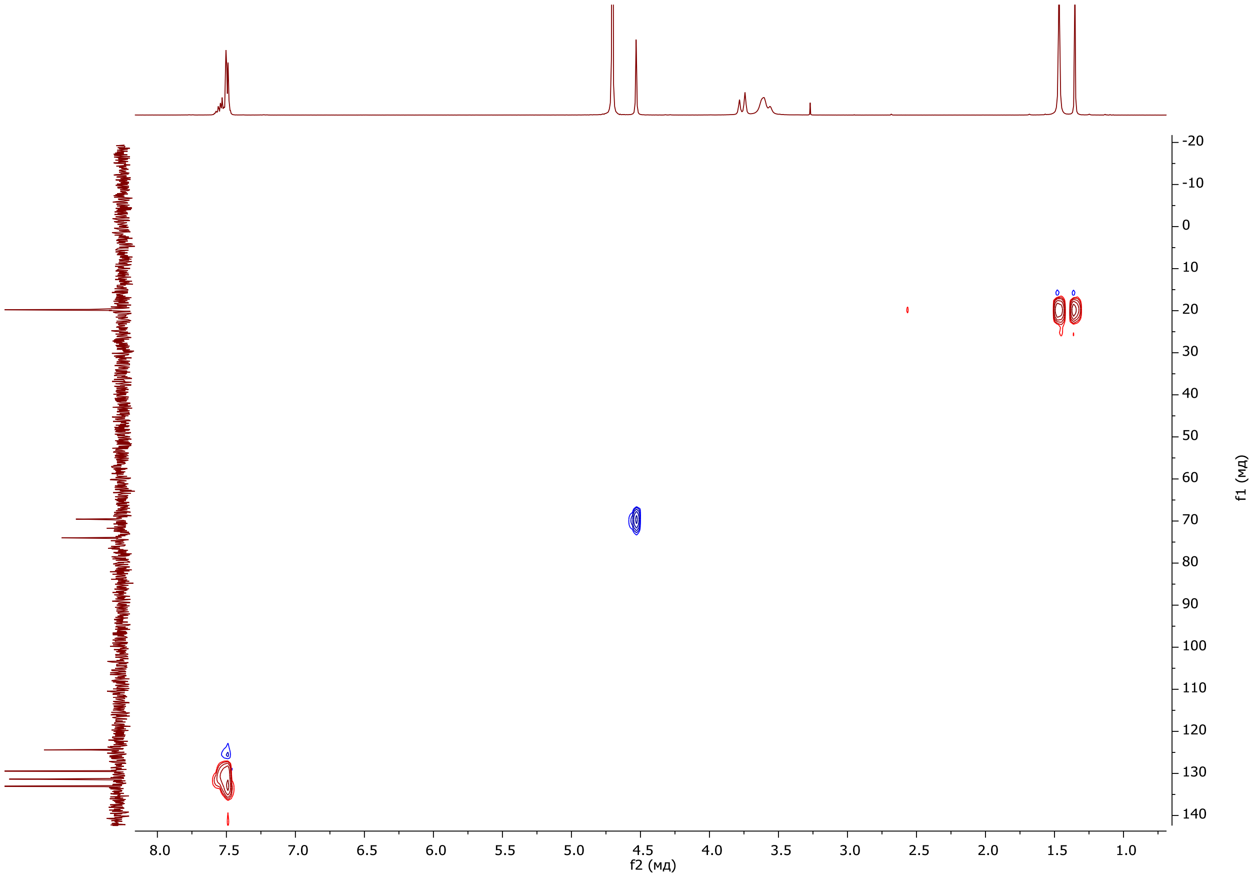

8.0
7.5
7.0
6.5
6.0
5.5
5.0
4.5
4.0
3.5
3.0
2.5
2.0
1.5
1.0
f2 (мд)
-20
-10
0
10
20
30
40
50
60
70
80
90
100
110
120
130
140
f1 (мд)

Ozatriazaadamantane 22

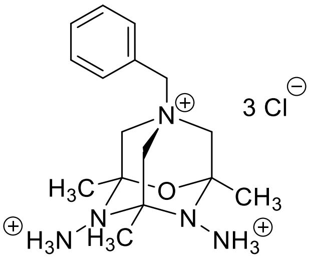

White solid, starts dec. at 115 °C

^{1}H NMR (300 MHz, D_2O): δ = 1.37 (s, 6 H, 2 C*H3*), 1.48 (s, 3 H, C*H3*), 3.59 and 3.76 (2 d, *J* = 12.8 Hz, 4 H, 2 C*H2*), 3.74 (s, 2 H, C*H2*), 4.51 (s, 2 H, PhC*H2*), 7.4-7.6 (m, 5 H, *Ph*).

^{13}C NMR (75 MHz, D_2O): δ = 20.2 (*C*H3), 21.5 (2 *C*H3), 59.0 and 59.2 (2 *C*H2 and *C*H2), 69.8 (Ph*C*H2), 73.9 (N*C*N), 85.8 (2 O*C*N), 123.7 (*i-Ph*), 129.4, 131.3 and 133.0 (*o,m,p-Ph*).

HRMS: Calcd for $C_{16}H_{26}N_5O^+$ [M-2H$^+$-3Cl$^-$)] m/z: 304.2132. Found: 304.2132.

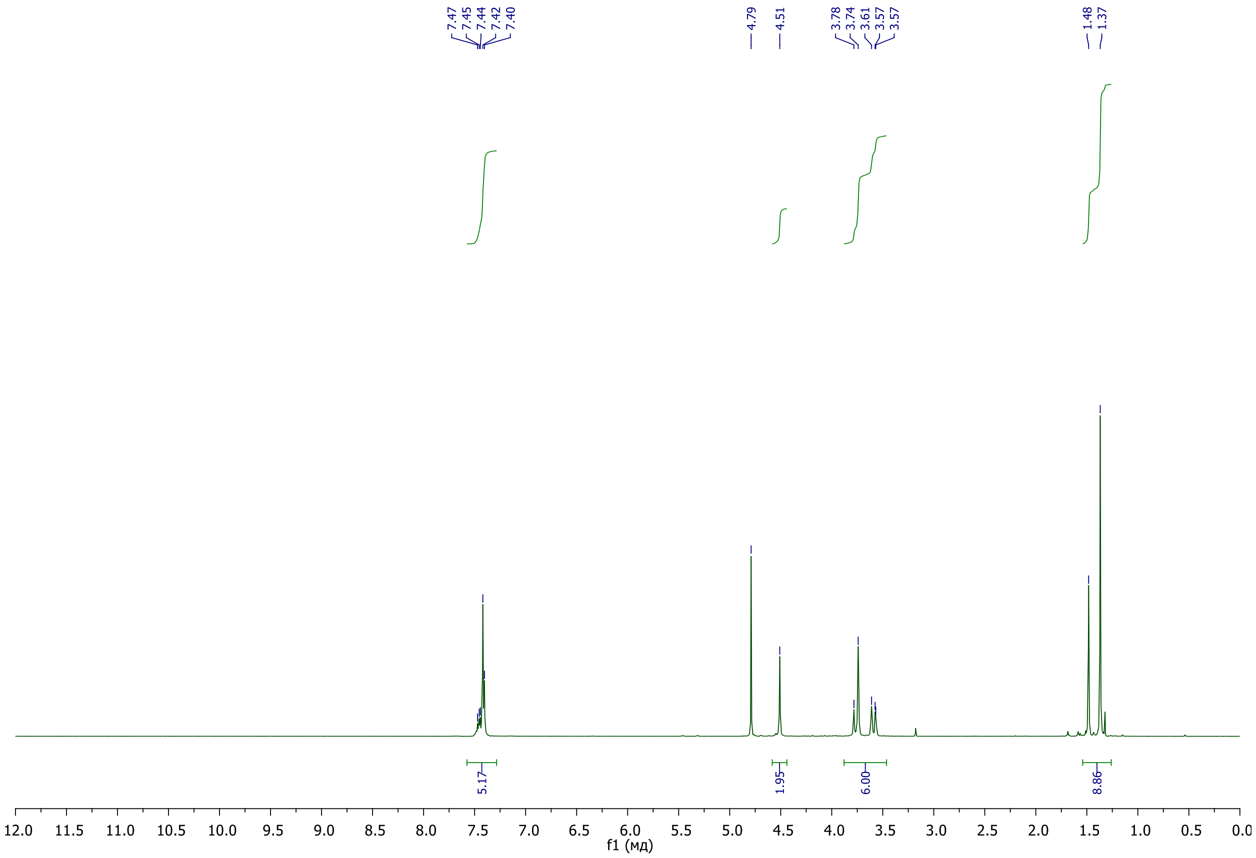

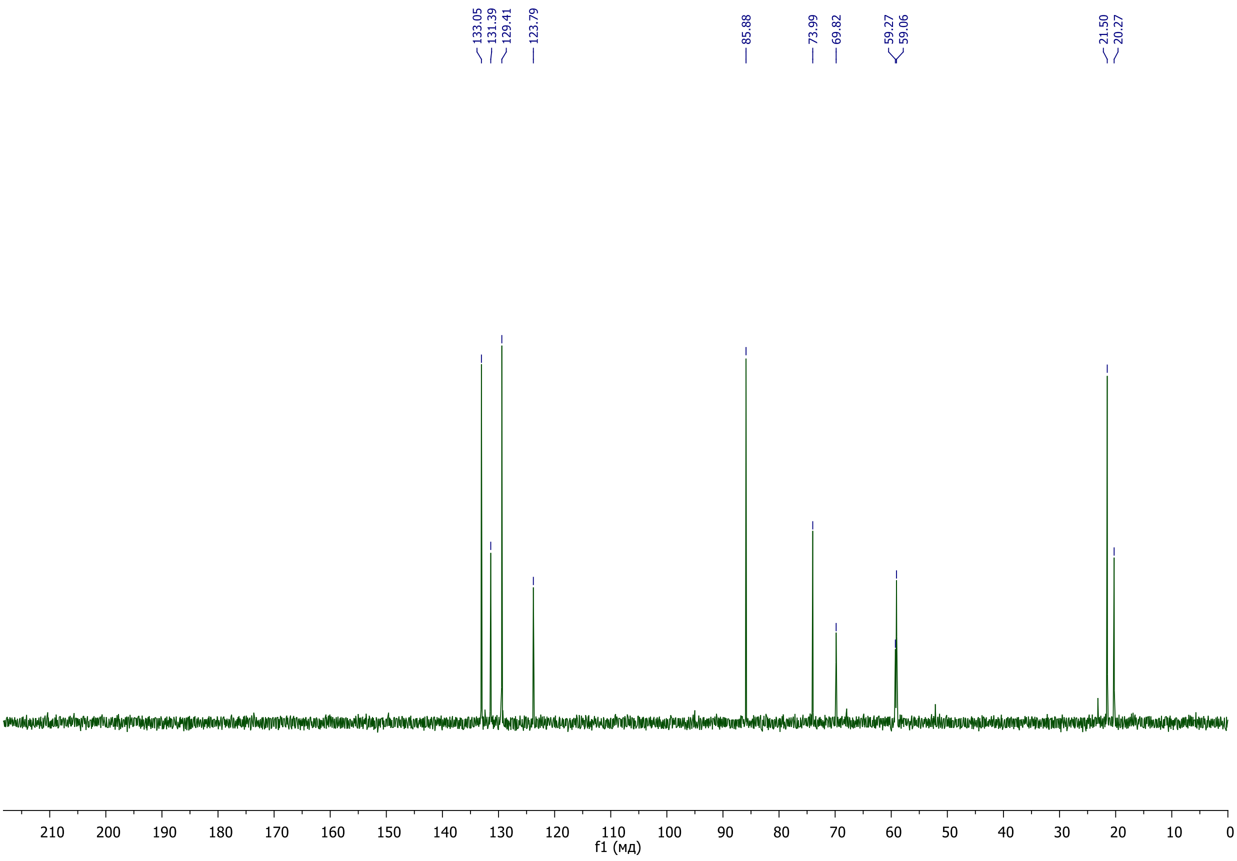

133.05
131.39
129.41
123.79
85.88
73.99
69.82
59.27
59.06
21.50
20.27
210 200 190 180 170 160 150 140 130 120 110 100 90 80 70 60 50 40 30 20 10 0
f1 (мд)

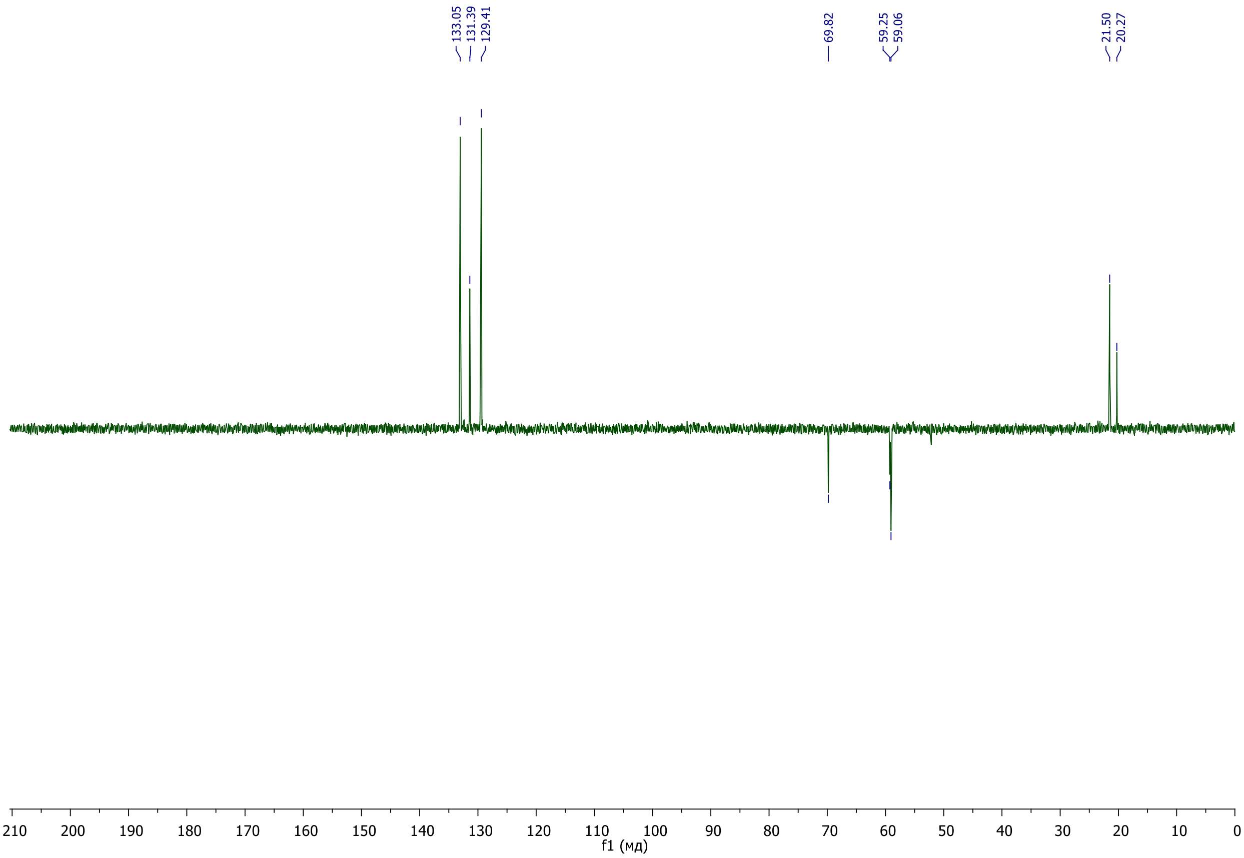

133.05
131.39
129.41
69.82
59.25
59.06
21.50
20.27
210 200 190 180 170 160 150 140 130 120 110 100 90 80 70 60 50 40 30 20 10 0
f1 (мд)

TAAD 23

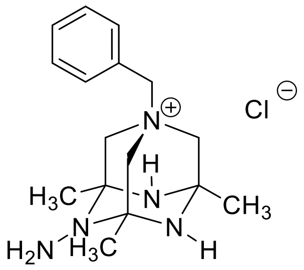

White solid, mp = 210-214 °C with dec.

^{1}H NMR (300 MHz, D_2O): δ = 1.26 (s, 6 H, 2 C*H*$_3$), 1.28 (s, 3 H, C*H*$_3$), 3.37 (s, 2 H, C*H*$_2$), 3.43 and 3.58 (2 d, *J* = 12 Hz, 4 H, 2 C*H*$_2$), 4.59 (s, 2 H, PhC*H*$_2$), 7.5-7.7 (m, 5 H, *Ph*).

^{13}C NMR (75 MHz, D_2O): δ = 22.5 (2 *C*H$_3$), 23.5 (*C*H$_3$), 62.5 (N*C*N), 62.9 (*C*H$_2$), 63.6 (2 *C*H$_2$), 69.2 (2 N*C*N), 70.0 (Ph*C*H$_2$), 124.6 (*i-Ph*), 129.3, 131.2 and 132.9 (*o*,*m*,*p-Ph*).

HRMS: Calcd for $C_{16}H_{26}N_5^{+}$ [M-Cl^{-}] m/z: 288.2188. Found: 288.2150.

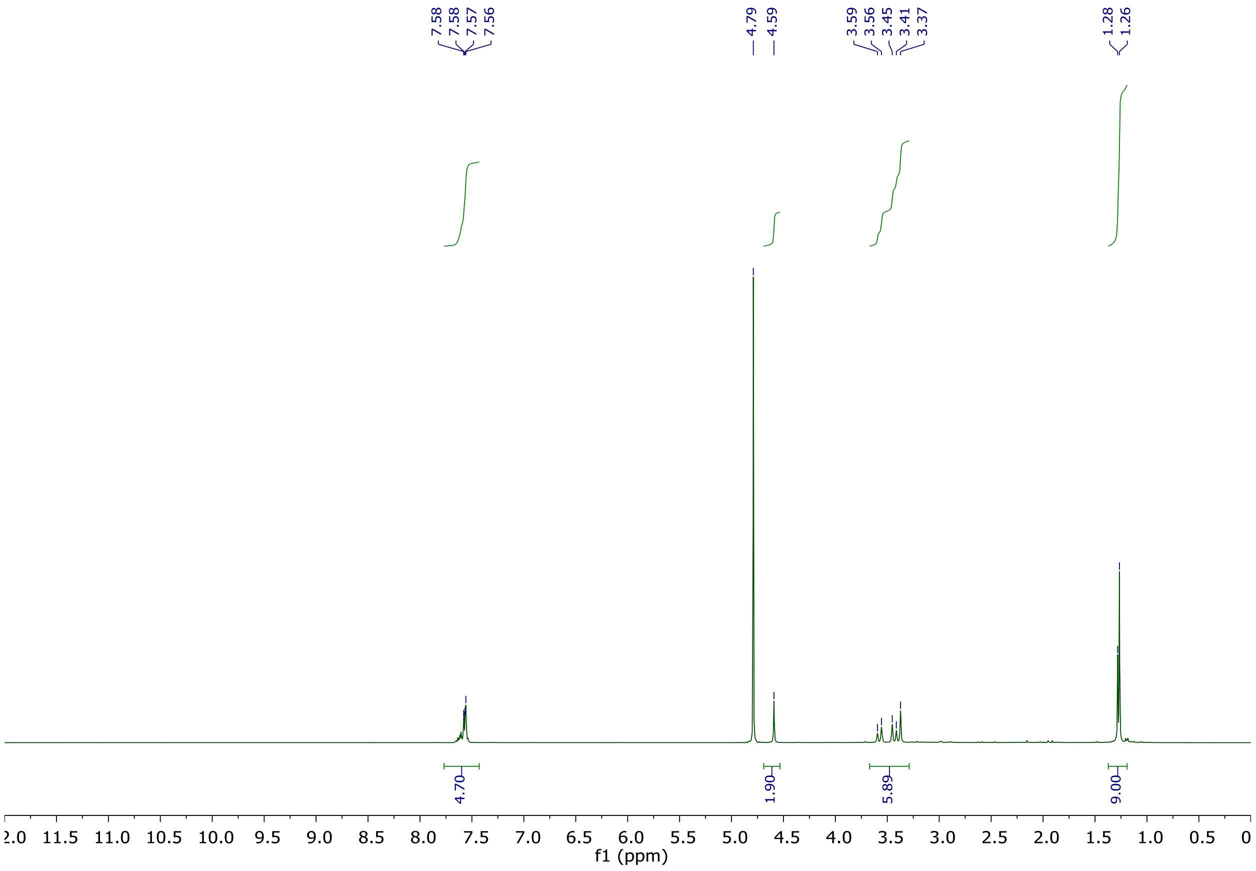

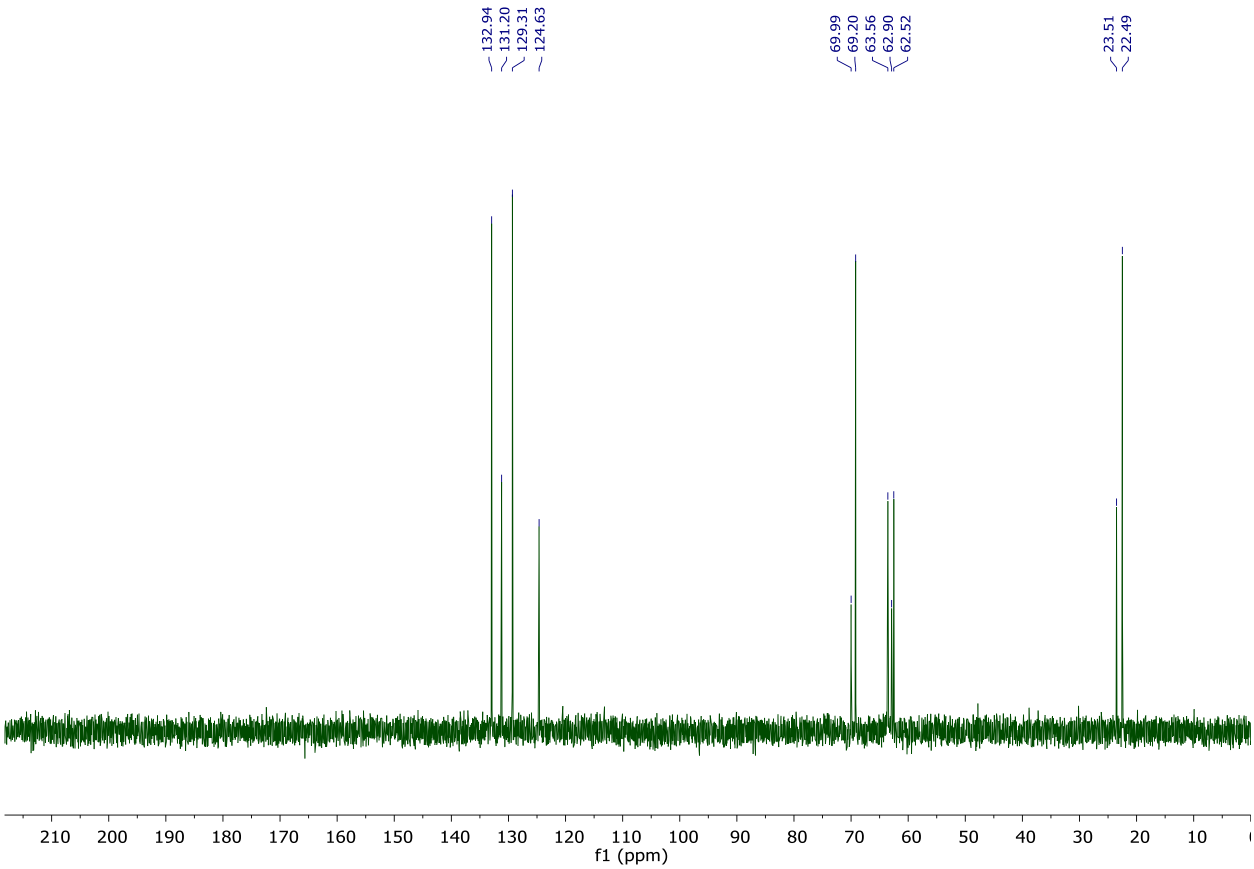
132.94
131.20
129.31
124.63
69.99
69.20
63.56
62.90
62.52
23.51
22.49
210 200 190 180 170 160 150 140 130 120 110 100 90 80 70 60 50 40 30 20 10
f1 (ppm)

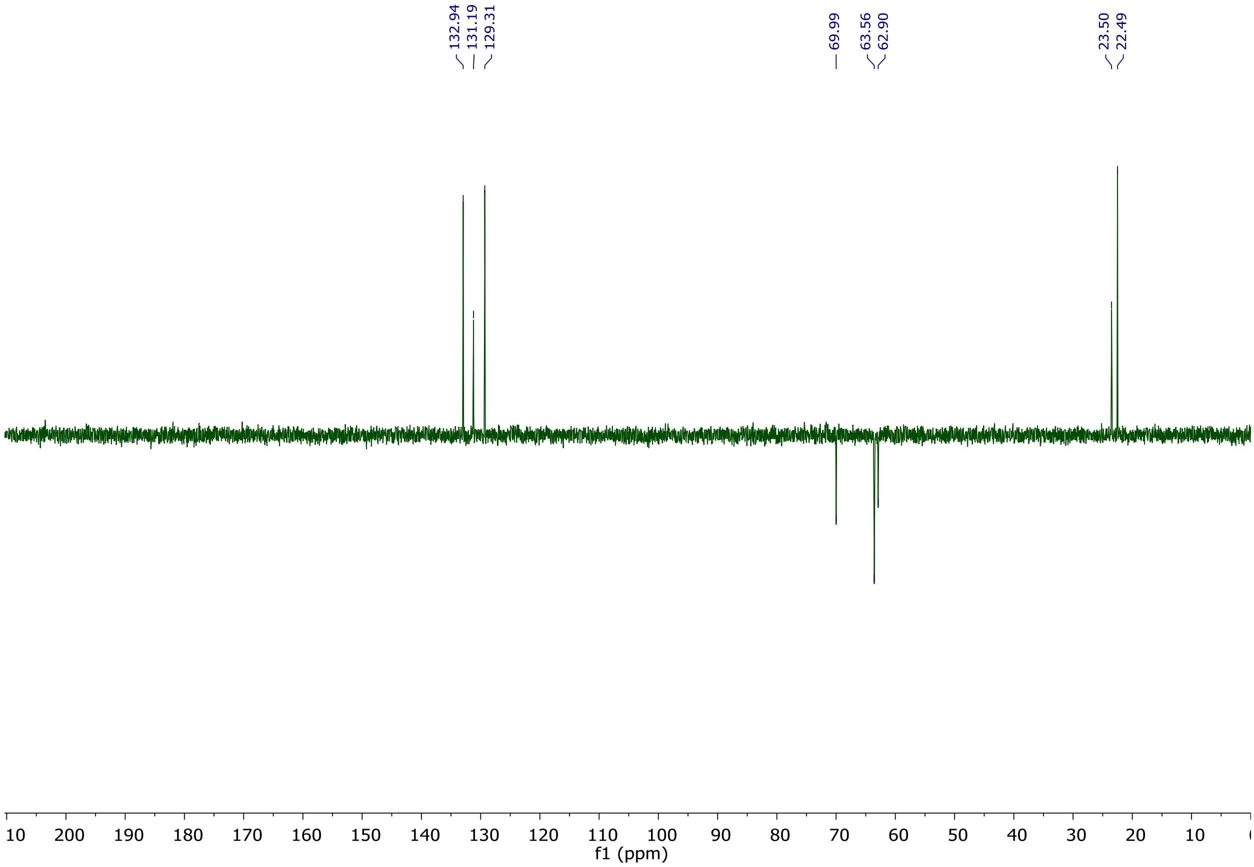
132.94
131.19
129.31
69.99
63.56
62.90
23.50
22.49
10 200 190 180 170 160 150 140 130 120 110 100 90 80 70 60 50 40 30 20 10
f1 (ppm)

HSQC

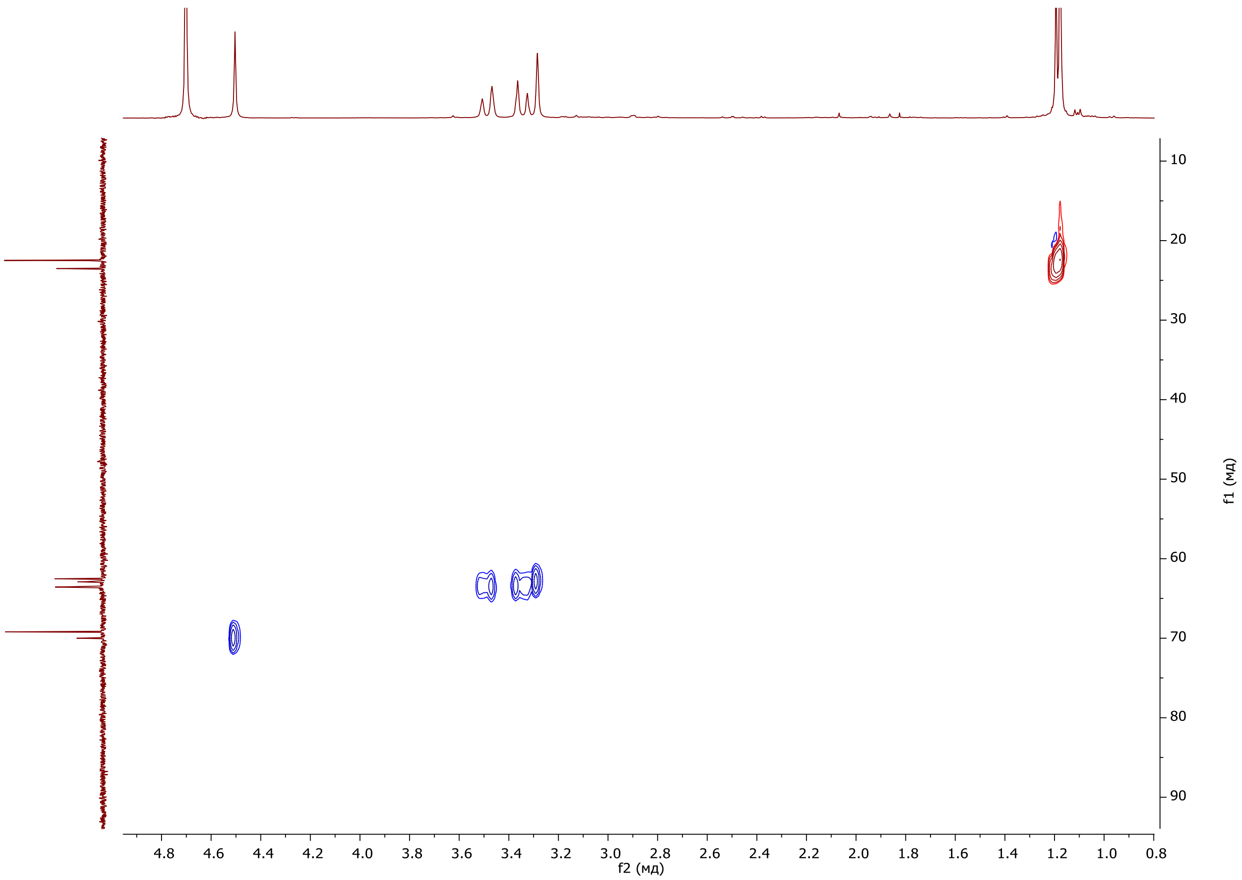

HMBC

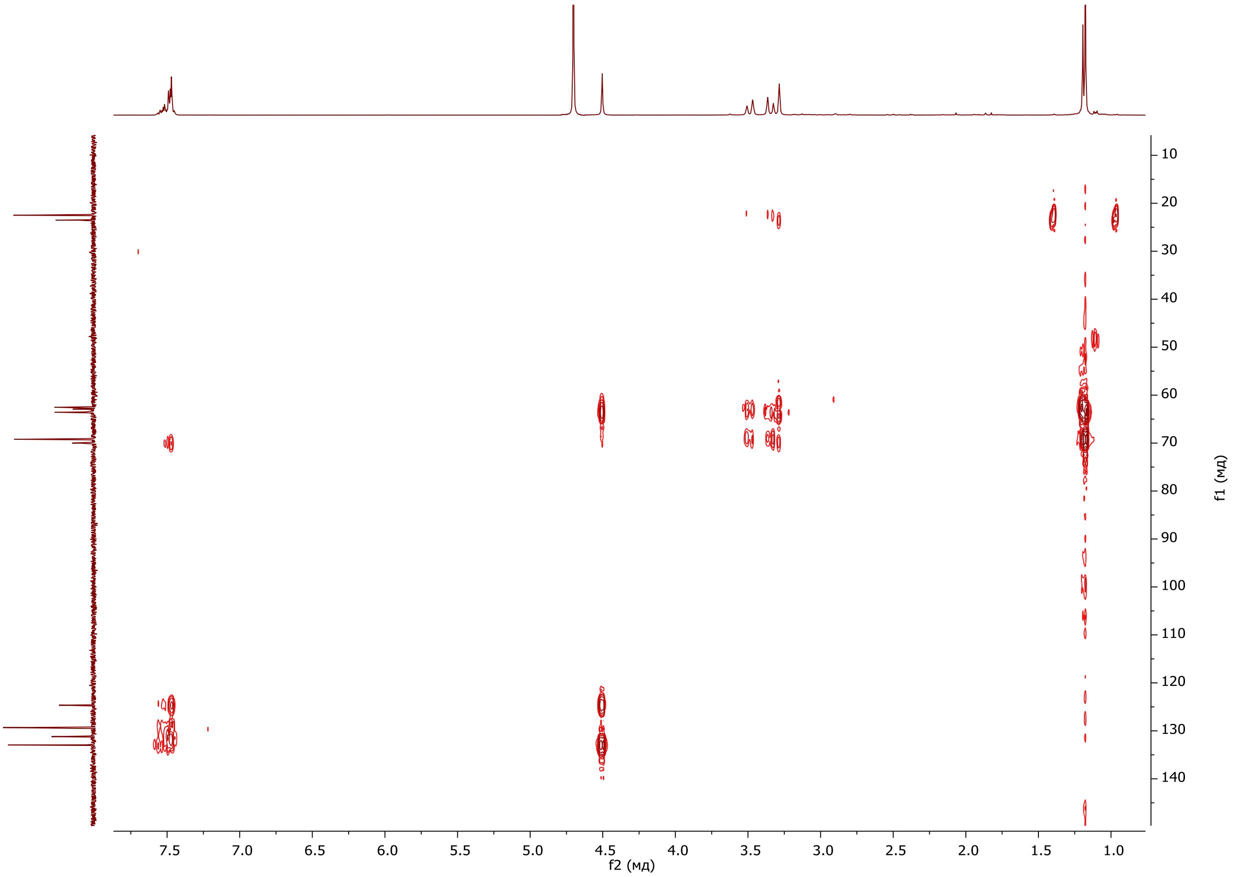

3. X-ray analysis

Preparation of single crystals for X-ray diffraction analysis

4a·H_2O·0.5MeOH: selective MeOH evaporation (by $CaCl_2$ in closed jar) from solution of **4a** in MeOH/ether.

4a·HCl·H_2O·MeOH: slow evaporation of solution of **4a**·HCl in H_2O/MeOH mixture.

4c·HCl·3.5MeOH: slow cooling of solution of **4c**·HCl in MeOH and thermal cycling 20–40 °C (8–10 times).

Bn-4c(bromide)·$3CD_3OD$: slow cooling of solution of **Bn-4c**(bromide) in CD_3OD.

8a: ether vapors diffusion into solution of **8a** in MeOH.

19e·3HCl·H_2O: MeOH evaporation (by $CaCl_2$ in closed jar) from solution of **19e**·3HCl·$1.5H_2O$ in MeOH at 2–4 °C.

21·HCl·$2H_2O$: selective MeOH evaporation (by $CaCl_2$ in closed jar) from solution of **21**·HCl·$2H_2O$ in MeOH/ether mixture at 2–4 °C.

21·$2D_2O$: slow cooling of solution of **21** in D_2O.

Crystals of hydrazinium dihydrochloride from the synthesis of ozatriazaadamantane **22**

TAAD 4a·H_2O·0.5CH_3OH

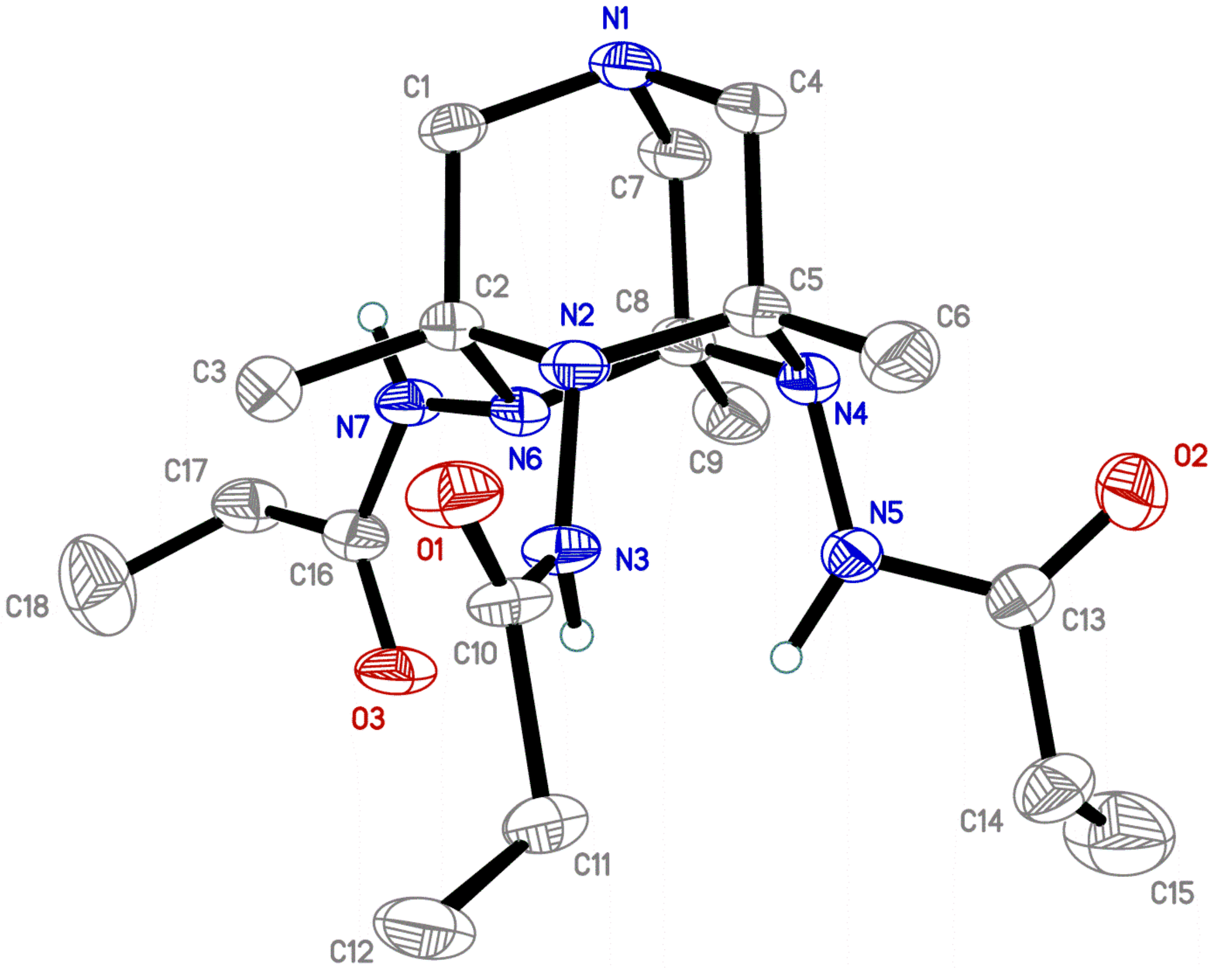

Figure S1. General view of **4a**·H_2O·0.5CH_3OH in representation of atoms via thermal ellipsoids at 50% probability level; hydrogen atoms (except for those of the NH groups) and solvate molecules are omitted for clarity.

X-ray crystallographic data and refinement details

X-ray diffraction data were collected at 200K on a Bruker Quest D8 diffractometer equipped with a Photon-III area-detector (graphite monochromator, shutterless φ- and ω-scan technique), using Mo Kα-radiation. The intensity data were integrated by the SAINT program[4] and were corrected for absorption and decay using SADABS.[5] The structure was solved by direct methods using SHELXT[6] and refined on F^2 using SHELXL-2018.[7] All non-hydrogen atoms were refined with anisotropic displacement parameters. Hydrogen atoms connected to nitrogen atoms (H7, H3NA, H3NB, H5NA and H5NB) were refined with individual isotropic displacement parameters; their positions were found from the electron density-difference map. All other hydrogen atoms were placed in ideal calculated positions and refined as riding atoms with relative isotropic displacement parameters. A rotating group model was applied for methyl groups. Crystal channels contained a highly disordered non-coordinating methanol molecule (located near an inversion center and having the total occupancy of 0.5); this molecule was

---

[4] Bruker. APEX-III. *Bruker AXS Inc.*, Madison, Wisconsin, USA, **2019**.

[5] Krause, L.; Herbst-Irmer, R.; Sheldrick, G. M.; Stalke, D. *J. Appl. Cryst.* **2015**, 48, 3–10.

[6] Sheldrick, G. M. *Acta Cryst.* **2015**, A71, 3-8.

[7] Sheldrick, G. M. *Acta Cryst.* **2015**, C71, 3-8.

removed by the SQUEEZE method[8] implemented in the PLATON program.[9] The SHELXTL program suite[4] was used for molecular graphics. CCDC 2182991 contains the supplementary crystallographic data for **4a**·H_2O·0.5CH_3OH. These data can be obtained free of charge via http://www.ccdc.cam.ac.uk/conts/retrieving.html (or from the CCDC, 12 Union Road, Cambridge, CB21EZ, UK; or deposit@ccdc.cam.ac.uk).

Table S1. Crystal data and structure refinement for **4a·H_2O·0.5CH_3OH**

Empirical formula	$C_{18.5}H_{37}N_7O_{4.5}$	
Formula weight	429.55	
Temperature	200(2) K	
Wavelength	0.71073 Å	
Crystal system	Monoclinic	
Space group	$P2_1/n$	
Unit cell dimensions	a = 12.0963(7) Å	α= 90°.
	b = 13.4159(8) Å	β= 108.0401(14)°.
	c = 16.0495(9) Å	γ = 90°.
Volume	2476.5(2) Å^3	
Z	4	
Density (calculated)	1.152 g/cm^3	
Absorption coefficient	0.084 mm^{-1}	
F(000)	932	
Crystal size	0.59 x 0.28 x 0.16 mm^3	
Theta range for data collection	2.021 to 32.034°.	
Index ranges	-18<=h<=18, -20<=k<=20, -23<=l<=23	
Reflections collected	70973	
Independent reflections	8590 [R(int) = 0.0587]	
Observed reflections	5248	
Completeness to theta = 25.242°	99.8 %	
Absorption correction	Semi-empirical from equivalents	
Max. and min. transmission	0.8626 and 0.7978	
Refinement method	Full-matrix least-squares on F^2	
Data / restraints / parameters	8590 / 25 / 328	
Goodness-of-fit on F^2	1.058	
Final R indices [I>2sigma(I)]	R1 = 0.0887, wR2 = 0.2460	
R indices (all data)	R1 = 0.1313, wR2 = 0.2832	
Extinction coefficient	0.017(3)	
Largest diff. peak and hole	0.512 and -0.403 e.Å^{-3}	

[8] Spek, A. L. *Acta Cryst.*, 2015, **C71**, 9-18.
[9] Spek, A. L. *Acta Cryst.*, 2009, **D65**, 148-155.

TAAD 4a·HCl·H_2O·MeOH

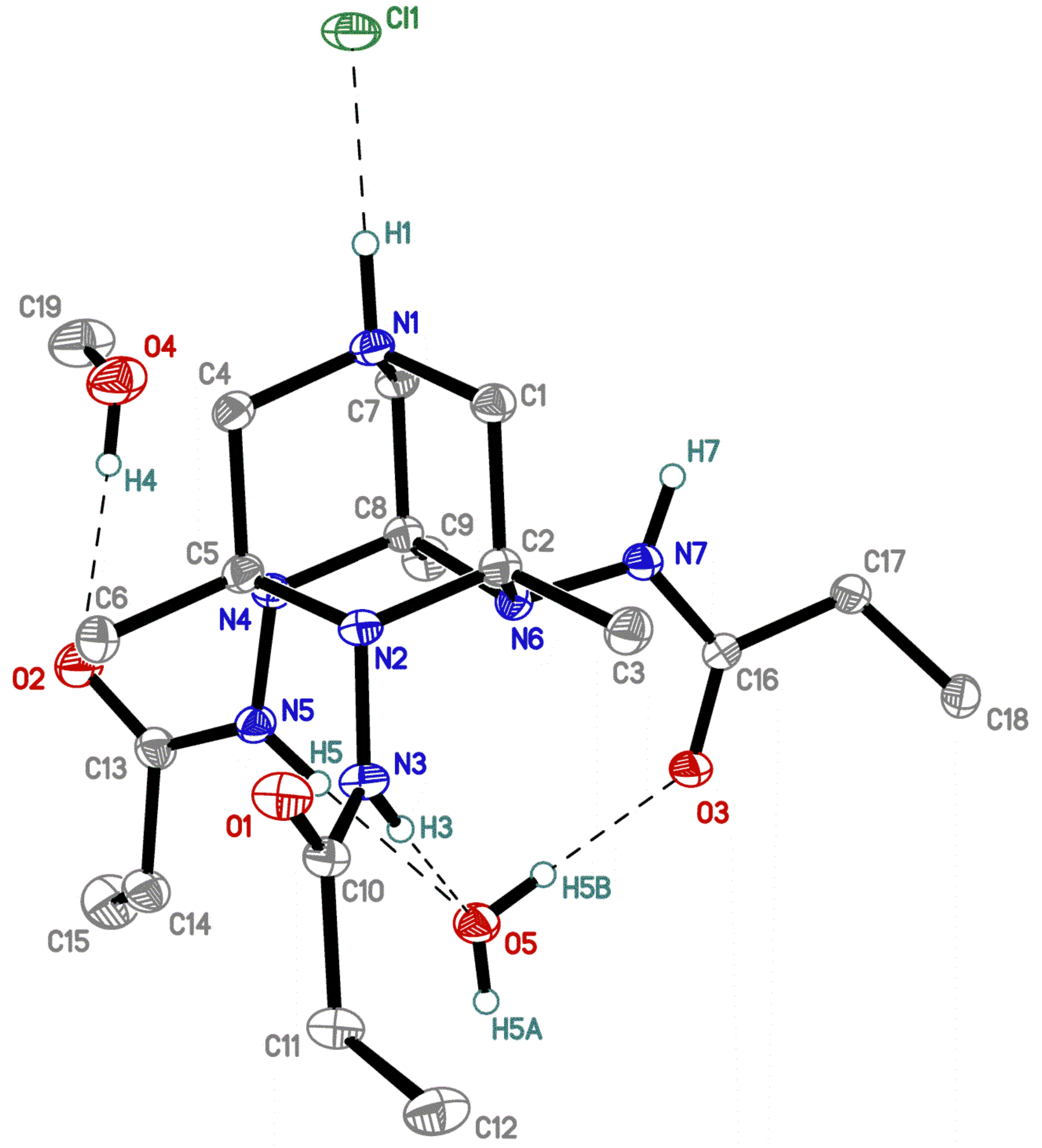

Figure S2. General view of **4a**·HCl·H_2O·MeOH in representation of atoms via thermal ellipsoids at 50% probability level; hydrogen atoms (except for those of the NH and OH groups) are omitted for clarity.

X-ray crystallographic data and refinement details

X-ray diffraction data were collected at 100K on a Bruker Quest D8 diffractometer equipped with a Photon-III area-detector (graphite monochromator, shutterless ω-scan technique), using Mo K_α-radiation. The intensity data were integrated by the SAINT program[4] and were corrected for absorption and decay using SADABS.[5] The structure was solved by direct methods using SHELXT[6] and refined on F^2 using SHELXL-2018.[7] Positions of all atoms were found from the electron density-difference map. Atoms were refined with individual anisotropic (non-hydrogen atoms) or isotropic (hydrogen atoms) displacement parameters. The SHELXTL program suite[4] was used for molecular graphics. CCDC 2182992 contains the supplementary crystallographic data for **4a**·HCl·H_2O·MeOH. These data can be obtained free of charge via

http://www.ccdc.cam.ac.uk/conts/retrieving.html (or from the CCDC, 12 Union Road, Cambridge, CB21EZ, UK; or deposit@ccdc.cam.ac.uk).

Table S2. Crystal data and structure refinement for **4a**·HCl·H_2O·MeOH

Empirical formula	$C_{19}H_{40}ClN_7O_5$	
Formula weight	482.03	
Temperature	100(2) K	
Wavelength	0.71073 Å	
Crystal system	Monoclinic	
Space group	$P2_1/c$	
Unit cell dimensions	a = 16.2637(6) Å	α= 90°.
	b = 11.4667(4) Å	β= 93.7393(8)°.
	c = 13.4039(5) Å	γ = 90°.
Volume	2494.38(16) Å^3	
Z	4	
Density (calculated)	1.284 g/cm^3	
Absorption coefficient	0.196 mm^{-1}	
F(000)	1040	
Crystal size	0.51 x 0.26 x 0.14 mm^3	
Theta range for data collection	2.175 to 37.789°.	
Index ranges	-28<=h<=28, -19<=k<=19, -23<=l<=23	
Reflections collected	105431	
Independent reflections	13390 [R(int) = 0.0896]	
Observed reflections	9031	
Completeness to theta = 25.242°	100.0 %	
Absorption correction	Semi-empirical from equivalents	
Max. and min. transmission	0.7478 and 0.6330	
Refinement method	Full-matrix least-squares on F^2	
Data / restraints / parameters	13390 / 0 / 449	
Goodness-of-fit on F^2	1.061	
Final R indices [I>2sigma(I)]	R1 = 0.0515, wR2 = 0.1053	
R indices (all data)	R1 = 0.0932, wR2 = 0.1266	
Largest diff. peak and hole	0.486 and -0.480 e.Å^{-3}	

TAAD 4c·HCl·3.5MeOH

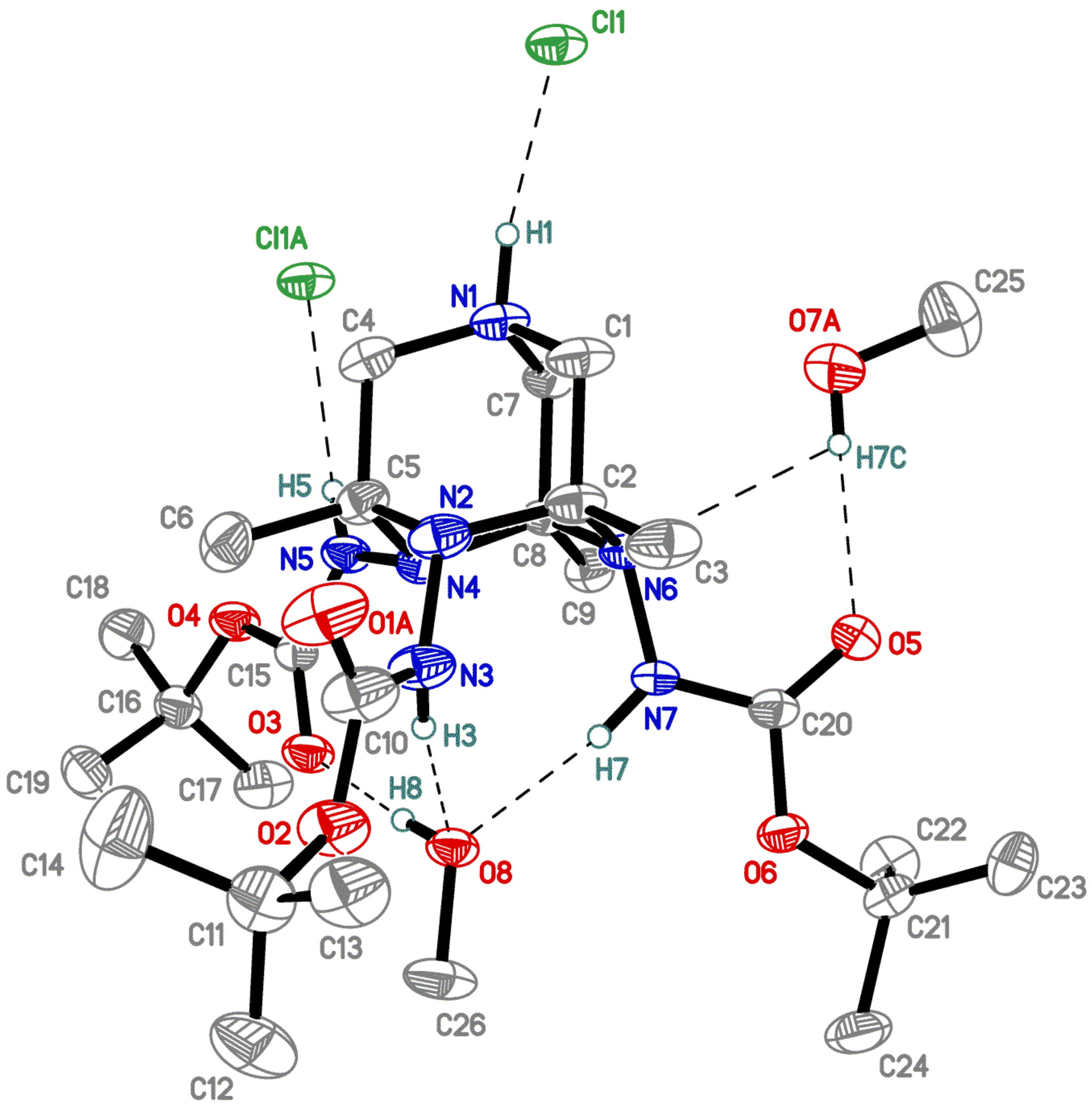

Figure S3. General view of **4c**·HCl·3.5MeOH in representation of atoms via thermal ellipsoids at 50% probability level; hydrogen atoms (except for those of the NH groups) and non-coordinating methanol molecules are omitted for clarity.

X-ray crystallographic data and refinement details

X-ray diffraction data were collected at 100K on a Bruker Quest D8 diffractometer equipped with a Photon-III area-detector (graphite monochromator, shutterless φ- and ω-scan technique), using Mo Kα-radiation. The intensity data were integrated by the SAINT program[4] and were corrected for absorption and decay using SADABS.[5] The structure was solved by direct methods using SHELXT[6] and refined on F^2 using SHELXL-2018.[7] All non-hydrogen atoms were refined with anisotropic displacement parameters. Hydrogen atoms H1, H3, H5, H7 and H8 connected to atoms N1, N3, N5, N7 and O8, correspondingly, were refined with individual isotropic displacement parameters; their positions were found from the electron density-difference map. All other hydrogen atoms were placed in ideal calculated positions and refined as riding atoms with relative isotropic displacement parameters. A rotating group model was applied for methyl groups. Crystal channels contained highly disordered non-coordinating methanol molecules

(overall occupancy of 1.5); these molecules were removed by the SQUEEZE method[8] implemented in the PLATON program.[9] The SHELXTL program suite[4] was used for molecular graphics. CCDC 2182993 contains the supplementary crystallographic data for **4c**·HCl·3.5MeOH. These data can be obtained free of charge via http://www.ccdc.cam.ac.uk/conts/retrieving.html (or from the CCDC, 12 Union Road, Cambridge, CB21EZ, UK; or deposit@ccdc.cam.ac.uk).

Table S3. Crystal data and structure refinement for **4c**·HCl·3.5MeOH

Empirical formula	$C_{27.5}H_{60}ClN_7O_{9.5}$	
Formula weight	676.27	
Temperature	100(2) K	
Wavelength	0.71073 Å	
Crystal system	Monoclinic	
Space group	C2/c	
Unit cell dimensions	a = 31.7369(7) Å	α= 90°.
	b = 12.6737(3) Å	β= 130.5508(5)°.
	c = 24.1811(5) Å	γ = 90°.
Volume	7390.3(3) Å^3	
Z	8	
Density (calculated)	1.216 g/cm^3	
Absorption coefficient	0.160 mm^{-1}	
F(000)	2936	
Crystal size	0.23 x 0.19 x 0.15 mm^3	
Theta range for data collection	2.217 to 33.145°.	
Index ranges	-48<=h<=48, -19<=k<=19, -37<=l<=37	
Reflections collected	139270	
Independent reflections	14100 [R(int) = 0.0725]	
Observed reflections	9013	
Completeness to theta = 25.242°	99.9 %	
Absorption correction	Semi-empirical from equivalents	
Max. and min. transmission	0.6501 and 0.6033	
Refinement method	Full-matrix least-squares on F^2	
Data / restraints / parameters	14100 / 14 / 422	
Goodness-of-fit on F^2	1.027	
Final R indices [I>2sigma(I)]	R1 = 0.0545, wR2 = 0.1312	
R indices (all data)	R1 = 0.0946, wR2 = 0.1570	
Extinction coefficient	0.00078(13)	
Largest diff. peak and hole	0.650 and -0.532 e.Å^{-3}	

Bn-4c·3CD3OD (bromide salt)

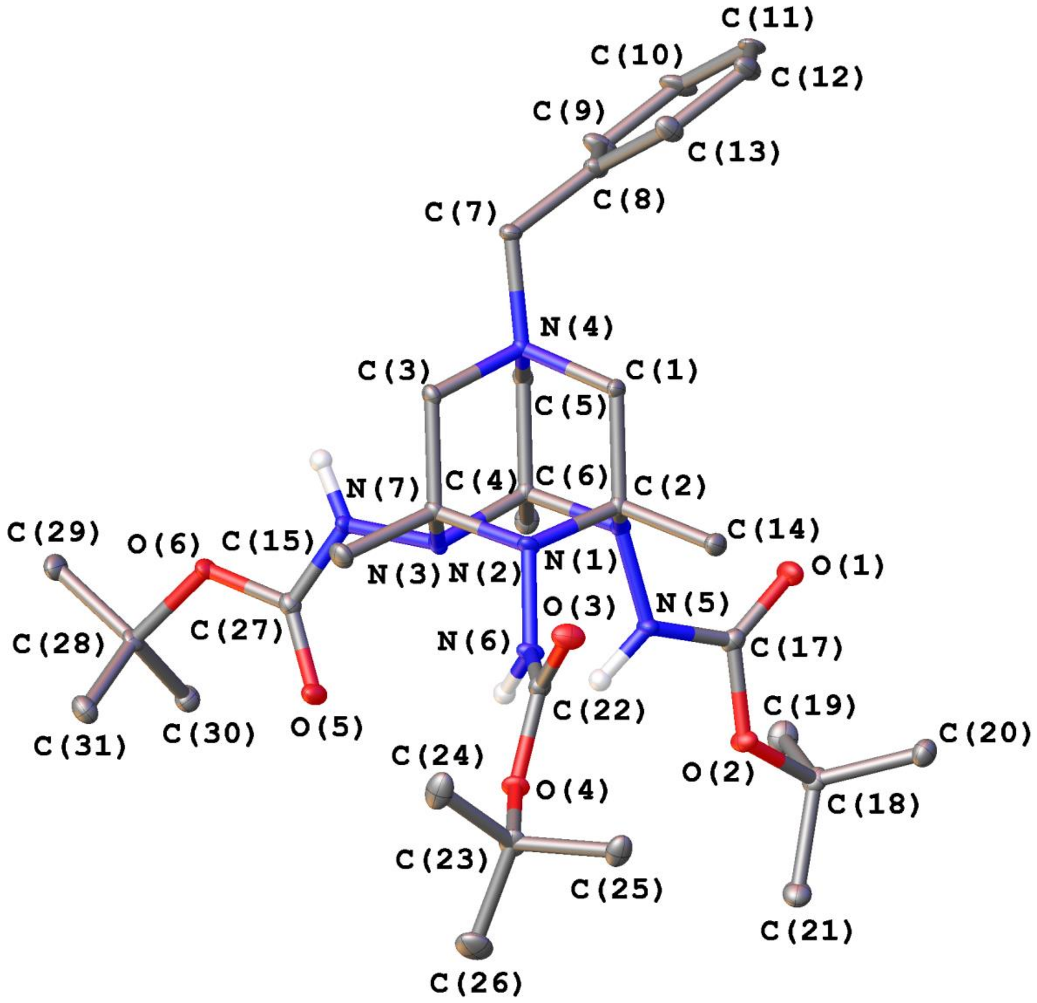

Figure S4. General view of TAAD cation in **Bn-4c(bromide)**·3CD3OD in representation of atoms via thermal ellipsoids at 30% probability level. Hydrogen atoms except those of NH and OH groups are omitted, as counterions and solvate methanol molecules are.

X-ray crystallographic data and refinement details

X-ray diffraction data were collected at 100 K with a Bruker Quest D8 CMOS diffractometer, using graphite monochromated Mo-Kα radiation (λ = 0.71073 Å, ω-scans). Structures were solved using Intrinsic Phasing with the ShelXT[6] structure solution program in Olex2[10] and then refined with the XL[11] refinement package using Least-Squares minimization against F^2 in the anisotropic approximation for non-hydrogen atoms. Hydrogen atoms of NH and OH groups and those of solvent water and methanol molecules were found in difference Fourier synthesis while positions of other hydrogen atoms were calculated, and they were refined in the isotropic approximation within the riding model. Crystal data and structure refinement parameters are given in Table S4. CCDC 2190362 contains the supplementary crystallographic data for **Bn-4c(bromide)**·3CD3OD. These data can be obtained free of charge via http://www.ccdc.cam.ac.uk/conts/retrieving.html (or from the CCDC, 12 Union Road, Cambridge, CB21EZ, UK; or deposit@ccdc.cam.ac.uk).

[10] O.V. Dolomanov, L.J. Bourhis, R.J. Gildea, J.A.K. Howard, H. Puschmann. *J. Appl. Cryst.* **2009**, *42*, 339-341.
[11] G.M. Sheldrick. *Acta Cryst.* **2008**, *A64*, 112-122.

Table S4. Crystal data and structure refinement parameters for **Bn-4c(bromide)**·$3CD_3OD$.

Empirical formula	$C_{34}H_{64}BrN_7O_9$
Formula weight	794.83
T, K	100
Crystal system	Monoclinic
Space group	$P2_1/c$
Z	4
a, Å	13.4137(3)
b, Å	12.8590(3)
c, Å	23.9435(5)
α, °	90
β, °	95.2850(10)
γ, °	90
V, Å^3	4112.38(16)
D_{calc} (g cm^{-1})	1.284
Linear absorption, μ (cm^{-1})	10.56
F(000)	1696
$2\theta_{max}$, °	54
Reflections measured	45792
Independent reflections	8961
Observed reflections [$I > 2\sigma(I)$]	7136
Parameters	480
R1	0.0442
wR2	0.1107
GOF	1.046
$\Delta\rho_{max}$/ $\Delta\rho_{min}$ (e Å^{-3})	0.738/-0.732

TAAD 8a

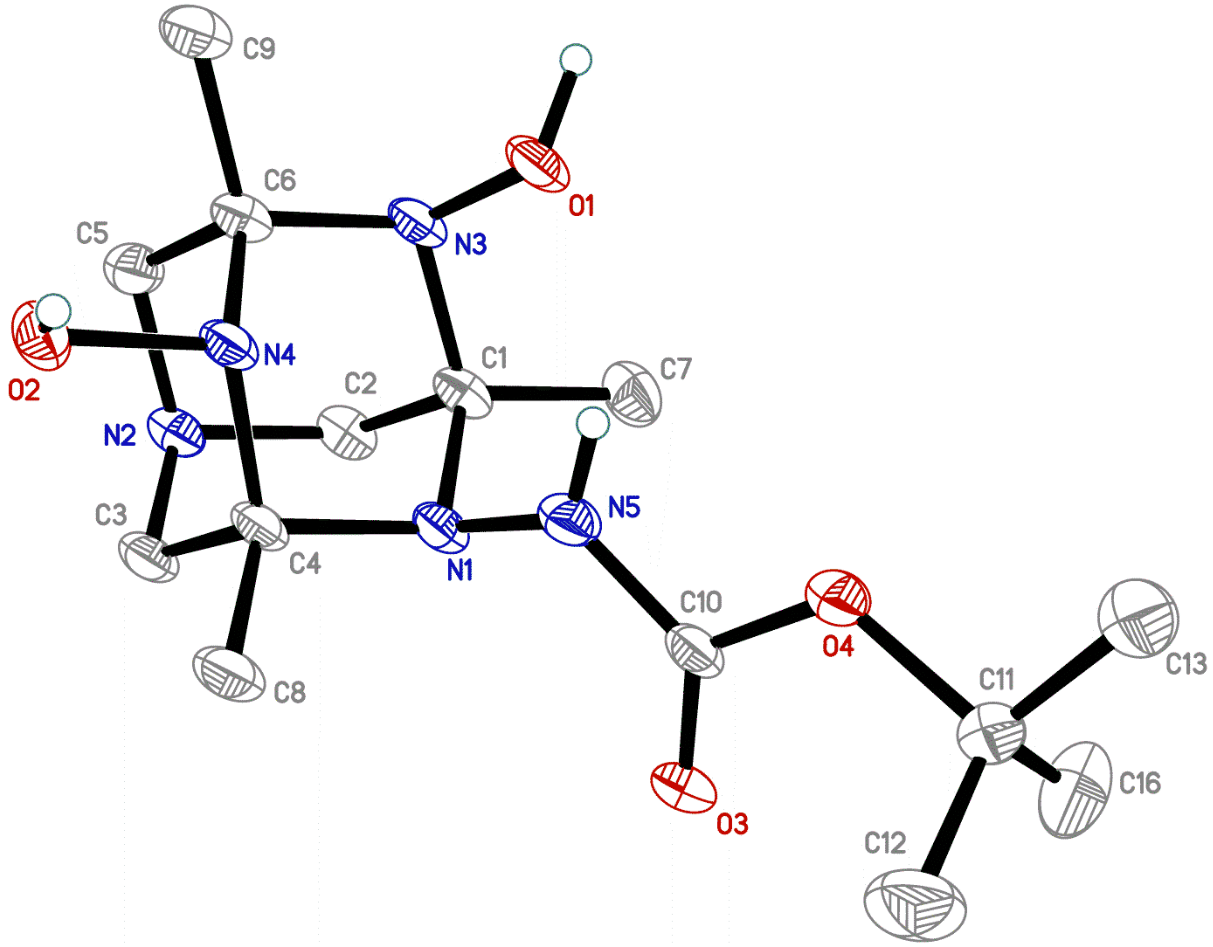

Figure S5. General view of **8a** in representation of atoms via thermal ellipsoids at 50% probability level; hydrogen atoms (except for those of the NH and OH groups) are omitted for clarity.

X-ray crystallographic data and refinement details

X-ray diffraction data were collected at 100K on a Bruker Quest D8 diffractometer equipped with a Photon-III area-detector (graphite monochromator, shutterless φ- and ω-scan technique), using Mo Kα-radiation. The intensity data were integrated by the SAINT program[4] and were corrected for absorption and decay using SADABS.[5] The structure was solved by direct methods using SHELXT[6] and refined on F^2 using SHELXL-2018.[7] All non-hydrogen atoms were refined with individual anisotropic displacement parameters. Locations of atoms H1, H2 and H5 were found from the electron density-difference map; these hydrogen atoms were refined with individual isotropic displacement parameters. All other hydrogen atoms were placed in ideal calculated positions and refined as riding atoms with relative isotropic displacement parameters. The SHELXTL program suite[4] was used for molecular graphics. CCDC 2182994 contains the supplementary crystallographic data for **8a**. These data can be obtained free of charge via http://www.ccdc.cam.ac.uk/conts/retrieving.html (or from the CCDC, 12 Union Road, Cambridge, CB21EZ, UK; or deposit@ccdc.cam.ac.uk).

Table S5. Crystal data and structure refinement for **8a**

Empirical formula	$C_{14}H_{27}N_5O_4$	
Formula weight	329.39	
Temperature	100(2) K	
Wavelength	0.71073 Å	
Crystal system	Monoclinic	
Space group	$P2_1/c$	
Unit cell dimensions	a = 6.1274(4) Å	α= 90°.
	b = 22.1748(16) Å	β= 99.314(2)°.
	c = 12.4734(9) Å	γ = 90°.
Volume	1672.5(2) $Å^3$	
Z	4	
Density (calculated)	1.308 g/cm^3	
Absorption coefficient	0.097 mm^{-1}	
F(000)	712	
Crystal size	0.400 x 0.238 x 0.100 mm^3	
Theta range for data collection	1.837 to 32.247°.	
Index ranges	-8<=h<=9, -33<=k<=33, -18<=l<=18	
Reflections collected	43484	
Independent reflections	5916 [R(int) = 0.1372]	
Observed reflections	3062	
Completeness to theta = 25.242°	100.0 %	
Absorption correction	Semi-empirical from equivalents	
Max. and min. transmission	0.8624 and 0.6164	
Refinement method	Full-matrix least-squares on F^2	
Data / restraints / parameters	5916 / 0 / 226	
Goodness-of-fit on F^2	1.028	
Final R indices [I>2sigma(I)]	R1 = 0.0874, wR2 = 0.1764	
R indices (all data)	R1 = 0.1753, wR2 = 0.2149	
Largest diff. peak and hole	0.383 and -0.365 e.$Å^{-3}$	

TAAD 19e·3HCl·H_2O

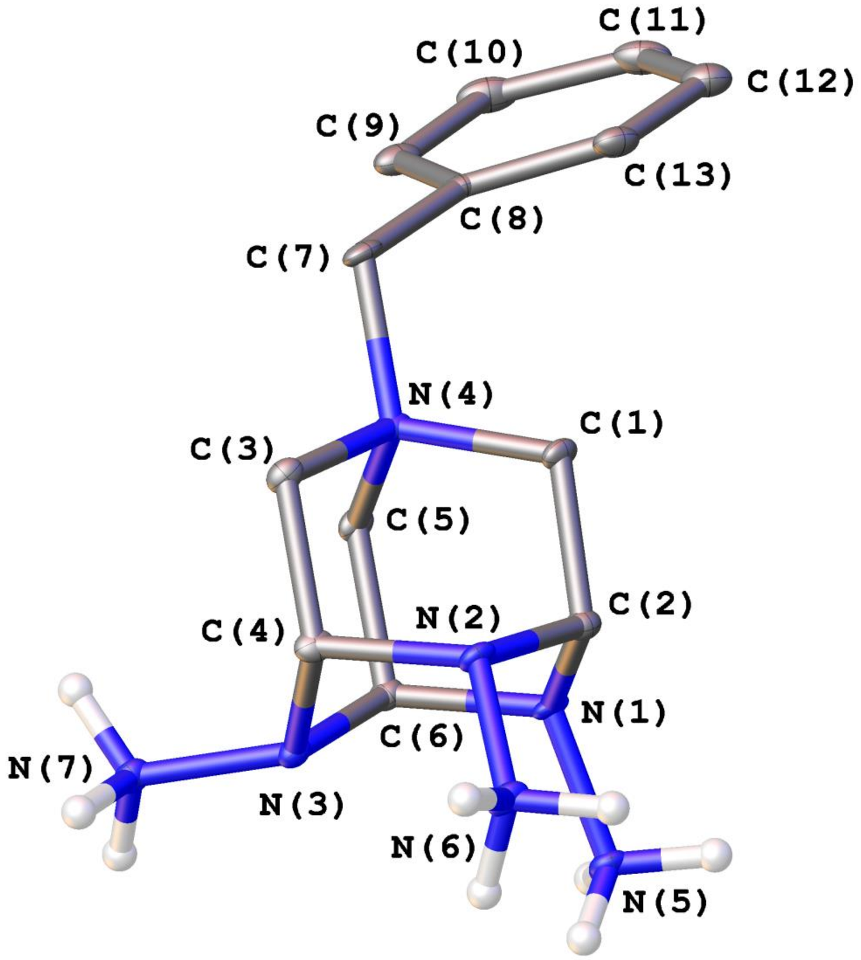

Figure S6. General view of TAAD cation in **19e**·3HCl·H_2O in representation of atoms via thermal ellipsoids at 30% probability level. Hydrogen atoms except those of NH and OH groups are omitted, as counterions and solvate water molecules are.

X-ray crystallographic data and refinement details

X-ray diffraction data were collected at 100 K with a Bruker Quest D8 CMOS diffractometer, using graphite monochromated Mo-Kα radiation (λ = 0.71073 Å, ω-scans). Structures were solved using Intrinsic Phasing with the ShelXT[6] structure solution program in Olex2[10] and then refined with the XL[11] refinement package using Least-Squares minimization against F^2 in the anisotropic approximation for non-hydrogen atoms. Hydrogen atoms of NH and OH groups and those of solvent water and methanol molecules were found in difference Fourier synthesis while positions of other hydrogen atoms were calculated, and they were refined in the isotropic approximation within the riding model. Crystal data and structure refinement parameters are given in Table S6. CCDC 2190361 contains the supplementary crystallographic data for **19e**·3HCl·H_2O. These data can be obtained free of charge via http://www.ccdc.cam.ac.uk/conts/retrieving.html (or from the CCDC, 12 Union Road, Cambridge, CB21EZ, UK; or deposit@ccdc.cam.ac.uk).

Table S6. Crystal data and structure refinement parameters for **19e**·3HCl·H_2O.

Empirical formula	$C_{13}H_{29}Cl_4N_7O_2$
Formula weight	457.23
T, K	100
Crystal system	Monoclinic
Space group	$P2_1/n$
Z	4
a, Å	13.2715(12)
b, Å	8.7048(8)
c, Å	18.2480(17)
α, °	90
β, °	110.502(6)
γ, °	90
V, $Å^3$	1974.6(3)
D_{calc} (g cm^{-1})	1.538
Linear absorption, μ (cm^{-1})	6.24
F(000)	960
$2\theta_{max}$, °	52
Reflections measured	18156
Independent reflections	3877
Observed reflections [$I > 2\sigma(I)$]	2959
Parameters	235
R1	0.0646
wR2	0.1812
GOF	1.043
$\Delta\rho_{max}$/ $\Delta\rho_{min}$ (e $Å^{-3}$)	0.806/-0.657

TAAD 21·$2D_2O$

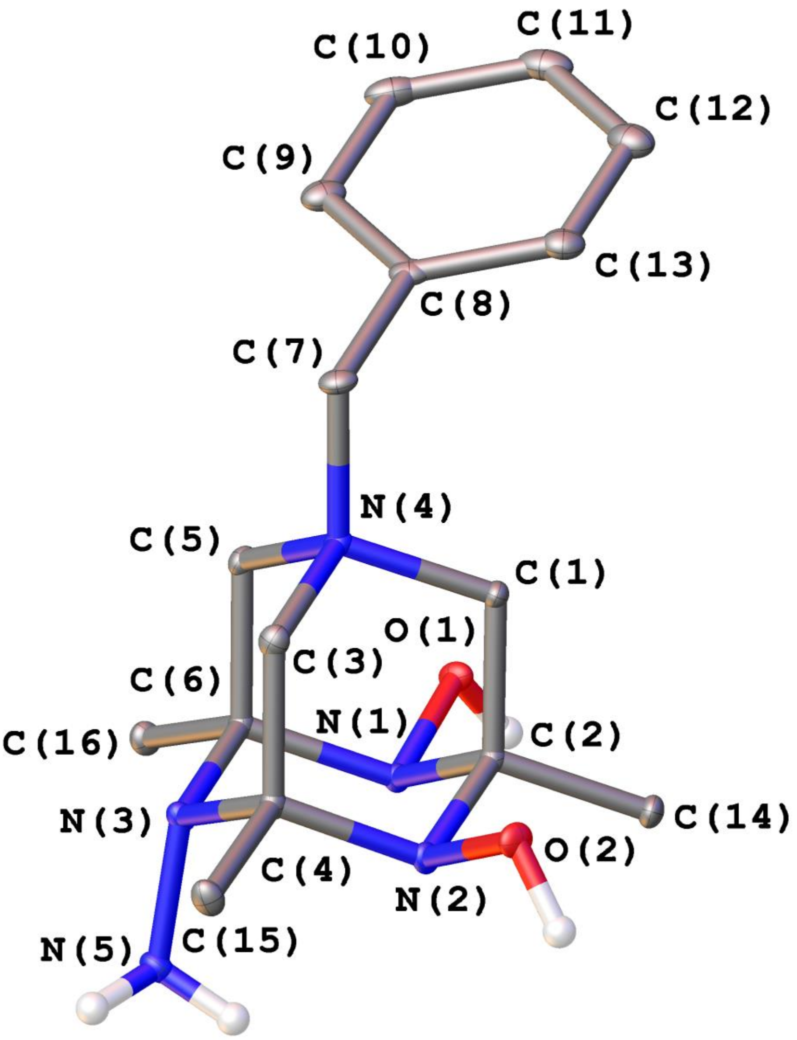

Figure S7. General view of TAAD cation in **21**·$2D_2O$ in representation of atoms via thermal ellipsoids at 30% probability level. Hydrogen atoms except those of NH and OH groups are omitted, as counterions and solvate water molecules are.

X-ray crystallographic data and refinement details

X-ray diffraction data were collected at 100 K with a Bruker Quest D8 CMOS diffractometer, using graphite monochromated Mo-Kα radiation (λ = 0.71073 Å, ω-scans). Structures were solved using Intrinsic Phasing with the ShelXT[6] structure solution program in Olex2[10] and then refined with the XL[11] refinement package using Least-Squares minimization against F^2 in the anisotropic approximation for non-hydrogen atoms. Hydrogen atoms of NH and OH groups and those of solvent water and methanol molecules were found in difference Fourier synthesis while positions of other hydrogen atoms were calculated, and they were refined in the isotropic approximation within the riding model. Crystal data and structure refinement parameters are given in Table S7. CCDC 2190363 contains the supplementary crystallographic data for **21**·$2D_2O$. These data can be obtained free of charge via http://www.ccdc.cam.ac.uk/conts/retrieving.html (or from the CCDC, 12 Union Road, Cambridge, CB21EZ, UK; or deposit@ccdc.cam.ac.uk).

Table S7. Crystal data and structure refinement parameters for **21**·$2D_2O$.

Empirical formula	$C_{16}H_{30}ClN_5O_4$
Formula weight	391.90
T, K	100
Crystal system	Orthorhombic
Space group	$Pna2_1$
Z	4
a, Å	10.4836(2)
b, Å	21.5372(4)
c, Å	8.4050(2)
α, °	90
β, °	90
γ, °	90
V, Å^3	1897.74(7)
D_{calc} (g cm^{-1})	1.372
Linear absorption, μ (cm^{-1})	2.34
F(000)	840
$2\theta_{max}$, °	56
Reflections measured	22665
Independent reflections	4579
Observed reflections [$I > 2\sigma(I)$]	4370
Parameters	238
R1	0.0312
wR2	0.0787
GOF	1.026
$\Delta\rho_{max}$/ $\Delta\rho_{min}$ (e Å^{-3})	0.538/-0.410

TAAD 21·HCl·2H_2O

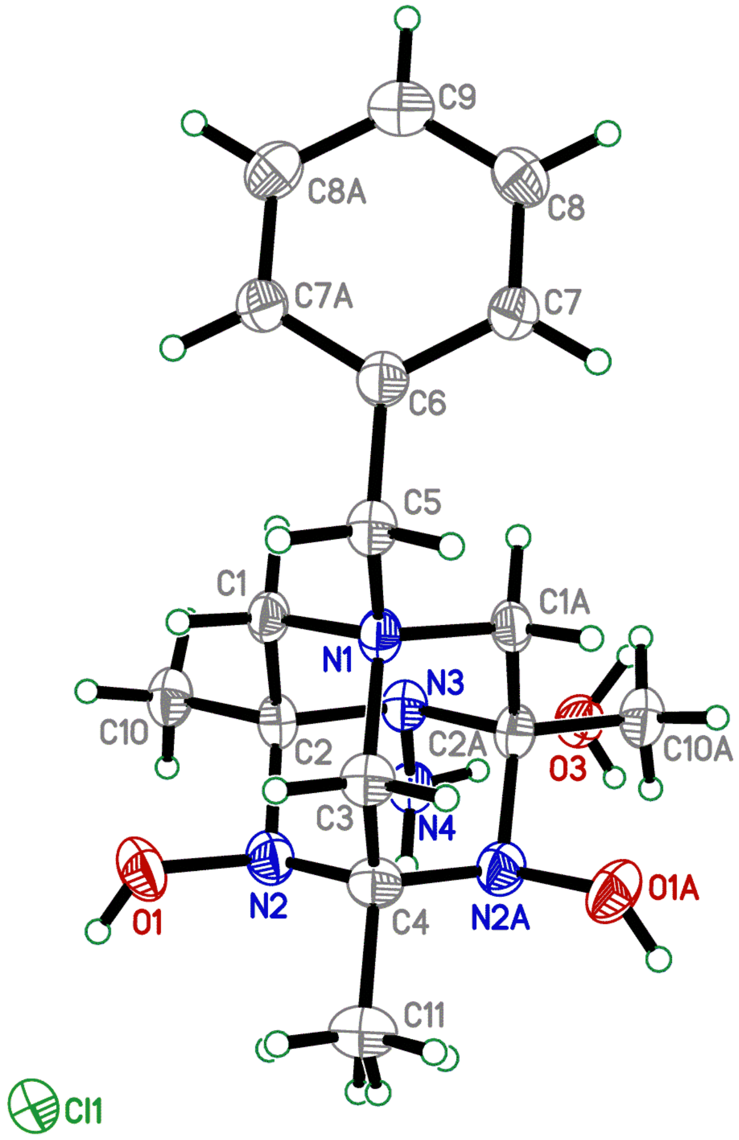

Figure S8. General view of **21**·HCl·2H_2O in representation of atoms via thermal ellipsoids at 50% probability level; hydrogen atoms (except for those of the NH and OH groups) and solvate molecules are omitted for clarity.

X-ray crystallographic data and refinement details

X-ray diffraction data were collected at 100K on a Bruker Quest D8 diffractometer equipped with a Photon-III area-detector (graphite monochromator, shutterless ω-scan technique), using Mo K_α-radiation. The intensity data were integrated by the SAINT program[4] and were corrected for absorption and decay using SADABS.[5] The structure was solved by direct methods using SHELXT[6] and refined on F^2 using SHELXL-2018.[7] All non-hydrogen atoms were refined with anisotropic displacement parameters. Locations of atoms H1, H2A, H2B, H4A and H4B were found from the electron density-difference map. The positions of H4A and H4B were restrained at the distance of 0.85(2)Å from N4, and the position of H1 was fixed at 0.840 Å from O1. These hydrogen atoms were refined with individual isotropic displacement parameters. All other hydrogen atoms were placed in ideal calculated positions and refined as riding atoms with relative isotropic displacement parameters; a rotating group model was applied for methyl groups. The SHELXTL program suite[4] was used for molecular graphics. CCDC 2182995

contains the supplementary crystallographic data for **21**·HCl·2H_2O. These data can be obtained free of charge via http://www.ccdc.cam.ac.uk/conts/retrieving.html (or from the CCDC, 12 Union Road, Cambridge, CB21EZ, UK; or deposit@ccdc.cam.ac.uk).

Table S8. Crystal data and structure refinement for **21**·HCl·2H_2O

Empirical formula	$C_{16}H_{31}Cl_2N_5O_4$	
Formula weight	428.36	
Temperature	100(2) K	
Wavelength	0.71073 Å	
Crystal system	Orthorhombic	
Space group	Pnma	
Unit cell dimensions	a = 7.6601(6) Å	α= 90°.
	b = 13.9764(12) Å	β= 90°.
	c = 18.8415(15) Å	γ = 90°.
Volume	2017.2(3) $Å^3$	
Z	4	
Density (calculated)	1.410 g/cm^3	
Absorption coefficient	0.354 mm^{-1}	
F(000)	912	
Crystal size	0.285 x 0.247 x 0.038 mm^3	
Theta range for data collection	1.814 to 27.000°.	
Index ranges	-9<=h<=9, -17<=k<=17, -23<=l<=24	
Reflections collected	33879	
Independent reflections	2283 [R(int) = 0.1989]	
Observed reflections	1335	
Completeness to theta = 25.242°	99.9 %	
Absorption correction	Semi-empirical from equivalents	
Max. and min. transmission	0.7461 and 0.6087	
Refinement method	Full-matrix least-squares on F^2	
Data / restraints / parameters	2283 / 2 / 154	
Goodness-of-fit on F^2	1.079	
Final R indices [I>2sigma(I)]	R1 = 0.0655, wR2 = 0.1168	
R indices (all data)	R1 = 0.1305, wR2 = 0.1453	
Largest diff. peak and hole	0.399 and -0.379 e.$Å^{-3}$	

Hydrazinium dihydrochloride

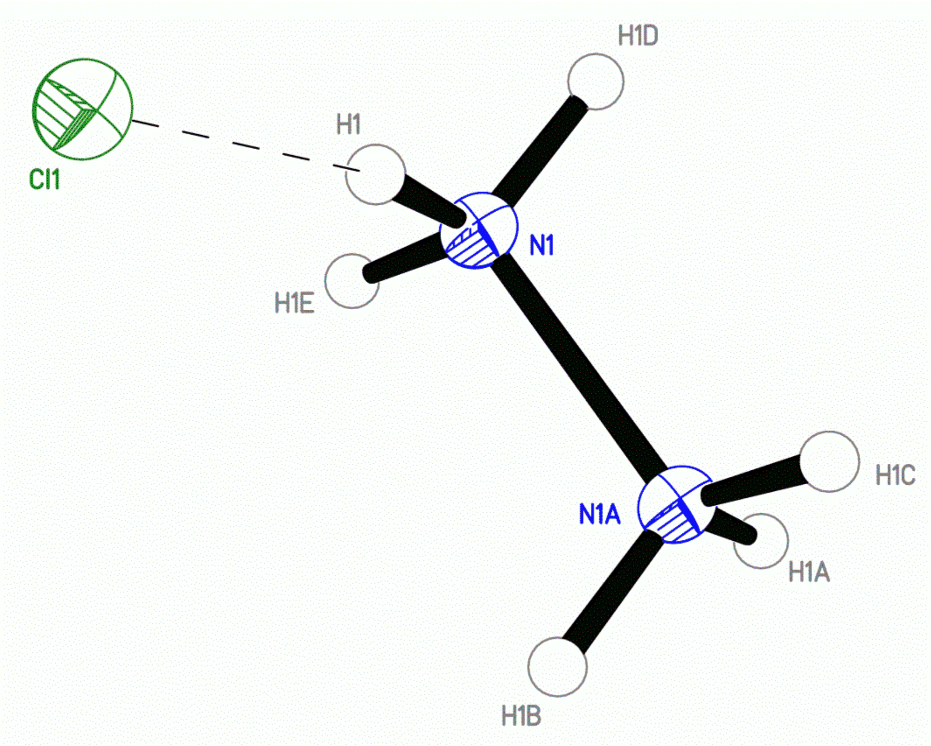

Figure S9. General view of hydrazinium dihydrochloride in representation of atoms via thermal ellipsoids at 50% probability level

X-ray crystallographic data and refinement details

X-ray diffraction data were collected at 100K on a Bruker Quest D8 diffractometer equipped with a Photon-III area-detector (graphite monochromator, shutterless φ- and ω-scan technique), using Mo Kα-radiation. The intensity data were integrated by the SAINT program[4] and were corrected for absorption and decay using SADABS.[5] The structure was solved by direct methods using SHELXT[6] and refined on F^2 using SHELXL-2018.[7] Positions of all atoms were found from the electron density-difference map. Atoms were refined with individual anisotropic (non-hydrogen atoms) or isotropic (hydrogen atom) displacement parameters. The SHELXTL program suite[4] was used for molecular graphics. CCDC 2183048 contains the supplementary crystallographic data for hydrazinium dihydrochloride. These data can be obtained free of charge via http://www.ccdc.cam.ac.uk/conts/retrieving.html (or from the CCDC, 12 Union Road, Cambridge, CB21EZ, UK; or deposit@ccdc.cam.ac.uk).

Table S9. Crystal data and structure refinement for hydrazinium dihydrochloride

Empirical formula	$Cl_2H_6N_2$	
Formula weight	104.97	
Temperature	100(2) K	
Wavelength	0.71073 Å	
Crystal system	Cubic	
Space group	Pa $\bar{3}$	
Unit cell dimensions	a = 7.83790(10) Å	α= 90°.
	b = 7.83790(10) Å	β= 90°.
	c = 7.83790(10) Å	γ = 90°.
Volume	481.503(18) Å^3	
Z	4	
Density (calculated)	1.448 g/cm^3	
Absorption coefficient	1.162 mm^{-1}	
F(000)	216	
Crystal size	0.15 x 0.13 x 0.11 mm^3	
Theta range for data collection	4.504 to 34.239°.	
Index ranges	-12<=h<=12, -11<=k<=12, -12<=l<=10	
Reflections collected	8503	
Independent reflections	342 [R(int) = 0.0356]	
Observed reflections	304	
Completeness to theta = 25.242°	99.3 %	
Absorption correction	Semi-empirical from equivalents	
Max. and min. transmission	0.5664 and 0.4911	
Refinement method	Full-matrix least-squares on F^2	
Data / restraints / parameters	342 / 0 / 11	
Goodness-of-fit on F^2	1.196	
Final R indices [I>2sigma(I)]	R1 = 0.0145, wR2 = 0.0368	
R indices (all data)	R1 = 0.0174, wR2 = 0.0379	
Largest diff. peak and hole	0.165 and -0.204 e.Å^{-3}	

4. DFT calculations

DFT calculations were performed with the Gaussian 16 Rev C.01.[12] ωB97XD DFT functional with GD2 empirical dispersion correction (included in functional by definition) and Def2TZVP basis set were used for geometry optimization and calculations of thermodynamics. Data from X-ray diffraction experiments were used as starting points for geometry optimizations. Basis set superposition error (BSSE) were accounted for by employing the counterpoise procedure, using *counterpoise* keyword. Cartesian coordinates are given in angstroms; absolute energies for all substances are given in hartrees. Analysis of vibrational frequencies was performed for all optimized structures. All compounds were characterized by only real vibrational frequencies. TS were characterized by one imaginary frequency. Wavefunction stability, using *stable* keyword, was also checked for each molecule.

For calculations of optimized geometries, frequencies and thermodynamics following keywords were used:

# opt freq wb97xd nosymm def2tzvp test

[12] Frisch, M. J.; Trucks, G. W.; Schlegel, H. B.; Scuseria, G. E.; Robb, M. A.; Cheeseman, J. R.; Scalmani, G.; Barone, V.; Petersson, G. A.; Nakatsuji, H.; Li, X.; Caricato, M.; Marenich, A. V.; Bloino, J.; Janesko, B. G.; Gomperts, R.; Mennucci, B.; Hratchian, H. P.; Ortiz, J. V.; Izmaylov, A. F.; Sonnenberg, J. L.; Williams-Young, D.; Ding, F.; Lipparini, F.; Egidi, F.; Goings, J.; Peng, B.; Petrone, A.; Henderson, T.; Ranasinghe, D.; Zakrzewski, V. G.; Gao, J.; Rega, N.; Zheng, G.; Liang, W.; Hada, M.; Ehara, M.; Toyota, K.; Fukuda, R.; Hasegawa, J.; Ishida, M.; Nakajima, T.; Honda, Y.; Kitao, O.; Nakai, H.; Vreven, T.; Throssell, K.; Montgomery, J. A., Jr.; Peralta, J. E.; Ogliaro, F.; Bearpark, M. J.; Heyd, J. J.; Brothers, E. N.; Kudin, K. N.; Staroverov, V. N.; Keith, T. A.; Kobayashi, R.; Normand, J.; Raghavachari, K.; Rendell, A. P.; Burant, J. C.; Iyengar, S. S.; Tomasi, J.; Cossi, M.; Millam, J. M.; Klene, M.; Adamo, C.; Cammi, R.; Ochterski, J. W.; Martin, R. L.; Morokuma, K.; Farkas, O.; Foresman, J. B.; Fox, D. J. Gaussian, Inc., Wallingford CT, 2016.

Relative stability of invertomers of TAAD 4a

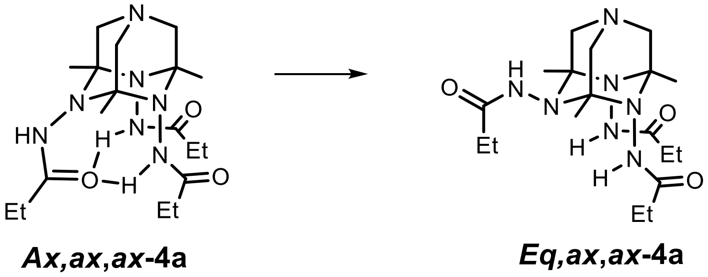

Results:

ΔE_0 Kcal/mol	+4.96
$\Delta H°_{298,15\ K}$ Kcal/mol	+3.97
$\Delta G°_{298,15\ K}$ Kcal/mol	+1.90

Thermochemistry of inclusion complexes of TAAD 4a and TAAD 4c·H[+]

For BSSE correction following input was used:

# opt freq wb97xd nosymm ***counterpoise=2*** *def2tzvp test*

0 1 0 1 0 1

N(Fragment=1) 4.22795100 2.99663800 13.70877200

C(Fragment=1) 5.14069200 3.60637300 12.75530100

…

H(Fragment=2) 2.61587600 3.16952700 8.47405800

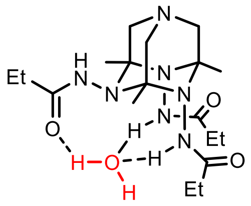

Figure S10. Fragmentation of **H_2O@TAAD 4a**.

Results:

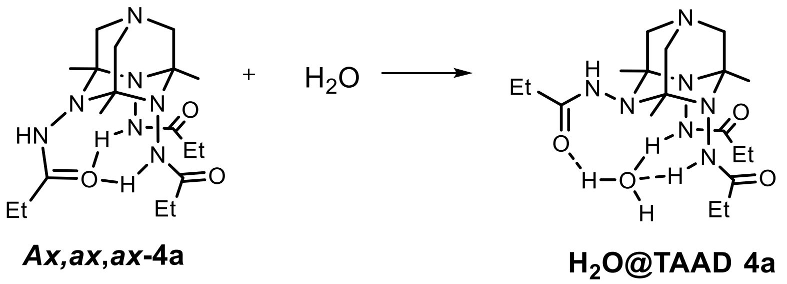

ΔE_0 Kcal/mol	−14.09
$\Delta H°_{298,15\ K}$ Kcal/mol	−12.57
$\Delta G°_{298,15\ K}$ Kcal/mol	−2.79

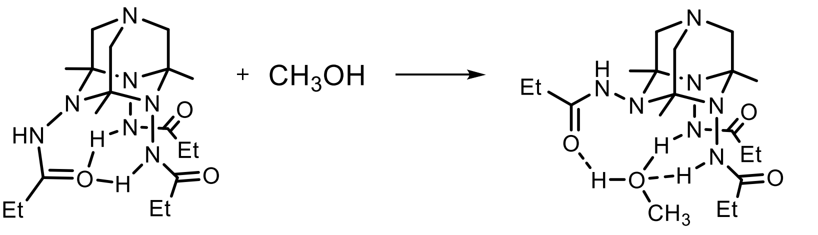

***Ax,ax,ax*-4a** **MeOH@TAAD 4a**

ΔE_0 Kcal/mol	−16.01
$\Delta H°_{298,15\ K}$ Kcal/mol	−14.61
$\Delta G°_{298,15\ K}$ Kcal/mol	−4.43

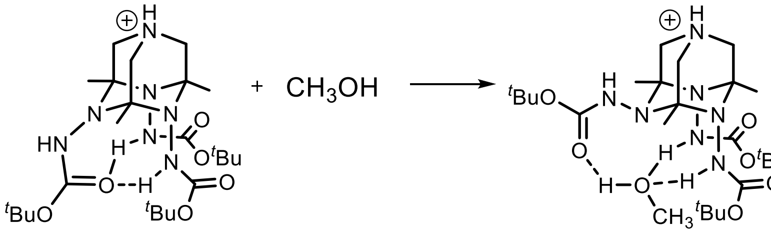

***Ax,ax,ax*-4c•*H*$^+$** **MeOH@TAAD 4c•*H*$^+$**

ΔE_0 Kcal/mol	−12.33
$\Delta H°_{298,15\ K}$ Kcal/mol	−10.91
$\Delta G°_{298,15\ K}$ Kcal/mol	−0.80

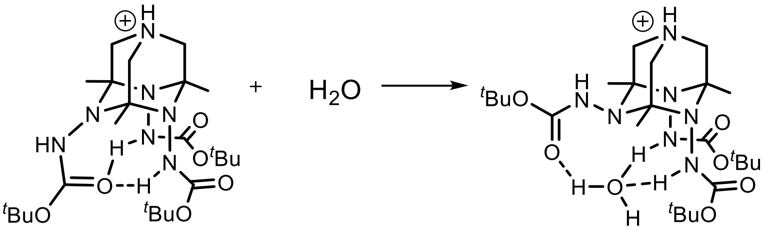

***Ax,ax,ax*-4c•*H*$^+$** **H_2O@TAAD 4c•*H*$^+$**

ΔE_0 Kcal/mol	−10.76
$\Delta H°_{298,15\ K}$ Kcal/mol	−9.05
$\Delta G°_{298,15\ K}$ Kcal/mol	+1.79

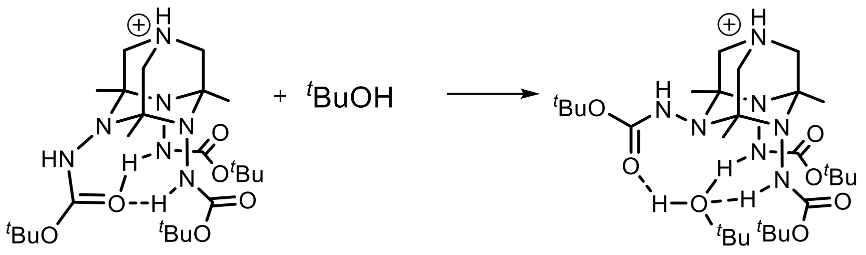

***Ax,ax,ax*-4c•*H*⁺**　　　**^{t}BuOH@TAAD 4c•*H*⁺**

ΔE_0 Kcal/mol	−18.02
$\Delta H°_{298,15\ K}$ Kcal/mol	−16.40
$\Delta G°_{298,15\ K}$ Kcal/mol	−3.73

Rotation across the amide/carbamate C–N bond in TAAD 4a and TAAD 4c·H⁺

The same parameters were calculated for transition state structures with keywords:

# opt=(calcfc,ts,noeigentest) wb97xd nosymm def2tzvp test

IRC calculation was performed for TS and proved that TS connects products and reactants:

# irc=(forward,calcfc,maxcycle=150,MaxPoints=10,ReCorrect=never,HPC) wb97xd nosymm def2tzvp test

# irc=(reverse,calcfc,maxcycle=150,MaxPoints=10,ReCorrect=never,HPC) wb97xd nosymm def2tzvp test

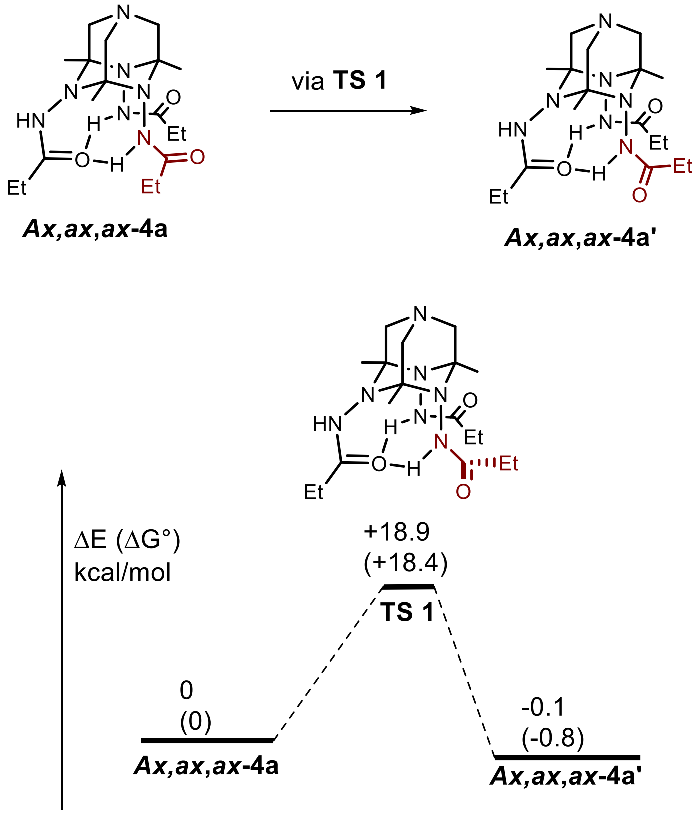

Figure S11. Activation barrier for the rotation across amide C–N bond for **4a**.

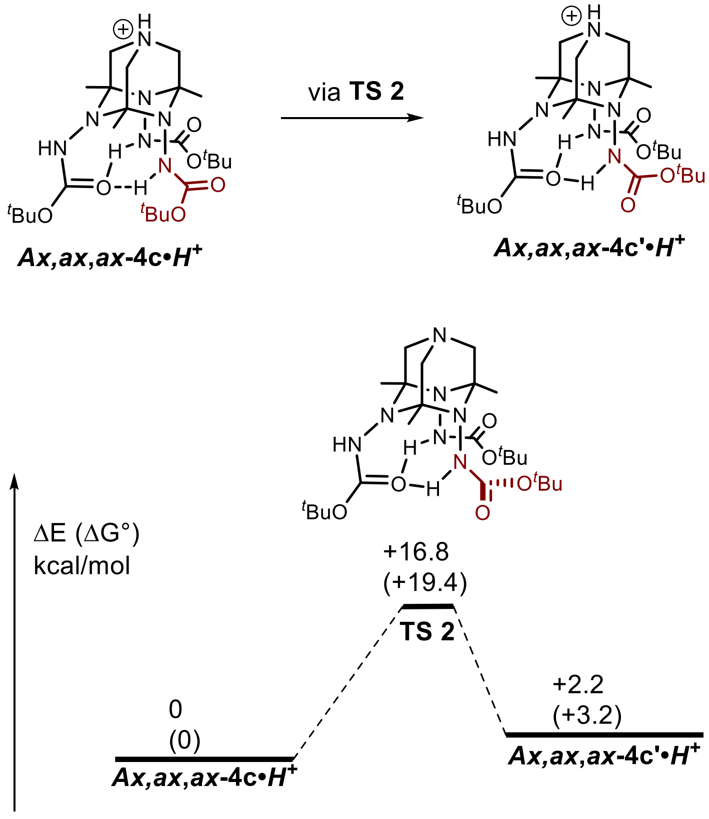

Figure S12. Activation barrier for the rotation across amide C–N bond for **4c·H**$^{+}$.

***ax*,*ax*,*ax*-4a**

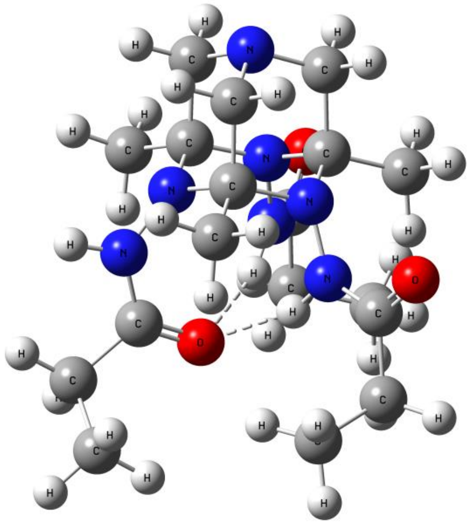

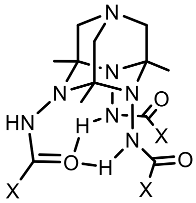

Charge 0; multiplicity 1

N	4.02772800	3.13479200	14.22659300
C	5.02601700	3.77727800	13.38877600
H	5.93552300	3.17290000	13.38468100
H	5.25281700	4.76377800	13.79538100
C	4.53427500	3.94169100	11.93658500
C	5.60446900	4.61050700	11.10382500
H	5.32242300	4.69504000	10.05571700
H	6.53482400	4.04516100	11.16882600
H	5.76626500	5.61599800	11.49008100
C	2.82238700	3.95045300	14.22331700
H	3.05443100	4.93733500	14.62520600
H	2.06416200	3.47613100	14.84726000
C	2.25990900	4.11476800	12.80327000
C	1.01080100	4.96512900	12.84541200
H	0.26616500	4.47445000	13.47150700
H	0.58647100	5.10873900	11.85485100
H	1.25947800	5.94315600	13.25655700
C	3.70541400	1.82403300	13.68827100
H	2.94849200	1.35532500	14.31863000
H	4.60490800	1.20480600	13.68630800
C	3.16280000	1.91497000	12.24757500
C	2.84154600	0.53210100	11.72841900
H	3.72266000	-0.10658800	11.79928000
H	2.50398500	0.54694300	10.69366500
H	2.04286000	0.11044400	12.33745800
O	2.84039200	3.12669700	9.00246000

N	3.32597300	4.77031500	12.01759500
N	1.95833300	2.74635300	12.32961700
N	4.53535800	2.17492400	10.18593000
H	5.36711000	1.61566100	10.09073900
N	4.24628500	2.56350200	11.46948200
N	2.91626500	5.32528500	10.82018700
H	2.71569100	4.69993800	10.04778200
O	3.48875700	7.45799600	11.38268700
C	3.08024600	6.65680200	10.57258300
C	2.72971900	7.03654500	9.14420000
H	1.84472100	6.48891900	8.81126100
H	2.49018400	8.09860100	9.15052300
C	3.89865300	6.75956600	8.19847300
H	4.10868800	5.68972800	8.13869800
H	4.80029500	7.26966500	8.54090900
H	3.67165400	7.10967900	7.19089000
N	1.11754600	2.66548200	11.23726400
H	1.49229700	2.88294500	10.32171100
O	-0.62375800	1.77571000	12.40231600
C	-0.13871400	2.15216800	11.35845400
C	-0.91180000	2.12920400	10.05228000
H	-0.26614800	2.38078600	9.20748100
H	-1.26147200	1.10487700	9.91043900
C	-2.10487900	3.07922000	10.11528800
H	-1.77459300	4.11309800	10.23376400
H	-2.74169700	2.82711500	10.96272200
H	-2.69857300	3.01611500	9.20272600
C	3.86746200	2.45931900	9.04740100
C	4.48450000	1.90292200	7.78508000
H	3.74325900	1.23483400	7.34166000
H	5.37006700	1.30489200	8.00877300
C	4.82588900	3.02302100	6.80594000
H	5.21804800	2.60998300	5.87698200
H	5.57973700	3.69315000	7.22338100
H	3.93725500	3.60964100	6.57595600

DFT ωB97XD / Def2TZVP, gas phase		
Total electronic energy=	-1314.803352	E_0
Sum of electronic and zero-point Energies=	-1314.269302	$E_0 + E_{ZPE}$
Sum of electronic and thermal Energies=	-1314.240907	$E_0 + E_{tot}$
Sum of electronic and thermal Enthalpies=	-1314.239963	$E_0 + H_{corr}$
Sum of electronic and thermal Free Energies=	-1314.328741	$E_0 + G_{corr}$
Zero-point correction *(unscaled)* =	0.534051	
Number of imaginary vibrational frequencies = 0		

***eq,ax,ax*-4a**

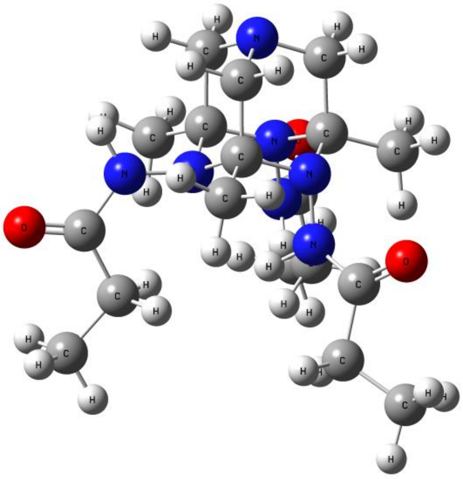

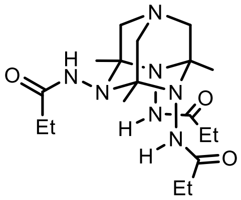

Charge 0; multiplicity 1

N	1.30640700	-0.52413400	2.99314800
C	2.30465300	0.08900500	2.13528600
H	3.16664100	-0.57865900	2.05152200
H	2.64959900	1.02241700	2.58323300
C	1.75071900	0.37862800	0.73521500
C	2.82548300	1.03320700	-0.11327400
H	2.54855200	1.08822100	-1.16475600
H	3.75996700	0.47883700	-0.04599100
H	2.98495700	2.04613200	0.25546800
C	0.17444800	0.38290100	3.09688500
H	0.51054100	1.32616300	3.52945200
H	-0.58486700	-0.05551800	3.74480000
C	-0.46114100	0.65722100	1.72623600
C	-1.63330900	1.60006300	1.88337700
H	-2.39627300	1.12799800	2.50220600
H	-2.07628800	1.86201900	0.92507800
H	-1.28941800	2.51989700	2.35374700
C	0.86867300	-1.77612800	2.39236600
H	0.12738200	-2.25089600	3.03634500
H	1.72673900	-2.44983100	2.31458300
C	0.24689400	-1.55679700	1.00363800
C	-0.21242200	-2.87520700	0.41840800
H	0.58569000	-3.61473300	0.45713900
H	-0.52957300	-2.76553000	-0.61691300
H	-1.06008600	-3.23456300	1.00105000
O	3.18461300	-2.82037200	-2.06660100
N	0.59288900	1.26788800	0.88338300
N	-0.88464300	-0.65454000	1.20786400
N	2.21222300	-1.69934000	-0.39717300

H	2.85236700	-2.17011700	0.22952000
N	1.22700000	-0.86830900	0.09832200
N	0.11855600	1.63613800	-0.37855500
H	0.22331900	0.92439800	-1.09161900
O	0.30668000	3.87541000	-0.02313800
C	0.15709100	2.95064400	-0.78120000
C	-0.02561500	3.11529500	-2.28159000
H	0.96041700	2.96250400	-2.73395800
H	-0.66826700	2.31912400	-2.66768900
C	-0.56885100	4.48298100	-2.65901300
H	0.06889000	5.26952400	-2.25916600
H	-1.56950000	4.63076000	-2.25159100
H	-0.61994700	4.58851500	-3.74294000
N	-1.80334100	-0.62417100	0.17845500
H	-1.61092300	-0.06021200	-0.63358200
O	-3.42772700	-1.70270200	1.34471900
C	-3.07476900	-1.10146700	0.35904400
C	-4.00332100	-0.78930900	-0.80277900
H	-4.43510600	0.19565500	-0.59798000
H	-3.43004500	-0.69097400	-1.72864900
C	-5.10716300	-1.82281600	-0.95757300
H	-5.66090700	-1.92853400	-0.02621800
H	-4.69426800	-2.80013900	-1.21062100
H	-5.79920000	-1.52849200	-1.74700800
C	2.32824500	-2.03820400	-1.71710300
C	1.36150900	-1.37776800	-2.68212400
H	1.57101800	-0.30352200	-2.67980100
H	0.34743900	-1.48212200	-2.29164900
C	1.47612000	-1.94057900	-4.08750000
H	0.78226300	-1.43048800	-4.75687000
H	1.24777300	-3.00597800	-4.09975900
H	2.48671000	-1.82029200	-4.47489100

DFT ωB97XD / Def2TZVP, gas phase		
Total electronic energy=	-1314.795452	E_0
Sum of electronic and zero-point Energies=	-1314.263691	$E_0 + E_{ZPE}$
Sum of electronic and thermal Energies=	-1314.234580	$E_0 + E_{tot}$
Sum of electronic and thermal Enthalpies=	-1314.233636	$E_0 + H_{corr}$
Sum of electronic and thermal Free Energies=	-1314.325709	$E_0 + G_{corr}$
Zero-point correction *(unscaled)* =	0.531761	
Number of imaginary vibrational frequencies = 0		

H_2O@TAAD 4a

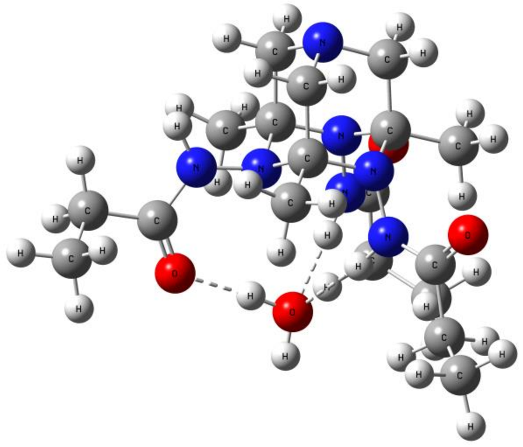

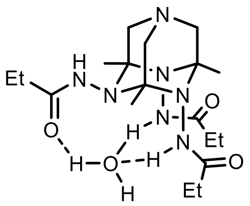

Charge 0; multiplicity 1

N	4.29661300	2.91662500	13.67769000
C	5.11279700	3.57957300	12.67144800
H	5.96984300	2.94156800	12.43272900
H	5.50077000	4.51607400	13.07426200
C	4.30946600	3.88511300	11.39771900
C	5.17525100	4.57760900	10.36386100
H	4.68655800	4.59764600	9.39071800
H	6.13362300	4.06918200	10.25970500
H	5.35631000	5.60123800	10.69028800
C	3.16426700	3.77367700	14.00274000
H	3.53221000	4.71752100	14.40597200
H	2.54448600	3.28048700	14.75216300
C	2.30507000	4.06796900	12.76521300
C	1.14548300	4.96057400	13.14510500
H	0.53175300	4.45322000	13.88824600
H	0.51976200	5.19193800	12.28635100
H	1.53183800	5.89595300	13.54869100
C	3.79189900	1.66314100	13.13775600
H	3.18729000	1.15744200	13.89189400
H	4.63872900	1.01076100	12.90176600
C	2.93363200	1.88796800	11.88387200
C	2.41469000	0.57210300	11.33833100
H	3.21009200	-0.17205700	11.30012500
H	2.00613800	0.69467900	10.33645000
H	1.62665100	0.20939800	11.99748800
O	3.80877700	1.96168200	8.14449700
N	3.19781700	4.73609600	11.80017400
N	1.83108200	2.75616500	12.27654600
N	4.68014100	1.81549200	10.23622500

H	5.43087000	1.43115400	10.79169300
N	3.72807200	2.61803900	10.84007600
N	2.56512700	5.37142700	10.74591000
H	2.20437900	4.82123400	9.97364700
O	3.15121500	7.46156700	11.42937800
C	2.60956100	6.72916000	10.63197600
C	1.86179100	7.24860500	9.41456100
H	1.73316500	6.45001100	8.68013900
H	0.86061100	7.52214300	9.76119700
C	2.54595000	8.45841800	8.79597900
H	3.52507600	8.19081600	8.39494600
H	2.69384900	9.23378200	9.54614100
H	1.94525800	8.86646900	7.98231100
N	0.83006500	2.82171400	11.32189300
H	1.06666900	3.06622000	10.36574500
O	-0.83393200	2.13398700	12.71700700
C	-0.45744200	2.49873000	11.62535500
C	-1.39888100	2.71371800	10.45276700
H	-0.95075400	2.31983100	9.53680600
H	-2.30237600	2.14431600	10.66424400
C	-1.72767900	4.19638600	10.27335800
H	-0.83247000	4.77006500	10.02431400
H	-2.15188700	4.60975900	11.18962600
H	-2.45293500	4.33734300	9.47098500
C	4.65911600	1.54171700	8.91352500
C	5.78974200	0.66629800	8.42267400
H	5.33129300	-0.24004400	8.02156000
H	6.43962400	0.36419100	9.24691300
C	6.59529700	1.36764900	7.33275800
H	7.36876100	0.70488600	6.94526500
H	7.07974000	2.26576500	7.71977400
H	5.94364600	1.65981000	6.51053700
O	1.67637300	3.55517000	8.58806900
H	1.24299600	3.60760700	7.73708600
H	2.46566800	2.97911600	8.47346300

DFT ωB97XD / Def2TZVP, gas phase		
Total electronic energy=	-1391.263491	E_0
Sum of electronic and zero-point Energies=	-1390.704445	$E_0 + E_{ZPE}$
Sum of electronic and thermal Energies=	-1390.673249	$E_0 + E_{tot}$
Sum of electronic and thermal Enthalpies=	-1390.672304	$E_0 + H_{corr}$
Sum of electronic and thermal Free Energies=	-1390.767551	$E_0 + G_{corr}$
Zero-point correction *(unscaled)* =	0.559046	
Number of imaginary vibrational frequencies = 0		

MeOH@TAAD 4a

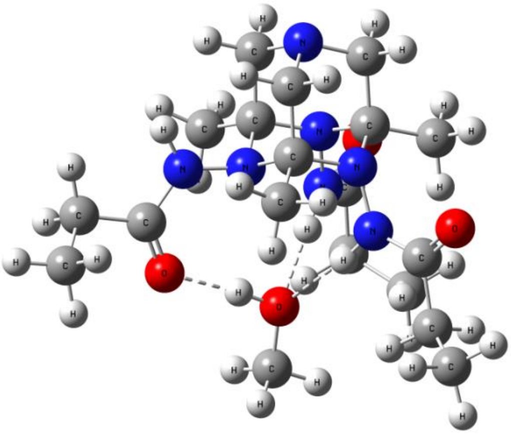

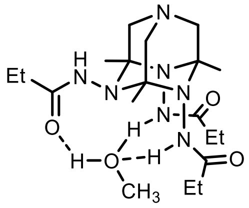

Charge 0; multiplicity 1

7	4.303891000	2.914176000	13.690838000
6	5.116255000	3.583205000	12.685524000
1	5.976647000	2.950115000	12.445708000
1	5.499122000	4.521189000	13.089766000
6	4.311381000	3.886146000	11.412175000
6	5.173610000	4.585383000	10.379966000
1	4.685883000	4.604734000	9.406329000
1	6.135114000	4.082966000	10.275581000
1	5.348056000	5.609543000	10.708350000
6	3.165819000	3.763243000	14.016614000
1	3.527076000	4.708759000	14.421904000
1	2.548944000	3.264771000	14.764963000
6	2.305766000	4.054762000	12.779276000
6	1.141187000	4.940503000	13.160048000
1	0.529397000	4.429196000	13.901918000
1	0.514862000	5.169970000	12.301230000
1	1.522443000	5.877567000	13.564604000
6	3.806864000	1.658673000	13.149088000
1	3.205704000	1.147890000	13.902616000
1	4.657479000	1.011842000	12.911541000
6	2.947718000	1.879802000	11.895560000
6	2.437831000	0.560616000	11.348964000
1	3.237163000	-0.179573000	11.314638000
1	2.031952000	0.679109000	10.345582000
1	1.649135000	0.194834000	12.005598000
8	3.805157000	1.964135000	8.155459000
7	3.194831000	4.730350000	11.815810000
7	1.838012000	2.740559000	12.286867000
7	4.690291000	1.821682000	10.241936000
1	5.445623000	1.439729000	10.792802000
7	3.736779000	2.616917000	10.852606000
7	2.557798000	5.361502000	10.761378000
1	2.204040000	4.809029000	9.987060000
8	3.117855000	7.459591000	11.443951000

6	2.585527000	6.719249000	10.647579000
6	1.829316000	7.227181000	9.430081000
1	1.700740000	6.423987000	8.700460000
1	0.828564000	7.497859000	9.780242000
6	2.504183000	8.436643000	8.800685000
1	3.482436000	8.171313000	8.395987000
1	2.652113000	9.217146000	9.545516000
1	1.897276000	8.836826000	7.987748000
7	0.856651000	2.812861000	11.311159000
1	1.118151000	3.057178000	10.360674000
8	-0.859106000	2.170297000	12.665877000
6	-0.444324000	2.523234000	11.584254000
6	-1.347617000	2.761736000	10.385116000
1	-0.896791000	2.331810000	9.486554000
1	-2.281370000	2.237995000	10.582234000
6	-1.602406000	4.254366000	10.172169000
1	-0.673519000	4.783983000	9.948832000
1	-2.039833000	4.701954000	11.065871000
1	-2.291344000	4.414872000	9.341768000
6	4.661620000	1.547181000	8.919131000
6	5.789467000	0.670940000	8.422822000
1	5.330244000	-0.250310000	8.057548000
1	6.461980000	0.396252000	9.238473000
6	6.561086000	1.349421000	7.295023000
1	7.329645000	0.682436000	6.905023000
1	7.048014000	2.260798000	7.646142000
1	5.886243000	1.615484000	6.482773000
8	1.684643000	3.573564000	8.598735000
1	2.471686000	2.998177000	8.487135000
6	0.945226000	3.615646000	7.400025000
1	0.057154000	4.223490000	7.576370000
1	1.521865000	4.067243000	6.586931000
1	0.626167000	2.616053000	7.088662000

DFT ωB97XD / Def2TZVP, gas phase		
Total electronic energy=	-1430.563817	E_0
Sum of electronic and zero-point Energies=	-1429.975944	$E_0 + E_{ZPE}$
Sum of electronic and thermal Energies=	-1429.943169	$E_0 + E_{tot}$
Sum of electronic and thermal Enthalpies=	-1429.942225	$E_0 + H_{corr}$
Sum of electronic and thermal Free Energies=	-1430.041799	$E_0 + G_{corr}$
Zero-point correction *(unscaled)* =	0.587872	
Number of imaginary vibrational frequencies = 0		

***ax,ax,ax*-4c·H**$^+$

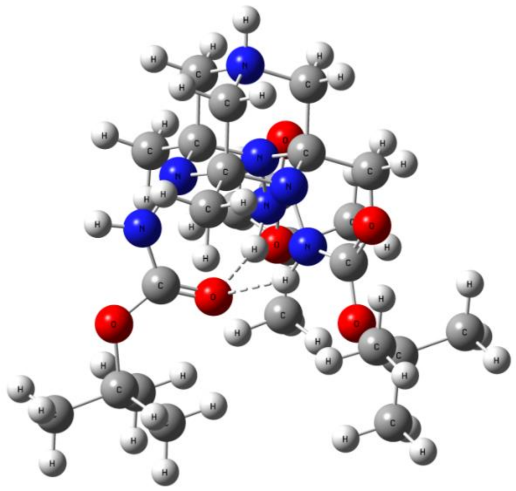

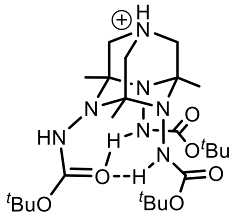

Charge 1; multiplicity 1

O	10.26923900	9.05061900	3.20720200
O	10.22520600	7.47840100	1.56010900
O	7.78157800	4.66181500	3.19765200
O	8.50152600	2.70089300	4.07324700
O	3.38467000	6.92648700	4.15676400
O	3.87827100	5.51729400	2.43740400
N	7.22768800	8.14400600	6.97846100
H	7.15303800	8.74484400	7.79673900
N	8.35126500	7.60550600	4.47539500
N	8.84213900	7.27171000	3.24327700
H	8.59672400	6.37875300	2.83603900
N	7.84807900	5.64051200	5.88103000
N	8.17076800	4.38868000	5.42751300
H	8.46783800	3.72184300	6.11922200
N	6.02490600	6.89339600	4.78670000
N	5.51458300	6.25713200	3.68871700
H	6.10009900	5.61293100	3.17299400
C	6.82693400	8.92440400	5.76486800
H	7.50207500	9.77247400	5.67405600
H	5.79860000	9.25173100	5.90156100
C	6.94229000	8.01238400	4.54180700
C	6.53398100	8.81529100	3.32590400
H	7.19896800	9.67138300	3.21617700
H	5.50451600	9.15254200	3.44239500
H	6.59831600	8.21197700	2.42597400
C	8.64537900	7.68482400	6.84116900
H	8.91084100	7.14512800	7.74781200
H	9.27172300	8.56537800	6.71558000
C	8.75682900	6.77566900	5.60921000
C	10.19833400	6.33150700	5.48367200
H	10.33211100	5.66973900	4.63214000
H	10.51019000	5.80383500	6.38451800
H	10.83034100	7.20609600	5.33432400

C	6.31469000	6.97104600	7.15264400
H	5.29869200	7.34860000	7.24559600
H	6.61748700	6.44337600	8.05467900
C	6.43102900	6.06313800	5.92028900
C	5.49442300	4.88975900	6.11292400
H	4.47165900	5.25681500	6.18918500
H	5.75589000	4.34359600	7.01873400
H	5.54498700	4.20526600	5.27028900
C	9.84328300	8.04313600	2.70093100
C	11.26264300	8.07339500	0.71236500
C	11.34204200	7.09823800	-0.45172000
H	11.62199200	6.10356400	-0.10266400
H	12.09241800	7.43711800	-1.16615200
H	10.38192700	7.03147000	-0.96424100
C	10.81718500	9.44822600	0.23565500
H	9.84042400	9.38470400	-0.24662400
H	11.53269600	9.82123000	-0.49821900
H	10.76480500	10.15667200	1.05922800
C	12.58398500	8.12215200	1.46622200
H	12.54338100	8.82571100	2.29455700
H	13.37417300	8.43556200	0.78280400
H	12.84067600	7.13188200	1.84680500
C	8.12462400	3.95899900	4.13090800
C	8.55212700	1.95071300	2.79974300
C	7.15688400	1.86976200	2.19957700
H	6.45152500	1.46537600	2.92721200
H	7.18018200	1.19417200	1.34391500
H	6.80569800	2.84118800	1.85909800
C	9.03207400	0.58025300	3.24656000
H	10.01244200	0.64829100	3.71853500
H	9.11117500	-0.07821800	2.38167800
H	8.33164600	0.13656100	3.95439500
C	9.55883900	2.60131600	1.86345800
H	9.22123700	3.57661800	1.52041300
H	9.69544200	1.95957200	0.99244700
H	10.52603200	2.70711800	2.35723200
C	4.15493700	6.29066700	3.48205500
C	2.50948800	5.35264500	1.93896400
C	1.64037700	4.71380900	3.01288500
H	2.09471900	3.78672900	3.36720300
H	0.66643500	4.47009300	2.58664000
H	1.49133300	5.38546700	3.85517600
C	1.96186800	6.69388300	1.47320300
H	1.81783300	7.37591500	2.30807700
H	1.00010900	6.53650300	0.98337400
H	2.63925600	7.14978800	0.74938400
C	2.69431700	4.40699700	0.76258900
H	3.35315300	4.84791800	0.01395800
H	1.72954500	4.20396900	0.29749600
H	3.12432200	3.46065600	1.09295100

DFT ωB97XD / Def2TZVP, gas phase		
Total electronic energy=	-1776.830404	E_0
Sum of electronic and zero-point Energies=	-1776.100194	$E_0 + E_{ZPE}$
Sum of electronic and thermal Energies=	-1776.060596	$E_0 + E_{tot}$
Sum of electronic and thermal Enthalpies=	-1776.059652	$E_0 + H_{corr}$
Sum of electronic and thermal Free Energies=	-1776.173799	$E_0 + G_{corr}$
Zero-point correction *(unscaled)* =	0.730210	
Number of imaginary vibrational frequencies = 0		

MeOH@TAAD 4c·H$^+$

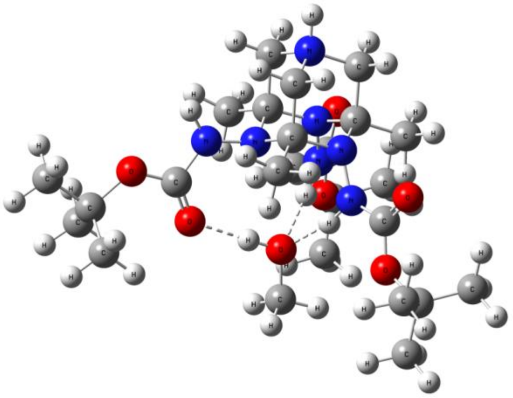

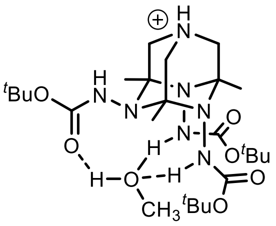

Charge 1; multiplicity 1

O	10.11034400	9.26482900	3.06943700
O	9.69473100	8.16389200	1.12024600
O	8.23764100	3.18158600	3.19974200
O	8.85626800	2.04434400	5.05937000
O	3.34321700	7.17686500	4.01794300
O	3.78837100	6.34540800	1.94557100
N	7.46809800	7.33090900	6.91379800
H	7.50845400	7.63369900	7.88457500
N	8.30517600	7.61883000	4.25360300
N	8.57540700	7.58250700	2.90684300
H	8.22227200	6.81644700	2.33413100
N	7.79425000	5.35782200	4.84559800
N	8.23501600	4.12971900	5.27741700
H	8.43147900	3.94134100	6.24948500
N	5.98453600	6.89908100	4.57518900
N	5.45037300	6.61691900	3.34123500
H	6.01050200	6.13555000	2.63840400
C	6.99695900	8.47398400	6.06879900
H	7.70758600	9.28951500	6.18312300
H	6.00814000	8.76294300	6.41792600
C	6.93788600	8.01481100	4.61320500
C	6.46006800	9.18261000	3.77677900
H	7.16039500	10.01145700	3.87495900
H	5.46691800	9.48517800	4.10718800
H	6.40397300	8.90228600	2.72988000
C	8.83569500	6.90507500	6.47633200
H	9.15406900	6.09283800	7.12993900
H	9.50990400	7.75162100	6.58714800
C	8.77144300	6.46265500	5.00679600
C	10.15808300	6.04236900	4.56074300
H	10.10612100	5.56567900	3.58422800
H	10.60104300	5.33948500	5.26454600

H	10.79174200	6.92567200	4.48937300
C	6.51197400	6.18398600	6.79783900
H	5.53379100	6.51781900	7.13720800
H	6.87370200	5.38520400	7.44546000
C	6.44455100	5.74076600	5.32869900
C	5.46791200	4.58742600	5.20937800
H	4.45634700	4.96004400	5.36659600
H	5.68271300	3.81322400	5.94427300
H	5.53148200	4.14733100	4.21651900
C	9.53698900	8.43167800	2.41354900
C	10.63924700	8.91197100	0.28791500
C	10.46637400	8.26408600	-1.07708500
H	10.71077600	7.20210400	-1.03276600
H	11.12902600	8.74120800	-1.79930900
H	9.43908000	8.37216100	-1.42668400
C	10.24227800	10.38055600	0.24203900
H	9.20280200	10.48293400	-0.07385300
H	10.86909200	10.89815800	-0.48527400
H	10.37020200	10.85678900	1.21141300
C	12.05745700	8.71228300	0.80365300
H	12.19482900	9.17975100	1.77604300
H	12.76089100	9.15941100	0.10010900
H	12.28690900	7.64790500	0.87997800
C	8.43213300	3.09923900	4.39205200
C	9.16260400	0.76157700	4.39289500
C	7.90312600	0.21074300	3.74272300
H	7.09402400	0.14447200	4.47166800
H	8.10681300	-0.79517800	3.37422200
H	7.58260400	0.82553300	2.90478700
C	9.60390000	-0.11480900	5.55291300
H	10.48088300	0.30656500	6.04503600
H	9.86122400	-1.10720000	5.18305700
H	8.80398000	-0.21830400	6.28657100
C	10.29610500	0.96033800	3.39885400
H	9.98846700	1.57755100	2.55790100
H	10.60592500	-0.01308400	3.01726900
H	11.15689800	1.41956200	3.88726800
C	4.09286300	6.75270500	3.17455200
C	2.41472500	6.38350700	1.43919200
C	1.52597100	5.47630900	2.27842100
H	1.94898300	4.47134200	2.32801500
H	0.54292000	5.40586500	1.81109900
H	1.40374300	5.86445400	3.28704100
C	1.91122000	7.81950600	1.40982900
H	1.79393500	8.21959400	2.41436800
H	0.94299900	7.84919700	0.90836700
H	2.60163300	8.45227500	0.84969600
C	2.56411000	5.83570900	0.02839800
H	3.23456000	6.46389900	-0.55905000
H	1.59187100	5.81312300	-0.46416300
H	2.96434800	4.82139300	0.04981100
O	7.37194900	5.28678100	1.67309700

H	7.67968000	4.52718100	2.20065800
C	7.29180700	4.93878400	0.30229300
H	6.95236300	5.81945700	-0.24018600
H	6.57596100	4.12908400	0.14047600
H	8.26789000	4.64060600	-0.08827600

DFT ωB97XD / Def2TZVP, gas phase		
Total electronic energy=	-1892.584994	E_0
Sum of electronic and zero-point Energies=	-1891.801006	$E_0 + E_{ZPE}$
Sum of electronic and thermal Energies=	-1891.756959	$E_0 + E_{tot}$
Sum of electronic and thermal Enthalpies=	-1891.756015	$E_0 + H_{corr}$
Sum of electronic and thermal Free Energies=	-1891.881075	$E_0 + G_{corr}$
Zero-point correction *(unscaled)* =	0.783987	
Number of imaginary vibrational frequencies = 0		

H_2O@TAAD 4c·H^+

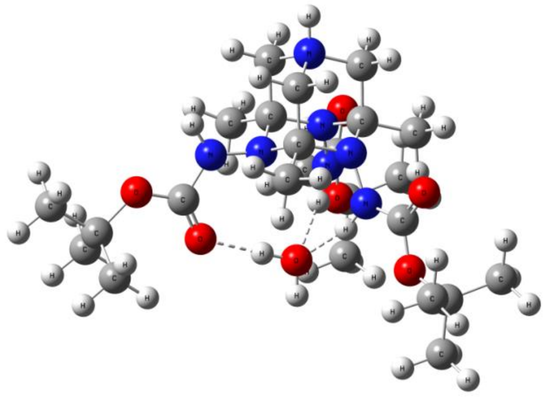

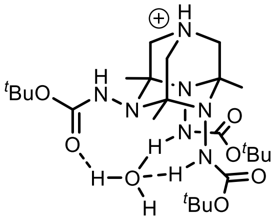

Charge 1; multiplicity 1

O	10.10579900	9.26057100	3.07701500
O	9.73519600	8.19135500	1.10211800
O	8.25775900	3.13116800	3.20788300
O	8.87024700	2.02255200	5.08761200
O	3.34821700	7.17953900	4.02509800
O	3.73080700	6.34771300	1.94124700
N	7.46048200	7.32261200	6.86397600
H	7.49676900	7.63550000	7.83169700
N	8.30621100	7.58401700	4.20592200
N	8.60075500	7.55879500	2.86534500
H	8.25414100	6.80427300	2.27663800
N	7.79774800	5.32794600	4.81823200
N	8.23907000	4.10769100	5.27159700
H	8.43202000	3.93472000	6.24723500
N	5.98743300	6.86442800	4.52648700
N	5.42839900	6.57938700	3.30581800
H	5.97186100	6.10200100	2.58983800
C	6.99057500	8.45659800	6.00591300
H	7.69980500	9.27414900	6.11451500
H	5.99989900	8.74710800	6.34847800
C	6.93756300	7.98207700	4.55454100
C	6.46090400	9.14015500	3.70419800
H	7.15987200	9.97084200	3.79610900
H	5.46659800	9.44509700	4.02885300
H	6.40787900	8.84942500	2.65987500
C	8.83050000	6.89432200	6.43625300
H	9.14761300	6.08977400	7.09992000
H	9.50293500	7.74312200	6.54026200
C	8.77238200	6.43674500	4.97074900
C	10.16127900	6.01460000	4.53406000
H	10.11446900	5.53168100	3.56038900
H	10.60091800	5.31669600	5.24483300
H	10.79506900	6.89761600	4.46059500
C	6.50682800	6.17261400	6.75678300

H	5.52672200	6.50817900	7.08877600
H	6.86780700	5.38164600	7.41435300
C	6.44521300	5.71424700	5.29168600
C	5.47064500	4.55856300	5.18057800
H	4.45839300	4.93021600	5.33553900
H	5.68699000	3.79045800	5.92133800
H	5.53493200	4.11138400	4.19097000
C	9.55513800	8.43127800	2.39686500
C	10.67879100	8.97315000	0.29854500
C	10.53367300	8.35475800	-1.08314800
H	10.79545500	7.29629900	-1.06088300
H	11.19752000	8.85985200	-1.78497100
H	9.50938900	8.45451100	-1.44369000
C	10.25949900	10.43613300	0.28077600
H	9.22233700	10.52969400	-0.04528200
H	10.88658600	10.97904700	-0.42757700
H	10.36903200	10.89262700	1.26176900
C	12.09333600	8.78310800	0.82769900
H	12.21324800	9.23224200	1.81091500
H	12.79824900	9.25489800	0.14196500
H	12.33750800	7.72084200	0.88454700
C	8.44575400	3.06574400	4.40241500
C	9.18731000	0.73030700	4.44514200
C	7.93543900	0.16208700	3.79543600
H	7.12082600	0.10590200	4.51904500
H	8.14636100	-0.84968800	3.44760000
H	7.61863700	0.75912600	2.94346300
C	9.62385200	-0.12406300	5.62335000
H	10.49484800	0.31005200	6.11501100
H	9.88898000	-1.12126800	5.27246800
H	8.81873000	-0.21913400	6.35243400
C	10.32763000	0.91724300	3.45674900
H	10.02442100	1.51806300	2.60249300
H	10.64497400	-0.06118600	3.09465400
H	11.18239500	1.38893000	3.94384000
C	4.07024900	6.74299300	3.16380600
C	2.34674600	6.42213500	1.46522900
C	1.45543800	5.52931800	2.31701500
H	1.85676000	4.51492000	2.35150400
H	0.46197700	5.48385800	1.86901700
H	1.36141100	5.91287600	3.33032000
C	1.87716000	7.86986600	1.45748400
H	1.79093600	8.26479800	2.46715600
H	0.89936800	7.92600100	0.97736000
H	2.56999300	8.49047600	0.88683300
C	2.45117000	5.88233200	0.04743900
H	3.12350400	6.49850500	-0.55037600
H	1.46769000	5.88814300	-0.42278600
H	2.82581600	4.85814000	0.05183800
O	7.38776200	5.21431100	1.65097000
H	7.69790100	4.46271200	2.19179700
H	7.35391000	4.92199300	0.74034500

DFT ωB97XD / Def2TZVP, gas phase		
Total electronic energy=	-1853.285246	E_0
Sum of electronic and zero-point Energies=	-1852.529461	$E_0 + E_{ZPE}$
Sum of electronic and thermal Energies=	-1852.487321	$E_0 + E_{tot}$
Sum of electronic and thermal Enthalpies=	-1852.486377	$E_0 + H_{corr}$
Sum of electronic and thermal Free Energies=	-1852.605307	$E_0 + G_{corr}$
Zero-point correction *(unscaled)* =	0.755785	
Number of imaginary vibrational frequencies = 0		

_t_-BuOH@TAAD 4c·H$^+$

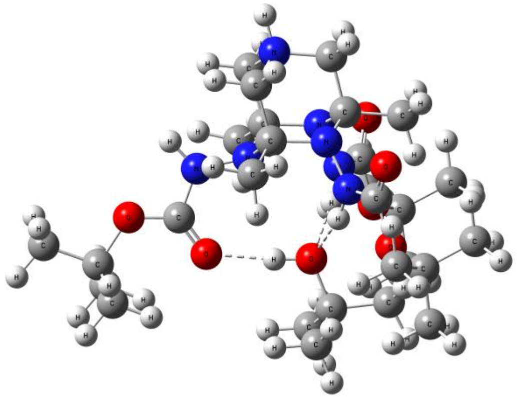

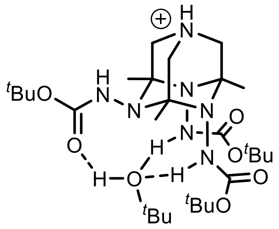

Charge 1; multiplicity 1

O	10.09109200	9.25495600	3.04120700
O	9.69819800	8.11250800	1.11083100
O	8.25466300	3.14071200	3.23081400
O	8.86974800	2.01847700	5.10024600
O	3.35648200	7.17538600	3.98289700
O	3.81579400	6.29655700	1.93283700
N	7.47398100	7.31943400	6.91185200
H	7.51267800	7.63029100	7.88010800
N	8.31579400	7.58803800	4.25239100
N	8.58951000	7.53999600	2.90637200
H	8.23591000	6.76555800	2.34256200
N	7.80474200	5.33044000	4.86160800
N	8.24582700	4.10541300	5.30154500
H	8.44094900	3.92359400	6.27516800
N	5.99255100	6.86789500	4.57674900
N	5.46373200	6.57217600	3.34320600
H	6.03238900	6.08402600	2.64965700
C	7.00280700	8.45558800	6.05702300
H	7.71261600	9.27258400	6.16580700
H	6.01327400	8.74629800	6.40265400
C	6.94637400	7.98421000	4.60533300
C	6.46887400	9.14250500	3.75570800
H	7.16794700	9.97326600	3.84606800
H	5.47439500	9.44700000	4.08021800
H	6.41585100	8.84964300	2.71178400
C	8.84296000	6.89096600	6.48036100
H	9.16034900	6.08407100	7.14104500
H	9.51628200	7.73885800	6.58601100
C	8.78090900	6.43683100	5.01426800
C	10.16727600	6.01323600	4.57064800
H	10.11346700	5.52855100	3.59803900

H	10.61117000	5.31643300	5.27988900
H	10.80081100	6.89594900	4.49119500
C	6.51899400	6.17039200	6.80454600
H	5.54031900	6.50605700	7.14062400
H	6.88126500	5.37741000	7.45896800
C	6.45407300	5.71545500	5.33888900
C	5.47935400	4.56003700	5.22441600
H	4.46663700	4.93236300	5.37485600
H	5.69213300	3.79142100	5.96573900
H	5.54801200	4.11356500	4.23461200
C	9.53329900	8.39886100	2.40190400
C	10.59636500	8.89166700	0.25641200
C	10.43946500	8.21676100	-1.09747900
H	10.75023700	7.17253500	-1.04737800
H	11.05858100	8.72446300	-1.83722700
H	9.40158400	8.25590900	-1.43040500
C	10.12664900	10.33796800	0.19446200
H	9.08070200	10.38414100	-0.11363100
H	10.72088300	10.87657700	-0.54476300
H	10.23876800	10.83304400	1.15636000
C	12.02859000	8.76840400	0.75644400
H	12.15579200	9.25871000	1.71877700
H	12.70121500	9.23496600	0.03554000
H	12.30855800	7.71747300	0.84845800
C	8.44595700	3.06851700	4.42372000
C	9.17753200	0.73306000	4.44073800
C	7.91953300	0.17825200	3.79069900
H	7.10894900	0.11541000	4.51827800
H	8.12430700	-0.82939700	3.42756600
H	7.60016600	0.78897200	2.94933700
C	9.61703900	-0.13800900	5.60546900
H	10.49297300	0.28597400	6.09721300
H	9.87529100	-1.13208100	5.24080600
H	8.81586200	-0.23816400	6.33821400
C	10.31292200	0.92680300	3.44760600
H	10.00683800	1.53969400	2.60292100
H	10.62386900	-0.04849100	3.07177000
H	11.17257400	1.38911500	3.93508300
C	4.11249800	6.72433700	3.15961500
C	2.45786600	6.38465400	1.39235800
C	1.50984800	5.53098200	2.22242800
H	1.88964000	4.51077500	2.30142200
H	0.53779800	5.49197500	1.72903100
H	1.37704000	5.94220000	3.22036800
C	2.02039700	7.84090700	1.32777200
H	1.90071600	8.26346200	2.32282200
H	1.06493700	7.90555700	0.80568400
H	2.75032700	8.43187200	0.77185700
C	2.61628700	5.80790800	-0.00597500
H	3.33880600	6.38665800	-0.58268900
H	1.65899500	5.83357500	-0.52676900
H	2.95560000	4.77236700	0.03904100

O	7.38204600	5.27345800	1.71998300
H	7.69337500	4.48709700	2.20402900
C	7.26801900	5.00176800	0.31286200
C	8.64212700	4.61717200	-0.22012700
C	6.25491000	3.88160000	0.11526100
C	6.78025200	6.30208700	-0.30673800
H	9.35399700	5.42126300	-0.02658600
H	9.00068800	3.70653700	0.26510500
H	8.60547400	4.43667400	-1.29549900
H	5.29356500	4.17042900	0.54322400
H	6.11321100	3.66822000	-0.94528400
H	6.59560900	2.96570000	0.60344400
H	6.66281700	6.19027400	-1.38512700
H	5.81492300	6.58339200	0.11851400
H	7.49889300	7.10204600	-0.11858000

DFT ωB97XD / Def2TZVP, gas phase		
Total electronic energy=	-2010.557488	E_0
Sum of electronic and zero-point Energies=	-2009.688493	$E_0 + E_{ZPE}$
Sum of electronic and thermal Energies=	-2009.641110	$E_0 + E_{tot}$
Sum of electronic and thermal Enthalpies=	-2009.640166	$E_0 + H_{corr}$
Sum of electronic and thermal Free Energies=	-2009.770646	$E_0 + G_{corr}$
Zero-point correction *(unscaled)* =	0.868995	
Number of imaginary vibrational frequencies = 0		

H_2O

Charge 0; multiplicity 1

O	1.873154000	3.795652000	8.547484000
H	1.494447000	3.849410000	7.669683000
H	2.602583000	3.179897000	8.472488000

DFT ωB97XD / Def2TZVP, gas phase		
Total electronic energy=	-76.437692	E_0
Sum of electronic and zero-point Energies=	-76.416084	$E_0 + E_{ZPE}$
Sum of electronic and thermal Energies=	-76.413248	$E_0 + E_{tot}$
Sum of electronic and thermal Enthalpies=	-76.412304	$E_0 + H_{corr}$
Sum of electronic and thermal Free Energies=	-76.434366	$E_0 + G_{corr}$
Zero-point correction *(unscaled)* =	0.021608	
Number of imaginary vibrational frequencies = 0		

CH_3OH

Charge 0; multiplicity 1

O	7.375184000	5.273774000	1.668965000
H	7.677474000	4.518182000	2.172451000
C	7.294790000	4.909008000	0.309221000
H	6.950247000	5.786059000	-0.236593000
H	6.579120000	4.096985000	0.139378000
H	8.267366000	4.612110000	-0.098625000

DFT ωB97XD / Def2TZVP, gas phase		
Total electronic energy=	-115.734946	E_0
Sum of electronic and zero-point Energies=	-115.683254	$E_0 + E_{ZPE}$
Sum of electronic and thermal Energies=	-115.679918	$E_0 + E_{tot}$
Sum of electronic and thermal Enthalpies=	-115.678974	$E_0 + H_{corr}$
Sum of electronic and thermal Free Energies=	-115.706004	$E_0 + G_{corr}$
Zero-point correction *(unscaled)* =	0.051692	
Number of imaginary vibrational frequencies = 0		

t-BuOH

Charge 0; multiplicity 1

O	7.37755500	5.29187900	1.72820100
H	7.68433200	4.50069300	2.17553400
C	7.26934100	5.00695300	0.33328100
C	8.63828300	4.61582900	-0.21851600
C	6.25922400	3.88274600	0.11579900
C	6.78161500	6.29994400	-0.30222600
H	9.36129700	5.41052100	-0.03012700
H	9.00022400	3.70096800	0.25893300
H	8.59152400	4.43415800	-1.29401400
H	5.29316400	4.15701300	0.54156500
H	6.12562300	3.67432200	-0.94750100
H	6.59794500	2.96073700	0.59652600
H	6.66689300	6.17959200	-1.38057100
H	5.81889900	6.58820200	0.12169800
H	7.49398700	7.10415800	-0.11410000

DFT ωB97XD / Def2TZVP, gas phase		
Total electronic energy=	-233.698369	E_0
Sum of electronic and zero-point Energies=	-233.561982	$E_0 + E_{ZPE}$
Sum of electronic and thermal Energies=	-233.555315	$E_0 + E_{tot}$
Sum of electronic and thermal Enthalpies=	-233.554371	$E_0 + H_{corr}$
Sum of electronic and thermal Free Energies=	-233.590909	$E_0 + G_{corr}$
Zero-point correction *(unscaled)* =	0.136387	
Number of imaginary vibrational frequencies = 0		

ax**,*****ax*****,*****ax*****-4a'** (conformer of ***ax*****,*****ax*****,*****ax*****-4a**)

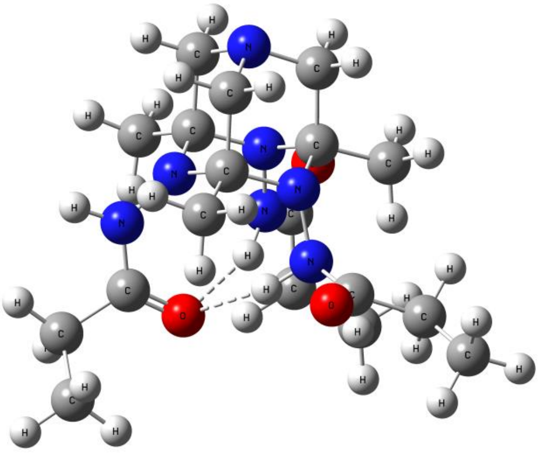

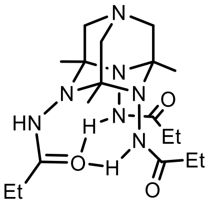

Charge 0; multiplicity 1

N	-1.00692100	0.13102000	-3.01459600
C	-2.05906400	0.56380800	-2.11161300
H	-2.95840200	-0.02725600	-2.29626400
H	-2.28044300	1.61581600	-2.30143600
C	-1.65217900	0.39545900	-0.63376900
C	-2.78350800	0.83855400	0.26703600
H	-2.53501500	0.74462600	1.32228600
H	-3.67449400	0.24479400	0.06098400
H	-3.01206500	1.88440100	0.06605700
C	0.18288000	0.92714100	-2.75612000
H	-0.04272200	1.97893200	-2.94000000
H	0.98267900	0.61055200	-3.42631600
C	0.66551200	0.76550800	-1.30650900
C	1.91626100	1.58847100	-1.09187900
H	2.71983000	1.17100800	-1.69782800
H	2.22921200	1.58640800	-0.05078800
H	1.73373600	2.61874700	-1.39724100
C	-0.69510700	-1.26825200	-2.76915400
H	0.10275200	-1.57944500	-3.44462900
H	-1.58406700	-1.87285100	-2.96023700
C	-0.23600000	-1.50300500	-1.31594100
C	0.07887400	-2.96567900	-1.10266500
H	-0.78689400	-3.57510700	-1.36371400
H	0.36022800	-3.18115000	-0.07369400
H	0.91559800	-3.23695800	-1.74530800
O	-0.07462700	-1.12033400	2.09851000
N	-0.45383700	1.23690800	-0.44994500
N	0.95571900	-0.66981000	-1.13874700
N	-1.73234000	-1.71934100	0.66352200
H	-2.57204700	-2.26863500	0.58381100

N	-1.36755000	-1.04765200	-0.47525100
N	-0.12376900	1.48363100	0.87252000
H	0.03064900	0.70186200	1.50232100
O	-0.05912600	2.85403400	2.63589500
C	-0.23689500	2.71836200	1.44237100
C	-0.56221800	3.87758200	0.52060000
H	0.22423600	3.93987300	-0.23496800
H	-1.47066300	3.63998100	-0.03614900
C	-0.70177500	5.18963800	1.27139300
H	0.21572700	5.43356800	1.80564200
H	-1.50088200	5.13479700	2.01046600
H	-0.92656100	6.00056100	0.57708700
N	1.75497100	-0.99676800	-0.06197500
H	1.33867700	-1.00065800	0.86175600
O	3.55381700	-1.56312300	-1.33526700
C	3.02532000	-1.45172800	-0.25048700
C	3.75282900	-1.76682900	1.04270400
H	3.06376800	-1.77459600	1.89030200
H	4.16566500	-2.77149100	0.93664700
C	4.87994200	-0.76491700	1.28230100
H	4.48302500	0.24325000	1.41551600
H	5.56216100	-0.75469500	0.43253700
H	5.44423800	-1.02583200	2.17808800
C	-1.11346300	-1.72214300	1.86719200
C	-1.81752200	-2.50386000	2.95024800
H	-1.08343300	-3.19185700	3.37238300
H	-2.63476700	-3.10186000	2.54152200
C	-2.33198800	-1.55910300	4.03595400
H	-2.78059700	-2.12608100	4.85124000
H	-3.08858300	-0.88027000	3.63843700
H	-1.51482800	-0.96045300	4.43647500

DFT ωB97XD / Def2TZVP, gas phase		
Total electronic energy=	-1314.803652	E_0
Sum of electronic and zero-point Energies=	-1314.270074	$E_0 + E_{ZPE}$
Sum of electronic and thermal Energies=	-1314.241592	$E_0 + E_{tot}$
Sum of electronic and thermal Enthalpies=	-1314.240648	$E_0 + H_{corr}$
Sum of electronic and thermal Free Energies=	-1314.330091	$E_0 + G_{corr}$
Zero-point correction *(unscaled)* =	0.533578	
Number of imaginary vibrational frequencies = 0		

TS 1 (calculations of rotation around C–N bond)

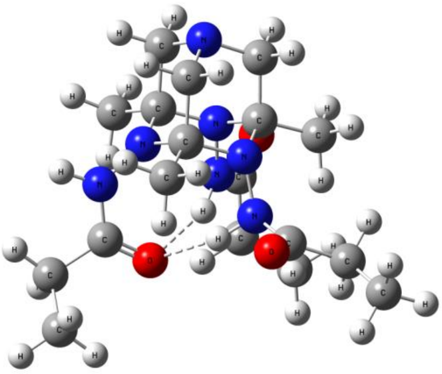

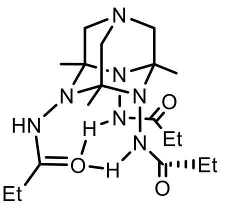

Charge 0; multiplicity 1

N	6.57212000	-3.36113700	8.77898000
C	5.58027700	-2.90115500	9.73478700
H	4.69770100	-3.54178400	9.67352500
H	5.29205600	-1.87878900	9.48465400
C	6.10651300	-2.93674000	11.18526300
C	4.99812700	-2.51525000	12.12204000
H	5.30168400	-2.52315600	13.16639900
H	4.14686000	-3.18613100	11.99742700
H	4.69308900	-1.49933900	11.88541900
C	7.73533700	-2.49742900	8.88273400
H	7.44613200	-1.47386100	8.63956000
H	8.49732000	-2.82852600	8.17625800
C	8.33518000	-2.51992500	10.29611800
C	9.55845700	-1.63010500	10.32022300
H	10.30508100	-2.03760500	9.63954000
H	9.98832000	-1.57028200	11.31556300
H	9.28189900	-0.62561400	9.99690900
C	6.97871000	-4.71993400	9.09938500
H	7.73098800	-5.04538700	8.37918300
H	6.11122600	-5.38022400	9.03060400
C	7.56291400	-4.82087900	10.52098300
C	7.96817300	-6.24746100	10.81504200
H	7.12495900	-6.92043800	10.65461500
H	8.32595100	-6.36998600	11.83526900
H	8.77728500	-6.52071600	10.13888200
O	7.69854400	-4.22058800	13.99933000
N	7.27579300	-2.02725000	11.21842400
N	8.72359300	-3.92541500	10.55326500
N	6.14246200	-5.01874900	12.55376400
H	5.34013700	-5.62292300	12.47302100
N	6.50634700	-4.33724600	11.43015400
N	7.79787600	-1.72591800	12.49994100
H	7.61322800	-2.45188000	13.18922800

O	6.31045100	-0.09691500	13.27154500
C	7.42760900	-0.41185800	12.96923800
C	8.59998600	0.52475700	13.06628700
H	9.36191300	0.01431000	13.66448700
H	9.03277900	0.58422100	12.06342300
C	8.26474900	1.89684500	13.61768000
H	7.85235300	1.82397000	14.62423400
H	7.52061800	2.39466200	12.99592400
H	9.15721900	2.52238400	13.65541700
N	9.50734400	-4.09923000	11.68036500
H	9.07526600	-3.97445800	12.58818400
O	11.44257500	-4.52487200	10.55677900
C	10.83820800	-4.36410200	11.59472500
C	11.51430700	-4.37055600	12.95473000
H	10.87560200	-4.86077400	13.69412100
H	12.42996900	-4.95108400	12.85358200
C	11.82974300	-2.94429800	13.40782200
H	10.91303600	-2.36362400	13.53224100
H	12.45639400	-2.43840200	12.67174700
H	12.35940300	-2.94895500	14.36111900
C	6.73631300	-4.93845300	13.76778100
C	6.11804100	-5.79791900	14.84556700
H	6.87977600	-6.52263000	15.14183400
H	5.26720500	-6.36226700	14.45801600
C	5.70412800	-4.95707200	16.04978000
H	5.31797200	-5.59494000	16.84451000
H	4.92568100	-4.24174000	15.77920600
H	6.55831800	-4.40137700	16.43406700

DFT ωB97XD / Def2TZVP, gas phase		
Total electronic energy=	-1314.773231	E_0
Sum of electronic and zero-point Energies=	-1314.241042	$E_0 + E_{ZPE}$
Sum of electronic and thermal Energies=	-1314.213253	$E_0 + E_{tot}$
Sum of electronic and thermal Enthalpies=	-1314.212309	$E_0 + H_{corr}$
Sum of electronic and thermal Free Energies=	-1314.299414	$E_0 + G_{corr}$
Zero-point correction *(unscaled)* =	0.532189	
Number of imaginary vibrational frequencies = 1; i107		

***ax*,*ax*,*ax*-4c'·H$^+$** (conformer of ***ax*,*ax*,*ax*-4c·H$^+$**)

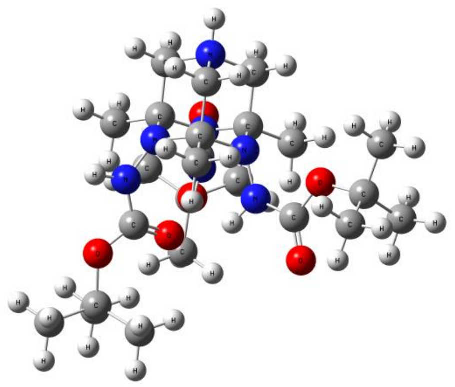

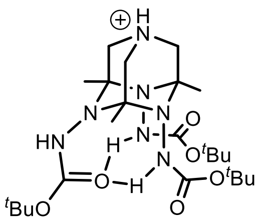

Charge 1; multiplicity 1

O	2.67177600	0.90837000	-2.63880200
O	2.54519700	2.70139900	-1.24571900
O	0.41192400	-1.76789900	-0.42017700
O	1.37415200	-3.48372300	0.70251100
O	-4.02388900	0.48663900	0.37523700
O	-3.59912000	-1.15014100	-1.14979100
N	-0.22235000	2.24658100	2.77429000
H	-0.32851500	2.95609000	3.49640700
N	0.89922800	1.42433800	0.33873600
N	1.34396300	0.85880300	-0.83103000
H	1.10420000	-0.10294400	-1.03977300
N	0.56959200	-0.33933000	2.04216000
N	0.98427400	-1.61825800	1.78043400
H	1.36622300	-2.13601200	2.55332000
N	-1.35934800	0.59776700	0.83149900
N	-1.90965600	-0.20310000	-0.12770500
H	-1.33581800	-0.90539000	-0.57542500
C	-0.70173200	2.80449500	1.47019400
H	-0.09633200	3.67821500	1.23858300
H	-1.75016200	3.07124800	1.58376700
C	-0.54024700	1.72976100	0.39308300
C	-1.03457900	2.30776600	-0.91529000
H	-0.43920100	3.18355300	-1.17170200
H	-2.08398100	2.58436600	-0.81881200
H	-0.94166000	1.58288800	-1.71778400
C	1.22319100	1.87824900	2.66239700
H	1.54868800	1.50066100	3.62937500
H	1.77598200	2.77535600	2.39202500
C	1.38222900	0.80488800	1.57683700
C	2.85094300	0.45194100	1.47704700
H	3.01908700	-0.32016500	0.73137200
H	3.22051300	0.09347300	2.43739100
H	3.41189400	1.33655600	1.17813500

C	-1.03850200	1.04875400	3.14957000
H	-2.07881600	1.36033000	3.21460900
H	-0.67822700	0.68868100	4.11108200
C	-0.87475300	-0.02275600	2.06189800
C	-1.71395300	-1.21977800	2.45330500
H	-2.75915400	-0.91964500	2.51752800
H	-1.38648900	-1.60938300	3.41658500
H	-1.63550800	-2.01263700	1.71421900
C	2.24735600	1.47437800	-1.66324200
C	3.47312600	3.55100600	-1.99616400
C	3.50144700	4.82429200	-1.16422900
H	2.50507400	5.26406800	-1.09919200
H	4.16853900	5.55356400	-1.62371300
H	3.86278600	4.61758400	-0.15545800
C	4.85208900	2.90826100	-2.04098200
H	5.19528100	2.67177600	-1.03179900
H	5.56084400	3.61095800	-2.48090600
H	4.84676200	1.99977800	-2.63816300
C	2.91904300	3.83005700	-3.38585400
H	2.90091200	2.92927700	-3.99433600
H	3.54806900	4.57166800	-3.87988100
H	1.90818200	4.23624400	-3.31801400
C	0.88725800	-2.27008600	0.58150000
C	1.42787900	-4.42943000	-0.43584300
C	0.01645200	-4.72941500	-0.91625400
H	-0.60731800	-5.06250000	-0.08520700
H	0.05859700	-5.53647600	-1.64834700
H	-0.44284400	-3.86388400	-1.38813200
C	2.06123800	-5.65916600	0.19215000
H	3.05354900	-5.42810400	0.57993900
H	2.15958300	-6.44197100	-0.55975000
H	1.44500900	-6.03963000	1.00712500
C	2.31441000	-3.85753800	-1.53073900
H	1.87101100	-2.98136000	-1.99815600
H	2.45950300	-4.61793300	-2.29874000
H	3.29462400	-3.59419300	-1.13073900
C	-3.28042800	-0.22863700	-0.24964800
C	-4.99005600	-1.40287700	-1.54083400
C	-5.78442300	-1.89325200	-0.33879200
H	-5.29104000	-2.75352700	0.11706800
H	-6.77452900	-2.20929700	-0.66959400
H	-5.90232400	-1.11000400	0.40652400
C	-5.59011900	-0.14860200	-2.15905800
H	-5.70211500	0.64311200	-1.42172500
H	-6.57393400	-0.38641500	-2.56545900
H	-4.96350100	0.20815400	-2.97799700
C	-4.84974200	-2.50362400	-2.58026300
H	-4.24281800	-2.16510900	-3.42039100
H	-5.83420800	-2.78365400	-2.95540400
H	-4.38192700	-3.38739200	-2.14492300

DFT ωB97XD / Def2TZVP, gas phase		
Total electronic energy=	-1776.826866	E_0
Sum of electronic and zero-point Energies=	-1776.096118	$E_0 + E_{ZPE}$
Sum of electronic and thermal Energies=	-1776.056780	$E_0 + E_{tot}$
Sum of electronic and thermal Enthalpies=	-1776.055835	$E_0 + H_{corr}$
Sum of electronic and thermal Free Energies=	-1776.168644	$E_0 + G_{corr}$
Zero-point correction *(unscaled)* =	0.730747	
Number of imaginary vibrational frequencies = 0		

TS 2 (calculations of rotation around C–N bond)

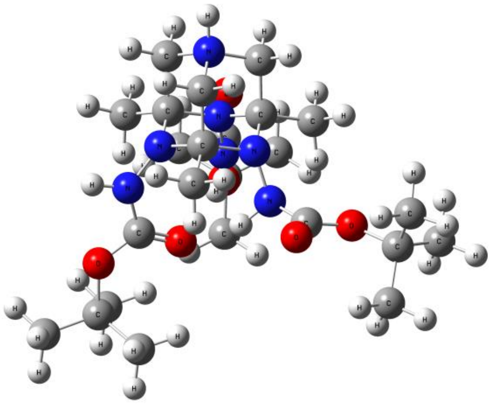

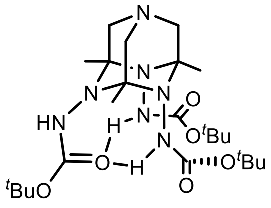

Charge 1; multiplicity 1

O	10.82593000	7.87720300	2.84272700
O	9.20814900	8.83016000	1.60497500
O	7.79838800	4.58941900	3.17659700
O	8.46516300	2.65985000	4.16113800
O	3.36833000	6.84806400	4.12493900
O	3.99454000	5.53790400	2.37083500
N	7.25311500	8.12325200	7.04176400
H	7.20156900	8.67551900	7.89525700
N	8.30698200	7.74439300	4.44495700
N	8.57445600	7.32868500	3.13457200
H	8.56790800	6.32313000	2.99768200
N	7.86166400	5.70776600	5.77469100
N	8.21658600	4.44223700	5.41009200
H	8.50125900	3.81154400	6.14039500
N	5.99551500	6.97070700	4.81653300
N	5.54959700	6.34865100	3.67883300
H	6.18155600	5.75597100	3.15540100
C	6.79958900	8.95747700	5.88812700
H	7.45395300	9.82391800	5.82382000
H	5.77174000	9.26109000	6.07315300
C	6.88399100	8.12083500	4.61282500
C	6.39509900	8.99108000	3.47358400
H	7.00909800	9.88898300	3.40741600
H	5.35432000	9.26292800	3.64877600
H	6.47467700	8.46123400	2.53140700
C	8.67128500	7.70300100	6.81802400
H	8.98818800	7.11914800	7.67968500
H	9.27338100	8.60419200	6.72465400
C	8.75489200	6.85663100	5.53894800
C	10.19166700	6.40995600	5.38087300
H	10.32717700	5.81665300	4.48133000
H	10.48837100	5.81139500	6.24205000

H	10.84048300	7.27710000	5.28920100
C	6.35675900	6.93294200	7.17943400
H	5.34207700	7.29618500	7.32868300
H	6.69284700	6.35855700	8.04029500
C	6.44641300	6.09341700	5.89736500
C	5.53219100	4.89714600	6.05620000
H	4.50620000	5.24682900	6.16353500
H	5.81430800	4.31526700	6.93312900
H	5.57772300	4.25073300	5.18358100
C	9.67393500	8.02241000	2.53501200
C	10.09282200	9.68753400	0.80064900
C	9.11274300	10.41073400	-0.10921700
H	8.56105600	9.69902900	-0.72374800
H	9.65364700	11.09038700	-0.76779500
H	8.39922200	10.99195700	0.47606500
C	10.81743100	10.67173500	1.70717000
H	10.10101400	11.22533400	2.31693500
H	11.35920400	11.38976900	1.09057500
H	11.53000000	10.16838100	2.35654800
C	11.05125100	8.82820700	-0.01036800
H	11.76976100	8.31730500	0.62624800
H	11.59784900	9.46692200	-0.70513300
H	10.49897500	8.09020600	-0.59403500
C	8.13250000	3.93093800	4.14355100
C	8.45034700	1.82440900	2.94086900
C	7.03312100	1.75575700	2.39269700
H	6.34134900	1.41604600	3.16520400
H	7.00536500	1.03335700	1.57627500
H	6.70117000	2.71836100	2.00974400
C	8.89817200	0.47074500	3.46525300
H	9.89698000	0.53439300	3.89750600
H	8.92276300	-0.24751300	2.64587300
H	8.20904500	0.10251100	4.22557900
C	9.44551800	2.37301000	1.93049700
H	9.12296100	3.32923900	1.52542800
H	9.53765800	1.66434400	1.10682100
H	10.42982800	2.48747200	2.38678100
C	4.19579100	6.29333900	3.44655100
C	2.65159300	5.29622100	1.83820600
C	1.81013600	4.55794800	2.86977000
H	2.32326500	3.65290100	3.20017500
H	0.86410000	4.26177800	2.41479300
H	1.59831900	5.18577700	3.73221100
C	2.01772200	6.61156700	1.40903300
H	1.81098300	7.25025200	2.26475300
H	1.07865400	6.40477500	0.89410300
H	2.67419000	7.14064800	0.71641400
C	2.92491600	4.41027200	0.63279000
H	3.56511000	4.92398700	-0.08492400
H	1.98595600	4.15732100	0.14007900
H	3.41506300	3.48498800	0.93823100

DFT ωB97XD / Def2TZVP, gas phase		
Total electronic energy=	-1776.80361	E_0
Sum of electronic and zero-point Energies=	-1776.073294	$E_0 + E_{ZPE}$
Sum of electronic and thermal Energies=	-1776.034880	$E_0 + E_{tot}$
Sum of electronic and thermal Enthalpies=	-1776.033936	$E_0 + H_{corr}$
Sum of electronic and thermal Free Energies=	-1776.142948	$E_0 + G_{corr}$
Zero-point correction *(unscaled)* =	0.730316	
Number of imaginary vibrational frequencies = 1; i64		
